# “Which comes first”: Religious/spiritual engagement or health? Initial observations from longitudinal analyses

**DOI:** 10.1371/journal.pone.0320410

**Published:** 2025-05-07

**Authors:** Salman S. Ahmad, Zachary T. Goodman, Emily Hylton, Gail Ironson

**Affiliations:** 1 Department of Psychology, University of Miami, Coral Gables, Florida, United States of America; 2 Veterans Affairs San Diego Healthcare System, San Diego, California, United States of America; 3 University of California, San Diego, San Diego, California, United States of America; 4 Atlanta Veterans Affairs Medical Center, Atlanta, GeorgiaUnited States of America; 5 Department of Psychiatry, University of Miami, Coral Gables, Florida, United States of America; Emory University, School of Public Health, UNITED STATES OF AMERICA

## Abstract

**Objective:**

Religious and spiritual (R/S) engagement is generally associated with better health. However, it is not known which comes first between R/S engagement and health due to a lack of longitudinal studies. We examined this issue in a sample assessed six years apart.

**Methods:**

Using a large nationwide sample (*N* = 3,010 at Wave 1; *n* = 607 at Wave 2) and structural equation modeling (SEM), we developed composite latent measures of R/S engagement and self-rated physical health (SRH). R/S engagement included identity, prayer, commitment, attendance, and coping. SRH included two subjective ratings of health alongside number of chronic illnesses. We examined the measurement invariance and reciprocal relationships of R/S engagement and SRH at two timepoints (six years apart), and controlled for multiple demographic variables (age, sex, education, income, race/ethnicity) as well as R/S engagement or SRH at Wave 1. We then assessed whether the strength of their relationships with each other differed.

**Results:**

Higher R/S engagement at Wave 1 significantly predicted better SRH at Wave 2 (β =.07, *b* = 0.09, SE = 0.04, *p* =.026), whereas higher SRH at Wave 1 did not significantly predict higher R/S engagement at Wave 2 (β =.02, *b* = 0.03, SE = 0.03, *p* =.224); however, such associations were not significantly different. Post-hoc weighted analyses indicated the findings were driven by older participants.

**Conclusion:**

Our findings demonstrate that R/S engagement predicts better SRH six years later, whereas better SRH does not significantly predict future R/S engagement. Future areas of growth in the R/S and health research field include addressing heterogeneity in the measurement of both constructs, increasing sample diversity/representation, and considering contextual nuances. Experimental methods or multiple-timepoint data, with a focus on mediators (e.g., inflammation), may help further disentangle the longitudinal relationships between R/S engagement and self-rated health.

## Introduction

In Western societies, religion and spirituality (R/S) generally demonstrate robust associations with better physical health [1–3]. This is thought to occur for various reasons, ranging from better adherence to exercise and healthy diets [4] and better self-control that leads to lower smoking [5] and substance use [6], to better social support [7] and resources for coping during difficult times [8]. At the same time, health difficulties are also known to interfere with R/S engagement, such as experiencing a decreased ability to attend religious services [9]. On the other hand, health difficulties may enhance R/S engagement, as some individuals are more likely to pray to cope with their difficulties [10–13]. Thus, the direction of causality between R/S and health is still not clear. That is, do individuals who engage with R/S have better health as a result, or do individuals with better health have a greater tendency to engage with R/S? The convincing literature on the relationships between R/S and better health begs further exploration of this question. Given this fundamental gap in the literature, the aim of this study is to disentangle three possibilities: 1) Does R/S lead to better health outcomes?; 2) are healthy people more likely to engage in R/S?; and 3) is there a reciprocal relationship between R/S and health? Although differentiating among these scenarios is challenging, research in this field awaits a resolution of these issues.

### Religion/Spirituality and health

Research on the relationship between R/S and health often focuses on the relationship between church attendance and health [14], but this narrow orientation overlooks the fact that religion and spirituality are vast multidimensional constructs. So far, progress toward disentangling “which comes first” has been hampered by the piecemeal approach that has been adopted when studying various dimensions of R/S and health. Consequently, attempts to move the literature forward must adopt broader conceptualizations of the religion/spirituality domain. Many variables reflect engagement with R/S, including but not limited to, identity (i.e., religious, spiritual, both, or neither), strength of commitment to a particular faith or belief system, service attendance, prayer and other meditative or contemplative practices, the use of R/S for coping and support, and importance of R/S in daily life. In practice, many religious/spiritual beliefs and behaviors have been studied individually and in combination with others, such as prayer, meditation, and bible study (e.g., [15]), when investigating the benefits of R/S. Further, although many aspects of R/S have shown to be related to better health, others such as spiritual struggles (i.e., struggling with one’s faith, and strained relationships with the divine and/or other congregants), or viewing one’s God or deity as a punishing entity, are associated with worse health [16,17]. Clearly, longitudinal research between multiple dimensions of R/S and health is needed to address this long-standing problem [11,12].

Perhaps some of the most illuminating research on R/S and health may be found in research on R/S and mortality. So-called mortality studies convey a certain advantage when it comes to specifying the direction of causality. For example, Chida and colleagues’ [18] meta-analysis observed a 20–25% reduction in mortality for initially healthy individuals who reported greater R/S in its many and multidimensional forms. Although their analyses did not observe significantly lower mortality in studies of individuals with a diagnosed disease, there are a few studies showing this. For some examples, in a nationwide study of individuals with chronic illness, we previously observed that individuals who prayed daily or more often were more likely to survive over six years [19]. In another study, we found that use of spirituality to cope with traumatic events predicted greater survival over 17 years in people living with HIV [20]. In a sub-study using the same sample, we found that individuals living with HIV who prayed for known others (vs. for unknown others or for themselves) were more likely to survive over 17 years [21]. Thus, the literature supporting greater survival in individuals with an illness or disease who engage with R/S is less robust relative to studies with initially healthy individuals, but is growing (for a review, see [2]). However, survival is not bi-directional (i.e., we cannot further assess someone who did not survive, or can only conduct prospective studies for those individuals). This limits our ability to answer the question of which has a stronger effect between R/S and health outcomes, and in turn the question of which comes first.

### Religion/spirituality and self-rated health

Although substantial contributions may be found in research on R/S and mortality, a link in this relationship has not been sufficiently fleshed out. Most deaths come from progression of chronic disease and the accumulation of comorbidities (with the exception of most deaths due to accidents or violence). Consequently, focusing on health rather than mortality per se helps to bring the underlying mechanisms between the two into sharper focus. Numerous studies have longitudinally explored the relationships between baseline R/S and specific non-fatal serious illnesses such as HIV [16] at follow-up (for a review, see [3]). But health is also a vast multidimensional construct in its own right. For example, there are many variations in the risks associated with specific illnesses, and not all diseases have biological markers that can determine severity or disease progression. This also makes it harder to establish the temporal sequence and strength of the relationships between R/S and health. Fortunately, the work of other investigators helps clarify this endeavor.

One indicator of health that can be measured bi-directionally and is strongly related to both R/S and mortality [22] is self-rated health (SRH). SRH has been commonly used in the literature since at least the 1940’s [23]. It is often assessed via the single item: “How would you rate your overall health at the present time?”, with participants rating this item on a Likert-scale with options ranging from “*Poor*” to “*Excellent*.” However, some researchers (e.g., [24]) have combined multiple items to develop an index of SRH, such as by asking participants to rate: 1) their overall health, 2) their health compared to others their own age, and 3) their overall satisfaction with their health, among other indicators of overall wellbeing. SRH is a unique variable that is strongly influenced by cognitions, contexts, and culture. Moreover, it is at times a stronger predictor of mortality than many objective and subjective measures of health [25].

Jylhä [25] explored potential reasons for the strength of SRH as an indicator of health, as well as its limitations. Of note, SRH encompasses biological, psychological, and social pathways since an individual is asked to assess their overall wellbeing. This may cover sensations and bio-psycho-social dysregulations only familiar to the individual (or conversely, those not captured by other assessments), whether consciously (e.g., stress) or unconsciously (e.g., inflammation). At the same time, limitations of SRH include its subjectivity, such that individuals answering this question often focus on different frames of reference (e.g., a particular aspect of their health over others) that may vary as a function of their age or race/ethnicity [26]. Further, for the same level of health status, lower-educated groups often report poorer levels of perceived health (e.g., [27,28]). Despite its inherent limitations, SRH is a valuable marker of health that can be leveraged when exploring relationships between R/S and health.

Many researchers have explored the relationships between R/S and SRH, and have done so in a number of ways (e.g., [22,23,29]). From a review of this growing literature, it is apparent that there are contextual nuances in how R/S predicts SRH that often emerge as a function of the sample and how both R/S and SRH are measured. For some examples, McCullough and Laurenceau [23] studied an American sample that was followed for more than 59 years, and observed that greater religiousness (assessed via a four-item composite) predicted better SRH (single item) in women, but not in men. In a sample of geriatric outpatients, Daaleman and colleagues [30,31] found that spirituality, but not religiosity, predicted better SRH. Lastly and most notably, Stavrova [22] utilized a large-scale cross-national sample of 85,748 individuals, and observed that religious attendance, identity, and importance (in a composite measure) were associated with better SRH (single item) in countries where religiosity was the social norm, including regions within the United States.

To further explore these questions and to reduce error, it may help to combine multiple indicators of R/S that have demonstrated associations with better health, thus developing an underlying factor of this multidimensional construct that is both quantitatively/empirically and theoretically justified. Likewise, some of the limitations of SRH may also be overcome by using multiple indicators of subjective health and considering them alongside more objective reports of health (e.g., number of chronic illnesses). The current study investigated these possibilities.

### The current study

Although the literature suggests that greater engagement with R/S is associated with better health, the direction of causality between the two is unclear (i.e., which comes first?). As a first step toward answering this question, the purpose of this study was to determine whether greater R/S engagement predicted better SRH, whether better SRH predicted greater R/S engagement, and which relationship was stronger between the two (or whether the relationship was reciprocal), in a nationwide sample from the United States that was assessed at two timepoints over six years. Rather than assessing many variables, we first developed underlying factors of both R/S engagement and SRH by combining indicators that theoretically and empirically fit within each construct. We subsequently tested for longitudinal measurement invariance to determine whether the constructs were stable over time. Within the structural equation modeling (SEM) framework, we then evaluated our overarching question of which predicts the other. We next constrained the associations to answer our secondary question of which has a stronger relationship on the other. As we were assessing these questions in a sample from a region generally considered religious (i.e., the United States), following Stavrova [22] we hypothesized that both R/S engagement and SRH would positively predict each other. We further hypothesized that the association of the underlying factor of R/S engagement on SRH would be stronger than the association of SRH on R/S engagement due to our R/S engagement construct reflecting personal, social, as well as coping resources.

## Materials and methods

### Participants

This study recruited participants from two waves of a nationwide (United States), face-to-face, random probability survey of individuals aged 18 and older (the Landmark Spirituality and Health Survey, and the Landmark Spirituality and Health Survey Follow-up). The National Opinion Research Center (NORC) conducted baseline in-person interviews for Wave 1 (*N* = 3,010) from 3/21/2014 to 10/18/2014, via clustered random household sampling using NORC’s national sampling frame from postal address lists. We aimed for a nationally representative sample with an oversampling of older adults in order to have similar representations of young (18–40 years old, *n* = 1,000), middle aged (41–64 years old, *n* = 1,002), and older populations (65 years and older, *n* = 1,008). Possibly due to participant self-selection, relative to 2010 US census data, our sample included fewer male (42.8% vs. 49.2% in the US) and White (66.6% vs. 72.4% in the US) participants, and reasonably representative Black (13.8% vs. 12.6% in the US) and Hispanic (15.7% vs. 16.3% in the US) participants compared to the general US population. There was a 50% response rate for Wave 1 in this study.

Harris Insight conducted follow-up phone interviews for Wave 2 (*n* = 615) from 10/2/2019 to 12/31/2020. Participants at Wave 1 provided various contact information for the follow-up study, including a permanent address (*n* = 2,996), phone number (*n* = 2,861), e-mail address (*n* = 1,695), and/or names and phone numbers for up to two secondary contacts (*n* = 1,438). When participants were recontacted by phone, phone numbers for 730 participants were either disconnected (*n* = 463) or wrong numbers (*n* = 267). Of the remaining 2,144, about half (*n* = 1,058) of the participants did not answer nor respond to their answering machine after 10 attempts, some refused to participate (*n* = 346), household members indicated 22 participants were uninterested or unavailable, and 56 participants reported health problems that prevented their participation. National Death Index (NDI) data was used to identify participants who had died (*n* = 226), including some participants that we were unable to recontact. In total, 615 individuals participated in the follow-up interview and had at least one of the variables used in this study, and 607 completed it. Accounting for those who died, did not have working phone numbers, or did not provide phone numbers (*n* = 136), the response rate for those recontacted for follow-up was 32.06% (615/1,918). Of note, Wave 2 interviews were conducted during the beginning of the COVID-19 pandemic and during the 2020 U.S. election cycle. This may have impacted availability and willingness of participants to answer phone calls from an unfamiliar number.

Covariates used in our analyses (i.e., age, sex, education, income, and race/ethnicity) were taken from the Wave 1 survey, although some (e.g., age) were assessed at both timepoints. See Table 1 for a demographic breakdown of participants at both timepoints, including information on Wave 1 participants who also participated at Wave 2 (i.e., Wave 1 subset; some information is repeated as participants’ demographic data was only collected at Wave 1).

**Table 1:** Demographic characteristics of study participants at Waves 1 (N = 3010) and 2 (n = 607) including Wave 1 data from those who also participated in Wave 2 (Wave 1 subset).

Variable	Wave 1 All	Wave 1 Subset	Wave 2
	*n* (%)	*n* (%)	*n* (%)
Male^a^	1287 (42.76%)	266 (43.82%)	–
Race/Ethnicity^a^			
African American	411 (13.76%)	57 (9.39%)	–
Hispanic	468 (15.67%)	68 (11.20%)	–
White	1990 (66.62%)	462 (76.11%)	–
Other	118 (3.95%)	20 (3.29%)	–
Religion^a^			
Catholic or Roman Catholic	603 (20.09%)	131 (21.30%)	–
Protestant	970 (32.32%)	207 (33.66%)	–
Jewish	48 (1.60%)	15 (2.44%)	–
Muslim	19 (0.63%)	3 (0.49%)	–
Buddhist	39 (1.30%)	12 (1.95%)	–
Hindu	7 (0.23%)	1 (0.16%)	–
Other	757 (25.22%)	125 (20.33%)	–
No religious preference	368 (12.26%)	67 (10.89%)	–
Agnostic	128 (4.27%)	35 (5.69%)	–
Atheist	62 (2.07%)	19 (3.09%)	–
Religious/Spiritual Identity			
Neither religious nor spiritual	258 (8.68%)	54 (8.90%)	69 (11.37%)
Either religious or spiritual	1089 (36.65%)	206 (33.94%)	214 (32.26%)
Both religious and spiritual	1624 (54.66%)	347 (57.17%)	324 (53.38%)
Prayer Frequency			
Did not pray	357 (11.94%)	94 (15.46%)	98 (16.17%)
Less than once a month	141 (4.72%)	32 (5.26%)	30 (4.95%)
Once a month	66 (2.21%)	9 (1.48%)	20 (3.30%)
A few times a month	154 (5.15%)	27 (4.44%)	33 (5.45%)
Once a week	80 (2.68%)	17 (2.80%)	18 (2.97%)
A few times a week	377 (12.61%)	63 (10.36%)	75 (12.38%)
Daily	697 (23.31%)	125 (20.56%)	132 (21.78%)
More than once daily	1,118 (37.39%)	241 (39.64%)	200 (33%)
Religious Service Attendance			
Never	550 (18.40%)	95 (15.57%)	174 (28.71%)
Less than once a year	286 (9.57%)	64 (10.49%)	46 (7.59%)
About once or twice a year	379 (12.68%)	75 (12.30%)	55 (9.08%)
Several times a year	320 (10.71%)	66 (10.82%)	43 (7.10%)
About once a month	142 (4.75%)	18 (2.95%)	21 (3.47%)
2 to 3 times a month	251 (8.40%)	51 (8.36%)	32 (5.28%)
Nearly every week	234 (7.83%)	54 (8.85%)	46 (7.59%)
Every week	574 (19.20%)	122 (20%)	133 (21.95%)
Several times a week	253 (8.46%)	65 (10.66%)	56 (9.24%)
Religious Commitment (“My faith shapes how I think and act every day”)			
Strongly Disagree	138 (4.63%)	42 (6.91%)	47 (7.74%)
Disagree	371 (12.45%)	68 (11.18%)	37 (6.10%)
Neutral	72 (2.42%)	11 (1.81%)	95 (15.65%)
Agree	1,317 (44.21%)	252 (41.45%)	191 (31.47%)
Strongly Agree	1,081 (36.29%)	235 (38.65%)	237 (39.04%)
Self-rated health 1: Rate your overall health at present			
Poor	99 (3.29%)	11 (1.79%)	15 (2.44%)
Fair	584 (19.43%)	85 (13.84%)	110 (17.89%)
Good	1,723 (57.34%)	358 (58.31%)	345 (56.10%)
Excellent	599 (19.93%)	160 (26.06%)	145 (23.58%)
Self-rated health 2: Compared to most people your age			
Worse	269 (9.01%)	49 (8.06%)	55 (8.96%)
About the same	1371 (45.91%)	247 (40.63%)	251 (40.88%)
Better	1346 (45.08%)	312 (51.32%)	308 (50.16%)
Variable	*M* (*SD*)	*M* (*SD*)	*M* (*SD*)
Benevolent R/S Coping (RCOPE)	2.92 (1.96)	2.74 (2.08)	2.77 (2.12)
Seeking Social Support (RCOPE)	4.14 (2.03)	3.95 (2.19)	3.88 (2.17)
Number of Chronic Illnesses	1.60 (1.75)	1.43 (1.60)	1.76 (1.62)
Age	51.64 (19.28)	51.80 (17.50)	57.97 (17.48)
Education in Years^a^	13.47 (3.16)	14.80 (3.07)	–
Variable	Median	Median	Median
Annual Household Income^a^	$30,000-$39,999	$40,000-$59,999	–

Wave 1 Subset reports Wave 1 data on participants who were also in Wave 2. Ranges for the two RCOPE measures = 0–6, for Number of Chronic Illnesses = 0–12. Some variables at Wave 2 (e.g., Self-rated health Item 1) had up to 615 responses, but 607 completed the interview. Denominators in percentage calculations were total numbers of participants who responded to the specific item. For example, 23 participants refused to report their race/ethnicity and were thus not included in percentage calculations for that variable.

^a^Sex, race/ethnicity, education, religion, and income were assessed only at Wave 1.

### Procedure

All study procedures were approved by NORC’s Institutional Review Board (IRB) for Wave 1 and by the University of Miami’s Institutional Review Board (IRB) for Wave 2. Informed consent was received by NORC at Wave 1 via a written consent form that was handed to participants to be read and signed, and informed consent at Wave 2 was received by Harris Insight’s team by verbally reading the consent script over the phone to participants, who were given the choice to decline or proceed to the interview. Consent was then documented on interview forms. This study was registered with the Open Science Foundation (https://doi.org/10.17605/OSF.IO/TS92Q).

For Wave 1, each in-person interview consisted of an informed consent form and questions that assessed participants on a number of variables of interest in this project, including physical and mental health (including health behaviors and other aspects of wellbeing) and religion/spirituality. In addition, physical measurements (e.g., blood pressure; an optional finger stick for blood spot analysis) were also taken during this meeting. Questions were asked in a logical format, such that certain answers led to follow-up questions, whereas others did not. This impacted parts of the religion/spirituality survey, such as in the case of religious/spiritual coping, where only individuals who reported in an earlier question that they had experienced a serious and stressful event over the last 18 months (*n* = 2,225) were asked whether they used religion/spirituality to cope with that stressor. Participants were compensated $25 at Wave 1 and $50 at Wave 2 for taking part in the study. Further details of the sampling procedures can be found in Krause et al. [32]. For Wave 2, participants were first called by phone, followed by being called at their listed secondary contacts, and then emailed to participate in the follow-up. After 10 phone attempts to reach them, they were considered “no longer interested.” The Wave 2 phone interview consisted of verbal consent and a subset of questions with some modifications from the Wave 1 in-person interview.

### Measures

#### Demographics.

Participants were asked a number of demographic questions related to their backgrounds. Age was calculated by asking participants their date of birth, and subsequently recording their age in years. Participants’ sex was determined by observation and was delineated as male or female. Education was determined by asking participants to report the highest grade or level of regular school they attended or attempted, and was recorded as number of years of education (between 1–20+; GED was considered 12 years). Annual household income was assessed by showing participants a card with ranges of yearly incomes, and then asking them to select the group their family’s total income fell into (from all sources, before taxes, in the past year). Regarding race/ethnicity, participants were asked if they were of Hispanic origin or descent, and also if they considered themselves White, Black, Asian, or something else (and to specify if so). We created two dummy coded variables for race/ethnicity to use as covariates: Hispanic (including Hispanic Black) vs. everyone else, and Black (non-Hispanic) vs. everyone else.

#### Self-rated health (SRH).

Self-rated health (SRH) was determined via a composite measure of three items asked at both time points. Following previous work, this included the item: “How would you rate your overall health at the present time?”, with response options on a 4-point Likert scale (0 [*Poor*] to 3 [*Excellent*]). This was initially followed by two additional items: “Would you say your health is better, about the same, or worse than most people your age?” and “Do you think your health is better, about the same, or worse than it was a year ago?”, that participants answered on a 3-point Likert scale (0 [*Worse*] to 2 [*Better*]). However, as described in the results, the latter item (rate your health compared to one year ago) was removed due to a low factor loading. We separately analyzed data with this item included (see Appendix in S1 File), and our results did not change. The third item asked participants to report the number of chronic illnesses they experienced in the past 12 months (Wave 1) or the past 5 years (Wave 2). These were assessed from a list of 12 chronic illness categories [33] that included cardiovascular diseases, diabetes, cancers/malignant tumors, eye diseases, and “other major health problem” (see S2 File). Number of chronic illnesses was reversed (i.e., number of illnesses not endorsed). Thus, higher scores indicated better SRH. See the results section for a breakdown of our measurement model of SRH, with standardized factor loadings and measurement error terms displayed in S1 Fig.

#### Religious and spiritual (R/S) engagement.

Our composite measure of religious and spiritual (R/S) engagement consisted of five categories of intrinsic (i.e., personal or subjective) R/S that were assessed at both time points: identity, frequency of service attendance, frequency of private prayer, commitment, and religious coping. Religious and spiritual identity was assessed by asking participants to select the statement that best described them: “I am spiritual and religious,” “I am spiritual but not religious,” “I am religious but not spiritual,” and “I am neither spiritual nor religious.” Participants were coded as 0 (neither spiritual nor religious), 1 (either spiritual or religious), or 2 (both spiritual and religious). Frequency of private prayer was assessed by asking participants: “How often do you pray by yourself?”, with responses on an 8-point scale (0 [*Never*] to 7 [*Several times a day*]) and higher scores indicating greater frequency of private prayer. Religious service attendance was determined by asking participants: “How often do you attend religious services?”, with answers on a 9-point scale (0 [*Never*] to 8 [*Several times a week*]) and higher scores indicating greater religious service attendance. Religious commitment was assessed via a single item from Benson and colleagues’ [34] Faith Maturity Scale (FMS): “My faith shapes how I think and act each and every day,” with responses on a 5-point Likert scale (0 [*Strongly disagree*] to 4 [*Strongly agree*]).

Two forms of religious coping were assessed using subscales from Pargament and colleagues’ [35] measure of religious coping (RCOPE) that were answered on a 4-point Likert scale (0 [*Not at all*] to 3 [*A great deal*]). The Benevolent Religious Coping subscale assesses the degree to which an individual finds meaning from stressful situations using religious methods, including redefining the stressor as an opportunity for spiritual growth. This subscale consisted of two items at Wave 1 (α =.74, *n* = 2,173) and Wave 2 (α =.78, *n* = 574) that assessed to what extent a participant utilized benevolent religious coping when dealing with the most stressful event they faced in the last 18 months (i.e., “Saw my situation as part of God’s plan” and “Tried to see how God might be trying to strengthen me in this situation”). The Seeking Social Support subscale assesses the degree to which an individual seeks comfort and reassurance through God’s love and care. This subscale also consisted of two items (i.e., “Trusted that God would be by my side” and “Looked to God for strength, support, and guidance”) that demonstrated adequate internal reliability at Wave 1 (α =.93, *n* = 2,191) and Wave 2 (α =.93, *n* = 574). We renamed the Seeking Social Support scale “Seeking God’s Social Support” to avoid confusion with common social support measures. See the results section for more information on our measurement model of R/S engagement. Standardized factor loadings and measurement error terms are also displayed in S1 Fig.

### Statistical analysis

Preprocessing of the data was conducted in IBM SPSS Statistics Version 28 [36]. The statistical program R [37] was used to analyze data in this study, including to test the measurement and structural models of R/S engagement and SRH. Skewness and kurtosis were assessed using Kline’s [38] criteria (|Skew| > 3 and |Kurtosis| > 8) to determine whether assumptions of normality had been met. Pearson’s correlations were used to determine significant associations between study variables. Preliminary analyses also consisted of performing a sample attrition analysis on the Wave 1 participants who did not attend at Wave 2 via *t*-tes*t*s across demographic and other study variables. No weighting or other manipulation was performed in our analyses.

We first developed a measurement model (that assesses the relationships between sets of latent and observed variables [39]) of both R/S and SRH to determine an adequate fit to the data by confirmatory factor analysis (CFA). We estimated parameters using maximum likelihood estimation with robust (Hubert-White) standard errors (i.e., MLR), and missing data was handled with Full Information Maximum Likelihood (FIML). Standardized factor loadings (λ) above |.40| were considered adequate. Parameters of model fit were interpreted using Hu and Bentler’s [40] recommendations for good fit (χ^2^ *p* value >.05; CFI >.95; RMSEA <.06; and SRMR <.08), and robust CFIs and RMSEAs were reported. We also assessed residual (co)variances to guide model modifications, such as whether to correlate errors between certain indicators. We first analyzed a measurement model of R/S engagement at Wave 1 and then at Wave 2, before conducting a CFA to assess the measurement model of R/S engagement at both time points. We used the same approach when developing the measurement model of SRH. Finally, we assessed a complete measurement model consisting of both R/S engagement and SRH at both time points.

Next, we tested measurement invariance of R/S engagement and SRH over time. The complete model was first identified by constraining latent variable means and standard deviations to 0 and 1, respectively (i.e., they were standardized), to allow for examination of all factor loadings. We assessed three nested levels (i.e., metric, scalar, residual) of invariance. Metric invariance was tested by constraining each indicator’s factor loading to be equal with its respective duplicate at both time points. Scalar invariance was subsequently tested by constraining indicator intercepts as equal and allowing the latent mean at Wave 2 to vary (to allow comparisons over time), while maintaining any equality constraints in factor loadings suggested by our tests of metric invariance. Residual invariance was tested by constraining the error variances as equal between indicators at both time points, again while retaining any constraints from the scalar model. The chi-square (χ^2^) statistic was used to assess differences in levels of invariance between the more constrained model and the prior model, with a significant chi-square difference (Δχ^2^) in fit between models suggesting significantly worse fit between nested models. Decreases in CFI ≥.01 and increases in RMSEA ≥.01 were also used to confirm decisions regarding meaningful differences in fit [41].

For cases of measurement non-invariance, we explored partial measurement invariance by freeing newly constrained parameters one-at-a-time, and comparing each model to its more constrained version, thus identifying parameters that produced the greatest misfit when constrained. The presence of partial invariance was indicated by non-significant and non-meaningful changes in fit between a model with its previously constrained model. For example, items with non-invariant factor loadings from partial metric invariance would not have their intercepts constrained in the subsequent scalar and residual models [42].

After confirming measurement invariance, we examined the latent variables in a structural equation model (SEM). We included covariates (i.e., age, sex, education, income, non-Hispanic Black, and Hispanic) within our model when determining whether the structural model fit the data and testing the relationships between R/S engagement and SRH. We initially faced estimation difficulties due to high variances between age and other variables, so we transformed age (dividing by 10) to address this issue.

To answer our first question of whether R/S engagement and SRH predict each other, we used the SEM framework to determine whether R/S engagement at Wave 1 predicted SRH at Wave 2, while controlling for R/S engagement and covariates at Wave 1, as well as whether SRH at Wave 1 predicted R/S engagement at Wave 2, while controlling for SRH and covariates at Wave 1. To test for statistical differences in coefficients, and thus address our second research question, we constrained the regression coefficient between R/S engagement at Wave 1 to SRH at Wave 2 to be equal to the coefficient between SRH at Wave 1 to R/S engagement at Wave 2. We compared whether this model differed from the model in which they were unconstrained via a likelihood ratio test. If constraining them significantly or noticeably decreased fit, this would suggest the relationships were different.

On a post-hoc basis, we re-analyzed our findings using sampling weights provided by NORC that accounted for the oversampling of older adults (we intentionally recruited ~1,000 older adult, middle aged, and younger adult participants) and other sampling differentials, yielding valid sample estimates. We also explored the reasons behind any differences we observed. The weights were calculated as follows. All selected households from the national frame received a base weight that reflected their probability of selection. An adjustment for household eligibility status was applied to the base weights to account for households with unknown eligibility. The base weights were further adjusted to account for screening nonresponse, subsampling, special cases that were completed in the pulled-back release, probability of selection for persons within a selected household, middle aged cases that were turned back on in subsampled out younger adult/middle aged households, main interview nonresponse, younger adult/middle aged member completes that were worked due to random error, and scaling to sample totals. Weighting details can be seen in Section 5 of S3 File.

## Results

### Preliminary analyses

The independent and dependent variables satisfactorily met the assumption of normality (skewness ≥ -1.16 and ≤ 1.25; kurtosis ≥ 1.36 and ≤ 4.42). Table 2 displays a correlation matrix of all study variables. Of note, most indicators of a latent variable were significantly correlated with each other, and all indicators at Wave 1 were significantly correlated with their respective duplicate indicator at Wave 2. However, most indicators of R/S engagement were not significantly associated with indicators of SRH. Further, our covariates (including Black vs. everyone else, and Hispanic vs. everyone else) were significantly correlated with various indicators, confirming the utility of their inclusion within subsequent analyses.

**Table 2:** Correlations among study variables.

Variable	1	2	3	4	5	6	7	8	9	10	11	12	13	14	15	16	17	18	19	20
1. Identity w1	–																			
2. Prayer w1	.60**	–																		
3. Attendance w1	.49**	.50**	–																	
4. Commit w1	.49**	.56**	.41**	–																
5. Benevolent w1	.36**	.48**	.36**	.38**	–															
6. Support w1	.50**	.68**	.45**	.54**	.68**	–														
7. SRH 1 w1	-.01	-.05*	.05*	.02	-.05^	-.06**	–													
8. SRH 2 w1	.06**	.01	.08**	.08**	-.04	-.03	.46**	–												
9. SRH 3 w1	.00	.01	.03	.04^	.04	-.00	.24**	.21**	–											
10. Illnesses w1	.12**	.14**	.06*	.09**	.04	.13**	-.44**	-.21**	-.17**	–										
11. Identity w2	.69**	.62**	.53**	.48**	.40**	.53**	-.01	-.05	-.01	.11*	–									
12. Prayer w2	.63**	.80**	.56**	.57**	.48**	.67**	-.01	-.07	-.02	.16**	.69**	–								
13. Attendance w2	.50**	.52**	.75**	.42**	.36**	.47**	-.04	-.04	-.01	.15**	.57**	.58**	–							
14. Commit w2	.56**	.60**	.51**	.57**	.42**	.55**	-.07	-.10^	.06	.10^	.58**	.65**	.51**	–						
15. Benevolent w2	.34**	.51**	.45**	.39**	.52**	.53**	.01	-.01	.02	.07	.47**	.56**	.48**	.49**	–					
16. Support w2	.56**	.69**	.53**	.56**	.55**	.72**	.03	-.03	.02	.12*	.67**	.74**	.57**	.61**	.70**	–				
17. SRH 1 w2	-.01	-.02	.03	-.00	.03	-.00	.55**	.33**	.07	-.35**	.00	.05	.01	.06	-.01	.04	–			
18. SRH 2 w2	.06	.09^	.10^	.08^	.04	.10^	.33**	.53**	.00	-.16**	.08	.09^	.13*	.13*	.09^	.14**	.49**	–		
19. SRH 3 w2	-.04	-.06	-.00	-.09^	.04	-.00	.03	.08	.06	-.17**	-.05	-.04	.02	-.05	-.02	.02	.23**	.20**	–	
20. Illnesses w2	.12*	.11*	.05	.11*	-.07	.08	-.31**	-.18**	.00	.65**	.09^	.10^	.09^	.08^	.01	.04	-.45**	-.23**	-.16**	–

w1 = Wave 1; w2 = Wave 2. Identity = Religious/Spiritual identity; Prayer = Frequency of private prayer; Attendance = Frequency of religious service attendance; Commit = Religious commitment; Benevolent = Benevolent Religious Coping (RCOPE); Support = Seeking God’s Social Support (RCOPE); SRH 1 = Self-rated health item 1; SRH 2 = Self-rated health item 2; SRH 3 = Self-rated health item 3; Illnesses = Number of chronic illnesses (not reverse-scored).

^*p* <.05, two-tailed.

*p <.01, two-tailed.

**p <.001, two-tailed.

Sample attrition analyses revealed that Wave 1 participants who did not participate at Wave 2 had reported higher R/S coping, including benevolent religious coping (Wave 1 only *M* = 2.97, Wave 2 *M* = 2.74, *t* (2171) = 2.21, *p* =.027, *d* = -.12) and seeking God’s social support (Wave 1 only *M* = 4.18, Wave 2 *M* = 3.95, *t* (2189) = 2.14, *p* =.032, *d* = -.11). Further, Wave 1 participants who did not participate at Wave 2 had reported lower education (Wave 1 only *M* = 13.13, Wave 2 M = 14.78, *t* (3007) = -11.78, *p* <.001, *d* =.53), income (Wave 1 only median = $30,000-$39,999, Wave 2 median = $40,000-$59,999, *t* (2760) = -10.81, *p* <.001, *d* =.51), attendance at religious services (Wave 1 only *M* = 3.71, Wave 2 *M* = 4.00, *t* (2987) = -2.21, *p* =.027, *d* =.10), and poorer self-rated health via item 1 (i.e., rate your overall health; Wave 1 only *M* = 1.90, Wave 2 *M* = 2.09, *t* (3003) = -5.69, *p* <.001, *d* =.26) and item 2 (i.e., compared to others your age; Wave 1 only *M* = 1.34, Wave 2 *M* = 1.43, *t* (2984) = -3.10, *p* =.002, *d* =.14), and a higher number of chronic illnesses (Wave 1 only *M* = 1.64, Wave 2 *M* = 1.43, *t* (3008) = 2.70, *p* =.007, *d* = -.12). The two groups did not significantly differ in age, R/S identity, frequency of prayer, commitment, and self-rated health item 3 (i.e., rate your health compared to one year ago). Recall that participants who died (*n* = 225) would not have been able to participate in Wave 2, which would skew some of these results.

### Measurement model

We first developed our measurement model of R/S engagement at Wave 1, χ^2^ (9) = 347.11, *p* <.001, CFI =.94, RMSEA =.14, SRMR =.04. Residual variances suggested the two RCOPE subscales (benevolent religious coping and seeking social support) were highly intercorrelated. As they are part of the same measure, which likely explains some non-random residual covariance, we correlated errors between these two subscales in a subsequent model, χ^2^ (8) = 79.86, *p* <.001, CFI =.99, RMSEA =.06, SRMR =.02. The fit of this model improved significantly, Δχ^2^ (1) = 328.85, *p* <.001. However, we elected to proceed without correlating errors between these two subscales as the initial model did not indicate any estimation difficulties, the fit was borderline acceptable, and we preferred a more parsimonious measurement model. Next, we developed a measurement model of R/S engagement at Wave 2, which had acceptable fit, χ^2^ (9) = 48.68, *p* <.001, CFI =.98, RMSEA =.09, SRMR =.02.

We then developed a measurement model of SRH at Wave 1, χ^2^ (2) = 34.12, *p* <.001, CFI =.98, RMSEA =.08, SRMR =.02. The factor loading for one item (comparing one’s health to one year ago) was low (λ =.29). Although this item was theoretically and conceptually relevant to the self-rated health construct, we elected to remove it from our measurement model as it did not meet our threshold for adequate reliability. For SRH at Wave 2, the model fit the data well, χ^2^ (2) = 5.99, *p* =.050, CFI =.99, RMSEA =.06, SRMR =.02. However, a similarly low factor loading (λ =.26) was observed for the item asking individuals to rate their health now compared to one year ago. This item was removed here as it was for SRH at Wave 1. We separately confirmed whether our results changed with this item included, and they did not.

We subsequently ran CFAs to assess the longitudinal measurement models of R/S engagement and SRH. The measurement model of R/S engagement at Waves 1 and 2 did not fit the data well, χ^2^ (53) = 797.20, *p* <.001, CFI =.85, RMSEA =.15, SRMR =.06. Residual variances and modification indices suggested that duplicate indicators from Wave 1 and Wave 2 were highly intercorrelated. Thus, we correlated errors between each indicator at Wave 1 with its respective duplicate at Wave 2. This model fit the data well, χ^2^ (47) = 446.56, *p* <.001, CFI =.96, RMSEA =.08, SRMR =.04, with a significant improvement in model fit, Δχ^2^ (6) = 234.56, *p* <.001.

The measurement model of SRH at Waves 1 and 2 also did not initially fit the data well, χ^2^ (8) = 343.11, *p* <.001, CFI =.69, RMSEA =.29, SRMR =.11. We similarly correlated errors between each indicator at Wave 1 with its respective duplicate at Wave 2. This model fit the data very well, χ^2^ (5) = 6.42, *p* =.267, CFI = 1.00, RMSEA =.03, SRMR =.02, with significantly improved fit, Δ*χ*^2^ (3) = 335.25, *p* <.001.

Finally, we assessed a complete measurement model of R/S engagement and SRH at Waves 1 and 2. The fit of this model was acceptable, χ^2^ (120) = 684.99, *p* <.001, CFI =.96, RMSEA =.06, SRMR =.05. However, the correlated errors between SRH item 1 (i.e., the original “self-rated health” item) from Wave 1 and Wave 2 caused model instability. Investigations suggested that as their factor loadings were very high, there was minimal residual variance to covary. Removing that covariance resolved estimation difficulties, while fit indices did not change, χ^2^ (121) = 696.97, *p* <.001, CFI =.96, RMSEA =.06, SRMR =.05. Table 3 displays standardized factor loadings for indicators of both latent variables at both timepoints. R/S engagement at Wave 1 and Wave 2 were highly correlated (*r* =.91, *p* <.001), as were SRH at Wave 1 and Wave 2 (*r* =.73, *p* <.001). R/S engagement at Wave 1 was significantly correlated with SRH at Wave 1 (*r* = -.05, *p* =.021) but not with SRH at Wave 2 (*r* =.02, *p* =.655). Finally, R/S engagement at Wave 2 was not significantly correlated with SRH at Wave 1 (*r* =.01, *p* =.698) nor at Wave 2 (*r* =.05, *p* =.216). This is the final measurement model that we retained for our measurement invariance and subsequently our structural analyses (see S1 Fig).

**Table 3:** Standardized factor loadings of the measurement model latent variables at Waves 1 (N = 3010) and 2 (n = 615).

Indicator	Wave 1		Wave 2	
	R/S Engagement	SRH	R/S Engagement	SRH
Identity	.69		.75	
Prayer	.82		.83	
Attendance	.60		.62	
Commit	.67		.71	
Benevolent	.70		.72	
Support	.87		.90	
SRH 1		.91		.89
SRH 2		.51		.55
Chronic Illnesses		.47		.49

All factor loadings are statistically significant at *p* <.001. Identity = Religious/Spiritual identity; Prayer = Frequency of private prayer; Attendance = Frequency of religious service attendance; Commit = Religious commitment; Benevolent = Benevolent Religious Coping (RCOPE); Support = Seeking God’s Social Support (RCOPE); SRH 1 = Self-Rated Health item 1; SRH 2 = Self-Rated Health item 2; Chronic Illnesses = Number of chronic illnesses (reverse-scored).

### Measurement invariance of R/S Engagement and SRH over time

Following model identification, we first established measurement invariance of SRH over time. The measurement model of SRH at both time points served as the baseline (i.e., configural) model. To assess metric invariance, we constrained the factor loadings of each indicator at Wave 1 to be equal with its respective duplicate at Wave 2, which did not significantly change fit indices, Δχ^2^ (3) = 1.22, *p* =.747. To assess scalar invariance, we constrained item intercepts of duplicate indicators over time to be equal (while allowing the latent mean of SRH at Wave 2 to vary), and this significantly worsened fit, Δχ^2^ (2) = 29.47, *p* <.001, with a meaningful decrease in fit indices (ΔCFI =.02, ΔRMSEA =.04). In order to examine which item(s) produced significant non-invariance over time, we freed each item intercept one-at-a-time. The model that allowed the intercept of the number of chronic illnesses reported by participants to vary across time demonstrated the best fit, and this model was not significantly different from the metric model, Δχ^2^ (1) = 0.34, *p* =.560. The intercept for number of chronic illnesses (reverse-coded) in the metric model was lower in Wave 2 (γ = 8.14, SE = 0.03, *p* <.001) than at Wave 1 (γ = 8.40, SE = 0.06, *p* <.001), suggesting participants with average SRH at Wave 2 had a higher number of chronic illnesses than participants with average SRH at Wave 1. Thus, number of chronic illnesses was not constrained in subsequent models.

Residual invariance was subsequently tested by constraining the error terms for each remaining indicator to be equal across both time points, which did not significantly change fit indices, Δχ^2^ (2) = 0.43, *p* =.806. Finally, we tested for a difference in SRH across time points via examining the significance of the latent variable difference across time points (*M* = -0.03, *SE* = 0.04, *p* =.477), which suggested that participants from Wave 1 who were healthy enough to and agreed to participate at Wave 2 did not differ in overall SRH, after accounting for their increased number of chronic illnesses.

A similar approach was taken to establish the measurement invariance of R/S engagement over time. To assess metric invariance, we constrained the factor loadings to be equal, which did not significantly change fit indices, Δχ^2^ (6) = 12.54, *p* =.051. To assess scalar invariance, we constrained item intercepts to be equal (while allowing the latent mean of R/S engagement at Wave 2 to vary), and this also did not significantly worsen fit, Δχ^2^ (5) = 4.86, *p* =.434. Residual invariance was subsequently tested by constraining the error terms for each indicator to be equal across both time points, which also did not significantly worsen fit indices, Δχ^2^ (6) = 10.41, *p* =.108. Finally, we tested for a difference in R/S engagement across time points via examining the significance of the latent variable difference across time points (*M* = -0.11, *SE* = 0.02, *p* <.001), which suggested that participants reported lower R/S engagement at Wave 2 than at Wave 1.

Finally, we assessed the complete measurement model of R/S engagement and SRH at both time points while maintaining all of the constraints from our previous tests of measurement invariance. This model showed acceptable fit to the data, χ^2^ (144) = 733.12, *p* <.001, CFI =.96, RMSEA =.05, SRMR =.06. In sum, we observed partial measurement invariance between R/S engagement and SRH over time, with the number of chronic illnesses reported by participants at both timepoints the only variable that was non-invariant over time (i.e., number of chronic illnesses increased at Wave 2). This was the model we retained for our structural analyses.

### Structural equation models (SEM)

**Fig 1:**
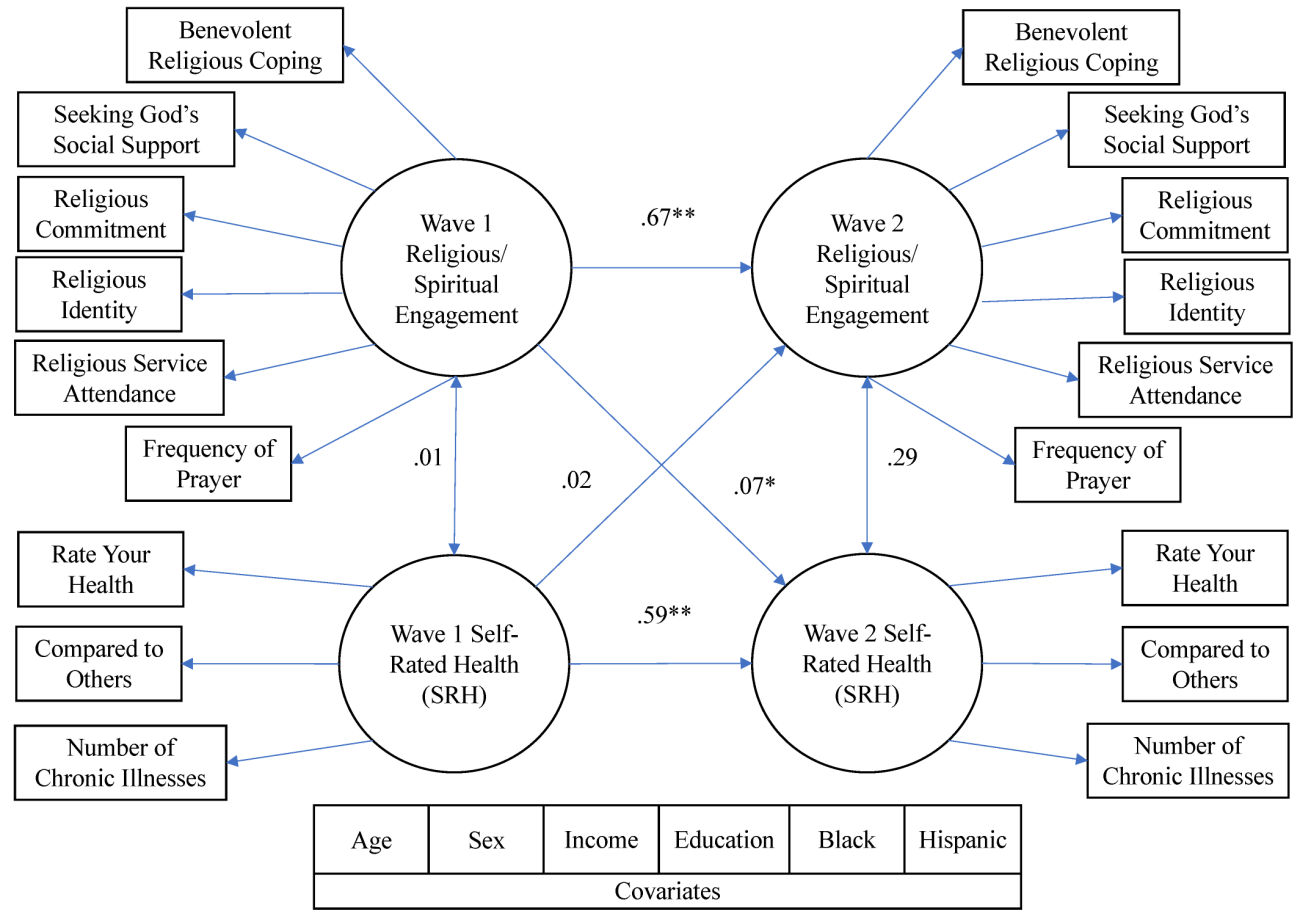
Final structural model investigating the relationships between religious/spiritual engagement and self-rated health after controlling for each other and covariates at Wave 1 (N = 3,010). Paths represent standardized coefficients. Covariates were regressed on all indicators. * *p* <.05. ** *p* <.001.

We analyzed the structural model of R/S engagement and SRH at both timepoints to test the hypothesis that both R/S engagement and SRH would predict each other. Covariates (sex, age, education, income, and whether participants identified as Black or Hispanic) were regressed on all indicators. The model had acceptable fit to the data, χ^2^ (147) = 1002.30, *p* <.001, CFI =.93, RMSEA =.08, SRMR =.07. Higher R/S engagement at Wave 1 predicted better self-rated health at Wave 2 (β =.07, *b* = 0.09, SE = 0.04, *p* =.026), after controlling for better self-rated health (β =.59, *b* = 0.73, SE = 0.04, *p* <.001) and covariates at Wave 1. However, better self-rated health at Wave 1 did not significantly predict higher R/S engagement at Wave 2 (β =.02, *b* = 0.03, SE = 0.03, *p* =.224), after controlling for higher R/S engagement (β =.67, *b* = 0.90, SE = 0.02, *p* <.001) and covariates at Wave 1. Table 4 displays these results. The correlations between R/S engagement and SRH at Wave 1, and between R/S engagement and SRH at Wave 2, were not significant. Fig 1 displays the final structural model tested, and S1 Table displays standardized factor loadings and measurement error terms for our study variables.

**Table 4:** Structural equation modeling (SEM) results (N = 3,010).

	β	*b*	*SE(b)*	*p*	95% CI	_*R*2_
Predicting Self-rated Health at Wave 2						.35
From Self-rated Health at Wave 1	.59	.73	.04	<.001	[.65,.82]	
From Religious/Spiritual Engagement at Wave 1	.07	.09	.04	.026	[.01,.07]	
Predicting Religious/Spiritual Engagement at Wave 2						.45
From Religious/Spiritual Engagement at Wave 1	.67	.90	.02	<.001	[.86,.94]	
From Self-rated Health at Wave 1	.02	.03	.03	.224	[-.02,.08]	

The latent variables were standardized in our analyses, with Wave 2 latent variable intercepts allowed to vary.

### Differences in strength between SRH and R/S Engagement relationships over time

To test the hypothesis that R/S engagement would have a stronger association with SRH over time than SRH would have with R/S engagement over time, we constrained the regression coefficient between R/S engagement at Wave 1 to SRH at Wave 2 to be equal to the coefficient between SRH at Wave 1 to R/S engagement at Wave 2. If the regression coefficients had been different, this would lead to a significant reduction in model fit as our constraint would not have been supported by the data. This constraint did not significantly worsen model fit, Δχ^2^ (1) = 1.48, *p* =.222, suggesting the directional strength of the relationships between R/S engagement and SRH across time did not differ.

### Post-hoc analyses with weighting

We subsequently reanalyzed all data using weighted values that were provided by NORC (see Appendix in S1 File for detailed results). The outcome for measurement models and tests of measurement invariance did not change. However, there were some changes in the structural model, which still had acceptable fit to the data, χ^2^ (147) = 871.30, *p* <.001, CFI =.93, RMSEA =.08, SRMR =.08. Specifically, higher R/S engagement at Wave 1 no longer significantly predicted better self-rated health at Wave 2 (β =.05, *b* = 0.06, SE = 0.04, *p* =.169), after controlling for better self-rated health (β =.60, *b* = 0.75, SE = 0.05, *p* <.001) and covariates at Wave 1. Better self-rated health at Wave 1 still did not significantly predict higher R/S engagement at Wave 2 (β =.02, *b* = 0.03, SE = 0.03, *p* =.305), after controlling for higher R/S engagement (β =.66, *b* = 0.88, SE = 0.02, *p* <.001) and covariates at Wave 1.

To explore why results differed for weighted and unweighted analyses, we split our sample along the median age (52 years) and separately analyzed the datasets. The model using the sample over 52 years of age had borderline acceptable fit to the data, χ^2^ (147) = 502.80, *p* <.001, CFI =.91, RMSEA =.08, SRMR =.09. Consistent with the analysis in the full sample, higher R/S engagement at Wave 1 did significantly predict better self-rated health at Wave 2 (β =.09, *b* = 0.11, SE = 0.05, *p* =.036), whereas better self-rated health at Wave 1 did not significantly predict higher R/S engagement at Wave 2 (β =.04, *b* = 0.06, SE = 0.04, *p* =.185). Also in support of our overall findings, constraining the regression coefficients did not significantly worsen model fit, Δχ^2^ (1) = 0.72, *p* =.397, suggesting the directional strength of the relationships between R/S engagement and SRH across time did not differ. The model using the sample under 52 years of age had acceptable fit to the data, χ^2^ (147) = 548.66, *p* <.001, CFI =.93, RMSEA =.07, SRMR =.08, but found that R/S engagement at Wave 1 did not significantly predict better self-rated health at Wave 2 (β =.05, *b* = 0.07, SE = 0.06, *p* =.266), and better self-rated health at Wave 1 also did not significantly predict higher R/S engagement at Wave 2 (β =.03, *b* = 0.04, SE = 0.04, *p* =.320).

## Discussion

As we noted earlier, a considerable number of studies have examined the relationship between religion and health. However, the wide majority of this research is based on the untested assumption that religion affects health and not the other way around. Consequently, greater confidence in the religion/health relationship calls for more rigorous tests of this assumption. The purpose of this study is to provide some preliminary insight into this issue. Our composite measure of R/S engagement included a number of the most commonly supported R/S variables in the literature (identity, commitment, prayer, attendance, and coping), and our composite measure of SRH also included a relatively more objective report of health (i.e., number of chronic illnesses). Our study is also strengthened by additional design and analytic features, including our use of longitudinal data, a large nationwide sample, tests of strict measurement invariance, and controls for multiple relevant covariates including both R/S engagement and SRH at Wave 1. We found that higher R/S engagement at Wave 1 significantly predicted better SRH at Wave 2, but that better SRH at Wave 1 did not significantly predict higher R/S engagement at Wave 2. However, the strength of their associations on each other over time did not differ significantly.

Post-hoc analyses with sample estimates that weighted (rather than unweighted analyses) for multiple sampling differentials suggested our finding was true for older rather than younger participants. Among older participants, results were consistent for both weighted and unweighted sample analyses. Although this analysis was conducted post-hoc, we intentionally recruited older participants in this study for various reasons. Older people have greater health concerns and tend to be more religious, and that is when self-rated health, chronic illnesses, and attendance at religious services may matter most. Our weighting primarily corrected for age (i.e., our oversampling of older adults), and due to the discrepancy in results we wanted to explore whether this could potentially be due to differences in older and younger people, which is what we found. Thus, future research may explore age-related differences in the dynamics between how R/S engagement and SRH are related to each other.

Our findings add to other investigations of the reciprocal relationships between R/S and health over time that have become of more interest due to the strength of these associations and the difficulty in disentangling which precedes the other. To our knowledge, the closest examination of this question was by Doane and Elliott [29], who investigated a Christian sample at three timepoints. They observed that greater conservative beliefs at Wave 1 predicted worsening self-rated health at Wave 2, and that worsening self-rated health at Wave 2 subsequently predicted greater conservative beliefs at Wave 3. The authors also found that engagement in religious activities was associated with improved self-rated health at all three timepoints; however, higher self-rated health at Wave 1 predicted less engagement in religious activities at Wave 2, and lower self-rated health at Wave 2 predicted more engagement in religious activities at Wave 3. Although Doane and Elliott [29] also used composite measures of beliefs (conservative vs. liberal Christian) and activities (including religious service attendance, participation in other organized activities, and prayer), their study was limited in its use of a Christian-only sample and its assessment of self-rated health via the standard single item. Although our sample was also predominantly Christian, our R/S engagement latent factor was broader, our questions were not Christian-centric, and we assessed for chronic illnesses alongside 2 items assessing self-rated health. Nevertheless, following multiple calls to investigate the relationships between R/S and health using more sophisticated longitudinal methods (e.g., [11,12]), both studies demonstrate that R/S and self-rated health likely impact each other reciprocally, with R/S engagement more consistently associated with significant improvements in self-rated health.

Toward understanding which comes first between R/S engagement and better health, our study may help identify next steps. Findings from our study and that by others suggest that higher R/S engagement more commonly predicts better self-rated health whereas better self-rated health does not always predict higher R/S engagement. However, since neither association was stronger than the other (a question that is rarely asked), we propose that the answer may be need and context dependent. That is, although individuals engaging with R/S are more likely to report better self-rated health, those struggling with their health may be more likely to engage with R/S due to a higher need, and those enjoying better health may be less likely to engage with R/S [29]. Further, whether R/S engagement predicts better or worse health also depends on the specific context [22] and R/S dimensions being assessed. As Stavrova [22] observed, religiosity is associated with better health and lower mortality in countries and regions where it represents the social norm and is desirable. Building upon this, in these contexts, certain central aspects of R/S engagement that have been found to be associated with worsening health may be even more potent, such as conservative or fundamentalist religious beliefs [29], punishing appraisals of God [16], avoidant R/S coping (i.e., spiritual bypass [43]), and spiritual struggles [17]. Likewise, intrinsic (i.e., personal) vs. extrinsic (i.e., social) and other conceptual and theoretical divisions of R/S may be related to wellbeing in unique ways (e.g., [44]). Findings thus far point toward the relationship between R/S engagement and health being both reciprocal and context dependent.

The answer to the religion/spirituality and health causality debate may also be informed by multiple additional research questions. The first has to do with the causal lag (i.e., effect of time between data collection points). For example, the lag in our study was six years, but the timing of the follow-up interviews was determined solely by the availability of funding and not some fully articulated theoretical discussion of how long it really takes for religion/spirituality to translate into better health. One way to proceed is to test models with varying between-round intervals and see where the strongest effects arise – whether one year, three years, or ten years? Further, there are two ways in which religion/spirituality may affect health: (1) lagged - as we studied it; and (2) contemporaneous (as reflected in the correlated disturbance terms at both Time 1 and Time 2). But the best way to assess contemporaneous effects is to simultaneously assess a structural path from religion/spirituality to health and health to religion/spirituality within each time period (i.e., a non-recursive model). However, our two-timepoint data precluded simultaneous tests of lagged and contemporaneous effects in the same model. One last issue involving lagged models is that, as in our study, religion/spirituality is measured with multiple dimensions. But this assumes that the causal lag for each dimension of religion/spirituality is the same (e.g., that the effect of religious service attendance on health is the same as the causal lag for religious coping on health). Our purpose was to begin by casting a wider net via evaluating a more comprehensive set of empirically supported religion/spirituality measures. A next step is to investigate whether the causal lag for each component of religion/spirituality is the same.

Our findings also suggest two additional lines of inquiry beyond the “which comes first” question, starting with the measurement of both variables. It has become increasingly clear from the R/S and health literature that not all aspects of R/S are beneficial. Adequate assessment of R/S variables using both theoretical and empirical methods may provide nuance and reduce bias. For example, spiritual struggles may statistically fit in models alongside other R/S variables, but theoretically should be assessed as a separate factor (e.g., [43]) or for its interactions with other, more adaptive aspects of R/S. There is also growing recognition that heterogeneity within and across religions requires better assessment tools to represent diverse beliefs and practices (e.g., as compiled by [45]) and to capture their respective impacts on health. For example, an individual may report a lack of prayer despite a regular spiritual practice (such as meditation), leading to potentially inaccurate representations of both their experiences and how they impact wellbeing. Whether and how R/S measures based on diverse groups and beliefs can be compared with each other is another matter. A similar issue arises around self-rated health that, although strongly predictive of both R/S and mortality, is still limited in its ability to provide us with specific clinical recommendations. Combining SRH with objective markers of health may help alleviate some of its ambiguity, yet exploring SRH further may help illuminate its true meaning and why it may sometimes outperform other objective measures of health or wellbeing. In sum, heterogeneity in the measurement of both R/S and SRH has consequences for our understanding of their reciprocal impacts over time and among diverse groups, and should be improved upon.

Another line of inquiry beyond “which comes first” surrounds the explanations and potential mediators of the longitudinal relationships between R/S and health, broadly defined. Multiple indices of physical health have been studied for their relationships with R/S, including stress hormones, immune function, cancer, heart disease, hypertension, and chronic pain, among others. These have been reviewed extensively by Koenig and colleagues [1–3], and generally demonstrate protective effects of R/S. Specific indicators of health that are objective and biological, such as C-Reactive Protein (CRP – a key marker of inflammation [46,47]), cortisol [48], and HIV progression [16] have allowed for greater specificity in this line of research. At the same time, R/S is also associated with improved psychosocial functioning such as hope, optimism, meaning in life, will to live, self-control, social support (both giving and receiving), depression, and anxiety [5,6,48–53]. Clearly, there is a breadth of literature on the various biological, physiological, and social benefits of R/S that can be leveraged to better explain mediators and moderators of the relationships between R/S and health over time.

In attempting to resolve the challenge of understanding how religion affects health, Krause [7] argued for dividing the relationships into three categories: 1) the role of R/S in buffering the negative effects of stress on health, 2) the important role of social relationships in improving health independent of the stress process, and 3) negative elements such as spiritual struggles. These three were conceptualized as being linked via a higher-order factor of R/S engagement that reflects the need to find meaning in life through R/S [7]. This also aligns with our improved understandings of mind-body connections in fields that are gaining traction, such as psychoneuroimmunology and psychosomatic medicine, and the roles played by certain key mechanisms. For example, random acts of kindness toward known others are associated with reduced Conserved Transcriptional Response to Adversity (CTRA) gene expression, which buffers against increased pro-inflammatory gene expression and decreased antiviral and antibody production [54,55]. In turn, CTRA gene expression has been linked to cancer and cardiovascular disease [51]. We [21] similarly found that people with HIV who prayed for known others (vs. for themselves or for unknown others) were more likely to survive over 17 years. Notably, in a recent meta-analysis, Shattuck and Muehlenbein [44] suggested that the mechanism whereby R/S improves physiological health is by disrupting the inflammation that follows stressful experiences. Thus, inflammation may be a mechanism that is tied to all three of the categories Krause [7] considered in the relationships between R/S and health, and may help us narrow down how R/S engagement translates to better health while reducing some of the difficulties caused by the multidimensional measurement of both constructs. In sum, depending on the cultural context, beneficial/harmful aspects of R/S engagement may promote/harm individual, emotional, and social wellbeing, subsequently influencing several biological systems (e.g., sympathetic, endocrine, and immune systems) and health in a bidirectional or cyclical manner.

### Clinical implications

Our study adds to the literature supporting the beneficial aspects of R/S engagement for health [1,2]. It supports the notion that R/S engagement may be recommended as an adjunctive or complementary intervention alongside primary or necessary treatments as it is consistently tied to improvements in individual, social, and other measurable aspects of wellbeing and health [1,2,44]. This advice is also supported by evidence. For example, Gonçalves and colleagues [56] conducted a systematic review of randomized controlled clinical trials investigating the benefits of complementary interventions. The authors found that complementary R/S interventions (compared to other complementary interventions) were related to improvements in physical health, quality of life, pain, weight management, and health-promoting behaviors (improvements were not noted in all domains assessed), with very small to small effect sizes. As such, individuals seeking to improve their health and wellbeing may turn to a regular and supportive R/S practice with an eye on increasing their individual, social, and spiritual resources.

### Limitations and future directions

Establishing causality is not possible in this or any other longitudinal study. Future attempts at exploring “which comes first” between R/S and health in one’s daily life should utilize methods better suited to answering it, such as experiments or at least three-wave data and multi-level modeling or autoregressive techniques (e.g., [29]), to determine how this dynamic functions across groups, time, R/S dimensions, and aspects of wellbeing. Another limitation is that our sample was based on Nationwide sampling methods but involved self-selection (possibly leading to information bias due to self-report), limited representation of diverse religious/spiritual and racial/ethnic backgrounds, and unknown generalizability to other countries that should be targeted more directly in future work. Similarly, our high attrition rate in Wave 2 (67.94%) may have biased our results as seen through the sample attrition analyses (e.g., those who were retained at Wave 2 had initially reported lower religious coping and number of chronic illnesses, higher education, income, and attendance, and better self-rated health). Our high attrition was likely due to limited resources relative to Wave 1 and the timing of Wave 2 (during a contested election year and COVID-19), although it was still in the range of other longitudinal studies (i.e., between 30–70%; [57]).

Although valid weighted estimates were calculated based on the oversampling of older adults and multiple sampling differentials, the variances we reported may still be underestimated due to the clustering of respondents, both within selected census tracts and allowing two respondents per household. Thus, future large-scale studies exploring such research questions may also attempt to gather and assess how weighting according to household and census tract clustering may impact findings on the relationships between R/S engagement and SRH over time.

Although we did assess for measurement invariance in our constructs of R/S engagement and self-rated health over time, we did not assess for measurement invariance across genders or racial/ethnic groups. Future work could also assess measurement invariance across groups when identifying beneficial and harmful elements of R/S for physical and mental health, which was not among our proposed aims in this study. Despite our focus on using the most widely supported and standardized items from the R/S and health literature, our measures of R/S engagement were also inherently limited (e.g., assessing for prayer but not meditation). Likewise, although biological markers of health and disease were collected at Wave 1, we did not collect them at Wave 2. Further, there is debate within the R/S and health research field around the many dimensions of R/S, with some arguing that religion and spirituality are not distinct [1,2], and others suggesting that dividing R/S into its intrinsic (i.e., subjective or personal) and extrinsic (i.e., social or affiliative) elements is critical [44]. Improving measurement – and measurement bias – in R/S and health research remains an important future direction, and requires healthy debate and consensus around the divisions between religion, spirituality, and the many dimensions within.

## Conclusion

Our study extends the literature supporting the beneficial aspects of R/S engagement for self-rated health [22]. It adds to the literature by exploring their reciprocal relationships using composite measures of both R/S engagement and self-rated health in a two-point longitudinal study conducted using a nationwide sample. Further, we examined these relationships within a structural equation modeling framework and established strict partial measurement invariance, with multiple controls for demographic variables (i.e., age, sex, education, income, race/ethnicity) as well as R/S engagement and self-rated health at Wave 1. Our findings demonstrate that R/S engagement significantly predicted better self-rated health, but that self-rated health did not significantly predict R/S engagement. However, the strengths of these relationships over time were not significantly different, and our findings were driven mostly by the older participants. Future research in this area should explore issues around their measurement, diversity of the samples and contexts being assessed, length and impact of multiple timepoint assessments, and particularly salient mediators such as inflammation, to further unravel the question of which comes first and through which mechanisms.

The vast majority of studies on many aspects of religion/spirituality are cross-sectional. This reflects a number of factors, including the availability of funding for gathering follow-up data. But it also may arise from the fact that many investigators find it easier to think in static terms, rather than dynamic terms (which, in all likelihood, come closer to social reality). Although the reasons for this situation are not entirely clear, we suspect that it may have something to do with training – many scholars simply may not know how to conduct a thorough set of longitudinal analyses. We have not provided a fully developed tutorial on this matter, even though such discussions exist (see for example [58]). Instead, information on how to conduct sound longitudinal analyses is unevenly spread across many disciplines (and subdisciplines). Even so, we hope the issues we have raised and the procedures we have utilized encourage other investigators to delve more deeply into some of the more challenging aspects of analyzing data that have been gathered at more than one point in time.

## Supporting information

S1 Fig Final measurement model without covariances between indicators. Standardized factor loadings and measurement error terms are reported for indicators and latent variables. Errors for all indicators at Wave 1 were correlated with their respective duplicate indicator at Wave 2, with the exception of Rate Your Health (SRH Item 1). * *p* <.05. ** *p* <.001.
(TIF)

S1 Table Standardized measurement error parameter estimates for item study measures (N = 3010). Factor loadings and measurement error terms are from the completely standardized solution, and are significant at the *p* <.001 level. The measurement error term for Number of Chronic Illnesses across time points was allowed to vary.
(PDF)

S1 File Which comes first: Religious/Spiritual engagement or self-rated health? Code and output file.
(HTML)

S2 File List of chronic illnesses. (DOCX)

S3 File Sampling and weighting report. Table 6 has been redacted to prevent disclosure risk to participants.
(DOC)
