# Supplementary figures and images for: “Which comes first”: Religious/spiritual engagement or health? Initial observations from longitudinal analyses
Source: PLoS One. 2025 May 7;20(5):e0320410. doi: 10.1371/journal.pone.0320410 (PMC12057932; doi:10.1371/journal.pone.0320410)

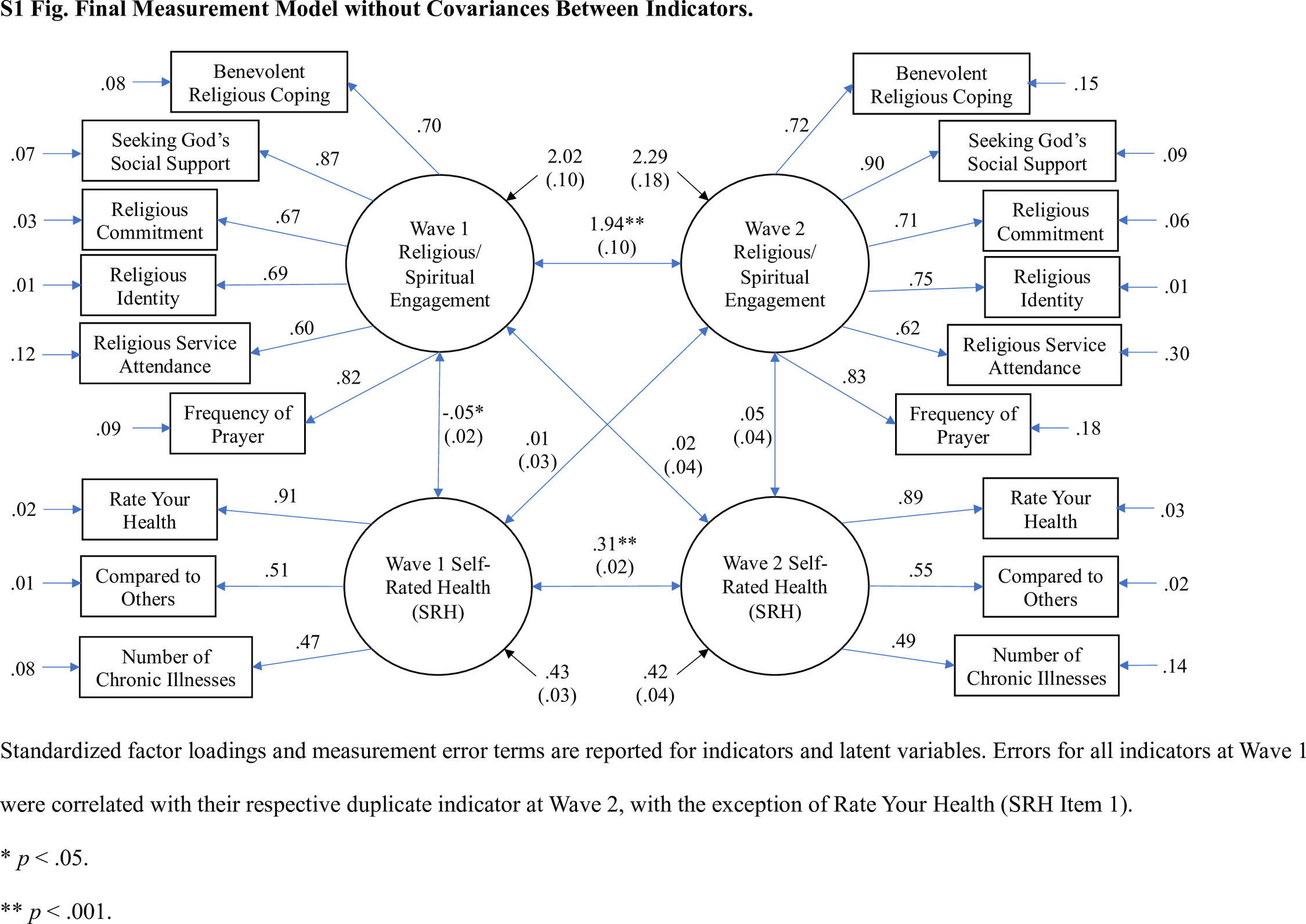

Supplement: S1 Fig — Standardized factor loadings and measurement error terms are reported for indicators and latent variables. Errors for all indicators at Wave 1 were correlated with their respective duplicate indicator at Wave 2, with the exception of Rate Your Health (SRH Item 1). * p <.05. ** p <.001. (TIF) [file pone.0320410.s001.tif]
