# Supplementary material for: “Which comes first”: Religious/spiritual engagement or health? Initial observations from longitudinal analyses
Source: PLoS One. 2025 May 7;20(5):e0320410. doi: 10.1371/journal.pone.0320410 (PMC12057932; doi:10.1371/journal.pone.0320410)
Supplement: S1 Table — Factor loadings and measurement error terms are from the completely standardized solution, and are significant at the p <.001 level. The measurement error term for Number of Chronic Illnesses across time points was allowed to vary. (PDF) [file pone.0320410.s002.pdf]

S1 Table. Standardized Measurement Error Parameter Estimates for Item Study Measures (N = 3010).

Construct	Factor Loading	Measurement Error
Wave 1 Self-rated Health		
Item 1: Rate your overall health at present	.744	.014
Item 2: Compared to most people your age	.577	.008
Number of Chronic Illnesses	.434	.059
Wave 1 R/S Engagement		
Religious Identity	.632	.006
Frequency of Prayer	.729	.083
Religious Service Attendance	.536	.119
Religious Commitment	.609	.024
Benevolent R/S Coping	.646	.065
Seeking God's Social Support	.795	.057
Wave 2 Self-rated Health		
Item 1: Rate your overall health at present	.801	.014
Item 2: Compared to most people your age	.662	.008
Number of Chronic Illnesses	.523	.107
Wave 2 R/S Engagement		
Religious Identity	.735	.006
Frequency of Prayer	.816	.083
Religious Service Attendance	.634	.119
Religious Commitment	.717	.024
Benevolent R/S Coping	.757	.065
Seeking God's Social Support	.871	.057

Factor loadings and measurement error terms are from the completely standardized solution, and are significant at the $p < .001$ level. The measurement error term for Number of Chronic Illnesses across time points was allowed to vary.
