# Supplementary material for: “Which comes first”: Religious/spiritual engagement or health? Initial observations from longitudinal analyses
Source: PLoS One. 2025 May 7;20(5):e0320410. doi: 10.1371/journal.pone.0320410 (PMC12057932; doi:10.1371/journal.pone.0320410)
Supplement: S1 File — Code and output file. (HTML) [file pone.0320410.s003.html]

S1-File.-Which-Comes-First-.knit


Code 

- Show All Code
- Hide All Code
- Download Rmd

# S1 File. Which Comes First: Religious/Spiritual Engagement or Self-Rated Health?

#### Ahmad et al., 2024

#### 2025-02-03

# Table of Contents

- Go to Preparatory Work
  - Go to Packages
  - Go to Preferences
  - Go to Load the Data
- Go to Preliminary Analyses
  - Go to Demographics
  - Go to Cronbach’s Alphas
  - Go to Skewness and Kurtosis
  - Go to Sample Attrition Analysis
  - Go to Correlation Matrix
- Go to Primary Analyses
  - Go to Measurement Models
    - Go to Religious/Spiritual (R/S)
      Engagement
    - Go to Self-Rated Health (SRH)
    - Go to R/S Engagement at both Timepoints
    - Go to SRH at both Timepoints
    - Go to Full Measurement Model
  - Go to Longitudinal Measurement Invariance
    - Go to Self-Rated Health Longitudinal
      Invariance
    - Go to R/S Engagement Longitudinal
      Invariance
    - Go to Full Model with Longitudinal
      Invariance
  - Go to Structural Equation Models
    - Go to Strength of Associations
- Go to Appendix
  - Go to Self-Rated Health Item 3
  - Go to Weighted Analyses
    - Go to Weighted Measurement Models
    - Go to Weighted Religious/Spiritual (R/S)
      Engagement
    - Go to Weighted Self-Rated Health (SRH)
    - Go to Weighted R/S Engagement at both
      Timepoints
    - Go to Weighted SRH at both Timepoints
    - Go to Weighted Full Measurement Model
    - Go to Weighted Longitudinal Measurement
      Invariance
    - Go to Weighted Self-Rated Health Longitudinal
      Invariance
    - Go to Weighted R/S Engagement Longitudinal
      Invariance
    - Go to Weighted Full Model with Longitudinal
      Invariance
    - Go to Weighted Structural Equation Models
    - Go to Weighted Strength of
      Associations
    - Go to Under and Over Median Age of 52
      Years

Welcome.

This is the code and output file associated with the PLOS ONE
manuscript titled: *Which comes first: Religious/spiritual engagement
or health? Initial observations from longitudinal analyses.* This
file was prepared in the **rUM** R
Markdown template, developed by Dr. Raymond Balise and colleagues at the
University of Miami. Some of the initial preparatory work done on the
template is not displayed, but for those interested, it can be seen by
downloading the R Markdown file. To download the file, go to the
**Code** drop down menu on the top right corner of this
page, and select **Download Rmd**. You will also need to
access the data file, which is in csv format and can be accessed from
the same location as this file within the Open Science Framework’s
online repository.

The order of the code and output follow that of the manuscript. To
make it easier to navigate the extensive analyses in this study, the
code and output can be collapsed/hidden and displayed. Click on
**Show** on the right side of a section to display or hide
its code and output. Alternatively, you can once again click on
**Code** above and then **Show All Code** if
you want to see everything. Comments have been used to help guide
readers (e.g., on the specific analyses taking place, or the values of
categories when looking at categorical variables). Hashtags
(**#**) indicate whether the line within a code chunk is a
comment or part of the analytic code and resulting output. For
additional details around our decision-making, such as on our selection
of standards for fit indices, please refer to the manuscript.

If you have questions or run into problems, please don’t hesitate to
email me at **salmanshaheenahmad@gmail.com**. Thank
you, and I hope you enjoy our work!

- Salman S. Ahmad

# Preparatory Work

Set up the **rUM** Markdown template, and select your
packages and preferences. Some specifications are hidden here but can be
accessed by downloading the full document.

```
knitr::opts_chunk$set(
  # These options can be set to FALSE (capitalization matters).
  echo = TRUE, # show code
  message = TRUE, # show messages
  warning = TRUE, # show warnings
  error = TRUE, # show errors
  comment = "" # don't show ## with printed output
)

# Chi-square difference values were not displaying to two decimal places, so the
# following code was used
# Register an inline hook:
knitr::knit_hooks$set(inline = function(x) {
  x <- sprintf("%1.2f", x)
  paste(x, collapse = ", ")
})
```

## Packages

Load the packages needed to analyze the data.

```
# Remove the hashtags if the packages have not already been installed

# install.packages("lavaan")
library(lavaan)
# install.packages("foreign")
library(foreign)
# install.packages("dplyr")
library(dplyr)
# install.packages("ltm")
library(ltm)
# install.packages("skimr")
library(skimr)
# install.packages("moments")
library(moments)
# install.packages("Hmisc")
library(Hmisc)
# install.packages("rstatix")
library(rstatix)
# install.packages("forcats")
library(forcats)
```

## Preferences

Set some preferences for how results are displayed.

```
# Get nice symbols when printing effect sizes

options(es.use_symbols = TRUE)
```

## Load the Data

Load in the csv file titled **Which Comes First
Data.csv**.

```
# Add in data

which_comes_first_data <- read_csv("Which Comes First Data.csv",
  show_col_types = FALSE
)
```

- Back to the Table of Contents

# Preliminary Analyses

## Demographic Data

Gather demographic and descriptive data on the sample. See comments
for values of categorical variables (above the code that analyzes the
respective variable).

```
#   Sex
# 0 - Female
# 1 - Male

table(which_comes_first_data$is_male)

   0    1 
1723 1287 

# Race/Ethnicity - White
# 0 - Non-White
# 1 - White

table(which_comes_first_data$is_white)

   0    1 
 992 1990 

# Race/Ethnicity - Black, non-Hispanic
# 0 - Non-Black
# 1 - Black

table(which_comes_first_data$is_black)

   0    1 
2567  411 

# Race/Ethnicity - Hispanic, including Hispanic Black
# 0 - Non-Hispanic
# 1 - Hispanic

table(which_comes_first_data$is_hispanic)

   0    1 
2529  468 

#   Religious Preference
# 1 - Catholic or Roman Catholic
# 2 - Protestant
# 3 - Jewish
# 4 - Muslim
# 5 - Buddhist
# 6 - Hindu
# 7 - Other
# 8 - No Religious Preference
# 9 - Agnostic
# 10 - Atheist
# 11 - Don't Know
# 12 - Refused

table(which_comes_first_data$religious_preference)

  1   2   3   4   5   6   7   8   9  10  11  12 
603 970  48  19  39   7 757 368 128  62   3   6 

# Religious/Spiritual Identity Wave 1
# 0 - Neither Religious nor Spiritual
# 1 - Either Religious or Spiritual
# 2 - Both Religious and Spiritual

table(which_comes_first_data$rs_identity_w1)

   0    1    2 
 258 1089 1624 

# Religious/Spiritual Identity Wave 2
# 0 - Neither Religious nor Spiritual
# 1 - Either Religious or Spiritual
# 2 - Both Religious and Spiritual

table(which_comes_first_data$rs_identity_w2)

  0   1   2 
 69 214 324 

# Prayer Frequency Wave 1
# 0 - Did not pray
# 1 - Less than once a month
# 2 - Once a month
# 3 - A few times a month
# 4 - Once a week
# 5 - A few times a week
# 6 - Daily
# 7 - More than once daily

table(which_comes_first_data$prayer_w1)

   0    1    2    3    4    5    6    7 
 357  141   66  154   80  377  697 1118 

# Prayer Frequency Wave 2
# 0 - Did not pray
# 1 - Less than once a month
# 2 - Once a month
# 3 - A few times a month
# 4 - Once a week
# 5 - A few times a week
# 6 - Daily
# 7 - More than once daily

table(which_comes_first_data$prayer_w2)

  0   1   2   3   4   5   6   7 
 98  30  20  33  18  75 132 200 

# Religious Service Attendance Frequency Wave 1
# 0 - Never
# 1 - Less than once a year
# 2 - About once or twice a year
# 3 - Several times a year
# 4 - About once a month
# 5 - 2 to 3 times a month
# 6 - Nearly every week
# 7 - Every week
# 8 - Several times a week

table(which_comes_first_data$attendance_w1)

  0   1   2   3   4   5   6   7   8 
550 286 379 320 142 251 234 574 253 

# Religious Service Attendance Frequency Wave 2
# 0 - Never
# 1 - Less than once a year
# 2 - About once or twice a year
# 3 - Several times a year
# 4 - About once a month
# 5 - 2 to 3 times a month
# 6 - Nearly every week
# 7 - Every week
# 8 - Several times a week

table(which_comes_first_data$attendance_w2)

  0   1   2   3   4   5   6   7   8 
174  46  55  43  21  32  46 133  56 

#   Commitment Wave 1
# 0 - Strongly Disagree
# 1 - Disagree
# 2 - Neutral
# 3 - Agree
# 4 - Strongly Agree

table(which_comes_first_data$commitment_w1)

   0    1    2    3    4 
 138  371   72 1317 1081 

#   Commitment Wave 2
# 0 - Strongly Disagree
# 1 - Disagree
# 2 - Neutral
# 3 - Agree
# 4 - Strongly Agree

table(which_comes_first_data$commitment_w2)

  0   1   2   3   4 
 47  37  95 191 237 

# Self-Rated Health Item 1 (Rate your overall health) Wave 1
# 0 - Poor
# 1 - Fair
# 2 - Good
# 3 - Excellent

table(which_comes_first_data$srh_item1_w1)

   0    1    2    3 
  99  584 1723  599 

# Self-Rated Health Item 1 (Rate your overall health) Wave 2
# 0 - Poor
# 1 - Fair
# 2 - Good
# 3 - Excellent

table(which_comes_first_data$srh_item1_w2)

  0   1   2   3 
 15 110 345 145 

# Self-Rated Health Item 2 (Compared to most people your age) Wave 1
# 0 - Worse
# 1 - About the same
# 2 - Better

table(which_comes_first_data$srh_item2_w1)

   0    1    2 
 269 1371 1346 

# Self-Rated Health Item 2 (Compared to most people your age) Wave 2
# 0 - Worse
# 1 - About the same
# 2 - Better

table(which_comes_first_data$srh_item2_w2)

  0   1   2 
 55 251 308 

# Benevolent Religious Coping Mean Wave 1

mean(which_comes_first_data$rcope_ben_total_w1, na.rm = TRUE)
[1] 2.924068

# Benevolent Religious Coping SD Wave 1

sd(which_comes_first_data$rcope_ben_total_w1, na.rm = TRUE)
[1] 1.964987

# Benevolent Religious Coping Mean Wave 2

mean(which_comes_first_data$rcope_ben_total_w2, na.rm = TRUE)
[1] 2.771777

# Benevolent Religious Coping SD Wave 2

sd(which_comes_first_data$rcope_ben_total_w2, na.rm = TRUE)
[1] 2.119099

# Seeking God's Social Support Mean Wave 1

mean(which_comes_first_data$rcope_sup_total_w1, na.rm = TRUE)
[1] 4.136467

# Seeking God's Social Support SD Wave 1

sd(which_comes_first_data$rcope_sup_total_w1, na.rm = TRUE)
[1] 2.03016

# Seeking God's Social Support Mean Wave 2

mean(which_comes_first_data$rcope_sup_total_w2, na.rm = TRUE)
[1] 3.883275

# Seeking God's Social Support SD Wave 2

sd(which_comes_first_data$rcope_sup_total_w2, na.rm = TRUE)
[1] 2.16798

# Number of chronic illnesses Mean Wave 1

mean(which_comes_first_data$chronic_w1, na.rm = TRUE)
[1] 1.596013

# Number of chronic illnesses SD Wave 1

sd(which_comes_first_data$chronic_w1, na.rm = TRUE)
[1] 1.749591

# Number of chronic illnesses Mean Wave 2

mean(which_comes_first_data$chronic_w2, na.rm = TRUE)
[1] 1.757774

# Number of chronic illnesses SD Wave 2

sd(which_comes_first_data$chronic_w2, na.rm = TRUE)
[1] 1.615575

# Age Mean Wave 1

meanAge <- mean(which_comes_first_data$age_w1, na.rm = TRUE)
meanAge
[1] 51.63507

# Age SD Wave 1

SDAge <- sd(which_comes_first_data$age_w1, na.rm = TRUE)
SDAge
[1] 19.28207

# Age Median Wave 1

median(which_comes_first_data$age_w1, na.rm = TRUE)
[1] 52

# Age Range Wave 1

range(which_comes_first_data$age_w1, na.rm = TRUE)
[1] 17 96

# Age Mean Wave 2

meanAgewave2 <- mean(which_comes_first_data$age_w2, na.rm = TRUE)
meanAgewave2
[1] 57.967

# Age SD Wave 2

SDAgewave2 <- sd(which_comes_first_data$age_w2, na.rm = TRUE)
SDAgewave2
[1] 17.47902

# Education Mean Wave 1

meanEduwave1 <- mean(which_comes_first_data$education, na.rm = TRUE)
meanEduwave1
[1] 13.46793

# Education SD Wave 1

SDEduwave2 <- sd(which_comes_first_data$education, na.rm = TRUE)
SDEduwave2
[1] 3.158341

# Income Median Wave 1

median(which_comes_first_data$income, na.rm = TRUE)
[1] 7


# Separating dataset by waves to identify Wave 1 subset participants in table 1

Missing <- is.na(which_comes_first_data$srh_item1_w2)
sum(Missing)
[1] 2395

wave2 <- subset(which_comes_first_data,
  subset = !Missing
)

# Sex Wave 1 Subset
# 0 - Female
# 1 - Male

table(wave2$is_male)

  0   1 
349 266 

# Race/Ethnicity - White Wave 1 Subset
# 0 - Non-White
# 1 - White

table(wave2$is_white)

  0   1 
145 462 

# Race/Ethnicity - Black, non-Hispanic Wave 1 Subset
# 0 - Non-Black
# 1 - Black

table(wave2$is_black)

  0   1 
549  57 

# Race/Ethnicity - Hispanic, including Hispanic Black Wave 1 Subset
# 0 - Non-Hispanic
# 1 - Hispanic

table(wave2$is_hispanic)

  0   1 
546  68 

# Religious Preference Wave 1 Subset
# 1 - Catholic or Roman Catholic
# 2 - Protestant
# 3 - Jewish
# 4 - Muslim
# 5 - Buddhist
# 6 - Hindu
# 7 - Other
# 8 - No Religious Preference
# 9 - Agnostic
# 10- Atheist

table(wave2$religious_preference)

  1   2   3   4   5   6   7   8   9  10 
131 207  15   3  12   1 125  67  35  19 

# Religious/Spiritual Identity Wave 1 Subset
# 0 - Neither Religious nor Spiritual
# 1 - Either Religious or Spiritual
# 2 - Both Religious and Spiritual

table(wave2$rs_identity_w1)

  0   1   2 
 54 206 347 

# Prayer Frequency Wave 1 Subset
# 0 - Did not pray
# 1 - Less than once a month
# 2 - Once a month
# 3 - A few times a month
# 4 - Once a week
# 5 - A few times a week
# 6 - Daily
# 7 - More than once daily

table(wave2$prayer_w1)

  0   1   2   3   4   5   6   7 
 94  32   9  27  17  63 125 241 

# Religious Service Attendance Frequency Wave 1 Subset
# 0 - Never
# 1 - Less than once a year
# 2 - About once or twice a year
# 3 - Several times a year
# 4 - About once a month
# 5 - 2 to 3 times a month
# 6 - Nearly every week
# 7 - Every week
# 8 - Several times a week

table(wave2$attendance_w1)

  0   1   2   3   4   5   6   7   8 
 95  64  75  66  18  51  54 122  65 

# Commitment Wave 1 Subset
# 0 - Strongly Disagree
# 1 - Disagree
# 2 - Neutral
# 3 - Agree
# 4 - Strongly Agree

table(wave2$commitment_w1)

  0   1   2   3   4 
 42  68  11 252 235 

# Self-Rated Health Item 1 (Rate your overall health) Wave 1 Subset
# 3 - Poor
# 2 - Fair
# 1 - Good
# 0 - Excellent

table(wave2$srh_item1_w1)

  0   1   2   3 
 11  85 358 160 

# Self-Rated Health Item 2 (Compared to most people your age) Wave 1 Subset
# 0 - Worse
# 1 - About the same
# 2 - Better

table(wave2$srh_item2_w1)

  0   1   2 
 49 247 312 

# Benevolent Religious Coping Mean Wave 1 Subset

mean(wave2$rcope_ben_total_w1, na.rm = TRUE)
[1] 2.740406

# Benevolent Religious Coping SD Wave 1 Subset

sd(wave2$rcope_ben_total_w1, na.rm = TRUE)
[1] 2.082651

# Seeking God's Social Support Mean Wave 1 Subset

mean(wave2$rcope_sup_total_w1, na.rm = TRUE)
[1] 3.952809

# Seeking God's Social Support SD Wave 1 Subset

sd(wave2$rcope_sup_total_w1, na.rm = TRUE)
[1] 2.18513

# Number of chronic illnesses Mean Wave 1 Subset

mean(wave2$chronic_w1, na.rm = TRUE)
[1] 1.426016

# Number of chronic illnesses SD Wave 1 Subset

sd(wave2$chronic_w1, na.rm = TRUE)
[1] 1.59518

# Age Mean Wave 1 Subset

meanAge <- mean(wave2$age_w1, na.rm = TRUE)
meanAge
[1] 51.83168

# Age SD Wave 1 Subset

SDAge <- sd(wave2$age_w1, na.rm = TRUE)
SDAge
[1] 17.50385

# Age Range Wave 1 Subset

range(wave2$age_w1, na.rm = TRUE)
[1] 18 88

# Education Mean Wave 1 Subset

meanEduwave1 <- mean(wave2$education, na.rm = TRUE)
meanEduwave1
[1] 14.77724

# Education SD Wave 1 Subset

SDEduwave2 <- sd(wave2$education, na.rm = TRUE)
SDEduwave2
[1] 3.073603

# Income Median Wave 1 Subset

median(wave2$income, na.rm = TRUE)
[1] 8
```

- Back to the Table of Contents

## Cronbach’s Alphas

Get Cronbach’s alphas for the two RCOPE subscales.

```
# Cronbachs Alpha for Benevolent Coping Wave 1

copeben_alpha <- which_comes_first_data %>%
  dplyr::select(rcope_ben_item1_w1, rcope_ben_item2_w1) %>%
  na.omit()
cronbach.alpha(copeben_alpha)

Cronbach's alpha for the 'copeben_alpha' data-set

Items: 2
Sample units: 2173
alpha: 0.738

# Cronbachs Alpha for Benevolent Coping Wave 2

copeben_w2_alpha <- which_comes_first_data %>%
  dplyr::select(rcope_ben_item1_w2, rcope_ben_item2_w2) %>%
  na.omit()
cronbach.alpha(copeben_w2_alpha)

Cronbach's alpha for the 'copeben_w2_alpha' data-set

Items: 2
Sample units: 574
alpha: 0.775

# Cronbachs Alpha for Seeking Social Support Wave 1

copesup_alpha <- which_comes_first_data %>%
  dplyr::select(rcope_sup_item1_w1, rcope_sup_item2_w1) %>%
  na.omit()
cronbach.alpha(copesup_alpha)

Cronbach's alpha for the 'copesup_alpha' data-set

Items: 2
Sample units: 2191
alpha: 0.925

# Cronbachs Alpha for Seeking Social Support Wave 2

copesup_w2_alpha <- which_comes_first_data %>%
  dplyr::select(rcope_sup_item1_w2, rcope_sup_item2_w2) %>%
  na.omit()
cronbach.alpha(copesup_w2_alpha)

Cronbach's alpha for the 'copesup_w2_alpha' data-set

Items: 2
Sample units: 574
alpha: 0.927
```

- Back to the Table of Contents

## Skewness and Kurtosis

Assess normality of all primary study variables using skewness and
kurtosis.

```
# Checking skewness and kurtosis

# Benevolent Religious Coping Wave 1

print(skewness(which_comes_first_data$rcope_ben_total_w1, na.rm = TRUE))
[1] 2.143801e-05
print(kurtosis(which_comes_first_data$rcope_ben_total_w1, na.rm = TRUE))
[1] 1.853289

# Benevolent Religious Coping Wave 2

print(skewness(which_comes_first_data$rcope_ben_total_w2, na.rm = TRUE))
[1] 0.1221656
print(kurtosis(which_comes_first_data$rcope_ben_total_w2, na.rm = TRUE))
[1] 1.696192

# Seeking God's Social Support Wave 1

print(skewness(which_comes_first_data$rcope_sup_total_w1, na.rm = TRUE))
[1] -0.847573
print(kurtosis(which_comes_first_data$rcope_sup_total_w1, na.rm = TRUE))
[1] 2.487078

# Seeking God's Social Support Wave 2

print(skewness(which_comes_first_data$rcope_sup_total_w2, na.rm = TRUE))
[1] -0.6899609
print(kurtosis(which_comes_first_data$rcope_sup_total_w2, na.rm = TRUE))
[1] 2.103585

# Attendance Frequency Wave 1

print(skewness(which_comes_first_data$attendance_w1, na.rm = TRUE))
[1] 0.05560339
print(kurtosis(which_comes_first_data$attendance_w1, na.rm = TRUE))
[1] 1.529941

# Attendance Frequency Wave 2

print(skewness(which_comes_first_data$attendance_w2, na.rm = TRUE))
[1] 0.08603793
print(kurtosis(which_comes_first_data$attendance_w2, na.rm = TRUE))
[1] 1.356351

# Commitment Wave 1

print(skewness(which_comes_first_data$commitment_w1, na.rm = TRUE))
[1] -1.158035
print(kurtosis(which_comes_first_data$commitment_w1, na.rm = TRUE))
[1] 3.418402

# Commitment Wave 2

print(skewness(which_comes_first_data$commitment_w2, na.rm = TRUE))
[1] -1.012862
print(kurtosis(which_comes_first_data$commitment_w2, na.rm = TRUE))
[1] 3.130865

# Prayer Frequency Wave 1

print(skewness(which_comes_first_data$prayer_w1, na.rm = TRUE))
[1] -1.098501
print(kurtosis(which_comes_first_data$prayer_w1, na.rm = TRUE))
[1] 2.744016

# Prayer Frequency Wave 2

print(skewness(which_comes_first_data$prayer_w2, na.rm = TRUE))
[1] -0.8137884
print(kurtosis(which_comes_first_data$prayer_w2, na.rm = TRUE))
[1] 2.092256

# Religious/Spiritual Identity Wave 1

print(skewness(which_comes_first_data$rs_identity_w1, na.rm = TRUE))
[1] -0.8008114
print(kurtosis(which_comes_first_data$rs_identity_w1, na.rm = TRUE))
[1] 2.566976

# Religious/Spiritual Identity Wave 2

print(skewness(which_comes_first_data$rs_identity_w2, na.rm = TRUE))
[1] -0.7660572
print(kurtosis(which_comes_first_data$rs_identity_w2, na.rm = TRUE))
[1] 2.40595

# Self-rated Health Item 1 Wave 1

print(skewness(which_comes_first_data$srh_item1_w1, na.rm = TRUE))
[1] -0.4325675
print(kurtosis(which_comes_first_data$srh_item1_w1, na.rm = TRUE))
[1] 3.193952

# Self-rated Health Item 1 Wave 2

print(skewness(which_comes_first_data$srh_item1_w2, na.rm = TRUE))
[1] -0.4111994
print(kurtosis(which_comes_first_data$srh_item1_w2, na.rm = TRUE))
[1] 3.086731

# Self-rated Health Item 2 Wave 1

print(skewness(which_comes_first_data$srh_item2_w1, na.rm = TRUE))
[1] -0.496488
print(kurtosis(which_comes_first_data$srh_item2_w1, na.rm = TRUE))
[1] 2.322582

# Self-rated Health Item 2 Wave 2

print(skewness(which_comes_first_data$srh_item2_w2, na.rm = TRUE))
[1] -0.6537529
print(kurtosis(which_comes_first_data$srh_item2_w2, na.rm = TRUE))
[1] 2.409145

# Self-rated Health Item 3 Wave 1

print(skewness(which_comes_first_data$srh_item3_w1, na.rm = TRUE))
[1] -0.09470328
print(kurtosis(which_comes_first_data$srh_item3_w1, na.rm = TRUE))
[1] 2.454848

# Self-rated Health Item 3 Wave 2

print(skewness(which_comes_first_data$srh_item3_w2, na.rm = TRUE))
[1] -0.02898045
print(kurtosis(which_comes_first_data$srh_item3_w2, na.rm = TRUE))
[1] 2.460942

# Number of chronic illnesses Wave 1

print(skewness(which_comes_first_data$chronic_w1, na.rm = TRUE))
[1] 1.249321
print(kurtosis(which_comes_first_data$chronic_w1, na.rm = TRUE))
[1] 4.418839

# Number of chronic illnesses Wave 2

print(skewness(which_comes_first_data$chronic_w2, na.rm = TRUE))
[1] 0.9632434
print(kurtosis(which_comes_first_data$chronic_w2, na.rm = TRUE))
[1] 3.938465
```

- Back to the Table of Contents

## Correlation Matrix

Create a separate dataset that includes all the variables wanted in
the correlation matrix, before developing the matrix.

```
# Create separate dataset for correlation matrix

correlation.matrix <- which_comes_first_data %>%
  dplyr::select(
    rs_identity_w1, prayer_w1, attendance_w1, commitment_w1, rcope_ben_total_w1,
    rcope_sup_total_w1,srh_item1_w1, srh_item2_w1, srh_item3_w1, chronic_w1,
    rs_identity_w2, prayer_w2, attendance_w2, commitment_w2, rcope_ben_total_w2,
    rcope_sup_total_w2, srh_item1_w2, srh_item2_w2, srh_item3_w2, chronic_w2
  )

# Create Correlation Matrix

cor_table <- rcorr(as.matrix(correlation.matrix))
cor_table
                   rs_identity_w1 prayer_w1 attendance_w1 commitment_w1
rs_identity_w1               1.00      0.60          0.49          0.49
prayer_w1                    0.60      1.00          0.50          0.56
attendance_w1                0.49      0.50          1.00          0.41
commitment_w1                0.49      0.56          0.41          1.00
rcope_ben_total_w1           0.36      0.48          0.36          0.38
rcope_sup_total_w1           0.50      0.68          0.45          0.54
srh_item1_w1                -0.01     -0.05          0.05          0.02
srh_item2_w1                 0.06      0.01          0.08          0.08
srh_item3_w1                 0.00      0.01          0.03          0.04
chronic_w1                   0.12      0.14          0.06          0.09
rs_identity_w2               0.69      0.62          0.53          0.48
prayer_w2                    0.63      0.80          0.56          0.57
attendance_w2                0.50      0.52          0.75          0.42
commitment_w2                0.56      0.60          0.51          0.57
rcope_ben_total_w2           0.34      0.51          0.45          0.39
rcope_sup_total_w2           0.56      0.69          0.53          0.56
srh_item1_w2                -0.01     -0.02          0.03          0.00
srh_item2_w2                 0.06      0.09          0.10          0.08
srh_item3_w2                -0.04     -0.06          0.00         -0.09
chronic_w2                   0.12      0.11          0.05          0.11
                   rcope_ben_total_w1 rcope_sup_total_w1 srh_item1_w1
rs_identity_w1                   0.36               0.50        -0.01
prayer_w1                        0.48               0.68        -0.05
attendance_w1                    0.36               0.45         0.05
commitment_w1                    0.38               0.54         0.02
rcope_ben_total_w1               1.00               0.68        -0.05
rcope_sup_total_w1               0.68               1.00        -0.06
srh_item1_w1                    -0.05              -0.06         1.00
srh_item2_w1                    -0.04              -0.03         0.46
srh_item3_w1                     0.04               0.00         0.24
chronic_w1                       0.04               0.13        -0.44
rs_identity_w2                   0.40               0.53         0.01
prayer_w2                        0.48               0.68         0.01
attendance_w2                    0.36               0.47         0.04
commitment_w2                    0.42               0.55         0.07
rcope_ben_total_w2               0.52               0.53        -0.01
rcope_sup_total_w2               0.55               0.72        -0.03
srh_item1_w2                     0.03               0.00         0.55
srh_item2_w2                     0.04               0.10         0.33
srh_item3_w2                     0.04               0.00         0.03
chronic_w2                      -0.07               0.08        -0.31
                   srh_item2_w1 srh_item3_w1 chronic_w1 rs_identity_w2
rs_identity_w1             0.06         0.00       0.12           0.69
prayer_w1                  0.01         0.01       0.14           0.62
attendance_w1              0.08         0.03       0.06           0.53
commitment_w1              0.08         0.04       0.09           0.48
rcope_ben_total_w1        -0.04         0.04       0.04           0.40
rcope_sup_total_w1        -0.03         0.00       0.13           0.53
srh_item1_w1               0.46         0.24      -0.44           0.01
srh_item2_w1               1.00         0.21      -0.21           0.05
srh_item3_w1               0.21         1.00      -0.17           0.01
chronic_w1                -0.21        -0.17       1.00           0.11
rs_identity_w2             0.05         0.01       0.11           1.00
prayer_w2                  0.07         0.02       0.16           0.69
attendance_w2              0.04         0.01       0.15           0.57
commitment_w2              0.10        -0.06       0.10           0.58
rcope_ben_total_w2         0.01        -0.02       0.07           0.47
rcope_sup_total_w2         0.03        -0.02       0.12           0.67
srh_item1_w2               0.33         0.07      -0.35           0.00
srh_item2_w2               0.53         0.00      -0.16           0.08
srh_item3_w2               0.08         0.06      -0.17          -0.05
chronic_w2                -0.18         0.00       0.65           0.09
                   prayer_w2 attendance_w2 commitment_w2 rcope_ben_total_w2
rs_identity_w1          0.63          0.50          0.56               0.34
prayer_w1               0.80          0.52          0.60               0.51
attendance_w1           0.56          0.75          0.51               0.45
commitment_w1           0.57          0.42          0.57               0.39
rcope_ben_total_w1      0.48          0.36          0.42               0.52
rcope_sup_total_w1      0.68          0.47          0.55               0.53
srh_item1_w1            0.01          0.04          0.07              -0.01
srh_item2_w1            0.07          0.04          0.10               0.01
srh_item3_w1            0.02          0.01         -0.06              -0.02
chronic_w1              0.16          0.15          0.10               0.07
rs_identity_w2          0.69          0.57          0.58               0.47
prayer_w2               1.00          0.58          0.65               0.56
attendance_w2           0.58          1.00          0.51               0.48
commitment_w2           0.65          0.51          1.00               0.49
rcope_ben_total_w2      0.56          0.48          0.49               1.00
rcope_sup_total_w2      0.74          0.57          0.61               0.70
srh_item1_w2            0.05          0.01          0.06              -0.01
srh_item2_w2            0.09          0.13          0.13               0.09
srh_item3_w2           -0.04          0.02         -0.05              -0.02
chronic_w2              0.10          0.09          0.08               0.01
                   rcope_sup_total_w2 srh_item1_w2 srh_item2_w2 srh_item3_w2
rs_identity_w1                   0.56        -0.01         0.06        -0.04
prayer_w1                        0.69        -0.02         0.09        -0.06
attendance_w1                    0.53         0.03         0.10         0.00
commitment_w1                    0.56         0.00         0.08        -0.09
rcope_ben_total_w1               0.55         0.03         0.04         0.04
rcope_sup_total_w1               0.72         0.00         0.10         0.00
srh_item1_w1                    -0.03         0.55         0.33         0.03
srh_item2_w1                     0.03         0.33         0.53         0.08
srh_item3_w1                    -0.02         0.07         0.00         0.06
chronic_w1                       0.12        -0.35        -0.16        -0.17
rs_identity_w2                   0.67         0.00         0.08        -0.05
prayer_w2                        0.74         0.05         0.09        -0.04
attendance_w2                    0.57         0.01         0.13         0.02
commitment_w2                    0.61         0.06         0.13        -0.05
rcope_ben_total_w2               0.70        -0.01         0.09        -0.02
rcope_sup_total_w2               1.00         0.04         0.14         0.02
srh_item1_w2                     0.04         1.00         0.49         0.23
srh_item2_w2                     0.14         0.49         1.00         0.20
srh_item3_w2                     0.02         0.23         0.20         1.00
chronic_w2                       0.04        -0.45        -0.23        -0.16
                   chronic_w2
rs_identity_w1           0.12
prayer_w1                0.11
attendance_w1            0.05
commitment_w1            0.11
rcope_ben_total_w1      -0.07
rcope_sup_total_w1       0.08
srh_item1_w1            -0.31
srh_item2_w1            -0.18
srh_item3_w1             0.00
chronic_w1               0.65
rs_identity_w2           0.09
prayer_w2                0.10
attendance_w2            0.09
commitment_w2            0.08
rcope_ben_total_w2       0.01
rcope_sup_total_w2       0.04
srh_item1_w2            -0.45
srh_item2_w2            -0.23
srh_item3_w2            -0.16
chronic_w2               1.00

n
                   rs_identity_w1 prayer_w1 attendance_w1 commitment_w1
rs_identity_w1               2971      2953          2954          2942
prayer_w1                    2953      2990          2982          2970
attendance_w1                2954      2982          2989          2969
commitment_w1                2942      2970          2969          2979
rcope_ben_total_w1           2152      2160          2161          2155
rcope_sup_total_w1           2167      2179          2179          2171
srh_item1_w1                 2966      2985          2984          2974
srh_item2_w1                 2948      2966          2965          2956
srh_item3_w1                 2966      2985          2983          2973
chronic_w1                   2971      2990          2989          2979
rs_identity_w2                599       600           602           600
prayer_w2                     598       599           601           599
attendance_w2                 598       599           601           599
commitment_w2                 599       600           602           600
rcope_ben_total_w2            566       568           570           568
rcope_sup_total_w2            566       568           570           568
srh_item1_w2                  607       608           610           608
srh_item2_w2                  606       607           609           607
srh_item3_w2                  604       605           607           605
chronic_w2                    603       604           606           604
                   rcope_ben_total_w1 rcope_sup_total_w1 srh_item1_w1
rs_identity_w1                   2152               2167         2966
prayer_w1                        2160               2179         2985
attendance_w1                    2161               2179         2984
commitment_w1                    2155               2171         2974
rcope_ben_total_w1               2173               2162         2171
rcope_sup_total_w1               2162               2191         2189
srh_item1_w1                     2171               2189         3005
srh_item2_w1                     2154               2172         2983
srh_item3_w1                     2168               2186         2999
chronic_w1                       2173               2191         3005
rs_identity_w2                    436                438          606
prayer_w2                         436                438          605
attendance_w2                     436                438          605
commitment_w2                     436                438          606
rcope_ben_total_w2                425                427          573
rcope_sup_total_w2                425                427          573
srh_item1_w2                      443                445          614
srh_item2_w2                      442                444          613
srh_item3_w2                      441                443          611
chronic_w2                        440                442          610
                   srh_item2_w1 srh_item3_w1 chronic_w1 rs_identity_w2
rs_identity_w1             2948         2966       2971            599
prayer_w1                  2966         2985       2990            600
attendance_w1              2965         2983       2989            602
commitment_w1              2956         2973       2979            600
rcope_ben_total_w1         2154         2168       2173            436
rcope_sup_total_w1         2172         2186       2191            438
srh_item1_w1               2983         2999       3005            606
srh_item2_w1               2986         2981       2986            600
srh_item3_w1               2981         3004       3004            605
chronic_w1                 2986         3004       3010            607
rs_identity_w2              600          605        607            607
prayer_w2                   599          604        606            606
attendance_w2               599          604        606            606
commitment_w2               600          605        607            607
rcope_ben_total_w2          567          572        574            574
rcope_sup_total_w2          567          572        574            574
srh_item1_w2                608          613        615            607
srh_item2_w2                607          612        614            607
srh_item3_w2                605          610        612            607
chronic_w2                  604          609        611            607
                   prayer_w2 attendance_w2 commitment_w2 rcope_ben_total_w2
rs_identity_w1           598           598           599                566
prayer_w1                599           599           600                568
attendance_w1            601           601           602                570
commitment_w1            599           599           600                568
rcope_ben_total_w1       436           436           436                425
rcope_sup_total_w1       438           438           438                427
srh_item1_w1             605           605           606                573
srh_item2_w1             599           599           600                567
srh_item3_w1             604           604           605                572
chronic_w1               606           606           607                574
rs_identity_w2           606           606           607                574
prayer_w2                606           606           606                574
attendance_w2            606           606           606                574
commitment_w2            606           606           607                574
rcope_ben_total_w2       574           574           574                574
rcope_sup_total_w2       574           574           574                574
srh_item1_w2             606           606           607                574
srh_item2_w2             606           606           607                574
srh_item3_w2             606           606           607                574
chronic_w2               606           606           607                574
                   rcope_sup_total_w2 srh_item1_w2 srh_item2_w2 srh_item3_w2
rs_identity_w1                    566          607          606          604
prayer_w1                         568          608          607          605
attendance_w1                     570          610          609          607
commitment_w1                     568          608          607          605
rcope_ben_total_w1                425          443          442          441
rcope_sup_total_w1                427          445          444          443
srh_item1_w1                      573          614          613          611
srh_item2_w1                      567          608          607          605
srh_item3_w1                      572          613          612          610
chronic_w1                        574          615          614          612
rs_identity_w2                    574          607          607          607
prayer_w2                         574          606          606          606
attendance_w2                     574          606          606          606
commitment_w2                     574          607          607          607
rcope_ben_total_w2                574          574          574          574
rcope_sup_total_w2                574          574          574          574
srh_item1_w2                      574          615          614          612
srh_item2_w2                      574          614          614          612
srh_item3_w2                      574          612          612          612
chronic_w2                        574          611          611          611
                   chronic_w2
rs_identity_w1            603
prayer_w1                 604
attendance_w1             606
commitment_w1             604
rcope_ben_total_w1        440
rcope_sup_total_w1        442
srh_item1_w1              610
srh_item2_w1              604
srh_item3_w1              609
chronic_w1                611
rs_identity_w2            607
prayer_w2                 606
attendance_w2             606
commitment_w2             607
rcope_ben_total_w2        574
rcope_sup_total_w2        574
srh_item1_w2              611
srh_item2_w2              611
srh_item3_w2              611
chronic_w2                611

P
                   rs_identity_w1 prayer_w1 attendance_w1 commitment_w1
rs_identity_w1                    0.0000    0.0000        0.0000       
prayer_w1          0.0000                   0.0000        0.0000       
attendance_w1      0.0000         0.0000                  0.0000       
commitment_w1      0.0000         0.0000    0.0000                     
rcope_ben_total_w1 0.0000         0.0000    0.0000        0.0000       
rcope_sup_total_w1 0.0000         0.0000    0.0000        0.0000       
srh_item1_w1       0.7462         0.0072    0.0126        0.2174       
srh_item2_w1       0.0010         0.5069    0.0000        0.0000       
srh_item3_w1       0.9068         0.7170    0.1587        0.0292       
chronic_w1         0.0000         0.0000    0.0024        0.0000       
rs_identity_w2     0.0000         0.0000    0.0000        0.0000       
prayer_w2          0.0000         0.0000    0.0000        0.0000       
attendance_w2      0.0000         0.0000    0.0000        0.0000       
commitment_w2      0.0000         0.0000    0.0000        0.0000       
rcope_ben_total_w2 0.0000         0.0000    0.0000        0.0000       
rcope_sup_total_w2 0.0000         0.0000    0.0000        0.0000       
srh_item1_w2       0.7311         0.6987    0.4452        0.9069       
srh_item2_w2       0.1274         0.0330    0.0115        0.0500       
srh_item3_w2       0.2784         0.1610    0.9814        0.0247       
chronic_w2         0.0044         0.0047    0.2642        0.0065       
                   rcope_ben_total_w1 rcope_sup_total_w1 srh_item1_w1
rs_identity_w1     0.0000             0.0000             0.7462      
prayer_w1          0.0000             0.0000             0.0072      
attendance_w1      0.0000             0.0000             0.0126      
commitment_w1      0.0000             0.0000             0.2174      
rcope_ben_total_w1                    0.0000             0.0186      
rcope_sup_total_w1 0.0000                                0.0036      
srh_item1_w1       0.0186             0.0036                         
srh_item2_w1       0.0958             0.1988             0.0000      
srh_item3_w1       0.0556             0.9393             0.0000      
chronic_w1         0.0919             0.0000             0.0000      
rs_identity_w2     0.0000             0.0000             0.7595      
prayer_w2          0.0000             0.0000             0.7396      
attendance_w2      0.0000             0.0000             0.3200      
commitment_w2      0.0000             0.0000             0.0793      
rcope_ben_total_w2 0.0000             0.0000             0.7442      
rcope_sup_total_w2 0.0000             0.0000             0.4288      
srh_item1_w2       0.5844             0.9866             0.0000      
srh_item2_w2       0.3708             0.0287             0.0000      
srh_item3_w2       0.3814             0.9637             0.5074      
chronic_w2         0.1466             0.0786             0.0000      
                   srh_item2_w1 srh_item3_w1 chronic_w1 rs_identity_w2
rs_identity_w1     0.0010       0.9068       0.0000     0.0000        
prayer_w1          0.5069       0.7170       0.0000     0.0000        
attendance_w1      0.0000       0.1587       0.0024     0.0000        
commitment_w1      0.0000       0.0292       0.0000     0.0000        
rcope_ben_total_w1 0.0958       0.0556       0.0919     0.0000        
rcope_sup_total_w1 0.1988       0.9393       0.0000     0.0000        
srh_item1_w1       0.0000       0.0000       0.0000     0.7595        
srh_item2_w1                    0.0000       0.0000     0.2539        
srh_item3_w1       0.0000                    0.0000     0.8658        
chronic_w1         0.0000       0.0000                  0.0058        
rs_identity_w2     0.2539       0.8658       0.0058                   
prayer_w2          0.0694       0.6600       0.0000     0.0000        
attendance_w2      0.3279       0.8581       0.0002     0.0000        
commitment_w2      0.0167       0.1493       0.0144     0.0000        
rcope_ben_total_w2 0.8876       0.6510       0.1045     0.0000        
rcope_sup_total_w2 0.5470       0.6027       0.0033     0.0000        
srh_item1_w2       0.0000       0.0812       0.0000     0.9299        
srh_item2_w2       0.0000       0.9883       0.0000     0.0646        
srh_item3_w2       0.0615       0.1733       0.0000     0.2493        
chronic_w2         0.0000       0.9326       0.0000     0.0273        
                   prayer_w2 attendance_w2 commitment_w2 rcope_ben_total_w2
rs_identity_w1     0.0000    0.0000        0.0000        0.0000            
prayer_w1          0.0000    0.0000        0.0000        0.0000            
attendance_w1      0.0000    0.0000        0.0000        0.0000            
commitment_w1      0.0000    0.0000        0.0000        0.0000            
rcope_ben_total_w1 0.0000    0.0000        0.0000        0.0000            
rcope_sup_total_w1 0.0000    0.0000        0.0000        0.0000            
srh_item1_w1       0.7396    0.3200        0.0793        0.7442            
srh_item2_w1       0.0694    0.3279        0.0167        0.8876            
srh_item3_w1       0.6600    0.8581        0.1493        0.6510            
chronic_w1         0.0000    0.0002        0.0144        0.1045            
rs_identity_w2     0.0000    0.0000        0.0000        0.0000            
prayer_w2                    0.0000        0.0000        0.0000            
attendance_w2      0.0000                  0.0000        0.0000            
commitment_w2      0.0000    0.0000                      0.0000            
rcope_ben_total_w2 0.0000    0.0000        0.0000                          
rcope_sup_total_w2 0.0000    0.0000        0.0000        0.0000            
srh_item1_w2       0.2685    0.7408        0.1347        0.8928            
srh_item2_w2       0.0239    0.0017        0.0018        0.0400            
srh_item3_w2       0.3827    0.5470        0.1788        0.6926            
chronic_w2         0.0141    0.0197        0.0485        0.8796            
                   rcope_sup_total_w2 srh_item1_w2 srh_item2_w2 srh_item3_w2
rs_identity_w1     0.0000             0.7311       0.1274       0.2784      
prayer_w1          0.0000             0.6987       0.0330       0.1610      
attendance_w1      0.0000             0.4452       0.0115       0.9814      
commitment_w1      0.0000             0.9069       0.0500       0.0247      
rcope_ben_total_w1 0.0000             0.5844       0.3708       0.3814      
rcope_sup_total_w1 0.0000             0.9866       0.0287       0.9637      
srh_item1_w1       0.4288             0.0000       0.0000       0.5074      
srh_item2_w1       0.5470             0.0000       0.0000       0.0615      
srh_item3_w1       0.6027             0.0812       0.9883       0.1733      
chronic_w1         0.0033             0.0000       0.0000       0.0000      
rs_identity_w2     0.0000             0.9299       0.0646       0.2493      
prayer_w2          0.0000             0.2685       0.0239       0.3827      
attendance_w2      0.0000             0.7408       0.0017       0.5470      
commitment_w2      0.0000             0.1347       0.0018       0.1788      
rcope_ben_total_w2 0.0000             0.8928       0.0400       0.6926      
rcope_sup_total_w2                    0.3082       0.0009       0.6449      
srh_item1_w2       0.3082                          0.0000       0.0000      
srh_item2_w2       0.0009             0.0000                    0.0000      
srh_item3_w2       0.6449             0.0000       0.0000                   
chronic_w2         0.3179             0.0000       0.0000       0.0001      
                   chronic_w2
rs_identity_w1     0.0044    
prayer_w1          0.0047    
attendance_w1      0.2642    
commitment_w1      0.0065    
rcope_ben_total_w1 0.1466    
rcope_sup_total_w1 0.0786    
srh_item1_w1       0.0000    
srh_item2_w1       0.0000    
srh_item3_w1       0.9326    
chronic_w1         0.0000    
rs_identity_w2     0.0273    
prayer_w2          0.0141    
attendance_w2      0.0197    
commitment_w2      0.0485    
rcope_ben_total_w2 0.8796    
rcope_sup_total_w2 0.3179    
srh_item1_w2       0.0000    
srh_item2_w2       0.0000    
srh_item3_w2       0.0001    
chronic_w2
```

- Back to the Table of Contents

## Sample Attrition Analysis

Determine significant differences between participants who only
attended Wave 1 of the study and participants who also attended Wave 2
(i.e., sample attrition analysis). When looking at results,
**Group 1** refers to participants who attended Wave 2,
whereas **Group 0** refers to participants who only
attended Wave 1. For significant differences between groups, also
determine effect sizes.

```
# Sample Attrition Analysis
# Group 1 is those who attended Wave 2


# Assessing differences in age. Group 1 is those who attended Wave 2

t.test(age_w1 ~ attended_wave2, data = which_comes_first_data, var.equal = T)

    Two Sample t-test

data:  age_w1 by attended_wave2
t = -0.2815, df = 2952, p-value = 0.7783
alternative hypothesis: true difference in means between group 0 and group 1 is not equal to 0
95 percent confidence interval:
 -1.970286  1.475574
sample estimates:
mean in group 0 mean in group 1 
       51.58433        51.83168 

# Assessing differences in education. Group 1 attended Wave 2

t.test(education ~ attended_wave2, data = which_comes_first_data, var.equal = T)

    Two Sample t-test

data:  education by attended_wave2
t = -11.787, df = 3007, p-value < 2.2e-16
alternative hypothesis: true difference in means between group 0 and group 1 is not equal to 0
95 percent confidence interval:
 -1.919409 -1.371905
sample estimates:
mean in group 0 mean in group 1 
       13.13158        14.77724 

# Determine effect size of difference
psych::cohen.d(which_comes_first_data$education,
               which_comes_first_data$attended_wave2)
Call: psych::cohen.d(x = which_comes_first_data$education, group = which_comes_first_data$attended_wave2)
Cohen d statistic of difference between two means
     lower effect upper
[1,]  0.44   0.53  0.62

Multivariate (Mahalanobis) distance between groups
[1] 0.53
r equivalent of difference between two means
data 
0.21 

# Assessing differences in income. Group 1 attended Wave 2

t.test(income ~ attended_wave2, data = which_comes_first_data, var.equal = T)

    Two Sample t-test

data:  income by attended_wave2
t = -10.806, df = 2760, p-value < 2.2e-16
alternative hypothesis: true difference in means between group 0 and group 1 is not equal to 0
95 percent confidence interval:
 -1.903805 -1.318990
sample estimates:
mean in group 0 mean in group 1 
       6.453175        8.064572 

# Determine effect size of difference
psych::cohen.d(which_comes_first_data$income,
               which_comes_first_data$attended_wave2)
Call: psych::cohen.d(x = which_comes_first_data$income, group = which_comes_first_data$attended_wave2)
Cohen d statistic of difference between two means
     lower effect upper
[1,]  0.41   0.51   0.6

Multivariate (Mahalanobis) distance between groups
[1] 0.51
r equivalent of difference between two means
data 
 0.2 

# Differences in self-rated health

# Assessing differences in self-rated health item 1. Group 1 attended Wave 2

t.test(srh_item1_w1 ~ attended_wave2, data = which_comes_first_data,
       var.equal = T)

    Two Sample t-test

data:  srh_item1_w1 by attended_wave2
t = -5.6902, df = 3003, p-value = 1.391e-08
alternative hypothesis: true difference in means between group 0 and group 1 is not equal to 0
95 percent confidence interval:
 -0.2487782 -0.1212671
sample estimates:
mean in group 0 mean in group 1 
       1.901297        2.086319 

# Determine effect size of difference
psych::cohen.d(which_comes_first_data$srh_item1_w1,
               which_comes_first_data$attended_wave2)
Call: psych::cohen.d(x = which_comes_first_data$srh_item1_w1, group = which_comes_first_data$attended_wave2)
Cohen d statistic of difference between two means
     lower effect upper
[1,]  0.17   0.26  0.35

Multivariate (Mahalanobis) distance between groups
[1] 0.26
r equivalent of difference between two means
data 
 0.1 

# Assessing differences in self-rated health item 2. Group 1 attended Wave 2

t.test(srh_item2_w1 ~ attended_wave2, data = which_comes_first_data,
       var.equal = T)

    Two Sample t-test

data:  srh_item2_w1 by attended_wave2
t = -3.1029, df = 2984, p-value = 0.001934
alternative hypothesis: true difference in means between group 0 and group 1 is not equal to 0
95 percent confidence interval:
 -0.14729800 -0.03322466
sample estimates:
mean in group 0 mean in group 1 
       1.342304        1.432566 

# Determine effect size of difference
psych::cohen.d(which_comes_first_data$srh_item2_w1,
               which_comes_first_data$attended_wave2)
Call: psych::cohen.d(x = which_comes_first_data$srh_item2_w1, group = which_comes_first_data$attended_wave2)
Cohen d statistic of difference between two means
     lower effect upper
[1,]  0.05   0.14  0.23

Multivariate (Mahalanobis) distance between groups
[1] 0.14
r equivalent of difference between two means
data 
0.06 

# Assessing differences in self-rated health item 3. Group 1 attended Wave 2

t.test(srh_item3_w1 ~ attended_wave2, data = which_comes_first_data,
       var.equal = T)

    Two Sample t-test

data:  srh_item3_w1 by attended_wave2
t = -1.7199, df = 3002, p-value = 0.08555
alternative hypothesis: true difference in means between group 0 and group 1 is not equal to 0
95 percent confidence interval:
 -0.105386367  0.006895765
sample estimates:
mean in group 0 mean in group 1 
       1.102468        1.151713 

# Assessing differences in number of chronic illnesses. Group 1 attended Wave 2

t.test(chronic_w1 ~ attended_wave2, data = which_comes_first_data,
       var.equal = T)

    Two Sample t-test

data:  chronic_w1 by attended_wave2
t = 2.7041, df = 3008, p-value = 0.006887
alternative hypothesis: true difference in means between group 0 and group 1 is not equal to 0
95 percent confidence interval:
 0.05873338 0.36856604
sample estimates:
mean in group 0 mean in group 1 
       1.639666        1.426016 

# Determine effect size of difference
psych::cohen.d(which_comes_first_data$chronic_w1,
               which_comes_first_data$attended_wave2)
Call: psych::cohen.d(x = which_comes_first_data$chronic_w1, group = which_comes_first_data$attended_wave2)
Cohen d statistic of difference between two means
     lower effect upper
[1,] -0.21  -0.12 -0.03

Multivariate (Mahalanobis) distance between groups
[1] 0.12
r equivalent of difference between two means
 data 
-0.05 


# Differences in religious and spiritual engagement

# Assessing differences in Religious/Spiritual Identity. Group 1 attended Wave 2

t.test(rs_identity_w1 ~ attended_wave2, data = which_comes_first_data,
       var.equal = T)

    Two Sample t-test

data:  rs_identity_w1 by attended_wave2
t = -0.97442, df = 2969, p-value = 0.3299
alternative hypothesis: true difference in means between group 0 and group 1 is not equal to 0
95 percent confidence interval:
 -0.08678271  0.02916251
sample estimates:
mean in group 0 mean in group 1 
       1.453892        1.482702 

# Assessing differences in attendance frequency. Group 1 attended Wave 2

t.test(attendance_w1 ~ attended_wave2, data = which_comes_first_data,
       var.equal = T)

    Two Sample t-test

data:  attendance_w1 by attended_wave2
t = -2.2107, df = 2987, p-value = 0.02713
alternative hypothesis: true difference in means between group 0 and group 1 is not equal to 0
95 percent confidence interval:
 -0.53007123 -0.03176147
sample estimates:
mean in group 0 mean in group 1 
       3.714166        3.995082 

# Determine effect size of difference
psych::cohen.d(which_comes_first_data$attendance_w1,
               which_comes_first_data$attended_wave2)
Call: psych::cohen.d(x = which_comes_first_data$attendance_w1, group = which_comes_first_data$attended_wave2)
Cohen d statistic of difference between two means
     lower effect upper
[1,]  0.01    0.1  0.19

Multivariate (Mahalanobis) distance between groups
[1] 0.1
r equivalent of difference between two means
data 
0.04 

# Assessing differences in prayer frequency. Group 1 attended Wave 2

t.test(prayer_w1 ~ attended_wave2, data = which_comes_first_data, var.equal = T)

    Two Sample t-test

data:  prayer_w1 by attended_wave2
t = 1.6575, df = 2988, p-value = 0.09752
alternative hypothesis: true difference in means between group 0 and group 1 is not equal to 0
95 percent confidence interval:
 -0.03346416  0.39927518
sample estimates:
mean in group 0 mean in group 1 
       5.036524        4.853618 

# Assessing differences in commitment. Group 1 attended Wave 2

t.test(commitment_w1 ~ attended_wave2, data = which_comes_first_data,
       var.equal = T)

    Two Sample t-test

data:  commitment_w1 by attended_wave2
t = 0.31882, df = 2977, p-value = 0.7499
alternative hypothesis: true difference in means between group 0 and group 1 is not equal to 0
95 percent confidence interval:
 -0.0851202  0.1181759
sample estimates:
mean in group 0 mean in group 1 
       2.954028        2.937500 

# Assessing differences in benevolent coping. Group 1 attended Wave 2

t.test(rcope_ben_total_w1 ~ attended_wave2, data = which_comes_first_data,
       var.equal = T)

    Two Sample t-test

data:  rcope_ben_total_w1 by attended_wave2
t = 2.2068, df = 2171, p-value = 0.02744
alternative hypothesis: true difference in means between group 0 and group 1 is not equal to 0
95 percent confidence interval:
 0.02568508 0.43569881
sample estimates:
mean in group 0 mean in group 1 
       2.971098        2.740406 

# Determine effect size of difference
psych::cohen.d(which_comes_first_data$rcope_ben_total_w1,
               which_comes_first_data$attended_wave2)
Call: psych::cohen.d(x = which_comes_first_data$rcope_ben_total_w1, 
    group = which_comes_first_data$attended_wave2)
Cohen d statistic of difference between two means
     lower effect upper
[1,] -0.22  -0.12 -0.01

Multivariate (Mahalanobis) distance between groups
[1] 0.12
r equivalent of difference between two means
 data 
-0.05 

# Assessing differences in seeking God's social support. Group 1 attended Wave 2

t.test(rcope_sup_total_w1 ~ attended_wave2, data = which_comes_first_data,
       var.equal = T)

    Two Sample t-test

data:  rcope_sup_total_w1 by attended_wave2
t = 2.1395, df = 2189, p-value = 0.0325
alternative hypothesis: true difference in means between group 0 and group 1 is not equal to 0
95 percent confidence interval:
 0.01922362 0.44171052
sample estimates:
mean in group 0 mean in group 1 
       4.183276        3.952809 

# Determine effect size of difference
psych::cohen.d(which_comes_first_data$rcope_sup_total_w1,
               which_comes_first_data$attended_wave2)
Call: psych::cohen.d(x = which_comes_first_data$rcope_sup_total_w1, 
    group = which_comes_first_data$attended_wave2)
Cohen d statistic of difference between two means
     lower effect upper
[1,] -0.22  -0.11 -0.01

Multivariate (Mahalanobis) distance between groups
[1] 0.11
r equivalent of difference between two means
 data 
-0.05
```

- Back to the Table of Contents

# Primary Analyses

Primary analyses are divided into measurement models, longitudinal
measurement invariance models, and the structural models.

## Measurement Models

First establish separate measurement models of religious and
spiritual (R/S) engagement at Wave 1 and Wave 2, followed by the
measurement models of self-rated health (SRH) at Wave 1 and Wave 2.
Then, establish measurement models of both constructs across timepoints.
Finally, establish the full measurement model that includes both
constructs at both timepoints.

### Religious and Spiritual (R/S) Engagement

```
# Measurement Model of R/S Engagement at Wave 1

modelRSw1 <- "RS_Eng_W1 =~ rs_identity_w1 + prayer_w1 + attendance_w1 +
                  commitment_w1 + rcope_ben_total_w1 + rcope_sup_total_w1"

# Fitting the model to a CFA and running summary statistics

fitRSw1 <- cfa(data = which_comes_first_data, model = modelRSw1,
               estimator = "MLR", missing = "ML")
summary(fitRSw1, fit.measures = TRUE, standardized = TRUE)
lavaan 0.6.17 ended normally after 30 iterations

  Estimator                                         ML
  Optimization method                           NLMINB
  Number of model parameters                        18

  Number of observations                          3010
  Number of missing patterns                        23

Model Test User Model:
                                              Standard      Scaled
  Test Statistic                               378.708     347.108
  Degrees of freedom                                 9           9
  P-value (Chi-square)                           0.000       0.000
  Scaling correction factor                                  1.091
    Yuan-Bentler correction (Mplus variant)                       

Model Test Baseline Model:

  Test statistic                              6729.615    5529.478
  Degrees of freedom                                15          15
  P-value                                        0.000       0.000
  Scaling correction factor                                  1.217

User Model versus Baseline Model:

  Comparative Fit Index (CFI)                    0.945       0.939
  Tucker-Lewis Index (TLI)                       0.908       0.898
                                                                  
  Robust Comparative Fit Index (CFI)                         0.939
  Robust Tucker-Lewis Index (TLI)                            0.899

Loglikelihood and Information Criteria:

  Loglikelihood user model (H0)             -27802.997  -27802.997
  Scaling correction factor                                  1.041
      for the MLR correction                                      
  Loglikelihood unrestricted model (H1)     -27613.643  -27613.643
  Scaling correction factor                                  1.058
      for the MLR correction                                      
                                                                  
  Akaike (AIC)                               55641.995   55641.995
  Bayesian (BIC)                             55750.169   55750.169
  Sample-size adjusted Bayesian (SABIC)      55692.976   55692.976

Root Mean Square Error of Approximation:

  RMSEA                                          0.117       0.112
  90 Percent confidence interval - lower         0.107       0.102
  90 Percent confidence interval - upper         0.127       0.121
  P-value H_0: RMSEA <= 0.050                    0.000       0.000
  P-value H_0: RMSEA >= 0.080                    1.000       1.000
                                                                  
  Robust RMSEA                                               0.137
  90 Percent confidence interval - lower                     0.125
  90 Percent confidence interval - upper                     0.149
  P-value H_0: Robust RMSEA <= 0.050                         0.000
  P-value H_0: Robust RMSEA >= 0.080                         1.000

Standardized Root Mean Square Residual:

  SRMR                                           0.040       0.040

Parameter Estimates:

  Standard errors                             Sandwich
  Information bread                           Observed
  Observed information based on                Hessian

Latent Variables:
                   Estimate  Std.Err  z-value  P(>|z|)   Std.lv  Std.all
  RS_Eng_W1 =~                                                          
    rs_identity_w1    1.000                               0.446    0.686
    prayer_w1         4.485    0.108   41.706    0.000    1.999    0.821
    attendance_w1     3.787    0.108   35.017    0.000    1.688    0.602
    commitment_w1     1.714    0.056   30.425    0.000    0.764    0.668
    rcop_bn_ttl_w1    3.188    0.115   27.642    0.000    1.421    0.703
    rcop_sp_ttl_w1    4.165    0.125   33.365    0.000    1.856    0.875

Intercepts:
                   Estimate  Std.Err  z-value  P(>|z|)   Std.lv  Std.all
   .rs_identity_w1    1.458    0.012  122.594    0.000    1.458    2.245
   .prayer_w1         4.988    0.045  112.011    0.000    4.988    2.050
   .attendance_w1     3.761    0.051   73.486    0.000    3.761    1.341
   .commitment_w1     2.943    0.021  140.265    0.000    2.943    2.576
   .rcop_bn_ttl_w1    2.828    0.041   69.073    0.000    2.828    1.398
   .rcop_sp_ttl_w1    4.014    0.042   95.157    0.000    4.014    1.892

Variances:
                   Estimate  Std.Err  z-value  P(>|z|)   Std.lv  Std.all
   .rs_identity_w1    0.223    0.007   30.557    0.000    0.223    0.529
   .prayer_w1         1.929    0.093   20.840    0.000    1.929    0.326
   .attendance_w1     5.019    0.124   40.387    0.000    5.019    0.638
   .commitment_w1     0.722    0.026   27.300    0.000    0.722    0.553
   .rcop_bn_ttl_w1    2.071    0.077   26.845    0.000    2.071    0.506
   .rcop_sp_ttl_w1    1.058    0.073   14.507    0.000    1.058    0.235
    RS_Eng_W1         0.199    0.010   19.299    0.000    1.000    1.000


# Looking at Residuals

lavResiduals(fitRSw1)
$type
[1] "cor.bentler"

$cov
                   rs_d_1 pryr_1 attn_1 cmmt_1 rcp_b__1 rcp_s__1
rs_identity_w1      0.000                                       
prayer_w1           0.036  0.000                                
attendance_w1       0.084  0.008  0.001                         
commitment_w1       0.032  0.010  0.012  0.001                  
rcope_ben_total_w1 -0.096 -0.073 -0.041 -0.060   -0.021         
rcope_sup_total_w1 -0.067 -0.013 -0.050 -0.020    0.067   -0.017

$mean
    rs_identity_w1          prayer_w1      attendance_w1      commitment_w1 
             0.000              0.000              0.000              0.000 
rcope_ben_total_w1 rcope_sup_total_w1 
             0.007              0.001 

$cov.z
                    rs_d_1  pryr_1  attn_1  cmmt_1 rcp_b__1 rcp_s__1
rs_identity_w1       0.063                                          
prayer_w1            5.018   0.296                                  
attendance_w1        7.362   1.268   0.116                          
commitment_w1        3.273   1.492   1.235   0.225                  
rcope_ben_total_w1  -9.512 -10.751  -3.519  -5.428   -1.662         
rcope_sup_total_w1 -13.210  -3.917  -8.939  -2.966    7.042   -4.166

$mean.z
    rs_identity_w1          prayer_w1      attendance_w1      commitment_w1 
             4.899             -2.265             -5.633             -5.580 
rcope_ben_total_w1 rcope_sup_total_w1 
            11.177              1.997 

$summary
                           cov     mean  total
srmr                     0.045    0.003  0.040
srmr.se                  0.002    0.000  0.002
srmr.exactfit.z         16.327   16.063 16.335
srmr.exactfit.pvalue     0.000    0.000  0.000
usrmr                    0.044    0.003  0.039
usrmr.se                 0.003    0.000  0.003
usrmr.ci.lower           0.039    0.003  0.034
usrmr.ci.upper           0.050    0.003  0.044
usrmr.closefit.h0.value  0.050    0.050  0.050
usrmr.closefit.z        -1.781 -176.475 -3.852
usrmr.closefit.pvalue    0.963    1.000  1.000


# Looking at Modification Indices

modindices(fitRSw1, sort. = TRUE)
                  lhs op                rhs      mi    epc sepc.lv sepc.all
35 rcope_ben_total_w1 ~~ rcope_sup_total_w1 325.905  0.902   0.902    0.609
25     rs_identity_w1 ~~ rcope_sup_total_w1  89.865 -0.150  -0.150   -0.308
22     rs_identity_w1 ~~      attendance_w1  78.273  0.198   0.198    0.187
28          prayer_w1 ~~ rcope_ben_total_w1  74.960 -0.499  -0.499   -0.250
24     rs_identity_w1 ~~ rcope_ben_total_w1  56.568 -0.127  -0.127   -0.187
32      attendance_w1 ~~ rcope_sup_total_w1  47.792 -0.480  -0.480   -0.208
21     rs_identity_w1 ~~          prayer_w1  43.730  0.115   0.115    0.175
33      commitment_w1 ~~ rcope_ben_total_w1  21.244 -0.139  -0.139   -0.114
23     rs_identity_w1 ~~      commitment_w1  13.335  0.032   0.032    0.080
31      attendance_w1 ~~ rcope_ben_total_w1   6.520 -0.198  -0.198   -0.061
34      commitment_w1 ~~ rcope_sup_total_w1   3.534 -0.052  -0.052   -0.060
27          prayer_w1 ~~      commitment_w1   2.674  0.050   0.050    0.042
26          prayer_w1 ~~      attendance_w1   1.543  0.095   0.095    0.030
30      attendance_w1 ~~      commitment_w1   1.408  0.047   0.047    0.025
29          prayer_w1 ~~ rcope_sup_total_w1   0.648 -0.050  -0.050   -0.035
   sepc.nox
35    0.609
25   -0.308
22    0.187
28   -0.250
24   -0.187
32   -0.208
21    0.175
33   -0.114
23    0.080
31   -0.061
34   -0.060
27    0.042
26    0.030
30    0.025
29   -0.035


# Measurement Model of R/S Engagement at Wave 1 with Correlated errors
# between the two RCOPE subscales

modelRSw1_RCOPE <- "RS_Eng_W1 =~ rs_identity_w1 + prayer_w1 + attendance_w1 +
                      commitment_w1 + rcope_ben_total_w1 + rcope_sup_total_w1
rcope_ben_total_w1~~rcope_sup_total_w1"

# Fitting the model to a CFA and running summary statistics

fitRSw1_RCOPE <- cfa(data = which_comes_first_data, model = modelRSw1_RCOPE,
                     estimator = "MLR", missing = "ML")
summary(fitRSw1_RCOPE, fit.measures = TRUE, standardized = TRUE)
lavaan 0.6.17 ended normally after 36 iterations

  Estimator                                         ML
  Optimization method                           NLMINB
  Number of model parameters                        19

  Number of observations                          3010
  Number of missing patterns                        23

Model Test User Model:
                                              Standard      Scaled
  Test Statistic                                89.232      79.858
  Degrees of freedom                                 8           8
  P-value (Chi-square)                           0.000       0.000
  Scaling correction factor                                  1.117
    Yuan-Bentler correction (Mplus variant)                       

Model Test Baseline Model:

  Test statistic                              6729.615    5529.478
  Degrees of freedom                                15          15
  P-value                                        0.000       0.000
  Scaling correction factor                                  1.217

User Model versus Baseline Model:

  Comparative Fit Index (CFI)                    0.988       0.987
  Tucker-Lewis Index (TLI)                       0.977       0.976
                                                                  
  Robust Comparative Fit Index (CFI)                         0.988
  Robust Tucker-Lewis Index (TLI)                            0.978

Loglikelihood and Information Criteria:

  Loglikelihood user model (H0)             -27658.259  -27658.259
  Scaling correction factor                                  1.032
      for the MLR correction                                      
  Loglikelihood unrestricted model (H1)     -27613.643  -27613.643
  Scaling correction factor                                  1.058
      for the MLR correction                                      
                                                                  
  Akaike (AIC)                               55354.518   55354.518
  Bayesian (BIC)                             55468.702   55468.702
  Sample-size adjusted Bayesian (SABIC)      55408.332   55408.332

Root Mean Square Error of Approximation:

  RMSEA                                          0.058       0.055
  90 Percent confidence interval - lower         0.048       0.045
  90 Percent confidence interval - upper         0.069       0.065
  P-value H_0: RMSEA <= 0.050                    0.101       0.214
  P-value H_0: RMSEA >= 0.080                    0.000       0.000
                                                                  
  Robust RMSEA                                               0.064
  90 Percent confidence interval - lower                     0.052
  90 Percent confidence interval - upper                     0.078
  P-value H_0: Robust RMSEA <= 0.050                         0.031
  P-value H_0: Robust RMSEA >= 0.080                         0.026

Standardized Root Mean Square Residual:

  SRMR                                           0.018       0.018

Parameter Estimates:

  Standard errors                             Sandwich
  Information bread                           Observed
  Observed information based on                Hessian

Latent Variables:
                   Estimate  Std.Err  z-value  P(>|z|)   Std.lv  Std.all
  RS_Eng_W1 =~                                                          
    rs_identity_w1    1.000                               0.461    0.709
    prayer_w1         4.462    0.111   40.083    0.000    2.056    0.845
    attendance_w1     3.735    0.105   35.615    0.000    1.721    0.614
    commitment_w1     1.677    0.056   30.158    0.000    0.773    0.676
    rcop_bn_ttl_w1    2.601    0.098   26.610    0.000    1.199    0.599
    rcop_sp_ttl_w1    3.731    0.110   33.958    0.000    1.719    0.817

Covariances:
                        Estimate  Std.Err  z-value  P(>|z|)   Std.lv  Std.all
 .rcope_ben_total_w1 ~~                                                      
   .rcop_sp_ttl_w1         0.861    0.067   12.916    0.000    0.861    0.443

Intercepts:
                   Estimate  Std.Err  z-value  P(>|z|)   Std.lv  Std.all
   .rs_identity_w1    1.458    0.012  122.605    0.000    1.458    2.245
   .prayer_w1         4.989    0.045  112.026    0.000    4.989    2.050
   .attendance_w1     3.762    0.051   73.482    0.000    3.762    1.341
   .commitment_w1     2.943    0.021  140.216    0.000    2.943    2.576
   .rcop_bn_ttl_w1    2.842    0.041   69.761    0.000    2.842    1.419
   .rcop_sp_ttl_w1    4.020    0.042   95.870    0.000    4.020    1.911

Variances:
                   Estimate  Std.Err  z-value  P(>|z|)   Std.lv  Std.all
   .rs_identity_w1    0.210    0.007   29.643    0.000    0.210    0.497
   .prayer_w1         1.695    0.092   18.325    0.000    1.695    0.286
   .attendance_w1     4.905    0.124   39.526    0.000    4.905    0.623
   .commitment_w1     0.708    0.026   26.742    0.000    0.708    0.542
   .rcop_bn_ttl_w1    2.574    0.081   31.842    0.000    2.574    0.642
   .rcop_sp_ttl_w1    1.470    0.084   17.568    0.000    1.470    0.332
    RS_Eng_W1         0.212    0.010   20.456    0.000    1.000    1.000


# Comparing fit of the models

anova(fitRSw1, fitRSw1_RCOPE)

Scaled Chi-Squared Difference Test (method = "satorra.bentler.2001")

lavaan NOTE:
    The "Chisq" column contains standard test statistics, not the
    robust test that should be reported per model. A robust difference
    test is a function of two standard (not robust) statistics.
 
              Df   AIC   BIC   Chisq Chisq diff Df diff Pr(>Chisq)    
fitRSw1_RCOPE  8 55355 55469  89.232                                  
fitRSw1        9 55642 55750 378.708     328.85       1  < 2.2e-16 ***
---
Signif. codes:  0 '***' 0.001 '**' 0.01 '*' 0.05 '.' 0.1 ' ' 1


# Measurement Model of R/S Engagement at Wave 2

modelRSw2 <- "RS_Eng_W2 =~ rs_identity_w2 + prayer_w2 + attendance_w2 +
                  commitment_w2 + rcope_ben_total_w2 + rcope_sup_total_w2"

# Fitting the model to a CFA and running summary statistics

fitRSw2 <- cfa(data = which_comes_first_data, model = modelRSw2,
               estimator = "MLR", missing = "ML")
summary(fitRSw2, fit.measures = TRUE, standardized = TRUE)
lavaan 0.6.17 ended normally after 27 iterations

  Estimator                                         ML
  Optimization method                           NLMINB
  Number of model parameters                        18

                                                  Used       Total
  Number of observations                           607        3010
  Number of missing patterns                         3            

Model Test User Model:
                                              Standard      Scaled
  Test Statistic                                53.566      48.678
  Degrees of freedom                                 9           9
  P-value (Chi-square)                           0.000       0.000
  Scaling correction factor                                  1.100
    Yuan-Bentler correction (Mplus variant)                       

Model Test Baseline Model:

  Test statistic                              2056.027    1678.157
  Degrees of freedom                                15          15
  P-value                                        0.000       0.000
  Scaling correction factor                                  1.225

User Model versus Baseline Model:

  Comparative Fit Index (CFI)                    0.978       0.976
  Tucker-Lewis Index (TLI)                       0.964       0.960
                                                                  
  Robust Comparative Fit Index (CFI)                         0.978
  Robust Tucker-Lewis Index (TLI)                            0.964

Loglikelihood and Information Criteria:

  Loglikelihood user model (H0)              -6091.212   -6091.212
  Scaling correction factor                                  0.993
      for the MLR correction                                      
  Loglikelihood unrestricted model (H1)      -6064.429   -6064.429
  Scaling correction factor                                  1.029
      for the MLR correction                                      
                                                                  
  Akaike (AIC)                               12218.424   12218.424
  Bayesian (BIC)                             12297.778   12297.778
  Sample-size adjusted Bayesian (SABIC)      12240.632   12240.632

Root Mean Square Error of Approximation:

  RMSEA                                          0.090       0.085
  90 Percent confidence interval - lower         0.068       0.064
  90 Percent confidence interval - upper         0.114       0.108
  P-value H_0: RMSEA <= 0.050                    0.002       0.004
  P-value H_0: RMSEA >= 0.080                    0.787       0.675
                                                                  
  Robust RMSEA                                               0.093
  90 Percent confidence interval - lower                     0.069
  90 Percent confidence interval - upper                     0.119
  P-value H_0: Robust RMSEA <= 0.050                         0.002
  P-value H_0: Robust RMSEA >= 0.080                         0.821

Standardized Root Mean Square Residual:

  SRMR                                           0.023       0.023

Parameter Estimates:

  Standard errors                             Sandwich
  Information bread                           Observed
  Observed information based on                Hessian

Latent Variables:
                   Estimate  Std.Err  z-value  P(>|z|)   Std.lv  Std.all
  RS_Eng_W2 =~                                                          
    rs_identity_w2    1.000                               0.538    0.785
    prayer_w2         4.119    0.163   25.283    0.000    2.218    0.852
    attendance_w2     3.853    0.190   20.288    0.000    2.074    0.678
    commitment_w2     1.646    0.092   17.969    0.000    0.886    0.730
    rcop_bn_ttl_w2    3.011    0.163   18.498    0.000    1.621    0.740
    rcop_sp_ttl_w2    3.827    0.157   24.338    0.000    2.060    0.904

Intercepts:
                   Estimate  Std.Err  z-value  P(>|z|)   Std.lv  Std.all
   .rs_identity_w2    1.420    0.028   50.982    0.000    1.420    2.069
   .prayer_w2         4.634    0.106   43.852    0.000    4.634    1.780
   .attendance_w2     3.605    0.124   29.012    0.000    3.605    1.178
   .commitment_w2     2.880    0.049   58.455    0.000    2.880    2.373
   .rcop_bn_ttl_w2    2.625    0.088   29.680    0.000    2.625    1.199
   .rcop_sp_ttl_w2    3.696    0.094   39.299    0.000    3.696    1.622

Variances:
                   Estimate  Std.Err  z-value  P(>|z|)   Std.lv  Std.all
   .rs_identity_w2    0.181    0.012   14.575    0.000    0.181    0.384
   .prayer_w2         1.859    0.188    9.898    0.000    1.859    0.274
   .attendance_w2     5.056    0.299   16.940    0.000    5.056    0.540
   .commitment_w2     0.688    0.059   11.680    0.000    0.688    0.467
   .rcop_bn_ttl_w2    2.165    0.146   14.878    0.000    2.165    0.452
   .rcop_sp_ttl_w2    0.947    0.098    9.675    0.000    0.947    0.182
    RS_Eng_W2         0.290    0.025   11.701    0.000    1.000    1.000


# Looking at Residuals

lavResiduals(fitRSw2)
$type
[1] "cor.bentler"

$cov
                   rs_d_2 pryr_2 attn_2 cmmt_2 rcp_b__2 rcp_s__2
rs_identity_w2      0.000                                       
prayer_w2           0.022  0.000                                
attendance_w2       0.038 -0.003  0.000                         
commitment_w2       0.010  0.025  0.011  0.000                  
rcope_ben_total_w2 -0.077 -0.045 -0.004 -0.029   -0.017         
rcope_sup_total_w2 -0.012 -0.010 -0.023 -0.024    0.039   -0.009

$mean
    rs_identity_w2          prayer_w2      attendance_w2      commitment_w2 
             0.000              0.000              0.000              0.000 
rcope_ben_total_w2 rcope_sup_total_w2 
             0.008              0.003 

$cov.z
                   rs_d_2 pryr_2 attn_2 cmmt_2 rcp_b__2 rcp_s__2
rs_identity_w2      0.000                                       
prayer_w2           2.057  0.000                                
attendance_w2       2.028 -0.231  0.000                         
commitment_w2       0.636  1.577  0.620  0.000                  
rcope_ben_total_w2 -5.345 -3.969 -0.226 -1.514   -1.188         
rcope_sup_total_w2 -1.684 -2.104 -3.136 -2.644    3.287   -1.811

$mean.z
    rs_identity_w2          prayer_w2      attendance_w2      commitment_w2 
             0.000              0.000              0.000              0.000 
rcope_ben_total_w2 rcope_sup_total_w2 
             5.758              3.920 

$summary
                           cov    mean  total
srmr                     0.027   0.004  0.023
srmr.se                  0.003   0.000  0.003
srmr.exactfit.z          4.630   7.645  4.646
srmr.exactfit.pvalue     0.000   0.000  0.000
usrmr                    0.024   0.004  0.021
usrmr.se                 0.005   0.001  0.004
usrmr.ci.lower           0.016   0.003  0.015
usrmr.ci.upper           0.031   0.005  0.028
usrmr.closefit.h0.value  0.050   0.050  0.050
usrmr.closefit.z        -5.726 -75.847 -7.169
usrmr.closefit.pvalue    1.000   1.000  1.000


# Looking at Modification Indices

modindices(fitRSw2, sort. = TRUE)
                  lhs op                rhs     mi    epc sepc.lv sepc.all
35 rcope_ben_total_w2 ~~ rcope_sup_total_w2 43.501  0.600   0.600    0.419
24     rs_identity_w2 ~~ rcope_ben_total_w2 18.534 -0.132  -0.132   -0.212
28          prayer_w2 ~~ rcope_ben_total_w2  8.809 -0.320  -0.320   -0.160
22     rs_identity_w2 ~~      attendance_w2  5.703  0.107   0.107    0.112
21     rs_identity_w2 ~~          prayer_w2  5.115  0.073   0.073    0.126
27          prayer_w2 ~~      commitment_w2  4.862  0.131   0.131    0.116
34      commitment_w2 ~~ rcope_sup_total_w2  4.847 -0.111  -0.111   -0.138
32      attendance_w2 ~~ rcope_sup_total_w2  4.768 -0.285  -0.285   -0.130
29          prayer_w2 ~~ rcope_sup_total_w2  1.457 -0.131  -0.131   -0.099
33      commitment_w2 ~~ rcope_ben_total_w2  1.105 -0.061  -0.061   -0.050
25     rs_identity_w2 ~~ rcope_sup_total_w2  1.031 -0.029  -0.029   -0.069
23     rs_identity_w2 ~~      commitment_w2  0.473  0.012   0.012    0.033
30      attendance_w2 ~~      commitment_w2  0.381  0.052   0.052    0.028
31      attendance_w2 ~~ rcope_ben_total_w2  0.049  0.034   0.034    0.010
26          prayer_w2 ~~      attendance_w2  0.046 -0.033  -0.033   -0.011
   sepc.nox
35    0.419
24   -0.212
28   -0.160
22    0.112
21    0.126
27    0.116
34   -0.138
32   -0.130
29   -0.099
33   -0.050
25   -0.069
23    0.033
30    0.028
31    0.010
26   -0.011
```

- Back to the Table of Contents

### Self-Rated Health (SRH)

```
# Measurement Model of Self-Rated Health at Wave 1

modelSRHw1 <- "SRHW1 =~ srh_item1_w1 + srh_item2_w1 + srh_item3_w1 + chronic_w1"

# Fitting the model to a CFA and running summary statistics

fitSRHw1 <- cfa(data = which_comes_first_data, model = modelSRHw1,
                estimator = "MLR", missing = "ML")
summary(fitSRHw1, fit.measures = TRUE, standardized = TRUE)
lavaan 0.6.17 ended normally after 26 iterations

  Estimator                                         ML
  Optimization method                           NLMINB
  Number of model parameters                        12

  Number of observations                          3010
  Number of missing patterns                         6

Model Test User Model:
                                              Standard      Scaled
  Test Statistic                                36.583      34.121
  Degrees of freedom                                 2           2
  P-value (Chi-square)                           0.000       0.000
  Scaling correction factor                                  1.072
    Yuan-Bentler correction (Mplus variant)                       

Model Test Baseline Model:

  Test statistic                              1595.212    1471.968
  Degrees of freedom                                 6           6
  P-value                                        0.000       0.000
  Scaling correction factor                                  1.084

User Model versus Baseline Model:

  Comparative Fit Index (CFI)                    0.978       0.978
  Tucker-Lewis Index (TLI)                       0.935       0.934
                                                                  
  Robust Comparative Fit Index (CFI)                         0.978
  Robust Tucker-Lewis Index (TLI)                            0.935

Loglikelihood and Information Criteria:

  Loglikelihood user model (H0)             -14256.622  -14256.622
  Scaling correction factor                                  1.046
      for the MLR correction                                      
  Loglikelihood unrestricted model (H1)     -14238.331  -14238.331
  Scaling correction factor                                  1.050
      for the MLR correction                                      
                                                                  
  Akaike (AIC)                               28537.244   28537.244
  Bayesian (BIC)                             28609.360   28609.360
  Sample-size adjusted Bayesian (SABIC)      28571.232   28571.232

Root Mean Square Error of Approximation:

  RMSEA                                          0.076       0.073
  90 Percent confidence interval - lower         0.055       0.053
  90 Percent confidence interval - upper         0.098       0.095
  P-value H_0: RMSEA <= 0.050                    0.019       0.028
  P-value H_0: RMSEA >= 0.080                    0.405       0.319
                                                                  
  Robust RMSEA                                               0.076
  90 Percent confidence interval - lower                     0.055
  90 Percent confidence interval - upper                     0.099
  P-value H_0: Robust RMSEA <= 0.050                         0.024
  P-value H_0: Robust RMSEA >= 0.080                         0.414

Standardized Root Mean Square Residual:

  SRMR                                           0.022       0.022

Parameter Estimates:

  Standard errors                             Sandwich
  Information bread                           Observed
  Observed information based on                Hessian

Latent Variables:
                   Estimate  Std.Err  z-value  P(>|z|)   Std.lv  Std.all
  SRHW1 =~                                                              
    srh_item1_w1      1.000                               0.641    0.887
    srh_item2_w1      0.524    0.032   16.221    0.000    0.336    0.524
    srh_item3_w1      0.284    0.028    9.999    0.000    0.182    0.288
    chronic_w1       -1.325    0.080  -16.461    0.000   -0.849   -0.485

Intercepts:
                   Estimate  Std.Err  z-value  P(>|z|)   Std.lv  Std.all
   .srh_item1_w1      1.940    0.013  147.259    0.000    1.940    2.685
   .srh_item2_w1      1.360    0.012  115.887    0.000    1.360    2.120
   .srh_item3_w1      1.113    0.012   96.427    0.000    1.113    1.759
   .chronic_w1        1.596    0.032   50.056    0.000    1.596    0.912

Variances:
                   Estimate  Std.Err  z-value  P(>|z|)   Std.lv  Std.all
   .srh_item1_w1      0.111    0.022    5.052    0.000    0.111    0.213
   .srh_item2_w1      0.299    0.008   36.264    0.000    0.299    0.726
   .srh_item3_w1      0.367    0.009   42.638    0.000    0.367    0.917
   .chronic_w1        2.340    0.080   29.252    0.000    2.340    0.765
    SRHW1             0.410    0.025   16.272    0.000    1.000    1.000


# Looking at Residuals

lavResiduals(fitSRHw1)
$type
[1] "cor.bentler"

$cov
             sr_1_1 sr_2_1 sr_3_1 chrn_1
srh_item1_w1  0.000                     
srh_item2_w1  0.002  0.000              
srh_item3_w1 -0.015  0.064  0.000       
chronic_w1   -0.005  0.043 -0.026  0.000

$mean
srh_item1_w1 srh_item2_w1 srh_item3_w1   chronic_w1 
           0            0            0            0 

$cov.z
             sr_1_1 sr_2_1 sr_3_1 chrn_1
srh_item1_w1  0.000                     
srh_item2_w1  1.703  0.000              
srh_item3_w1 -6.215  4.555  0.000       
chronic_w1   -4.637  5.913 -1.858  0.000

$mean.z
srh_item1_w1 srh_item2_w1 srh_item3_w1   chronic_w1 
        0.00         5.97         0.00         0.00 

$summary
                           cov      mean  total
srmr                     0.026     0.000  0.022
srmr.se                  0.003     0.000  0.003
srmr.exactfit.z          5.827     7.737  5.827
srmr.exactfit.pvalue     0.000     0.000  0.000
usrmr                    0.026     0.000  0.022
usrmr.se                 0.005     0.000  0.004
usrmr.ci.lower           0.018     0.000  0.015
usrmr.ci.upper           0.033     0.000  0.028
usrmr.closefit.h0.value  0.050     0.050  0.050
usrmr.closefit.z        -5.289 -4966.389 -7.284
usrmr.closefit.pvalue    1.000     1.000  1.000


# Measurement Model of Self-Rated Health at Wave 1 Without SRH Item 3

modelSRHw1_3removed <- "SRHW1 =~ srh_item1_w1 + srh_item2_w1 + chronic_w1"

# Fitting the model to a CFA and running summary statistics

fitSRHw1_3removed <- cfa(data = which_comes_first_data,
                         model = modelSRHw1_3removed, estimator = "MLR",
                         missing = "ML")
summary(fitSRHw1_3removed, fit.measures = TRUE, standardized = TRUE)
lavaan 0.6.17 ended normally after 25 iterations

  Estimator                                         ML
  Optimization method                           NLMINB
  Number of model parameters                         9

  Number of observations                          3010
  Number of missing patterns                         4

Model Test User Model:
                                              Standard      Scaled
  Test Statistic                                 0.000       0.000
  Degrees of freedom                                 0           0

Model Test Baseline Model:

  Test statistic                              1359.269    1275.921
  Degrees of freedom                                 3           3
  P-value                                        0.000       0.000
  Scaling correction factor                                  1.065

User Model versus Baseline Model:

  Comparative Fit Index (CFI)                    1.000       1.000
  Tucker-Lewis Index (TLI)                       1.000       1.000
                                                                  
  Robust Comparative Fit Index (CFI)                         1.000
  Robust Tucker-Lewis Index (TLI)                            1.000

Loglikelihood and Information Criteria:

  Loglikelihood user model (H0)             -11469.618  -11469.618
  Loglikelihood unrestricted model (H1)     -11469.618  -11469.618
                                                                  
  Akaike (AIC)                               22957.236   22957.236
  Bayesian (BIC)                             23011.323   23011.323
  Sample-size adjusted Bayesian (SABIC)      22982.727   22982.727

Root Mean Square Error of Approximation:

  RMSEA                                          0.000          NA
  90 Percent confidence interval - lower         0.000          NA
  90 Percent confidence interval - upper         0.000          NA
  P-value H_0: RMSEA <= 0.050                       NA          NA
  P-value H_0: RMSEA >= 0.080                       NA          NA
                                                                  
  Robust RMSEA                                               0.000
  90 Percent confidence interval - lower                     0.000
  90 Percent confidence interval - upper                     0.000
  P-value H_0: Robust RMSEA <= 0.050                            NA
  P-value H_0: Robust RMSEA >= 0.080                            NA

Standardized Root Mean Square Residual:

  SRMR                                           0.000       0.000

Parameter Estimates:

  Standard errors                             Sandwich
  Information bread                           Observed
  Observed information based on                Hessian

Latent Variables:
                   Estimate  Std.Err  z-value  P(>|z|)   Std.lv  Std.all
  SRHW1 =~                                                              
    srh_item1_w1      1.000                               0.709    0.981
    srh_item2_w1      0.430    0.036   11.895    0.000    0.305    0.475
    chronic_w1       -1.095    0.095  -11.513    0.000   -0.777   -0.444

Intercepts:
                   Estimate  Std.Err  z-value  P(>|z|)   Std.lv  Std.all
   .srh_item1_w1      1.940    0.013  147.263    0.000    1.940    2.685
   .srh_item2_w1      1.360    0.012  115.901    0.000    1.360    2.121
   .chronic_w1        1.596    0.032   50.056    0.000    1.596    0.912

Variances:
                   Estimate  Std.Err  z-value  P(>|z|)   Std.lv  Std.all
   .srh_item1_w1      0.019    0.037    0.515    0.607    0.019    0.037
   .srh_item2_w1      0.319    0.009   36.016    0.000    0.319    0.775
   .chronic_w1        2.457    0.086   28.688    0.000    2.457    0.803
    SRHW1             0.503    0.039   12.776    0.000    1.000    1.000


# Measurement Model of Self-Rated Health at Wave 2

modelSRHw2 <- "SRHW2 =~ srh_item1_w2 + srh_item2_w2 + srh_item3_w2 + chronic_w2"

# Fitting the model to a CFA and running summary statistics

fitSRHw2 <- cfa(data = which_comes_first_data, model = modelSRHw2,
                estimator = "MLR", missing = "ML")
summary(fitSRHw2, fit.measures = TRUE, standardized = TRUE)
lavaan 0.6.17 ended normally after 24 iterations

  Estimator                                         ML
  Optimization method                           NLMINB
  Number of model parameters                        12

                                                  Used       Total
  Number of observations                           615        3010
  Number of missing patterns                         4            

Model Test User Model:
                                              Standard      Scaled
  Test Statistic                                 6.247       5.990
  Degrees of freedom                                 2           2
  P-value (Chi-square)                           0.044       0.050
  Scaling correction factor                                  1.043
    Yuan-Bentler correction (Mplus variant)                       

Model Test Baseline Model:

  Test statistic                               352.872     328.511
  Degrees of freedom                                 6           6
  P-value                                        0.000       0.000
  Scaling correction factor                                  1.074

User Model versus Baseline Model:

  Comparative Fit Index (CFI)                    0.988       0.988
  Tucker-Lewis Index (TLI)                       0.963       0.963
                                                                  
  Robust Comparative Fit Index (CFI)                         0.988
  Robust Tucker-Lewis Index (TLI)                            0.965

Loglikelihood and Information Criteria:

  Loglikelihood user model (H0)              -2851.389   -2851.389
  Scaling correction factor                                  1.026
      for the MLR correction                                      
  Loglikelihood unrestricted model (H1)      -2848.265   -2848.265
  Scaling correction factor                                  1.028
      for the MLR correction                                      
                                                                  
  Akaike (AIC)                                5726.778    5726.778
  Bayesian (BIC)                              5779.838    5779.838
  Sample-size adjusted Bayesian (SABIC)       5741.740    5741.740

Root Mean Square Error of Approximation:

  RMSEA                                          0.059       0.057
  90 Percent confidence interval - lower         0.008       0.005
  90 Percent confidence interval - upper         0.114       0.111
  P-value H_0: RMSEA <= 0.050                    0.309       0.329
  P-value H_0: RMSEA >= 0.080                    0.309       0.283
                                                                  
  Robust RMSEA                                               0.057
  90 Percent confidence interval - lower                     0.000
  90 Percent confidence interval - upper                     0.116
  P-value H_0: Robust RMSEA <= 0.050                         0.325
  P-value H_0: Robust RMSEA >= 0.080                         0.312

Standardized Root Mean Square Residual:

  SRMR                                           0.020       0.020

Parameter Estimates:

  Standard errors                             Sandwich
  Information bread                           Observed
  Observed information based on                Hessian

Latent Variables:
                   Estimate  Std.Err  z-value  P(>|z|)   Std.lv  Std.all
  SRHW2 =~                                                              
    srh_item1_w2      1.000                               0.656    0.917
    srh_item2_w2      0.534    0.077    6.921    0.000    0.351    0.540
    srh_item3_w2      0.252    0.062    4.098    0.000    0.166    0.260
    chronic_w2       -1.211    0.163   -7.406    0.000   -0.795   -0.492

Intercepts:
                   Estimate  Std.Err  z-value  P(>|z|)   Std.lv  Std.all
   .srh_item1_w2      2.008    0.029   69.589    0.000    2.008    2.806
   .srh_item2_w2      1.411    0.026   53.703    0.000    1.411    2.170
   .srh_item3_w2      1.033    0.026   40.039    0.000    1.033    1.622
   .chronic_w2        1.763    0.065   26.927    0.000    1.763    1.091

Variances:
                   Estimate  Std.Err  z-value  P(>|z|)   Std.lv  Std.all
   .srh_item1_w2      0.081    0.052    1.576    0.115    0.081    0.158
   .srh_item2_w2      0.300    0.019   15.831    0.000    0.300    0.709
   .srh_item3_w2      0.379    0.020   19.192    0.000    0.379    0.932
   .chronic_w2        1.979    0.137   14.445    0.000    1.979    0.758
    SRHW2             0.431    0.058    7.387    0.000    1.000    1.000


# Looking at Residuals

lavResiduals(fitSRHw1)
$type
[1] "cor.bentler"

$cov
             sr_1_1 sr_2_1 sr_3_1 chrn_1
srh_item1_w1  0.000                     
srh_item2_w1  0.002  0.000              
srh_item3_w1 -0.015  0.064  0.000       
chronic_w1   -0.005  0.043 -0.026  0.000

$mean
srh_item1_w1 srh_item2_w1 srh_item3_w1   chronic_w1 
           0            0            0            0 

$cov.z
             sr_1_1 sr_2_1 sr_3_1 chrn_1
srh_item1_w1  0.000                     
srh_item2_w1  1.703  0.000              
srh_item3_w1 -6.215  4.555  0.000       
chronic_w1   -4.637  5.913 -1.858  0.000

$mean.z
srh_item1_w1 srh_item2_w1 srh_item3_w1   chronic_w1 
        0.00         5.97         0.00         0.00 

$summary
                           cov      mean  total
srmr                     0.026     0.000  0.022
srmr.se                  0.003     0.000  0.003
srmr.exactfit.z          5.827     7.737  5.827
srmr.exactfit.pvalue     0.000     0.000  0.000
usrmr                    0.026     0.000  0.022
usrmr.se                 0.005     0.000  0.004
usrmr.ci.lower           0.018     0.000  0.015
usrmr.ci.upper           0.033     0.000  0.028
usrmr.closefit.h0.value  0.050     0.050  0.050
usrmr.closefit.z        -5.289 -4966.389 -7.284
usrmr.closefit.pvalue    1.000     1.000  1.000


# Measurement Model of Self-Rated Health at Wave 2 Without SRH Item 3

modelSRHw2_3removed <- "SRHW2 =~ srh_item1_w2 + srh_item2_w2 + chronic_w1"

# Fitting the model to a CFA and running summary statistics

fitSRHw2_3removed <- cfa(data = which_comes_first_data,
                         model = modelSRHw2_3removed, estimator = "MLR",
                         missing = "ML")
summary(fitSRHw2_3removed, fit.measures = TRUE, standardized = TRUE)
lavaan 0.6.17 ended normally after 26 iterations

  Estimator                                         ML
  Optimization method                           NLMINB
  Number of model parameters                         9

  Number of observations                          3010
  Number of missing patterns                         3

Model Test User Model:
                                              Standard      Scaled
  Test Statistic                                 0.000       0.000
  Degrees of freedom                                 0           0

Model Test Baseline Model:

  Test statistic                               251.971     237.027
  Degrees of freedom                                 3           3
  P-value                                        0.000       0.000
  Scaling correction factor                                  1.063

User Model versus Baseline Model:

  Comparative Fit Index (CFI)                    1.000       1.000
  Tucker-Lewis Index (TLI)                       1.000       1.000
                                                                  
  Robust Comparative Fit Index (CFI)                         1.000
  Robust Tucker-Lewis Index (TLI)                            1.000

Loglikelihood and Information Criteria:

  Loglikelihood user model (H0)              -7101.074   -7101.074
  Loglikelihood unrestricted model (H1)      -7101.074   -7101.074
                                                                  
  Akaike (AIC)                               14220.148   14220.148
  Bayesian (BIC)                             14274.235   14274.235
  Sample-size adjusted Bayesian (SABIC)      14245.638   14245.638

Root Mean Square Error of Approximation:

  RMSEA                                          0.000          NA
  90 Percent confidence interval - lower         0.000          NA
  90 Percent confidence interval - upper         0.000          NA
  P-value H_0: RMSEA <= 0.050                       NA          NA
  P-value H_0: RMSEA >= 0.080                       NA          NA
                                                                  
  Robust RMSEA                                               0.000
  90 Percent confidence interval - lower                     0.000
  90 Percent confidence interval - upper                     0.000
  P-value H_0: Robust RMSEA <= 0.050                            NA
  P-value H_0: Robust RMSEA >= 0.080                            NA

Standardized Root Mean Square Residual:

  SRMR                                           0.000       0.000

Parameter Estimates:

  Standard errors                             Sandwich
  Information bread                           Observed
  Observed information based on                Hessian

Latent Variables:
                   Estimate  Std.Err  z-value  P(>|z|)   Std.lv  Std.all
  SRHW2 =~                                                              
    srh_item1_w2      1.000                               0.748    1.032
    srh_item2_w2      0.422    0.100    4.210    0.000    0.316    0.485
    chronic_w1       -0.859    0.217   -3.956    0.000   -0.642   -0.367

Intercepts:
                   Estimate  Std.Err  z-value  P(>|z|)   Std.lv  Std.all
   .srh_item1_w2      1.981    0.028   71.053    0.000    1.981    2.735
   .srh_item2_w2      1.399    0.027   52.114    0.000    1.399    2.147
   .chronic_w1        1.596    0.032   50.056    0.000    1.596    0.912

Variances:
                   Estimate  Std.Err  z-value  P(>|z|)   Std.lv  Std.all
   .srh_item1_w2     -0.034    0.119   -0.287    0.774   -0.034   -0.065
   .srh_item2_w2      0.325    0.024   13.416    0.000    0.325    0.765
   .chronic_w1        2.648    0.153   17.353    0.000    2.648    0.865
    SRHW2             0.559    0.120    4.659    0.000    1.000    1.000
```

- Back to the Table of Contents

### R/S Engagement at both Timepoints

```
# Measurement Model of R/S Engagement at Both Waves

modelRSw1and2 <- "RS_Eng_W1 =~ rs_identity_w1 + prayer_w1 + attendance_w1 +
                      commitment_w1 + rcope_ben_total_w1 + rcope_sup_total_w1
RS_Eng_W2 =~ rs_identity_w2 + prayer_w2 + attendance_w2 + commitment_w2 +
                      rcope_ben_total_w2 + rcope_sup_total_w2"

# Fitting the model to a CFA and running summary statistics

fitRSw1and2 <- cfa(data = which_comes_first_data, model = modelRSw1and2,
                   estimator = "MLR", missing = "ML")
summary(fitRSw1and2, fit.measures = TRUE, standardized = TRUE)
lavaan 0.6.17 ended normally after 46 iterations

  Estimator                                         ML
  Optimization method                           NLMINB
  Number of model parameters                        37

  Number of observations                          3010
  Number of missing patterns                        37

Model Test User Model:
                                              Standard      Scaled
  Test Statistic                               940.102     797.203
  Degrees of freedom                                53          53
  P-value (Chi-square)                           0.000       0.000
  Scaling correction factor                                  1.179
    Yuan-Bentler correction (Mplus variant)                       

Model Test Baseline Model:

  Test statistic                             10104.231    8201.277
  Degrees of freedom                                66          66
  P-value                                        0.000       0.000
  Scaling correction factor                                  1.232

User Model versus Baseline Model:

  Comparative Fit Index (CFI)                    0.912       0.909
  Tucker-Lewis Index (TLI)                       0.890       0.886
                                                                  
  Robust Comparative Fit Index (CFI)                         0.854
  Robust Tucker-Lewis Index (TLI)                            0.818

Loglikelihood and Information Criteria:

  Loglikelihood user model (H0)             -33488.828  -33488.828
  Scaling correction factor                                  1.041
      for the MLR correction                                      
  Loglikelihood unrestricted model (H1)     -33018.777  -33018.777
  Scaling correction factor                                  1.122
      for the MLR correction                                      
                                                                  
  Akaike (AIC)                               67051.656   67051.656
  Bayesian (BIC)                             67274.015   67274.015
  Sample-size adjusted Bayesian (SABIC)      67156.452   67156.452

Root Mean Square Error of Approximation:

  RMSEA                                          0.075       0.068
  90 Percent confidence interval - lower         0.070       0.064
  90 Percent confidence interval - upper         0.079       0.072
  P-value H_0: RMSEA <= 0.050                    0.000       0.000
  P-value H_0: RMSEA >= 0.080                    0.017       0.000
                                                                  
  Robust RMSEA                                               0.149
  90 Percent confidence interval - lower                     0.139
  90 Percent confidence interval - upper                     0.159
  P-value H_0: Robust RMSEA <= 0.050                         0.000
  P-value H_0: Robust RMSEA >= 0.080                         1.000

Standardized Root Mean Square Residual:

  SRMR                                           0.064       0.064

Parameter Estimates:

  Standard errors                             Sandwich
  Information bread                           Observed
  Observed information based on                Hessian

Latent Variables:
                   Estimate  Std.Err  z-value  P(>|z|)   Std.lv  Std.all
  RS_Eng_W1 =~                                                          
    rs_identity_w1    1.000                               0.449    0.691
    prayer_w1         4.487    0.106   42.356    0.000    2.014    0.827
    attendance_w1     3.802    0.107   35.518    0.000    1.706    0.608
    commitment_w1     1.702    0.055   30.686    0.000    0.764    0.668
    rcop_bn_ttl_w1    3.129    0.111   28.190    0.000    1.405    0.694
    rcop_sp_ttl_w1    4.114    0.118   34.797    0.000    1.847    0.868
  RS_Eng_W2 =~                                                          
    rs_identity_w2    1.000                               0.497    0.757
    prayer_w2         4.218    0.163   25.899    0.000    2.095    0.851
    attendance_w2     3.870    0.189   20.533    0.000    1.922    0.650
    commitment_w2     1.668    0.090   18.594    0.000    0.828    0.710
    rcop_bn_ttl_w2    2.971    0.153   19.465    0.000    1.475    0.698
    rcop_sp_ttl_w2    3.843    0.146   26.372    0.000    1.908    0.884

Covariances:
                   Estimate  Std.Err  z-value  P(>|z|)   Std.lv  Std.all
  RS_Eng_W1 ~~                                                          
    RS_Eng_W2         0.209    0.011   18.564    0.000    0.938    0.938

Intercepts:
                   Estimate  Std.Err  z-value  P(>|z|)   Std.lv  Std.all
   .rs_identity_w1    1.458    0.012  122.635    0.000    1.458    2.245
   .prayer_w1         4.987    0.045  111.960    0.000    4.987    2.048
   .attendance_w1     3.761    0.051   73.472    0.000    3.761    1.340
   .commitment_w1     2.943    0.021  140.216    0.000    2.943    2.575
   .rcop_bn_ttl_w1    2.825    0.041   69.040    0.000    2.825    1.396
   .rcop_sp_ttl_w1    4.009    0.042   95.003    0.000    4.009    1.886
   .rs_identity_w2    1.444    0.021   67.223    0.000    1.444    2.202
   .prayer_w2         4.738    0.072   66.047    0.000    4.738    1.926
   .attendance_w2     3.700    0.105   35.343    0.000    3.700    1.251
   .commitment_w2     2.920    0.038   76.279    0.000    2.920    2.504
   .rcop_bn_ttl_w2    2.693    0.074   36.278    0.000    2.693    1.275
   .rcop_sp_ttl_w2    3.781    0.065   58.175    0.000    3.781    1.752

Variances:
                   Estimate  Std.Err  z-value  P(>|z|)   Std.lv  Std.all
   .rs_identity_w1    0.220    0.007   31.015    0.000    0.220    0.522
   .prayer_w1         1.873    0.091   20.624    0.000    1.873    0.316
   .attendance_w1     4.959    0.124   40.027    0.000    4.959    0.630
   .commitment_w1     0.722    0.026   27.649    0.000    0.722    0.553
   .rcop_bn_ttl_w1    2.123    0.078   27.250    0.000    2.123    0.518
   .rcop_sp_ttl_w1    1.112    0.072   15.496    0.000    1.112    0.246
   .rs_identity_w2    0.184    0.011   16.116    0.000    0.184    0.427
   .prayer_w2         1.668    0.160   10.425    0.000    1.668    0.275
   .attendance_w2     5.058    0.288   17.547    0.000    5.058    0.578
   .commitment_w2     0.674    0.057   11.897    0.000    0.674    0.495
   .rcop_bn_ttl_w2    2.285    0.149   15.346    0.000    2.285    0.512
   .rcop_sp_ttl_w2    1.015    0.091   11.113    0.000    1.015    0.218
    RS_Eng_W1         0.201    0.010   19.836    0.000    1.000    1.000
    RS_Eng_W2         0.247    0.019   13.175    0.000    1.000    1.000


# Looking at Residuals

lavResiduals(fitRSw1and2)
$type
[1] "cor.bentler"

$cov
                   rs_d_1 pryr_1 attn_1 cmmt_1 rcp_b__1 rcp_s__1 rs_d_2 pryr_2
rs_identity_w1      0.000                                                     
prayer_w1           0.027  0.000                                              
attendance_w1       0.075 -0.001  0.000                                       
commitment_w1       0.028  0.006  0.007  0.000                                
rcope_ben_total_w1 -0.093 -0.069 -0.042 -0.053   -0.022                       
rcope_sup_total_w1 -0.066 -0.012 -0.053 -0.015    0.079   -0.017              
rs_identity_w2      0.183 -0.009  0.060 -0.019   -0.121   -0.079  0.030       
prayer_w2           0.020  0.112  0.002 -0.016   -0.105   -0.023  0.023  0.001
attendance_w2       0.032 -0.050  0.373 -0.008   -0.102   -0.075  0.059 -0.020
commitment_w2       0.056 -0.008  0.058  0.092   -0.066   -0.035  0.023  0.005
rcope_ben_total_w2 -0.142 -0.049  0.021 -0.057    0.038   -0.046 -0.065 -0.051
rcope_sup_total_w2 -0.024 -0.011  0.000 -0.006   -0.051    0.003  0.013 -0.020
                   attn_2 cmmt_2 rcp_b__2 rcp_s__2
rs_identity_w1                                    
prayer_w1                                         
attendance_w1                                     
commitment_w1                                     
rcope_ben_total_w1                                
rcope_sup_total_w1                                
rs_identity_w2                                    
prayer_w2                                         
attendance_w2       0.033                         
commitment_w2       0.016  0.004                  
rcope_ben_total_w2  0.006 -0.025   -0.019         
rcope_sup_total_w2 -0.018 -0.022    0.063   -0.005

$mean
    rs_identity_w1          prayer_w1      attendance_w1      commitment_w1 
             0.000              0.000              0.000              0.000 
rcope_ben_total_w1 rcope_sup_total_w1     rs_identity_w2          prayer_w2 
             0.007              0.001             -0.046             -0.004 
     attendance_w2      commitment_w2 rcope_ben_total_w2 rcope_sup_total_w2 
            -0.078             -0.026              0.017              0.002 

$cov.z
                    rs_d_1  pryr_1  attn_1  cmmt_1 rcp_b__1 rcp_s__1  rs_d_2
rs_identity_w1      -0.057                                                  
prayer_w1            3.816   0.079                                          
attendance_w1        6.677  -0.113  -0.028                                  
commitment_w1        2.931   0.900   0.766   0.123                          
rcope_ben_total_w1  -9.079 -10.426  -3.590  -4.732   -1.476                 
rcope_sup_total_w1 -11.577  -3.078  -9.217  -2.015    7.496   -3.244        
rs_identity_w2       6.768  -0.489   2.499  -0.902   -4.930   -4.292   1.519
prayer_w2            0.847   5.453   0.076  -0.878   -5.230   -1.328   1.615
attendance_w2        1.385  -2.849  13.831  -0.423   -3.997   -3.844   2.740
commitment_w2        2.161  -0.359   2.249   3.295   -2.570   -1.642   1.229
rcope_ben_total_w2  -4.922  -2.521   0.713  -2.226    1.308   -2.317  -3.425
rcope_sup_total_w2  -0.948  -0.680  -0.013  -0.269   -2.780    0.185   0.944
                    pryr_2  attn_2  cmmt_2 rcp_b__2 rcp_s__2
rs_identity_w1                                              
prayer_w1                                                   
attendance_w1                                               
commitment_w1                                               
rcope_ben_total_w1                                          
rcope_sup_total_w1                                          
rs_identity_w2                                              
prayer_w2            0.056                                  
attendance_w2       -1.340   0.998                          
commitment_w2        0.282   0.756   0.357                  
rcope_ben_total_w2  -3.797   0.293  -1.196   -0.706         
rcope_sup_total_w2  -2.194  -1.335  -1.691    3.373   -0.443

$mean.z
    rs_identity_w1          prayer_w1      attendance_w1      commitment_w1 
            -1.537             -1.793             -4.462             -4.987 
rcope_ben_total_w1 rcope_sup_total_w1     rs_identity_w2          prayer_w2 
             9.070              1.766             -5.055             -0.485 
     attendance_w2      commitment_w2 rcope_ben_total_w2 rcope_sup_total_w2 
            -8.606             -2.920              1.551              0.161 

$summary
                           cov   mean  total
srmr                     0.068  0.028  0.064
srmr.se                  0.003  0.002  0.003
srmr.exactfit.z         17.651  9.293 17.552
srmr.exactfit.pvalue     0.000  0.000  0.000
usrmr                    0.065  0.027  0.061
usrmr.se                 0.003  0.003  0.003
usrmr.ci.lower           0.060  0.022  0.056
usrmr.ci.upper           0.070  0.032  0.066
usrmr.closefit.h0.value  0.050  0.050  0.050
usrmr.closefit.z         4.699 -7.546  3.675
usrmr.closefit.pvalue    0.000  1.000  0.000


# Looking at Modification Indices

modindices(fitRSw1and2, sort. = TRUE)
                   lhs op                rhs      mi    epc sepc.lv sepc.all
92  rcope_ben_total_w1 ~~ rcope_sup_total_w1 338.469  0.877   0.877    0.571
80       attendance_w1 ~~      attendance_w2 171.477  2.822   2.822    0.563
70           prayer_w1 ~~          prayer_w2  81.557  0.820   0.820    0.464
58      rs_identity_w1 ~~ rcope_sup_total_w1  76.088 -0.131  -0.131   -0.265
59      rs_identity_w1 ~~     rs_identity_w2  67.628  0.074   0.074    0.369
55      rs_identity_w1 ~~      attendance_w1  65.245  0.176   0.176    0.169
67           prayer_w1 ~~ rcope_ben_total_w1  60.728 -0.434  -0.434   -0.218
119 rcope_ben_total_w2 ~~ rcope_sup_total_w2  52.423  0.585   0.585    0.384
57      rs_identity_w1 ~~ rcope_ben_total_w1  50.944 -0.120  -0.120   -0.175
77       attendance_w1 ~~ rcope_sup_total_w1  47.817 -0.465  -0.465   -0.198
63      rs_identity_w1 ~~ rcope_ben_total_w2  26.533 -0.166  -0.166   -0.234
54      rs_identity_w1 ~~          prayer_w1  25.125  0.083   0.083    0.128
89       commitment_w1 ~~      commitment_w2  20.021  0.137   0.137    0.197
97  rcope_ben_total_w1 ~~ rcope_ben_total_w2  19.492  0.506   0.506    0.230
99  rcope_sup_total_w1 ~~     rs_identity_w2  16.609 -0.106  -0.106   -0.236
94  rcope_ben_total_w1 ~~          prayer_w2  15.393 -0.407  -0.407   -0.216
84       commitment_w1 ~~ rcope_ben_total_w1  15.374 -0.118  -0.118   -0.095
52           RS_Eng_W2 =~ rcope_ben_total_w1  14.064 -2.621  -1.301   -0.643
104 rcope_sup_total_w1 ~~ rcope_sup_total_w2  12.762  0.248   0.248    0.233
50           RS_Eng_W2 =~      attendance_w1  12.243  3.234   1.606    0.572
43           RS_Eng_W1 =~          prayer_w2  12.145  2.635   1.183    0.481
56      rs_identity_w1 ~~      commitment_w1  10.549  0.028   0.028    0.070
101 rcope_sup_total_w1 ~~      attendance_w2   9.245 -0.405  -0.405   -0.171
93  rcope_ben_total_w1 ~~     rs_identity_w2   9.114 -0.098  -0.098   -0.158
46           RS_Eng_W1 =~ rcope_ben_total_w2   8.895 -2.231  -1.001   -0.474
71           prayer_w1 ~~      attendance_w2   8.346 -0.415  -0.415   -0.135
108     rs_identity_w2 ~~ rcope_ben_total_w2   7.889 -0.084  -0.084   -0.130
53           RS_Eng_W2 =~ rcope_sup_total_w1   6.982 -1.596  -0.793   -0.373
112          prayer_w2 ~~ rcope_ben_total_w2   6.883 -0.255  -0.255   -0.131
76       attendance_w1 ~~ rcope_ben_total_w1   6.410 -0.195  -0.195   -0.060
83       attendance_w1 ~~ rcope_sup_total_w2   6.402 -0.283  -0.283   -0.126
106     rs_identity_w2 ~~      attendance_w2   5.895  0.104   0.104    0.108
113          prayer_w2 ~~ rcope_sup_total_w2   5.880 -0.201  -0.201   -0.154
64      rs_identity_w1 ~~ rcope_sup_total_w2   5.648 -0.057  -0.057   -0.121
79       attendance_w1 ~~          prayer_w2   4.457 -0.283  -0.283   -0.098
73           prayer_w1 ~~ rcope_ben_total_w2   4.277 -0.207  -0.207   -0.100
49           RS_Eng_W2 =~          prayer_w1   4.032  1.348   0.669    0.275
74           prayer_w1 ~~ rcope_sup_total_w2   3.801 -0.148  -0.148   -0.107
118      commitment_w2 ~~ rcope_sup_total_w2   3.655 -0.084  -0.084   -0.102
95  rcope_ben_total_w1 ~~      attendance_w2   3.563 -0.315  -0.315   -0.096
48           RS_Eng_W2 =~     rs_identity_w1   2.909  0.343   0.171    0.263
42           RS_Eng_W1 =~     rs_identity_w2   2.710 -0.357  -0.160   -0.244
90       commitment_w1 ~~ rcope_ben_total_w2   2.583 -0.093  -0.093   -0.072
86       commitment_w1 ~~     rs_identity_w2   2.330 -0.025  -0.025   -0.068
62      rs_identity_w1 ~~      commitment_w2   2.283  0.026   0.026    0.067
116      attendance_w2 ~~ rcope_sup_total_w2   2.065 -0.169  -0.169   -0.074
69           prayer_w1 ~~     rs_identity_w2   1.882 -0.039  -0.039   -0.066
88       commitment_w1 ~~      attendance_w2   1.617 -0.106  -0.106   -0.055
100 rcope_sup_total_w1 ~~          prayer_w2   1.547 -0.104  -0.104   -0.077
105     rs_identity_w2 ~~          prayer_w2   1.476  0.034   0.034    0.061
45           RS_Eng_W1 =~      commitment_w2   1.442  0.483   0.217    0.186
102 rcope_sup_total_w1 ~~      commitment_w2   1.393 -0.058  -0.058   -0.067
96  rcope_ben_total_w1 ~~      commitment_w2   1.276 -0.070  -0.070   -0.058
110          prayer_w2 ~~      attendance_w2   1.201 -0.153  -0.153   -0.053
72           prayer_w1 ~~      commitment_w2   1.056 -0.055  -0.055   -0.049
85       commitment_w1 ~~ rcope_sup_total_w1   1.047 -0.027  -0.027   -0.031
78       attendance_w1 ~~     rs_identity_w2   0.914  0.040   0.040    0.042
66           prayer_w1 ~~      commitment_w1   0.892  0.028   0.028    0.024
47           RS_Eng_W1 =~ rcope_sup_total_w2   0.785 -0.586  -0.263   -0.122
115      attendance_w2 ~~ rcope_ben_total_w2   0.614  0.119   0.119    0.035
75       attendance_w1 ~~      commitment_w1   0.547  0.029   0.029    0.015
61      rs_identity_w1 ~~      attendance_w2   0.547  0.034   0.034    0.032
98  rcope_ben_total_w1 ~~ rcope_sup_total_w2   0.474  0.059   0.059    0.040
87       commitment_w1 ~~          prayer_w2   0.440 -0.034  -0.034   -0.031
81       attendance_w1 ~~      commitment_w2   0.383  0.049   0.049    0.027
117      commitment_w2 ~~ rcope_ben_total_w2   0.347 -0.033  -0.033   -0.027
111          prayer_w2 ~~      commitment_w2   0.325  0.030   0.030    0.028
109     rs_identity_w2 ~~ rcope_sup_total_w2   0.320  0.013   0.013    0.031
82       attendance_w1 ~~ rcope_ben_total_w2   0.267  0.077   0.077    0.023
107     rs_identity_w2 ~~      commitment_w2   0.238  0.008   0.008    0.022
114      attendance_w2 ~~      commitment_w2   0.118  0.028   0.028    0.015
51           RS_Eng_W2 =~      commitment_w1   0.101 -0.114  -0.057   -0.050
68           prayer_w1 ~~ rcope_sup_total_w1   0.063 -0.014  -0.014   -0.010
91       commitment_w1 ~~ rcope_sup_total_w2   0.036  0.008   0.008    0.010
65           prayer_w1 ~~      attendance_w1   0.023 -0.011  -0.011   -0.004
60      rs_identity_w1 ~~          prayer_w2   0.021  0.004   0.004    0.007
44           RS_Eng_W1 =~      attendance_w2   0.002  0.047   0.021    0.007
103 rcope_sup_total_w1 ~~ rcope_ben_total_w2   0.002  0.004   0.004    0.002
    sepc.nox
92     0.571
80     0.563
70     0.464
58    -0.265
59     0.369
55     0.169
67    -0.218
119    0.384
57    -0.175
77    -0.198
63    -0.234
54     0.128
89     0.197
97     0.230
99    -0.236
94    -0.216
84    -0.095
52    -0.643
104    0.233
50     0.572
43     0.481
56     0.070
101   -0.171
93    -0.158
46    -0.474
71    -0.135
108   -0.130
53    -0.373
112   -0.131
76    -0.060
83    -0.126
106    0.108
113   -0.154
64    -0.121
79    -0.098
73    -0.100
49     0.275
74    -0.107
118   -0.102
95    -0.096
48     0.263
42    -0.244
90    -0.072
86    -0.068
62     0.067
116   -0.074
69    -0.066
88    -0.055
100   -0.077
105    0.061
45     0.186
102   -0.067
96    -0.058
110   -0.053
72    -0.049
85    -0.031
78     0.042
66     0.024
47    -0.122
115    0.035
75     0.015
61     0.032
98     0.040
87    -0.031
81     0.027
117   -0.027
111    0.028
109    0.031
82     0.023
107    0.022
114    0.015
51    -0.050
68    -0.010
91     0.010
65    -0.004
60     0.007
44     0.007
103    0.002


# Correlating Errors Between Each Wave 1 Indicator with its Respective Duplicate at Wave 2

modelRSw1and2_corr_duplicates <- "RS_Eng_W1 =~ rs_identity_w1 + prayer_w1 +
                                      attendance_w1 + commitment_w1 +
                                      rcope_ben_total_w1 + rcope_sup_total_w1
RS_Eng_W2 =~ rs_identity_w2 + prayer_w2 + attendance_w2 + commitment_w2 +
    rcope_ben_total_w2 + rcope_sup_total_w2
rs_identity_w1~~rs_identity_w2
prayer_w1~~prayer_w2
attendance_w1~~attendance_w2
commitment_w1~~commitment_w2
rcope_ben_total_w1~~rcope_ben_total_w2
rcope_sup_total_w1~~rcope_sup_total_w2"

# Fitting the model to a CFA and running summary statistics

fitRSw1and2_corr_duplicates <- cfa(data = which_comes_first_data,
                                   model = modelRSw1and2_corr_duplicates,
                                   estimator = "MLR", missing = "ML")
summary(fitRSw1and2_corr_duplicates, fit.measures = TRUE, standardized = TRUE)
lavaan 0.6.17 ended normally after 73 iterations

  Estimator                                         ML
  Optimization method                           NLMINB
  Number of model parameters                        43

  Number of observations                          3010
  Number of missing patterns                        37

Model Test User Model:
                                              Standard      Scaled
  Test Statistic                               482.648     446.555
  Degrees of freedom                                47          47
  P-value (Chi-square)                           0.000       0.000
  Scaling correction factor                                  1.081
    Yuan-Bentler correction (Mplus variant)                       

Model Test Baseline Model:

  Test statistic                             10104.231    8201.277
  Degrees of freedom                                66          66
  P-value                                        0.000       0.000
  Scaling correction factor                                  1.232

User Model versus Baseline Model:

  Comparative Fit Index (CFI)                    0.957       0.951
  Tucker-Lewis Index (TLI)                       0.939       0.931
                                                                  
  Robust Comparative Fit Index (CFI)                         0.963
  Robust Tucker-Lewis Index (TLI)                            0.948

Loglikelihood and Information Criteria:

  Loglikelihood user model (H0)             -33260.101  -33260.101
  Scaling correction factor                                  1.168
      for the MLR correction                                      
  Loglikelihood unrestricted model (H1)     -33018.777  -33018.777
  Scaling correction factor                                  1.122
      for the MLR correction                                      
                                                                  
  Akaike (AIC)                               66606.202   66606.202
  Bayesian (BIC)                             66864.619   66864.619
  Sample-size adjusted Bayesian (SABIC)      66727.992   66727.992

Root Mean Square Error of Approximation:

  RMSEA                                          0.055       0.053
  90 Percent confidence interval - lower         0.051       0.049
  90 Percent confidence interval - upper         0.060       0.058
  P-value H_0: RMSEA <= 0.050                    0.021       0.112
  P-value H_0: RMSEA >= 0.080                    0.000       0.000
                                                                  
  Robust RMSEA                                               0.080
  90 Percent confidence interval - lower                     0.069
  90 Percent confidence interval - upper                     0.091
  P-value H_0: Robust RMSEA <= 0.050                         0.000
  P-value H_0: Robust RMSEA >= 0.080                         0.493

Standardized Root Mean Square Residual:

  SRMR                                           0.042       0.042

Parameter Estimates:

  Standard errors                             Sandwich
  Information bread                           Observed
  Observed information based on                Hessian

Latent Variables:
                   Estimate  Std.Err  z-value  P(>|z|)   Std.lv  Std.all
  RS_Eng_W1 =~                                                          
    rs_identity_w1    1.000                               0.447    0.687
    prayer_w1         4.479    0.106   42.254    0.000    2.000    0.822
    attendance_w1     3.797    0.107   35.481    0.000    1.696    0.604
    commitment_w1     1.710    0.056   30.627    0.000    0.764    0.668
    rcop_bn_ttl_w1    3.178    0.113   28.216    0.000    1.419    0.701
    rcop_sp_ttl_w1    4.158    0.121   34.330    0.000    1.857    0.874
  RS_Eng_W2 =~                                                          
    rs_identity_w2    1.000                               0.498    0.750
    prayer_w2         4.115    0.164   25.096    0.000    2.047    0.832
    attendance_w2     3.680    0.180   20.443    0.000    1.831    0.616
    commitment_w2     1.656    0.092   18.056    0.000    0.824    0.706
    rcop_bn_ttl_w2    3.041    0.152   20.018    0.000    1.513    0.715
    rcop_sp_ttl_w2    3.914    0.149   26.353    0.000    1.947    0.901

Covariances:
                        Estimate  Std.Err  z-value  P(>|z|)   Std.lv  Std.all
 .rs_identity_w1 ~~                                                          
   .rs_identity_w2         0.089    0.011    7.786    0.000    0.089    0.429
 .prayer_w1 ~~                                                               
   .prayer_w2              0.864    0.130    6.636    0.000    0.864    0.457
 .attendance_w1 ~~                                                           
   .attendance_w2          3.273    0.242   13.495    0.000    3.273    0.625
 .commitment_w1 ~~                                                           
   .commitment_w2          0.129    0.039    3.328    0.001    0.129    0.183
 .rcope_ben_total_w1 ~~                                                      
   .rcop_bn_ttl_w2         0.405    0.120    3.388    0.001    0.405    0.190
 .rcope_sup_total_w1 ~~                                                      
   .rcop_sp_ttl_w2         0.112    0.095    1.185    0.236    0.112    0.116
  RS_Eng_W1 ~~                                                               
    RS_Eng_W2              0.201    0.011   18.455    0.000    0.905    0.905

Intercepts:
                   Estimate  Std.Err  z-value  P(>|z|)   Std.lv  Std.all
   .rs_identity_w1    1.458    0.012  122.668    0.000    1.458    2.243
   .prayer_w1         4.987    0.045  111.991    0.000    4.987    2.050
   .attendance_w1     3.761    0.051   73.475    0.000    3.761    1.340
   .commitment_w1     2.943    0.021  140.231    0.000    2.943    2.576
   .rcop_bn_ttl_w1    2.824    0.041   69.052    0.000    2.824    1.395
   .rcop_sp_ttl_w1    4.010    0.042   95.138    0.000    4.010    1.887
   .rs_identity_w2    1.425    0.021   67.891    0.000    1.425    2.147
   .prayer_w2         4.751    0.070   68.119    0.000    4.751    1.931
   .attendance_w2     3.492    0.095   36.808    0.000    3.492    1.175
   .commitment_w2     2.915    0.038   76.828    0.000    2.915    2.495
   .rcop_bn_ttl_w2    2.718    0.074   36.829    0.000    2.718    1.285
   .rcop_sp_ttl_w2    3.792    0.064   58.803    0.000    3.792    1.753

Variances:
                   Estimate  Std.Err  z-value  P(>|z|)   Std.lv  Std.all
   .rs_identity_w1    0.223    0.007   31.053    0.000    0.223    0.528
   .prayer_w1         1.916    0.091   21.038    0.000    1.916    0.324
   .attendance_w1     4.998    0.124   40.378    0.000    4.998    0.635
   .commitment_w1     0.722    0.026   27.453    0.000    0.722    0.553
   .rcop_bn_ttl_w1    2.085    0.077   27.075    0.000    2.085    0.509
   .rcop_sp_ttl_w1    1.069    0.071   14.969    0.000    1.069    0.237
   .rs_identity_w2    0.193    0.012   16.216    0.000    0.193    0.438
   .prayer_w2         1.865    0.175   10.647    0.000    1.865    0.308
   .attendance_w2     5.483    0.297   18.477    0.000    5.483    0.621
   .commitment_w2     0.685    0.058   11.818    0.000    0.685    0.502
   .rcop_bn_ttl_w2    2.185    0.146   14.959    0.000    2.185    0.488
   .rcop_sp_ttl_w2    0.884    0.091    9.699    0.000    0.884    0.189
    RS_Eng_W1         0.199    0.010   19.593    0.000    1.000    1.000
    RS_Eng_W2         0.248    0.018   13.400    0.000    1.000    1.000


# Comparing fit of the models

anova(fitRSw1and2_corr_duplicates, fitRSw1and2)

Scaled Chi-Squared Difference Test (method = "satorra.bentler.2001")

lavaan NOTE:
    The "Chisq" column contains standard test statistics, not the
    robust test that should be reported per model. A robust difference
    test is a function of two standard (not robust) statistics.
 
                            Df   AIC   BIC  Chisq Chisq diff Df diff Pr(>Chisq)
fitRSw1and2_corr_duplicates 47 66606 66865 482.65                              
fitRSw1and2                 53 67052 67274 940.10     234.56       6  < 2.2e-16
                               
fitRSw1and2_corr_duplicates    
fitRSw1and2                 ***
---
Signif. codes:  0 '***' 0.001 '**' 0.01 '*' 0.05 '.' 0.1 ' ' 1
```

- Back to the Table of Contents

### SRH at both Timepoints

```
# Measurement Model of Self-Rated Health at Both Waves

modelSRHw1and2 <- "SRHW1 =~ srh_item1_w1 + srh_item2_w1 + chronic_w1_reversed
SRHW2 =~ srh_item1_w2 + srh_item2_w2 + chronic_w2_reversed"

# Fitting the model to a CFA and running summary statistics

fitSRHw1and2 <- cfa(data = which_comes_first_data, model = modelSRHw1and2,
                    estimator = "MLR", missing = "ML")
summary(fitSRHw1and2, fit.measures = TRUE, standardized = TRUE)
lavaan 0.6.17 ended normally after 45 iterations

  Estimator                                         ML
  Optimization method                           NLMINB
  Number of model parameters                        19

  Number of observations                          3010
  Number of missing patterns                         9

Model Test User Model:
                                              Standard      Scaled
  Test Statistic                               397.121     343.108
  Degrees of freedom                                 8           8
  P-value (Chi-square)                           0.000       0.000
  Scaling correction factor                                  1.157
    Yuan-Bentler correction (Mplus variant)                       

Model Test Baseline Model:

  Test statistic                              2321.008    2010.100
  Degrees of freedom                                15          15
  P-value                                        0.000       0.000
  Scaling correction factor                                  1.155

User Model versus Baseline Model:

  Comparative Fit Index (CFI)                    0.831       0.832
  Tucker-Lewis Index (TLI)                       0.684       0.685
                                                                  
  Robust Comparative Fit Index (CFI)                         0.685
  Robust Tucker-Lewis Index (TLI)                            0.410

Loglikelihood and Information Criteria:

  Loglikelihood user model (H0)             -13619.686  -13619.686
  Scaling correction factor                                  1.092
      for the MLR correction                                      
  Loglikelihood unrestricted model (H1)     -13421.126  -13421.126
  Scaling correction factor                                  1.111
      for the MLR correction                                      
                                                                  
  Akaike (AIC)                               27277.372   27277.372
  Bayesian (BIC)                             27391.557   27391.557
  Sample-size adjusted Bayesian (SABIC)      27331.186   27331.186

Root Mean Square Error of Approximation:

  RMSEA                                          0.127       0.118
  90 Percent confidence interval - lower         0.117       0.108
  90 Percent confidence interval - upper         0.138       0.128
  P-value H_0: RMSEA <= 0.050                    0.000       0.000
  P-value H_0: RMSEA >= 0.080                    1.000       1.000
                                                                  
  Robust RMSEA                                               0.293
  90 Percent confidence interval - lower                     0.268
  90 Percent confidence interval - upper                     0.319
  P-value H_0: Robust RMSEA <= 0.050                         0.000
  P-value H_0: Robust RMSEA >= 0.080                         1.000

Standardized Root Mean Square Residual:

  SRMR                                           0.114       0.114

Parameter Estimates:

  Standard errors                             Sandwich
  Information bread                           Observed
  Observed information based on                Hessian

Latent Variables:
                   Estimate  Std.Err  z-value  P(>|z|)   Std.lv  Std.all
  SRHW1 =~                                                              
    srh_item1_w1      1.000                               0.633    0.877
    srh_item2_w1      0.532    0.033   15.957    0.000    0.337    0.525
    chrnc_w1_rvrsd    1.366    0.094   14.479    0.000    0.865    0.494
  SRHW2 =~                                                              
    srh_item1_w2      1.000                               0.632    0.864
    srh_item2_w2      0.599    0.057   10.559    0.000    0.379    0.577
    chrnc_w2_rvrsd    1.380    0.145    9.537    0.000    0.872    0.535

Covariances:
                   Estimate  Std.Err  z-value  P(>|z|)   Std.lv  Std.all
  SRHW1 ~~                                                              
    SRHW2             0.312    0.020   15.720    0.000    0.779    0.779

Intercepts:
                   Estimate  Std.Err  z-value  P(>|z|)   Std.lv  Std.all
   .srh_item1_w1      1.940    0.013  147.259    0.000    1.940    2.685
   .srh_item2_w1      1.360    0.012  115.880    0.000    1.360    2.120
   .chrnc_w1_rvrsd    8.404    0.032  263.575    0.000    8.404    4.804
   .srh_item1_w2      1.918    0.026   73.817    0.000    1.918    2.624
   .srh_item2_w2      1.357    0.027   50.675    0.000    1.357    2.068
   .chrnc_w2_rvrsd    8.112    0.067  121.459    0.000    8.112    4.980

Variances:
                   Estimate  Std.Err  z-value  P(>|z|)   Std.lv  Std.all
   .srh_item1_w1      0.121    0.024    5.071    0.000    0.121    0.232
   .srh_item2_w1      0.298    0.008   36.451    0.000    0.298    0.724
   .chrnc_w1_rvrsd    2.312    0.082   28.157    0.000    2.312    0.756
   .srh_item1_w2      0.136    0.028    4.795    0.000    0.136    0.254
   .srh_item2_w2      0.287    0.016   18.111    0.000    0.287    0.667
   .chrnc_w2_rvrsd    1.894    0.124   15.211    0.000    1.894    0.714
    SRHW1             0.401    0.027   14.694    0.000    1.000    1.000
    SRHW2             0.399    0.040   10.047    0.000    1.000    1.000


# Looking at Residuals

lavResiduals(fitSRHw1and2)
$type
[1] "cor.bentler"

$cov
                    sr_1_1 sr_2_1 chr_1_ sr_1_2 sr_2_2 chr_2_
srh_item1_w1         0.000                                   
srh_item2_w1         0.006  0.000                            
chronic_w1_reversed  0.002 -0.049  0.000                     
srh_item1_w2        -0.012 -0.019  0.058  0.006              
srh_item2_w2        -0.060  0.290 -0.063 -0.007 -0.015       
chronic_w2_reversed  0.009 -0.041  0.490  0.038 -0.072  0.070

$mean
       srh_item1_w1        srh_item2_w1 chronic_w1_reversed        srh_item1_w2 
              0.000               0.000               0.000               0.002 
       srh_item2_w2 chronic_w2_reversed 
              0.007              -0.006 

$cov.z
                    sr_1_1 sr_2_1 chr_1_ sr_1_2 sr_2_2 chr_2_
srh_item1_w1         0.000                                   
srh_item2_w1         2.979 -0.045                            
chronic_w1_reversed  0.879 -6.627  0.000                     
srh_item1_w2        -1.459 -0.748  1.800  3.335              
srh_item2_w2        -2.207  9.214 -1.931 -0.612 -0.515       
chronic_w2_reversed  0.303 -1.453 13.505  2.648 -3.517  1.935

$mean.z
       srh_item1_w1        srh_item2_w1 chronic_w1_reversed        srh_item1_w2 
              0.000              -5.575               0.000               2.095 
       srh_item2_w2 chronic_w2_reversed 
              1.279              -1.005 

$summary
                           cov    mean  total
srmr                     0.129   0.004  0.114
srmr.se                  0.007   0.002  0.007
srmr.exactfit.z         14.571   0.575 14.520
srmr.exactfit.pvalue     0.000   0.283  0.000
usrmr                    0.128   0.003  0.113
usrmr.se                 0.008   0.005  0.007
usrmr.ci.lower           0.115  -0.005  0.102
usrmr.ci.upper           0.140   0.010  0.124
usrmr.closefit.h0.value  0.050   0.050  0.050
usrmr.closefit.z        10.221 -10.384  9.336
usrmr.closefit.pvalue    0.000   1.000  0.000


# Looking at Modification Indices

modindices(fitSRHw1and2, sort. = TRUE)
                   lhs op                 rhs      mi    epc sepc.lv sepc.all
41 chronic_w1_reversed ~~ chronic_w2_reversed 184.105  1.235   1.235    0.590
37        srh_item2_w1 ~~        srh_item2_w2 105.745  0.134   0.134    0.457
35        srh_item2_w1 ~~ chronic_w1_reversed  29.942 -0.127  -0.127   -0.153
27               SRHW2 =~        srh_item1_w1  29.941 -0.867  -0.548   -0.758
30        srh_item1_w1 ~~        srh_item2_w1  21.442  0.091   0.091    0.481
29               SRHW2 =~ chronic_w1_reversed  21.442  1.165   0.736    0.420
34        srh_item1_w1 ~~ chronic_w2_reversed  17.820 -0.144  -0.144   -0.302
33        srh_item1_w1 ~~        srh_item2_w2  13.816 -0.052  -0.052   -0.278
36        srh_item2_w1 ~~        srh_item1_w2  11.914 -0.045  -0.045   -0.223
40 chronic_w1_reversed ~~        srh_item2_w2   8.708 -0.106  -0.106   -0.130
24               SRHW1 =~        srh_item1_w2   7.860 -0.590  -0.374   -0.511
44        srh_item2_w2 ~~ chronic_w2_reversed   7.860 -0.098  -0.098   -0.133
38        srh_item2_w1 ~~ chronic_w2_reversed   5.250 -0.076  -0.076   -0.101
32        srh_item1_w1 ~~        srh_item1_w2   3.894  0.035   0.035    0.277
26               SRHW1 =~ chronic_w2_reversed   2.963  0.435   0.275    0.169
42        srh_item1_w2 ~~        srh_item2_w2   2.963  0.038   0.038    0.193
31        srh_item1_w1 ~~ chronic_w1_reversed   2.733  0.080   0.080    0.152
28               SRHW2 =~        srh_item2_w1   2.733  0.155   0.098    0.153
39 chronic_w1_reversed ~~        srh_item1_w2   1.876 -0.048  -0.048   -0.086
25               SRHW1 =~        srh_item2_w2   0.489  0.075   0.048    0.072
43        srh_item1_w2 ~~ chronic_w2_reversed   0.489  0.035   0.035    0.069
   sepc.nox
41    0.590
37    0.457
35   -0.153
27   -0.758
30    0.481
29    0.420
34   -0.302
33   -0.278
36   -0.223
40   -0.130
24   -0.511
44   -0.133
38   -0.101
32    0.277
26    0.169
42    0.193
31    0.152
28    0.153
39   -0.086
25    0.072
43    0.069


# Correlating Errors Between Each Wave 1 Indicator with its Respective
# Duplicate at Wave 2

modelSRHw1and2_corr_duplicates <- "SRHW1 =~ srh_item1_w1 + srh_item2_w1 +
                                      chronic_w1_reversed
SRHW2 =~ srh_item1_w2 + srh_item2_w2 + chronic_w2_reversed
srh_item1_w1~~srh_item1_w2
srh_item2_w1~~srh_item2_w2
chronic_w1_reversed~~chronic_w2_reversed"

# Fitting the model to a CFA and running summary statistics

fitSRHw1and2_corr_duplicates <- cfa(data = which_comes_first_data,
                                    model = modelSRHw1and2_corr_duplicates,
                                    estimator = "MLR", missing = "ML")
summary(fitSRHw1and2_corr_duplicates, fit.measures = TRUE, standardized = TRUE)
lavaan 0.6.17 ended normally after 50 iterations

  Estimator                                         ML
  Optimization method                           NLMINB
  Number of model parameters                        22

  Number of observations                          3010
  Number of missing patterns                         9

Model Test User Model:
                                              Standard      Scaled
  Test Statistic                                 7.412       6.421
  Degrees of freedom                                 5           5
  P-value (Chi-square)                           0.192       0.267
  Scaling correction factor                                  1.154
    Yuan-Bentler correction (Mplus variant)                       

Model Test Baseline Model:

  Test statistic                              2321.008    2010.100
  Degrees of freedom                                15          15
  P-value                                        0.000       0.000
  Scaling correction factor                                  1.155

User Model versus Baseline Model:

  Comparative Fit Index (CFI)                    0.999       0.999
  Tucker-Lewis Index (TLI)                       0.997       0.998
                                                                  
  Robust Comparative Fit Index (CFI)                         0.998
  Robust Tucker-Lewis Index (TLI)                            0.995

Loglikelihood and Information Criteria:

  Loglikelihood user model (H0)             -13424.832  -13424.832
  Scaling correction factor                                  1.101
      for the MLR correction                                      
  Loglikelihood unrestricted model (H1)     -13421.126  -13421.126
  Scaling correction factor                                  1.111
      for the MLR correction                                      
                                                                  
  Akaike (AIC)                               26893.664   26893.664
  Bayesian (BIC)                             27025.878   27025.878
  Sample-size adjusted Bayesian (SABIC)      26955.975   26955.975

Root Mean Square Error of Approximation:

  RMSEA                                          0.013       0.010
  90 Percent confidence interval - lower         0.000       0.000
  90 Percent confidence interval - upper         0.030       0.027
  P-value H_0: RMSEA <= 0.050                    1.000       1.000
  P-value H_0: RMSEA >= 0.080                    0.000       0.000
                                                                  
  Robust RMSEA                                               0.027
  90 Percent confidence interval - lower                     0.000
  90 Percent confidence interval - upper                     0.072
  P-value H_0: Robust RMSEA <= 0.050                         0.758
  P-value H_0: Robust RMSEA >= 0.080                         0.021

Standardized Root Mean Square Residual:

  SRMR                                           0.018       0.018

Parameter Estimates:

  Standard errors                             Sandwich
  Information bread                           Observed
  Observed information based on                Hessian

Latent Variables:
                   Estimate  Std.Err  z-value  P(>|z|)   Std.lv  Std.all
  SRHW1 =~                                                              
    srh_item1_w1      1.000                               0.708    0.980
    srh_item2_w1      0.431    0.035   12.334    0.000    0.305    0.475
    chrnc_w1_rvrsd    1.096    0.092   11.913    0.000    0.776    0.444
  SRHW2 =~                                                              
    srh_item1_w2      1.000                               0.747    1.022
    srh_item2_w2      0.432    0.047    9.106    0.000    0.323    0.492
    chrnc_w2_rvrsd    1.008    0.117    8.649    0.000    0.753    0.451

Covariances:
                         Estimate  Std.Err  z-value  P(>|z|)   Std.lv  Std.all
 .srh_item1_w1 ~~                                                             
   .srh_item1_w2           -0.100    0.032   -3.093    0.002   -0.100   -4.431
 .srh_item2_w1 ~~                                                             
   .srh_item2_w2            0.156    0.013   11.705    0.000    0.156    0.483
 .chronic_w1_reversed ~~                                                      
   .chrnc_w2_rvrsd          1.515    0.104   14.557    0.000    1.515    0.650
  SRHW1 ~~                                                                    
    SRHW2                   0.402    0.037   10.831    0.000    0.761    0.761

Intercepts:
                   Estimate  Std.Err  z-value  P(>|z|)   Std.lv  Std.all
   .srh_item1_w1      1.940    0.013  147.261    0.000    1.940    2.685
   .srh_item2_w1      1.360    0.012  115.900    0.000    1.360    2.118
   .chrnc_w1_rvrsd    8.404    0.032  263.575    0.000    8.404    4.812
   .srh_item1_w2      1.920    0.026   73.871    0.000    1.920    2.628
   .srh_item2_w2      1.357    0.025   55.273    0.000    1.357    2.068
   .chrnc_w2_rvrsd    8.112    0.057  142.827    0.000    8.112    4.858

Variances:
                   Estimate  Std.Err  z-value  P(>|z|)   Std.lv  Std.all
   .srh_item1_w1      0.021    0.036    0.581    0.561    0.021    0.040
   .srh_item2_w1      0.319    0.009   37.000    0.000    0.319    0.775
   .chrnc_w1_rvrsd    2.448    0.084   28.973    0.000    2.448    0.803
   .srh_item1_w2     -0.024    0.054   -0.451    0.652   -0.024   -0.045
   .srh_item2_w2      0.326    0.016   20.130    0.000    0.326    0.758
   .chrnc_w2_rvrsd    2.221    0.146   15.234    0.000    2.221    0.796
    SRHW1             0.501    0.038   13.165    0.000    1.000    1.000
    SRHW2             0.558    0.063    8.812    0.000    1.000    1.000


# Comparing fit of the models

anova(fitSRHw1and2, fitSRHw1and2_corr_duplicates)

Scaled Chi-Squared Difference Test (method = "satorra.bentler.2001")

lavaan NOTE:
    The "Chisq" column contains standard test statistics, not the
    robust test that should be reported per model. A robust difference
    test is a function of two standard (not robust) statistics.
 
                             Df   AIC   BIC    Chisq Chisq diff Df diff
fitSRHw1and2_corr_duplicates  5 26894 27026   7.4124                   
fitSRHw1and2                  8 27277 27392 397.1206     335.25       3
                             Pr(>Chisq)    
fitSRHw1and2_corr_duplicates               
fitSRHw1and2                  < 2.2e-16 ***
---
Signif. codes:  0 '***' 0.001 '**' 0.01 '*' 0.05 '.' 0.1 ' ' 1
```

- Back to the Table of Contents

### Full Measurement Model

```
# Full measurement model

modelmsm <- "SRHW1 =~ srh_item1_w1 + srh_item2_w1 + chronic_w1_reversed
SRHW2 =~ srh_item1_w2 + srh_item2_w2 + chronic_w2_reversed
RS_Eng_W1 =~ rs_identity_w1 + prayer_w1 + attendance_w1 + commitment_w1 +
    rcope_ben_total_w1 + rcope_sup_total_w1
RS_Eng_W2 =~ rs_identity_w2 + prayer_w2 + attendance_w2 + commitment_w2 +
    rcope_ben_total_w2 + rcope_sup_total_w2
rs_identity_w1~~rs_identity_w2
srh_item1_w1~~srh_item1_w2
srh_item2_w1~~srh_item2_w2
chronic_w1_reversed~~chronic_w2_reversed
rs_identity_w1~~rs_identity_w2
prayer_w1~~prayer_w2
attendance_w1~~attendance_w2
commitment_w1~~commitment_w2
rcope_ben_total_w1~~rcope_ben_total_w2
rcope_sup_total_w1~~rcope_sup_total_w2"

# Moving from CFA to SEM
# Fitting model and running summary statistics
fitmsm <- cfa(data = which_comes_first_data, model = modelmsm,
              estimator = "MLR", missing = "ML")
summary(fitmsm, fit.measures = TRUE, standardized = TRUE)
lavaan 0.6.17 ended normally after 100 iterations

  Estimator                                         ML
  Optimization method                           NLMINB
  Number of model parameters                        69

  Number of observations                          3010
  Number of missing patterns                        51

Model Test User Model:
                                              Standard      Scaled
  Test Statistic                               726.086     684.993
  Degrees of freedom                               120         120
  P-value (Chi-square)                           0.000       0.000
  Scaling correction factor                                  1.060
    Yuan-Bentler correction (Mplus variant)                       

Model Test Baseline Model:

  Test statistic                             12671.421   11199.710
  Degrees of freedom                               153         153
  P-value                                        0.000       0.000
  Scaling correction factor                                  1.131

User Model versus Baseline Model:

  Comparative Fit Index (CFI)                    0.952       0.949
  Tucker-Lewis Index (TLI)                       0.938       0.935
                                                                  
  Robust Comparative Fit Index (CFI)                         0.963
  Robust Tucker-Lewis Index (TLI)                            0.953

Loglikelihood and Information Criteria:

  Loglikelihood user model (H0)             -46679.856  -46679.856
  Scaling correction factor                                  1.135
      for the MLR correction                                      
  Loglikelihood unrestricted model (H1)     -46316.813  -46316.813
  Scaling correction factor                                  1.087
      for the MLR correction                                      
                                                                  
  Akaike (AIC)                               93497.712   93497.712
  Bayesian (BIC)                             93912.381   93912.381
  Sample-size adjusted Bayesian (SABIC)      93693.141   93693.141

Root Mean Square Error of Approximation:

  RMSEA                                          0.041       0.040
  90 Percent confidence interval - lower         0.038       0.037
  90 Percent confidence interval - upper         0.044       0.042
  P-value H_0: RMSEA <= 0.050                    1.000       1.000
  P-value H_0: RMSEA >= 0.080                    0.000       0.000
                                                                  
  Robust RMSEA                                               0.057
  90 Percent confidence interval - lower                     0.050
  90 Percent confidence interval - upper                     0.064
  P-value H_0: Robust RMSEA <= 0.050                         0.054
  P-value H_0: Robust RMSEA >= 0.080                         0.000

Standardized Root Mean Square Residual:

  SRMR                                           0.053       0.053

Parameter Estimates:

  Standard errors                             Sandwich
  Information bread                           Observed
  Observed information based on                Hessian

Latent Variables:
                   Estimate  Std.Err  z-value  P(>|z|)   Std.lv  Std.all
  SRHW1 =~                                                              
    srh_item1_w1      1.000                               0.703    0.973
    srh_item2_w1      0.437    0.035   12.507    0.000    0.307    0.478
    chrnc_w1_rvrsd    1.113    0.094   11.803    0.000    0.782    0.448
  SRHW2 =~                                                              
    srh_item1_w2      1.000                               0.742    1.014
    srh_item2_w2      0.439    0.049    9.024    0.000    0.325    0.496
    chrnc_w2_rvrsd    1.025    0.118    8.683    0.000    0.760    0.455
  RS_Eng_W1 =~                                                          
    rs_identity_w1    1.000                               0.446    0.687
    prayer_w1         4.482    0.106   42.205    0.000    2.000    0.822
    attendance_w1     3.794    0.107   35.429    0.000    1.693    0.604
    commitment_w1     1.709    0.056   30.607    0.000    0.763    0.668
    rcop_bn_ttl_w1    3.182    0.113   28.206    0.000    1.420    0.701
    rcop_sp_ttl_w1    4.163    0.121   34.293    0.000    1.858    0.874
  RS_Eng_W2 =~                                                          
    rs_identity_w2    1.000                               0.498    0.750
    prayer_w2         4.118    0.164   25.098    0.000    2.050    0.833
    attendance_w2     3.678    0.180   20.467    0.000    1.830    0.616
    commitment_w2     1.658    0.092   18.062    0.000    0.825    0.706
    rcop_bn_ttl_w2    3.039    0.152   20.029    0.000    1.513    0.715
    rcop_sp_ttl_w2    3.912    0.149   26.339    0.000    1.947    0.900

Covariances:
                         Estimate  Std.Err  z-value  P(>|z|)   Std.lv  Std.all
 .rs_identity_w1 ~~                                                           
   .rs_identity_w2          0.089    0.011    7.824    0.000    0.089    0.430
 .srh_item1_w1 ~~                                                             
   .srh_item1_w2           -0.094    0.032   -2.965    0.003   -0.094   -4.488
 .srh_item2_w1 ~~                                                             
   .srh_item2_w2            0.155    0.013   11.562    0.000    0.155    0.482
 .chronic_w1_reversed ~~                                                      
   .chrnc_w2_rvrsd          1.509    0.104   14.532    0.000    1.509    0.649
 .prayer_w1 ~~                                                                
   .prayer_w2               0.866    0.129    6.687    0.000    0.866    0.459
 .attendance_w1 ~~                                                            
   .attendance_w2           3.269    0.243   13.479    0.000    3.269    0.624
 .commitment_w1 ~~                                                            
   .commitment_w2           0.130    0.039    3.350    0.001    0.130    0.184
 .rcope_ben_total_w1 ~~                                                       
   .rcop_bn_ttl_w2          0.406    0.119    3.396    0.001    0.406    0.190
 .rcope_sup_total_w1 ~~                                                       
   .rcop_sp_ttl_w2          0.110    0.095    1.160    0.246    0.110    0.113
  SRHW1 ~~                                                                    
    SRHW2                   0.397    0.037   10.829    0.000    0.762    0.762
    RS_Eng_W1              -0.013    0.006   -2.094    0.036   -0.042   -0.042
    RS_Eng_W2               0.005    0.010    0.472    0.637    0.013    0.013
  SRHW2 ~~                                                                    
    RS_Eng_W1               0.006    0.011    0.516    0.606    0.018    0.018
    RS_Eng_W2               0.016    0.013    1.240    0.215    0.043    0.043
  RS_Eng_W1 ~~                                                                
    RS_Eng_W2               0.201    0.011   18.475    0.000    0.905    0.905

Intercepts:
                   Estimate  Std.Err  z-value  P(>|z|)   Std.lv  Std.all
   .srh_item1_w1      1.940    0.013  147.258    0.000    1.940    2.685
   .srh_item2_w1      1.360    0.012  115.902    0.000    1.360    2.117
   .chrnc_w1_rvrsd    8.404    0.032  263.575    0.000    8.404    4.813
   .srh_item1_w2      1.922    0.026   74.248    0.000    1.922    2.629
   .srh_item2_w2      1.358    0.024   55.490    0.000    1.358    2.069
   .chrnc_w2_rvrsd    8.113    0.057  142.998    0.000    8.113    4.855
   .rs_identity_w1    1.458    0.012  122.667    0.000    1.458    2.243
   .prayer_w1         4.987    0.045  111.996    0.000    4.987    2.050
   .attendance_w1     3.761    0.051   73.479    0.000    3.761    1.340
   .commitment_w1     2.943    0.021  140.239    0.000    2.943    2.576
   .rcop_bn_ttl_w1    2.823    0.041   69.004    0.000    2.823    1.394
   .rcop_sp_ttl_w1    4.009    0.042   95.032    0.000    4.009    1.887
   .rs_identity_w2    1.420    0.021   67.614    0.000    1.420    2.139
   .prayer_w2         4.731    0.071   67.005    0.000    4.731    1.922
   .attendance_w2     3.474    0.095   36.540    0.000    3.474    1.169
   .commitment_w2     2.907    0.038   75.750    0.000    2.907    2.489
   .rcop_bn_ttl_w2    2.704    0.073   36.806    0.000    2.704    1.278
   .rcop_sp_ttl_w2    3.774    0.065   58.169    0.000    3.774    1.744

Variances:
                   Estimate  Std.Err  z-value  P(>|z|)   Std.lv  Std.all
   .srh_item1_w1      0.028    0.035    0.800    0.424    0.028    0.054
   .srh_item2_w1      0.318    0.009   36.960    0.000    0.318    0.771
   .chrnc_w1_rvrsd    2.437    0.085   28.771    0.000    2.437    0.799
   .srh_item1_w2     -0.015    0.054   -0.289    0.772   -0.015   -0.029
   .srh_item2_w2      0.324    0.016   19.764    0.000    0.324    0.754
   .chrnc_w2_rvrsd    2.214    0.145   15.280    0.000    2.214    0.793
   .rs_identity_w1    0.223    0.007   31.054    0.000    0.223    0.529
   .prayer_w1         1.915    0.091   21.019    0.000    1.915    0.324
   .attendance_w1     5.005    0.124   40.330    0.000    5.005    0.636
   .commitment_w1     0.723    0.026   27.442    0.000    0.723    0.554
   .rcop_bn_ttl_w1    2.082    0.077   27.064    0.000    2.082    0.508
   .rcop_sp_ttl_w1    1.064    0.071   14.902    0.000    1.064    0.236
   .rs_identity_w2    0.193    0.012   16.193    0.000    0.193    0.438
   .prayer_w2         1.860    0.175   10.652    0.000    1.860    0.307
   .attendance_w2     5.478    0.296   18.483    0.000    5.478    0.621
   .commitment_w2     0.683    0.058   11.800    0.000    0.683    0.501
   .rcop_bn_ttl_w2    2.188    0.146   14.967    0.000    2.188    0.489
   .rcop_sp_ttl_w2    0.890    0.092    9.652    0.000    0.890    0.190
    SRHW1             0.494    0.038   13.107    0.000    1.000    1.000
    SRHW2             0.550    0.063    8.710    0.000    1.000    1.000
    RS_Eng_W1         0.199    0.010   19.571    0.000    1.000    1.000
    RS_Eng_W2         0.248    0.018   13.416    0.000    1.000    1.000

# Looking at Residuals
lavResiduals(fitmsm)
$type
[1] "cor.bentler"

$cov
                    sr_1_1 sr_2_1 chr_1_ sr_1_2 sr_2_2 chr_2_ rs_d_1 pryr_1
srh_item1_w1         0.000                                                 
srh_item2_w1         0.000 -0.003                                          
chronic_w1_reversed  0.001 -0.003  0.004                                   
srh_item1_w2         0.006 -0.039  0.047  0.008                            
srh_item2_w2        -0.032 -0.027 -0.002 -0.012 -0.016                     
chronic_w2_reversed  0.028  0.003  0.021  0.028  0.005  0.017              
rs_identity_w1       0.023  0.073 -0.108 -0.020  0.063 -0.121 -0.002       
prayer_w1           -0.016  0.030 -0.124 -0.026  0.056 -0.104  0.034  0.002
attendance_w1        0.070  0.097 -0.046  0.024  0.104 -0.043  0.081  0.007
commitment_w1        0.048  0.091 -0.081 -0.008  0.082 -0.111  0.031  0.010
rcope_ben_total_w1  -0.025 -0.010 -0.026  0.019  0.009  0.050 -0.096 -0.071
rcope_sup_total_w1  -0.024  0.007 -0.112 -0.010  0.051 -0.079 -0.067 -0.012
rs_identity_w2       0.021  0.063 -0.092 -0.032  0.059 -0.097 -0.003  0.013
prayer_w2            0.007  0.070 -0.138 -0.001  0.059 -0.104  0.056  0.007
attendance_w2        0.042  0.055 -0.135 -0.016  0.115 -0.100  0.069 -0.008
commitment_w2        0.076  0.113 -0.080  0.028  0.112 -0.084  0.080  0.016
rcope_ben_total_w2  -0.019 -0.006 -0.070 -0.046  0.039 -0.032 -0.137 -0.040
rcope_sup_total_w2  -0.038  0.018 -0.124 -0.016  0.081 -0.077 -0.015  0.001
                    attn_1 cmmt_1 rcp_b__1 rcp_s__1 rs_d_2 pryr_2 attn_2 cmmt_2
srh_item1_w1                                                                   
srh_item2_w1                                                                   
chronic_w1_reversed                                                            
srh_item1_w2                                                                   
srh_item2_w2                                                                   
chronic_w2_reversed                                                            
rs_identity_w1                                                                 
prayer_w1                                                                      
attendance_w1        0.000                                                     
commitment_w1        0.011  0.001                                              
rcope_ben_total_w1  -0.041 -0.056   -0.022                                     
rcope_sup_total_w1  -0.052 -0.018    0.069   -0.017                            
rs_identity_w2       0.077 -0.002   -0.110   -0.064  0.009                     
prayer_w2            0.033  0.016   -0.076    0.008  0.036 -0.001              
attendance_w2        0.017  0.026   -0.061   -0.037  0.081  0.017  0.025       
commitment_w2        0.077  0.016   -0.047   -0.018  0.026  0.020  0.041  0.004
rcope_ben_total_w2   0.026 -0.053   -0.054   -0.045 -0.081 -0.054  0.016 -0.037
rcope_sup_total_w2   0.010  0.002   -0.046   -0.017 -0.004 -0.021 -0.003 -0.034
                    rcp_b__2 rcp_s__2
srh_item1_w1                         
srh_item2_w1                         
chronic_w1_reversed                  
srh_item1_w2                         
srh_item2_w2                         
chronic_w2_reversed                  
rs_identity_w1                       
prayer_w1                            
attendance_w1                        
commitment_w1                        
rcope_ben_total_w1                   
rcope_sup_total_w1                   
rs_identity_w2                       
prayer_w2                            
attendance_w2                        
commitment_w2                        
rcope_ben_total_w2    -0.024         
rcope_sup_total_w2     0.032   -0.014

$mean
       srh_item1_w1        srh_item2_w1 chronic_w1_reversed        srh_item1_w2 
              0.000               0.000               0.000               0.007 
       srh_item2_w2 chronic_w2_reversed      rs_identity_w1           prayer_w1 
              0.007               0.005               0.000               0.000 
      attendance_w1       commitment_w1  rcope_ben_total_w1  rcope_sup_total_w1 
              0.000               0.000               0.005              -0.002 
     rs_identity_w2           prayer_w2       attendance_w2       commitment_w2 
             -0.016              -0.010              -0.007              -0.033 
 rcope_ben_total_w2  rcope_sup_total_w2 
              0.011               0.006 

$cov.z
                    sr_1_1 sr_2_1 chr_1_ sr_1_2 sr_2_2 chr_2_ rs_d_1 pryr_1
srh_item1_w1        -0.026                                                 
srh_item2_w1         0.045 -0.353                                          
chronic_w1_reversed  0.177 -0.405  0.594                                   
srh_item1_w2         0.558 -1.798  1.601  0.766                            
srh_item2_w2        -1.519 -2.117 -0.070 -0.840 -1.102                     
chronic_w2_reversed  1.515  0.100  1.253  1.342  0.159  0.740              
rs_identity_w1       1.807  4.329 -6.372 -0.744  1.844 -3.848 -0.234       
prayer_w1           -1.714  1.883 -7.858 -1.397  1.836 -3.664  4.067  0.436
attendance_w1        4.669  5.487 -2.552  0.839  2.959 -1.368  6.780  0.868
commitment_w1        3.521  5.298 -4.942 -0.311  2.653 -3.713  2.983  1.329
rcope_ben_total_w1  -1.640 -0.512 -1.387  0.690  0.245  1.593 -8.884 -9.212
rcope_sup_total_w1  -2.496  0.430 -6.561 -0.541  1.614 -2.633 -9.263 -2.043
rs_identity_w2       0.896  2.046 -3.033 -1.210  1.752 -2.745 -0.234  0.693
prayer_w2            0.325  2.506 -5.093 -0.027  1.852 -3.196  2.454  0.614
attendance_w2        1.402  1.923 -3.870 -0.524  3.233 -2.782  3.027 -0.455
commitment_w2        2.390  3.276 -2.121  0.922  3.044 -2.169  2.903  0.670
rcope_ben_total_w2  -0.636 -0.181 -1.913 -1.615  1.077 -0.876 -4.754 -1.980
rcope_sup_total_w2  -1.919  0.636 -4.461 -0.845  2.523 -2.436 -0.642  0.036
                    attn_1 cmmt_1 rcp_b__1 rcp_s__1 rs_d_2 pryr_2 attn_2 cmmt_2
srh_item1_w1                                                                   
srh_item2_w1                                                                   
chronic_w1_reversed                                                            
srh_item1_w2                                                                   
srh_item2_w2                                                                   
chronic_w2_reversed                                                            
rs_identity_w1                                                                 
prayer_w1                                                                      
attendance_w1       -0.056                                                     
commitment_w1        1.061  0.155                                              
rcope_ben_total_w1  -3.364 -4.857   -1.567                                     
rcope_sup_total_w1  -7.345 -2.222    6.443   -2.463                            
rs_identity_w2       3.191 -0.101   -4.384   -3.280  0.538                     
prayer_w2            1.444  0.833   -3.902    0.489  2.133 -0.072              
attendance_w2        1.167  1.369   -2.509   -1.891  3.522  0.935  1.284       
commitment_w2        3.006  1.040   -1.774   -0.782  1.250  1.087  1.800  0.205
rcope_ben_total_w2   0.899 -2.052   -3.123   -2.021 -3.736 -3.107  0.644 -1.704
rcope_sup_total_w2   0.447  0.089   -2.129   -1.342 -0.277 -1.688 -0.218 -2.379
                    rcp_b__2 rcp_s__2
srh_item1_w1                         
srh_item2_w1                         
chronic_w1_reversed                  
srh_item1_w2                         
srh_item2_w2                         
chronic_w2_reversed                  
rs_identity_w1                       
prayer_w1                            
attendance_w1                        
commitment_w1                        
rcope_ben_total_w1                   
rcope_sup_total_w1                   
rs_identity_w2                       
prayer_w2                            
attendance_w2                        
commitment_w2                        
rcope_ben_total_w2    -1.176         
rcope_sup_total_w2     1.817   -1.075

$mean.z
       srh_item1_w1        srh_item2_w1 chronic_w1_reversed        srh_item1_w2 
              0.000               0.000               0.000               0.805 
       srh_item2_w2 chronic_w2_reversed      rs_identity_w1           prayer_w1 
              0.728               0.531               3.124               0.000 
      attendance_w1       commitment_w1  rcope_ben_total_w1  rcope_sup_total_w1 
             -3.911              -4.011               3.202              -1.925 
     rs_identity_w2           prayer_w2       attendance_w2       commitment_w2 
             -1.957              -1.122              -0.914              -2.832 
 rcope_ben_total_w2  rcope_sup_total_w2 
              0.994               0.818 

$summary
                           cov    mean  total
srmr                     0.055   0.010  0.053
srmr.se                  0.003   0.002  0.003
srmr.exactfit.z         12.129   2.116 12.103
srmr.exactfit.pvalue     0.000   0.017  0.000
usrmr                    0.051   0.008  0.048
usrmr.se                 0.005   0.003  0.004
usrmr.ci.lower           0.043   0.003  0.041
usrmr.ci.upper           0.058   0.013  0.056
usrmr.closefit.h0.value  0.050   0.050  0.050
usrmr.closefit.z         0.137 -14.310 -0.402
usrmr.closefit.pvalue    0.446   1.000  0.656

# Looking at Modification Indices
modindices(fitmsm, sort. = TRUE)
                    lhs op                 rhs      mi    epc sepc.lv sepc.all
1                 SRHW1 =~        srh_item1_w1 426.955 -0.823  -0.578   -0.800
93                SRHW2 =~        srh_item1_w1 343.114 -0.864  -0.641   -0.887
250  rcope_ben_total_w1 ~~  rcope_sup_total_w1 318.583  0.851   0.851    0.572
132        srh_item1_w1 ~~        srh_item2_w1  98.375  0.136   0.136    0.241
133        srh_item1_w1 ~~ chronic_w1_reversed  72.030  0.306   0.306    0.196
4                 SRHW2 =~        srh_item1_w2  70.521 -0.670  -0.497   -0.680
220      rs_identity_w1 ~~  rcope_sup_total_w1  68.267 -0.122  -0.122   -0.250
94                SRHW2 =~        srh_item2_w1  62.086  0.356   0.264    0.411
228           prayer_w1 ~~  rcope_ben_total_w1  61.252 -0.423  -0.423   -0.212
217      rs_identity_w1 ~~       attendance_w1  56.293  0.151   0.151    0.143
110           RS_Eng_W1 =~ chronic_w1_reversed  47.813 -0.493  -0.220   -0.126
122           RS_Eng_W2 =~ chronic_w1_reversed  46.765 -0.465  -0.231   -0.133
237       attendance_w1 ~~  rcope_sup_total_w1  41.853 -0.408  -0.408   -0.177
219      rs_identity_w1 ~~  rcope_ben_total_w1  41.638 -0.105  -0.105   -0.154
275  rcope_ben_total_w2 ~~  rcope_sup_total_w2  39.142  0.502   0.502    0.360
95                SRHW2 =~ chronic_w1_reversed  37.822  0.684   0.507    0.290
216      rs_identity_w1 ~~           prayer_w1  27.884  0.083   0.083    0.127
78                SRHW1 =~        srh_item1_w2  26.685 -0.466  -0.327   -0.448
83                SRHW1 =~       attendance_w1  19.838  0.352   0.247    0.088
243       commitment_w1 ~~  rcope_ben_total_w1  17.637 -0.125  -0.125   -0.102
153        srh_item2_w1 ~~       attendance_w1  16.597  0.095   0.095    0.075
98                SRHW2 =~       attendance_w1  15.554  0.348   0.258    0.092
154        srh_item2_w1 ~~       commitment_w1  15.030  0.037   0.037    0.078
177        srh_item1_w2 ~~        srh_item2_w2  14.549  0.101   0.101    0.177
77            RS_Eng_W2 ~1                      12.947  0.781   1.569    1.569
121           RS_Eng_W2 =~        srh_item2_w1  12.464  0.092   0.046    0.071
109           RS_Eng_W1 =~        srh_item2_w1  11.611  0.092   0.041    0.064
148        srh_item2_w1 ~~ chronic_w1_reversed  11.488 -0.099  -0.099   -0.112
218      rs_identity_w1 ~~       commitment_w1  11.414  0.028   0.028    0.070
130           RS_Eng_W2 =~  rcope_ben_total_w1  11.381 -1.491  -0.742   -0.367
86                SRHW1 =~  rcope_sup_total_w1  11.182 -0.179  -0.126   -0.059
224      rs_identity_w1 ~~  rcope_ben_total_w2  10.731 -0.094  -0.094   -0.134
76            RS_Eng_W1 ~1                      10.040 -0.625  -1.401   -1.401
151        srh_item2_w1 ~~      rs_identity_w1   9.997  0.017   0.017    0.063
208 chronic_w2_reversed ~~  rcope_ben_total_w1   9.989  0.268   0.268    0.125
101               SRHW2 =~  rcope_sup_total_w1   9.012 -0.184  -0.136   -0.064
269           prayer_w2 ~~  rcope_sup_total_w2   8.133 -0.220  -0.220   -0.171
178        srh_item1_w2 ~~ chronic_w2_reversed   7.962  0.170   0.170    0.115
169 chronic_w1_reversed ~~  rcope_ben_total_w1   7.931  0.142   0.142    0.063
82                SRHW1 =~           prayer_w1   7.373 -0.156  -0.109   -0.045
118           RS_Eng_W1 =~  rcope_ben_total_w2   7.318 -1.271  -0.567   -0.268
274       commitment_w2 ~~  rcope_sup_total_w2   7.304 -0.119  -0.119   -0.153
156        srh_item2_w1 ~~  rcope_sup_total_w1   7.203 -0.042  -0.042   -0.073
84                SRHW1 =~       commitment_w1   7.111  0.086   0.061    0.053
165 chronic_w1_reversed ~~      rs_identity_w1   6.552 -0.035  -0.035   -0.048
97                SRHW2 =~           prayer_w1   6.487 -0.165  -0.122   -0.050
223      rs_identity_w1 ~~       commitment_w2   6.464  0.039   0.039    0.101
124           RS_Eng_W2 =~        srh_item2_w2   5.948  0.116   0.058    0.088
128           RS_Eng_W2 =~       attendance_w1   5.783  1.237   0.615    0.219
155        srh_item2_w1 ~~  rcope_ben_total_w1   5.772 -0.046  -0.046   -0.057
158        srh_item2_w1 ~~           prayer_w2   5.452  0.067   0.067    0.087
166 chronic_w1_reversed ~~           prayer_w1   5.380 -0.101  -0.101   -0.047
268           prayer_w2 ~~  rcope_ben_total_w2   5.368 -0.204  -0.204   -0.101
80                SRHW1 =~ chronic_w2_reversed   5.340  0.322   0.226    0.135
112           RS_Eng_W1 =~        srh_item2_w2   5.097  0.121   0.054    0.082
257  rcope_sup_total_w1 ~~           prayer_w2   4.969  0.185   0.185    0.131
207 chronic_w2_reversed ~~       commitment_w1   4.950 -0.095  -0.095   -0.075
236       attendance_w1 ~~  rcope_ben_total_w1   4.830 -0.158  -0.158   -0.049
115           RS_Eng_W1 =~           prayer_w2   4.565  1.036   0.462    0.188
200        srh_item2_w2 ~~       attendance_w2   4.563  0.087   0.087    0.066
260  rcope_sup_total_w1 ~~  rcope_ben_total_w2   4.425 -0.189  -0.189   -0.124
99                SRHW2 =~       commitment_w1   4.328  0.077   0.057    0.050
114           RS_Eng_W1 =~      rs_identity_w2   4.000 -0.268  -0.120   -0.180
90                SRHW1 =~       commitment_w2   3.183  0.116   0.081    0.070
162        srh_item2_w1 ~~  rcope_sup_total_w2   3.179 -0.045  -0.045   -0.084
240       attendance_w1 ~~       commitment_w2   3.105  0.110   0.110    0.060
199        srh_item2_w2 ~~           prayer_w2   2.952 -0.050  -0.050   -0.064
173 chronic_w1_reversed ~~       attendance_w2   2.781 -0.162  -0.162   -0.044
244       commitment_w1 ~~  rcope_sup_total_w1   2.645 -0.043  -0.043   -0.050
117           RS_Eng_W1 =~       commitment_w2   2.633  0.417   0.186    0.159
159        srh_item2_w1 ~~       attendance_w2   2.385 -0.062  -0.062   -0.047
196        srh_item2_w2 ~~  rcope_ben_total_w1   2.317 -0.057  -0.057   -0.069
131           RS_Eng_W2 =~  rcope_sup_total_w1   2.314 -0.603  -0.300   -0.141
267           prayer_w2 ~~       commitment_w2   2.302  0.073   0.073    0.064
262      rs_identity_w2 ~~       attendance_w2   2.300  0.050   0.050    0.048
203        srh_item2_w2 ~~  rcope_sup_total_w2   2.256  0.038   0.038    0.071
234           prayer_w1 ~~  rcope_sup_total_w2   2.246  0.107   0.107    0.082
229           prayer_w1 ~~  rcope_sup_total_w1   2.138 -0.080  -0.080   -0.056
227           prayer_w1 ~~       commitment_w1   2.128  0.041   0.041    0.035
256  rcope_sup_total_w1 ~~      rs_identity_w2   2.084 -0.036  -0.036   -0.079
255  rcope_ben_total_w1 ~~  rcope_sup_total_w2   2.031 -0.119  -0.119   -0.087
160        srh_item2_w1 ~~       commitment_w2   2.002  0.025   0.025    0.055
105               SRHW2 =~       commitment_w2   1.806  0.082   0.061    0.052
79                SRHW1 =~        srh_item2_w2   1.605  0.079   0.056    0.085
251  rcope_ben_total_w1 ~~      rs_identity_w2   1.573 -0.037  -0.037   -0.059
75                SRHW2 ~1                       1.564  0.090   0.121    0.121
170 chronic_w1_reversed ~~  rcope_sup_total_w1   1.511 -0.051  -0.051   -0.032
221      rs_identity_w1 ~~           prayer_w2   1.510  0.031   0.031    0.048
140        srh_item1_w1 ~~  rcope_ben_total_w1   1.508 -0.046  -0.046   -0.032
264      rs_identity_w2 ~~  rcope_ben_total_w2   1.462 -0.033  -0.033   -0.050
261      rs_identity_w2 ~~           prayer_w2   1.398  0.029   0.029    0.048
204 chronic_w2_reversed ~~      rs_identity_w1   1.330 -0.025  -0.025   -0.036
193        srh_item2_w2 ~~           prayer_w1   1.323  0.034   0.034    0.044
129           RS_Eng_W2 =~       commitment_w1   1.305  0.260   0.129    0.113
226           prayer_w1 ~~       attendance_w1   1.289  0.076   0.076    0.025
108           RS_Eng_W1 =~        srh_item1_w1   1.186  0.059   0.027    0.037
139        srh_item1_w1 ~~       commitment_w1   1.157  0.020   0.020    0.024
89                SRHW1 =~       attendance_w2   1.121 -0.155  -0.109   -0.037
92                SRHW1 =~  rcope_sup_total_w2   1.088 -0.096  -0.067   -0.031
113           RS_Eng_W1 =~ chronic_w2_reversed   1.077 -0.128  -0.057   -0.034
172 chronic_w1_reversed ~~           prayer_w2   1.053 -0.071  -0.071   -0.033
247       commitment_w1 ~~       attendance_w2   1.040  0.069   0.069    0.035
213 chronic_w2_reversed ~~       commitment_w2   1.029 -0.042  -0.042   -0.034
161        srh_item2_w1 ~~  rcope_ben_total_w2   1.011 -0.034  -0.034   -0.040
241       attendance_w1 ~~  rcope_ben_total_w2   1.006  0.116   0.116    0.035
253  rcope_ben_total_w1 ~~       attendance_w2   0.929 -0.129  -0.129   -0.038
138        srh_item1_w1 ~~       attendance_w1   0.916  0.044   0.044    0.020
215 chronic_w2_reversed ~~  rcope_sup_total_w2   0.882  0.054   0.054    0.038
144        srh_item1_w1 ~~       attendance_w2   0.817  0.079   0.079    0.034
249       commitment_w1 ~~  rcope_sup_total_w2   0.815  0.039   0.039    0.048
167 chronic_w1_reversed ~~       attendance_w1   0.802  0.054   0.054    0.016
248       commitment_w1 ~~  rcope_ben_total_w2   0.769 -0.049  -0.049   -0.039
189        srh_item1_w2 ~~  rcope_ben_total_w2   0.767 -0.063  -0.063   -0.043
125           RS_Eng_W2 =~ chronic_w2_reversed   0.726 -0.093  -0.047   -0.028
145        srh_item1_w1 ~~       commitment_w2   0.713  0.033   0.033    0.039
205 chronic_w2_reversed ~~           prayer_w1   0.689 -0.056  -0.056   -0.027
231           prayer_w1 ~~       attendance_w2   0.668 -0.089  -0.089   -0.027
272       attendance_w2 ~~  rcope_sup_total_w2   0.651 -0.077  -0.077   -0.035
186        srh_item1_w2 ~~           prayer_w2   0.646  0.050   0.050    0.037
147        srh_item1_w1 ~~  rcope_sup_total_w2   0.639 -0.043  -0.043   -0.046
233           prayer_w1 ~~  rcope_ben_total_w2   0.610  0.069   0.069    0.034
225      rs_identity_w1 ~~  rcope_sup_total_w2   0.608 -0.017  -0.017   -0.039
81                SRHW1 =~      rs_identity_w1   0.550  0.013   0.009    0.014
152        srh_item2_w1 ~~           prayer_w1   0.533 -0.012  -0.012   -0.016
168 chronic_w1_reversed ~~       commitment_w1   0.524 -0.018  -0.018   -0.014
119           RS_Eng_W1 =~  rcope_sup_total_w2   0.502  0.285   0.127    0.059
171 chronic_w1_reversed ~~      rs_identity_w2   0.501  0.015   0.015    0.022
141        srh_item1_w1 ~~  rcope_sup_total_w1   0.477 -0.021  -0.021   -0.021
194        srh_item2_w2 ~~       attendance_w1   0.463 -0.027  -0.027   -0.021
88                SRHW1 =~           prayer_w2   0.459  0.071   0.050    0.020
13            RS_Eng_W2 =~      rs_identity_w2   0.426  1.737   0.865    1.303
176 chronic_w1_reversed ~~  rcope_sup_total_w2   0.415 -0.039  -0.039   -0.026
214 chronic_w2_reversed ~~  rcope_ben_total_w2   0.379  0.047   0.047    0.021
265      rs_identity_w2 ~~  rcope_sup_total_w2   0.375  0.014   0.014    0.033
127           RS_Eng_W2 =~           prayer_w1   0.364  0.259   0.129    0.053
273       commitment_w2 ~~  rcope_ben_total_w2   0.356 -0.033  -0.033   -0.027
136        srh_item1_w1 ~~      rs_identity_w1   0.350  0.006   0.006    0.013
210 chronic_w2_reversed ~~      rs_identity_w2   0.350 -0.012  -0.012   -0.019
137        srh_item1_w1 ~~           prayer_w1   0.348 -0.019  -0.019   -0.014
245       commitment_w1 ~~      rs_identity_w2   0.323 -0.008  -0.008   -0.023
157        srh_item2_w1 ~~      rs_identity_w2   0.279  0.005   0.005    0.019
198        srh_item2_w2 ~~      rs_identity_w2   0.272 -0.005  -0.005   -0.019
96                SRHW2 =~      rs_identity_w1   0.268  0.010   0.008    0.012
201        srh_item2_w2 ~~       commitment_w2   0.260  0.009   0.009    0.020
180        srh_item1_w2 ~~           prayer_w1   0.248 -0.032  -0.032   -0.023
195        srh_item2_w2 ~~       commitment_w1   0.246  0.009   0.009    0.019
187        srh_item1_w2 ~~       attendance_w2   0.245 -0.043  -0.043   -0.019
143        srh_item1_w1 ~~           prayer_w2   0.244  0.030   0.030    0.022
235       attendance_w1 ~~       commitment_w1   0.238  0.018   0.018    0.009
87                SRHW1 =~      rs_identity_w2   0.232  0.016   0.011    0.017
212 chronic_w2_reversed ~~       attendance_w2   0.214 -0.043  -0.043   -0.012
106               SRHW2 =~  rcope_ben_total_w2   0.211 -0.052  -0.038   -0.018
252  rcope_ben_total_w1 ~~           prayer_w2   0.204 -0.043  -0.043   -0.022
174 chronic_w1_reversed ~~       commitment_w2   0.200  0.019   0.019    0.015
104               SRHW2 =~       attendance_w2   0.193 -0.060  -0.045   -0.015
191        srh_item2_w2 ~~ chronic_w2_reversed   0.182 -0.013  -0.013   -0.015
91                SRHW1 =~  rcope_ben_total_w2   0.171 -0.050  -0.035   -0.016
266           prayer_w2 ~~       attendance_w2   0.165  0.043   0.043    0.014
185        srh_item1_w2 ~~      rs_identity_w2   0.160 -0.008  -0.008   -0.018
164 chronic_w1_reversed ~~        srh_item2_w2   0.152 -0.011  -0.011   -0.012
123           RS_Eng_W2 =~        srh_item1_w2   0.150 -0.040  -0.020   -0.027
116           RS_Eng_W1 =~       attendance_w2   0.150 -0.233  -0.104   -0.035
259  rcope_sup_total_w1 ~~       commitment_w2   0.147 -0.019  -0.019   -0.022
85                SRHW1 =~  rcope_ben_total_w1   0.137 -0.024  -0.017   -0.008
270       attendance_w2 ~~       commitment_w2   0.136 -0.024  -0.024   -0.013
149        srh_item2_w1 ~~        srh_item1_w2   0.127 -0.009  -0.009   -0.016
209 chronic_w2_reversed ~~  rcope_sup_total_w1   0.115  0.023   0.023    0.015
74                SRHW1 ~1                       0.111 -0.012  -0.017   -0.017
103               SRHW2 =~           prayer_w2   0.107  0.032   0.024    0.010
242       attendance_w1 ~~  rcope_sup_total_w2   0.104 -0.029  -0.029   -0.014
183        srh_item1_w2 ~~  rcope_ben_total_w1   0.101  0.025   0.025    0.018
111           RS_Eng_W1 =~        srh_item1_w2   0.081 -0.033  -0.015   -0.020
142        srh_item1_w1 ~~      rs_identity_w2   0.078  0.005   0.005    0.012
188        srh_item1_w2 ~~       commitment_w2   0.071  0.010   0.010    0.013
163 chronic_w1_reversed ~~        srh_item1_w2   0.070  0.016   0.016    0.010
181        srh_item1_w2 ~~       attendance_w1   0.068  0.022   0.022    0.010
238       attendance_w1 ~~      rs_identity_w2   0.061  0.008   0.008    0.008
102               SRHW2 =~      rs_identity_w2   0.059 -0.007  -0.006   -0.008
192        srh_item2_w2 ~~      rs_identity_w1   0.056  0.002   0.002    0.008
197        srh_item2_w2 ~~  rcope_sup_total_w1   0.055 -0.007  -0.007   -0.012
232           prayer_w1 ~~       commitment_w2   0.052 -0.011  -0.011   -0.010
206 chronic_w2_reversed ~~       attendance_w1   0.049  0.020   0.020    0.006
202        srh_item2_w2 ~~  rcope_ben_total_w2   0.049  0.007   0.007    0.009
184        srh_item1_w2 ~~  rcope_sup_total_w1   0.047  0.014   0.014    0.014
120           RS_Eng_W2 =~        srh_item1_w1   0.041  0.011   0.005    0.007
179        srh_item1_w2 ~~      rs_identity_w1   0.030  0.004   0.004    0.008
126           RS_Eng_W2 =~      rs_identity_w1   0.030  0.022   0.011    0.016
134        srh_item1_w1 ~~        srh_item2_w2   0.030 -0.004  -0.004   -0.008
254  rcope_ben_total_w1 ~~       commitment_w2   0.029 -0.010  -0.010   -0.009
239       attendance_w1 ~~           prayer_w2   0.029 -0.017  -0.017   -0.006
246       commitment_w1 ~~           prayer_w2   0.027 -0.008  -0.008   -0.007
263      rs_identity_w2 ~~       commitment_w2   0.018 -0.002  -0.002   -0.005
100               SRHW2 =~  rcope_ben_total_w1   0.014  0.009   0.006    0.003
107               SRHW2 =~  rcope_sup_total_w2   0.012  0.010   0.007    0.003
190        srh_item1_w2 ~~  rcope_sup_total_w2   0.009  0.005   0.005    0.005
182        srh_item1_w2 ~~       commitment_w1   0.008 -0.004  -0.004   -0.004
211 chronic_w2_reversed ~~           prayer_w2   0.007  0.005   0.005    0.003
271       attendance_w2 ~~  rcope_ben_total_w2   0.006  0.010   0.010    0.003
146        srh_item1_w1 ~~  rcope_ben_total_w2   0.005  0.005   0.005    0.003
230           prayer_w1 ~~      rs_identity_w2   0.004  0.002   0.002    0.003
150        srh_item2_w1 ~~ chronic_w2_reversed   0.004  0.002   0.002    0.002
222      rs_identity_w1 ~~       attendance_w2   0.004  0.002   0.002    0.002
258  rcope_sup_total_w1 ~~       attendance_w2   0.001  0.004   0.004    0.002
135        srh_item1_w1 ~~ chronic_w2_reversed   0.001 -0.002  -0.002   -0.001
7             RS_Eng_W1 =~      rs_identity_w1   0.000  0.044   0.020    0.030
175 chronic_w1_reversed ~~  rcope_ben_total_w2   0.000  0.002   0.002    0.001
    sepc.nox
1     -0.800
93    -0.887
250    0.572
132    0.241
133    0.196
4     -0.680
220   -0.250
94     0.411
228   -0.212
217    0.143
110   -0.126
122   -0.133
237   -0.177
219   -0.154
275    0.360
95     0.290
216    0.127
78    -0.448
83     0.088
243   -0.102
153    0.075
98     0.092
154    0.078
177    0.177
77     1.569
121    0.071
109    0.064
148   -0.112
218    0.070
130   -0.367
86    -0.059
224   -0.134
76    -1.401
151    0.063
208    0.125
101   -0.064
269   -0.171
178    0.115
169    0.063
82    -0.045
118   -0.268
274   -0.153
156   -0.073
84     0.053
165   -0.048
97    -0.050
223    0.101
124    0.088
128    0.219
155   -0.057
158    0.087
166   -0.047
268   -0.101
80     0.135
112    0.082
257    0.131
207   -0.075
236   -0.049
115    0.188
200    0.066
260   -0.124
99     0.050
114   -0.180
90     0.070
162   -0.084
240    0.060
199   -0.064
173   -0.044
244   -0.050
117    0.159
159   -0.047
196   -0.069
131   -0.141
267    0.064
262    0.048
203    0.071
234    0.082
229   -0.056
227    0.035
256   -0.079
255   -0.087
160    0.055
105    0.052
79     0.085
251   -0.059
75     0.121
170   -0.032
221    0.048
140   -0.032
264   -0.050
261    0.048
204   -0.036
193    0.044
129    0.113
226    0.025
108    0.037
139    0.024
89    -0.037
92    -0.031
113   -0.034
172   -0.033
247    0.035
213   -0.034
161   -0.040
241    0.035
253   -0.038
138    0.020
215    0.038
144    0.034
249    0.048
167    0.016
248   -0.039
189   -0.043
125   -0.028
145    0.039
205   -0.027
231   -0.027
272   -0.035
186    0.037
147   -0.046
233    0.034
225   -0.039
81     0.014
152   -0.016
168   -0.014
119    0.059
171    0.022
141   -0.021
194   -0.021
88     0.020
13     1.303
176   -0.026
214    0.021
265    0.033
127    0.053
273   -0.027
136    0.013
210   -0.019
137   -0.014
245   -0.023
157    0.019
198   -0.019
96     0.012
201    0.020
180   -0.023
195    0.019
187   -0.019
143    0.022
235    0.009
87     0.017
212   -0.012
106   -0.018
252   -0.022
174    0.015
104   -0.015
191   -0.015
91    -0.016
266    0.014
185   -0.018
164   -0.012
123   -0.027
116   -0.035
259   -0.022
85    -0.008
270   -0.013
149   -0.016
209    0.015
74    -0.017
103    0.010
242   -0.014
183    0.018
111   -0.020
142    0.012
188    0.013
163    0.010
181    0.010
238    0.008
102   -0.008
192    0.008
197   -0.012
232   -0.010
206    0.006
202    0.009
184    0.014
120    0.007
179    0.008
126    0.016
134   -0.008
254   -0.009
239   -0.006
246   -0.007
263   -0.005
100    0.003
107    0.003
190    0.005
182   -0.004
211    0.003
271    0.003
146    0.003
230    0.003
150    0.002
222    0.002
258    0.002
135   -0.001
7      0.030
175    0.001

# Removing Correlated Error Between SRH Item 1 at Waves 1 and 2
modelmsm_2 <- "SRHW1 =~ srh_item1_w1 + srh_item2_w1 + chronic_w1_reversed
SRHW2 =~ srh_item1_w2 + srh_item2_w2 + chronic_w2_reversed
RS_Eng_W1 =~ rs_identity_w1 + prayer_w1 + attendance_w1 + commitment_w1 +
    rcope_ben_total_w1 + rcope_sup_total_w1
RS_Eng_W2 =~ rs_identity_w2 + prayer_w2 + attendance_w2 + commitment_w2 +
    rcope_ben_total_w2 + rcope_sup_total_w2
rs_identity_w1~~rs_identity_w2
srh_item2_w1~~srh_item2_w2
chronic_w1_reversed~~chronic_w2_reversed
rs_identity_w1~~rs_identity_w2
prayer_w1~~prayer_w2
attendance_w1~~attendance_w2
commitment_w1~~commitment_w2
rcope_ben_total_w1~~rcope_ben_total_w2
rcope_sup_total_w1~~rcope_sup_total_w2"

# Fitting model and running summary statistics
fitmsm_2 <- cfa(data = which_comes_first_data, model = modelmsm_2,
  estimator = "MLR", missing = "ML")
summary(fitmsm_2, fit.measures = TRUE, standardized = TRUE)
lavaan 0.6.17 ended normally after 97 iterations

  Estimator                                         ML
  Optimization method                           NLMINB
  Number of model parameters                        68

  Number of observations                          3010
  Number of missing patterns                        51

Model Test User Model:
                                              Standard      Scaled
  Test Statistic                               739.133     696.970
  Degrees of freedom                               121         121
  P-value (Chi-square)                           0.000       0.000
  Scaling correction factor                                  1.060
    Yuan-Bentler correction (Mplus variant)                       

Model Test Baseline Model:

  Test statistic                             12671.421   11199.710
  Degrees of freedom                               153         153
  P-value                                        0.000       0.000
  Scaling correction factor                                  1.131

User Model versus Baseline Model:

  Comparative Fit Index (CFI)                    0.951       0.948
  Tucker-Lewis Index (TLI)                       0.938       0.934
                                                                  
  Robust Comparative Fit Index (CFI)                         0.961
  Robust Tucker-Lewis Index (TLI)                            0.951

Loglikelihood and Information Criteria:

  Loglikelihood user model (H0)             -46686.379  -46686.379
  Scaling correction factor                                  1.135
      for the MLR correction                                      
  Loglikelihood unrestricted model (H1)     -46316.813  -46316.813
  Scaling correction factor                                  1.087
      for the MLR correction                                      
                                                                  
  Akaike (AIC)                               93508.759   93508.759
  Bayesian (BIC)                             93917.418   93917.418
  Sample-size adjusted Bayesian (SABIC)      93701.355   93701.355

Root Mean Square Error of Approximation:

  RMSEA                                          0.041       0.040
  90 Percent confidence interval - lower         0.038       0.037
  90 Percent confidence interval - upper         0.044       0.043
  P-value H_0: RMSEA <= 0.050                    1.000       1.000
  P-value H_0: RMSEA >= 0.080                    0.000       0.000
                                                                  
  Robust RMSEA                                               0.058
  90 Percent confidence interval - lower                     0.051
  90 Percent confidence interval - upper                     0.064
  P-value H_0: Robust RMSEA <= 0.050                         0.035
  P-value H_0: Robust RMSEA >= 0.080                         0.000

Standardized Root Mean Square Residual:

  SRMR                                           0.053       0.053

Parameter Estimates:

  Standard errors                             Sandwich
  Information bread                           Observed
  Observed information based on                Hessian

Latent Variables:
                   Estimate  Std.Err  z-value  P(>|z|)   Std.lv  Std.all
  SRHW1 =~                                                              
    srh_item1_w1      1.000                               0.656    0.909
    srh_item2_w1      0.499    0.028   18.027    0.000    0.327    0.510
    chrnc_w1_rvrsd    1.259    0.081   15.596    0.000    0.826    0.474
  SRHW2 =~                                                              
    srh_item1_w2      1.000                               0.649    0.887
    srh_item2_w2      0.549    0.044   12.568    0.000    0.356    0.547
    chrnc_w2_rvrsd    1.261    0.114   11.109    0.000    0.818    0.494
  RS_Eng_W1 =~                                                          
    rs_identity_w1    1.000                               0.446    0.687
    prayer_w1         4.482    0.106   42.202    0.000    2.000    0.822
    attendance_w1     3.794    0.107   35.425    0.000    1.693    0.603
    commitment_w1     1.709    0.056   30.607    0.000    0.763    0.668
    rcop_bn_ttl_w1    3.182    0.113   28.210    0.000    1.420    0.701
    rcop_sp_ttl_w1    4.163    0.121   34.298    0.000    1.858    0.874
  RS_Eng_W2 =~                                                          
    rs_identity_w2    1.000                               0.498    0.750
    prayer_w2         4.118    0.164   25.099    0.000    2.049    0.832
    attendance_w2     3.678    0.180   20.463    0.000    1.830    0.616
    commitment_w2     1.658    0.092   18.058    0.000    0.825    0.706
    rcop_bn_ttl_w2    3.039    0.152   20.030    0.000    1.512    0.715
    rcop_sp_ttl_w2    3.912    0.149   26.328    0.000    1.947    0.900

Covariances:
                         Estimate  Std.Err  z-value  P(>|z|)   Std.lv  Std.all
 .rs_identity_w1 ~~                                                           
   .rs_identity_w2          0.089    0.011    7.826    0.000    0.089    0.430
 .srh_item2_w1 ~~                                                             
   .srh_item2_w2            0.138    0.012   11.370    0.000    0.138    0.458
 .chronic_w1_reversed ~~                                                      
   .chrnc_w2_rvrsd          1.412    0.098   14.441    0.000    1.412    0.638
 .prayer_w1 ~~                                                                
   .prayer_w2               0.866    0.130    6.688    0.000    0.866    0.459
 .attendance_w1 ~~                                                            
   .attendance_w2           3.269    0.243   13.475    0.000    3.269    0.624
 .commitment_w1 ~~                                                            
   .commitment_w2           0.130    0.039    3.355    0.001    0.130    0.185
 .rcope_ben_total_w1 ~~                                                       
   .rcop_bn_ttl_w2          0.405    0.120    3.392    0.001    0.405    0.190
 .rcope_sup_total_w1 ~~                                                       
   .rcop_sp_ttl_w2          0.110    0.095    1.159    0.246    0.110    0.113
  SRHW1 ~~                                                                    
    SRHW2                   0.312    0.020   15.307    0.000    0.732    0.732
    RS_Eng_W1              -0.014    0.006   -2.315    0.021   -0.049   -0.049
    RS_Eng_W2               0.004    0.010    0.387    0.698    0.012    0.012
  SRHW2 ~~                                                                    
    RS_Eng_W1               0.005    0.011    0.447    0.655    0.017    0.017
    RS_Eng_W2               0.016    0.013    1.238    0.216    0.049    0.049
  RS_Eng_W1 ~~                                                                
    RS_Eng_W2               0.201    0.011   18.467    0.000    0.905    0.905

Intercepts:
                   Estimate  Std.Err  z-value  P(>|z|)   Std.lv  Std.all
   .srh_item1_w1      1.940    0.013  147.258    0.000    1.940    2.685
   .srh_item2_w1      1.360    0.012  115.900    0.000    1.360    2.118
   .chrnc_w1_rvrsd    8.404    0.032  263.575    0.000    8.404    4.818
   .srh_item1_w2      1.920    0.026   73.834    0.000    1.920    2.625
   .srh_item2_w2      1.358    0.024   55.512    0.000    1.358    2.085
   .chrnc_w2_rvrsd    8.116    0.057  142.939    0.000    8.116    4.899
   .rs_identity_w1    1.458    0.012  122.667    0.000    1.458    2.243
   .prayer_w1         4.987    0.045  111.997    0.000    4.987    2.050
   .attendance_w1     3.761    0.051   73.479    0.000    3.761    1.340
   .commitment_w1     2.943    0.021  140.240    0.000    2.943    2.576
   .rcop_bn_ttl_w1    2.823    0.041   69.001    0.000    2.823    1.394
   .rcop_sp_ttl_w1    4.009    0.042   95.026    0.000    4.009    1.886
   .rs_identity_w2    1.420    0.021   67.547    0.000    1.420    2.139
   .prayer_w2         4.730    0.071   66.855    0.000    4.730    1.922
   .attendance_w2     3.474    0.095   36.528    0.000    3.474    1.169
   .commitment_w2     2.907    0.038   75.701    0.000    2.907    2.489
   .rcop_bn_ttl_w2    2.703    0.073   36.781    0.000    2.703    1.278
   .rcop_sp_ttl_w2    3.773    0.065   58.091    0.000    3.773    1.745

Variances:
                   Estimate  Std.Err  z-value  P(>|z|)   Std.lv  Std.all
   .srh_item1_w1      0.091    0.021    4.332    0.000    0.091    0.174
   .srh_item2_w1      0.305    0.007   40.718    0.000    0.305    0.740
   .chrnc_w1_rvrsd    2.361    0.079   29.694    0.000    2.361    0.776
   .srh_item1_w2      0.114    0.026    4.457    0.000    0.114    0.214
   .srh_item2_w2      0.298    0.015   19.890    0.000    0.298    0.701
   .chrnc_w2_rvrsd    2.076    0.135   15.361    0.000    2.076    0.756
   .rs_identity_w1    0.223    0.007   31.055    0.000    0.223    0.529
   .prayer_w1         1.915    0.091   21.014    0.000    1.915    0.324
   .attendance_w1     5.006    0.124   40.334    0.000    5.006    0.636
   .commitment_w1     0.723    0.026   27.443    0.000    0.723    0.554
   .rcop_bn_ttl_w1    2.082    0.077   27.066    0.000    2.082    0.508
   .rcop_sp_ttl_w1    1.063    0.071   14.897    0.000    1.063    0.235
   .rs_identity_w2    0.193    0.012   16.191    0.000    0.193    0.438
   .prayer_w2         1.861    0.175   10.660    0.000    1.861    0.307
   .attendance_w2     5.478    0.296   18.481    0.000    5.478    0.621
   .commitment_w2     0.683    0.058   11.805    0.000    0.683    0.501
   .rcop_bn_ttl_w2    2.188    0.146   14.969    0.000    2.188    0.489
   .rcop_sp_ttl_w2    0.889    0.092    9.640    0.000    0.889    0.190
    SRHW1             0.431    0.025   17.319    0.000    1.000    1.000
    SRHW2             0.421    0.040   10.526    0.000    1.000    1.000
    RS_Eng_W1         0.199    0.010   19.570    0.000    1.000    1.000
    RS_Eng_W2         0.248    0.018   13.412    0.000    1.000    1.000

# The Covariances section above displays correlations (under std.all)
# The Latent Variables section above displays factor loadings (under std.all)
# The above is the final Measurement Model retained for structural analyses
```

- Back to the Table of Contents

## Longitudinal Measurement Invariance

Establish the longitudinal measurement invariance of the constructs
at four nested levels (configural, metric, scalar, and error). Begin
with just self-rated health, then assess religious and spiritual
engagement, followed by a combined model. In cases of non-invariance at
a particular level, allow for partial measurement invariance by relaxing
constraints one-at-a-time and identifying the model that a) fits best to
the data and b) makes theoretical sense.

### Self-Rated Health Longitudinal Invariance

```
# Reparameterizing - from constraining first loading to the standardized
# latent variable approach.

# Configural Model

modelSRHconfig <- "SRHW1 =~ NA*srh_item1_w1 + srh_item2_w1 + chronic_w1_reversed
SRHW2 =~ NA*srh_item1_w2 + srh_item2_w2 + chronic_w2_reversed
SRHW1~0
SRHW2~0
SRHW1~~1*SRHW1
SRHW2~~1*SRHW2
srh_item2_w1~~srh_item2_w2
chronic_w1_reversed~~chronic_w2_reversed"

# Fitting model and running summary statistics

fitSRHconfig <- cfa(
  data = which_comes_first_data, model = modelSRHconfig,
  estimator = "MLR", missing = "ML", std.lv = T,
  meanstructure = T
)
summary(fitSRHconfig, fit.measures = TRUE, standardized = TRUE)
lavaan 0.6.17 ended normally after 42 iterations

  Estimator                                         ML
  Optimization method                           NLMINB
  Number of model parameters                        21

  Number of observations                          3010
  Number of missing patterns                         9

Model Test User Model:
                                              Standard      Scaled
  Test Statistic                                21.594      18.839
  Degrees of freedom                                 6           6
  P-value (Chi-square)                           0.001       0.004
  Scaling correction factor                                  1.146
    Yuan-Bentler correction (Mplus variant)                       

Model Test Baseline Model:

  Test statistic                              2321.008    2010.100
  Degrees of freedom                                15          15
  P-value                                        0.000       0.000
  Scaling correction factor                                  1.155

User Model versus Baseline Model:

  Comparative Fit Index (CFI)                    0.993       0.994
  Tucker-Lewis Index (TLI)                       0.983       0.984
                                                                  
  Robust Comparative Fit Index (CFI)                         0.991
  Robust Tucker-Lewis Index (TLI)                            0.977

Loglikelihood and Information Criteria:

  Loglikelihood user model (H0)             -13431.923  -13431.923
  Scaling correction factor                                  1.101
      for the MLR correction                                      
  Loglikelihood unrestricted model (H1)     -13421.126  -13421.126
  Scaling correction factor                                  1.111
      for the MLR correction                                      
                                                                  
  Akaike (AIC)                               26905.846   26905.846
  Bayesian (BIC)                             27032.050   27032.050
  Sample-size adjusted Bayesian (SABIC)      26965.325   26965.325

Root Mean Square Error of Approximation:

  RMSEA                                          0.029       0.027
  90 Percent confidence interval - lower         0.017       0.014
  90 Percent confidence interval - upper         0.043       0.040
  P-value H_0: RMSEA <= 0.050                    0.994       0.999
  P-value H_0: RMSEA >= 0.080                    0.000       0.000
                                                                  
  Robust RMSEA                                               0.057
  90 Percent confidence interval - lower                     0.025
  90 Percent confidence interval - upper                     0.091
  P-value H_0: Robust RMSEA <= 0.050                         0.310
  P-value H_0: Robust RMSEA >= 0.080                         0.149

Standardized Root Mean Square Residual:

  SRMR                                           0.027       0.027

Parameter Estimates:

  Standard errors                             Sandwich
  Information bread                           Observed
  Observed information based on                Hessian

Latent Variables:
                   Estimate  Std.Err  z-value  P(>|z|)   Std.lv  Std.all
  SRHW1 =~                                                              
    srh_item1_w1      0.658    0.019   34.968    0.000    0.658    0.911
    srh_item2_w1      0.327    0.014   23.380    0.000    0.327    0.509
    chrnc_w1_rvrsd    0.823    0.042   19.701    0.000    0.823    0.472
  SRHW2 =~                                                              
    srh_item1_w2      0.649    0.031   21.198    0.000    0.649    0.888
    srh_item2_w2      0.355    0.025   14.019    0.000    0.355    0.545
    chrnc_w2_rvrsd    0.816    0.065   12.580    0.000    0.816    0.493

Covariances:
                         Estimate  Std.Err  z-value  P(>|z|)   Std.lv  Std.all
 .srh_item2_w1 ~~                                                             
   .srh_item2_w2            0.138    0.012   11.424    0.000    0.138    0.459
 .chronic_w1_reversed ~~                                                      
   .chrnc_w2_rvrsd          1.411    0.097   14.469    0.000    1.411    0.637
  SRHW1 ~~                                                                    
    SRHW2                   0.729    0.040   18.359    0.000    0.729    0.729

Intercepts:
                   Estimate  Std.Err  z-value  P(>|z|)   Std.lv  Std.all
    SRHW1             0.000                               0.000    0.000
    SRHW2             0.000                               0.000    0.000
   .srh_item1_w1      1.940    0.013  147.261    0.000    1.940    2.685
   .srh_item2_w1      1.360    0.012  115.898    0.000    1.360    2.118
   .chrnc_w1_rvrsd    8.404    0.032  263.575    0.000    8.404    4.817
   .srh_item1_w2      1.919    0.026   73.491    0.000    1.919    2.624
   .srh_item2_w2      1.358    0.025   55.257    0.000    1.358    2.085
   .chrnc_w2_rvrsd    8.114    0.057  142.769    0.000    8.114    4.905

Variances:
                   Estimate  Std.Err  z-value  P(>|z|)   Std.lv  Std.all
    SRHW1             1.000                               1.000    1.000
    SRHW2             1.000                               1.000    1.000
   .srh_item1_w1      0.089    0.021    4.250    0.000    0.089    0.170
   .srh_item2_w1      0.305    0.008   40.473    0.000    0.305    0.740
   .chrnc_w1_rvrsd    2.367    0.079   29.913    0.000    2.367    0.778
   .srh_item1_w2      0.113    0.026    4.442    0.000    0.113    0.212
   .srh_item2_w2      0.298    0.015   20.146    0.000    0.298    0.703
   .chrnc_w2_rvrsd    2.071    0.135   15.315    0.000    2.071    0.757


# Metric Model

modelSRHmetric <- "SRHW1 =~ NA*L1*srh_item1_w1 + L2*srh_item2_w1 +
                                L3*chronic_w1_reversed
SRHW2 =~ NA*L1*srh_item1_w2 + L2*srh_item2_w2 + L3*chronic_w2_reversed
SRHW1~0
SRHW2~0
SRHW1~~1*SRHW1
SRHW2~~1*SRHW2
srh_item2_w1~~srh_item2_w2
chronic_w1_reversed~~chronic_w2_reversed"

# Fitting model and running summary statistics

fitSRHmetric <- cfa(
  data = which_comes_first_data, model = modelSRHmetric,
  estimator = "MLR", missing = "ML", std.lv = T,
  meanstructure = T
)
summary(fitSRHmetric, fit.measures = TRUE, standardized = TRUE)
lavaan 0.6.17 ended normally after 36 iterations

  Estimator                                         ML
  Optimization method                           NLMINB
  Number of model parameters                        21
  Number of equality constraints                     3

  Number of observations                          3010
  Number of missing patterns                         9

Model Test User Model:
                                              Standard      Scaled
  Test Statistic                                22.923      20.362
  Degrees of freedom                                 9           9
  P-value (Chi-square)                           0.006       0.016
  Scaling correction factor                                  1.126
    Yuan-Bentler correction (Mplus variant)                       

Model Test Baseline Model:

  Test statistic                              2321.008    2010.100
  Degrees of freedom                                15          15
  P-value                                        0.000       0.000
  Scaling correction factor                                  1.155

User Model versus Baseline Model:

  Comparative Fit Index (CFI)                    0.994       0.994
  Tucker-Lewis Index (TLI)                       0.990       0.991
                                                                  
  Robust Comparative Fit Index (CFI)                         0.993
  Robust Tucker-Lewis Index (TLI)                            0.989

Loglikelihood and Information Criteria:

  Loglikelihood user model (H0)             -13432.587  -13432.587
  Scaling correction factor                                  0.946
      for the MLR correction                                      
  Loglikelihood unrestricted model (H1)     -13421.126  -13421.126
  Scaling correction factor                                  1.111
      for the MLR correction                                      
                                                                  
  Akaike (AIC)                               26901.175   26901.175
  Bayesian (BIC)                             27009.349   27009.349
  Sample-size adjusted Bayesian (SABIC)      26952.156   26952.156

Root Mean Square Error of Approximation:

  RMSEA                                          0.023       0.020
  90 Percent confidence interval - lower         0.011       0.009
  90 Percent confidence interval - upper         0.034       0.032
  P-value H_0: RMSEA <= 0.050                    1.000       1.000
  P-value H_0: RMSEA >= 0.080                    0.000       0.000
                                                                  
  Robust RMSEA                                               0.040
  90 Percent confidence interval - lower                     0.001
  90 Percent confidence interval - upper                     0.070
  P-value H_0: Robust RMSEA <= 0.050                         0.670
  P-value H_0: Robust RMSEA >= 0.080                         0.010

Standardized Root Mean Square Residual:

  SRMR                                           0.026       0.026

Parameter Estimates:

  Standard errors                             Sandwich
  Information bread                           Observed
  Observed information based on                Hessian

Latent Variables:
                   Estimate  Std.Err  z-value  P(>|z|)   Std.lv  Std.all
  SRHW1 =~                                                              
    srh_tm1_1 (L1)    0.654    0.015   43.198    0.000    0.654    0.906
    srh_tm2_1 (L2)    0.332    0.013   25.505    0.000    0.332    0.516
    chrnc_w1_ (L3)    0.824    0.039   21.163    0.000    0.824    0.473
  SRHW2 =~                                                              
    srh_tm1_2 (L1)    0.654    0.015   43.198    0.000    0.654    0.895
    srh_tm2_2 (L2)    0.332    0.013   25.505    0.000    0.332    0.517
    chrnc_w2_ (L3)    0.824    0.039   21.163    0.000    0.824    0.497

Covariances:
                         Estimate  Std.Err  z-value  P(>|z|)   Std.lv  Std.all
 .srh_item2_w1 ~~                                                             
   .srh_item2_w2            0.139    0.012   11.455    0.000    0.139    0.459
 .chronic_w1_reversed ~~                                                      
   .chrnc_w2_rvrsd          1.408    0.098   14.429    0.000    1.408    0.637
  SRHW1 ~~                                                                    
    SRHW2                   0.728    0.040   18.090    0.000    0.728    0.728

Intercepts:
                   Estimate  Std.Err  z-value  P(>|z|)   Std.lv  Std.all
    SRHW1             0.000                               0.000    0.000
    SRHW2             0.000                               0.000    0.000
   .srh_item1_w1      1.940    0.013  147.259    0.000    1.940    2.685
   .srh_item2_w1      1.360    0.012  115.901    0.000    1.360    2.113
   .chrnc_w1_rvrsd    8.404    0.032  263.575    0.000    8.404    4.820
   .srh_item1_w2      1.919    0.025   75.696    0.000    1.919    2.625
   .srh_item2_w2      1.362    0.023   58.624    0.000    1.362    2.119
   .chrnc_w2_rvrsd    8.114    0.054  149.861    0.000    8.114    4.893

Variances:
                   Estimate  Std.Err  z-value  P(>|z|)   Std.lv  Std.all
    SRHW1             1.000                               1.000    1.000
    SRHW2             1.000                               1.000    1.000
   .srh_item1_w1      0.094    0.016    5.737    0.000    0.094    0.180
   .srh_item2_w1      0.304    0.007   41.593    0.000    0.304    0.734
   .chrnc_w1_rvrsd    2.361    0.078   30.458    0.000    2.361    0.777
   .srh_item1_w2      0.106    0.018    5.805    0.000    0.106    0.199
   .srh_item2_w2      0.303    0.014   22.114    0.000    0.303    0.733
   .chrnc_w2_rvrsd    2.071    0.136   15.209    0.000    2.071    0.753

# Comparing fit of the models

anova(fitSRHmetric, fitSRHconfig)

Scaled Chi-Squared Difference Test (method = "satorra.bentler.2001")

lavaan NOTE:
    The "Chisq" column contains standard test statistics, not the
    robust test that should be reported per model. A robust difference
    test is a function of two standard (not robust) statistics.
 
             Df   AIC   BIC  Chisq Chisq diff Df diff Pr(>Chisq)
fitSRHconfig  6 26906 27032 21.594                              
fitSRHmetric  9 26901 27009 22.923     1.2244       3     0.7471


# Scalar model

modelSRHscalar <- "SRHW1 =~ NA*L1*srh_item1_w1 + L2*srh_item2_w1 +
                                L3*chronic_w1_reversed
SRHW2 =~ NA*L1*srh_item1_w2 + L2*srh_item2_w2 + L3*chronic_w2_reversed
SRHW1~0
SRHW2~NA*0
SRHW1~~1*SRHW1
SRHW2~~1*SRHW2
srh_item2_w1~~srh_item2_w2
chronic_w1_reversed~~chronic_w2_reversed
srh_item1_w1~i1*1
srh_item1_w2~i1*1
srh_item2_w1~i2*1
srh_item2_w2~i2*1
chronic_w1_reversed~i3*1
chronic_w2_reversed~i3*1"

# Fitting model and running summary statistics

fitSRHscalar <- cfa(
  data = which_comes_first_data, model = modelSRHscalar,
  estimator = "MLR", missing = "ML", std.lv = T,
  meanstructure = T
)
summary(fitSRHscalar, fit.measures = TRUE, standardized = TRUE)
lavaan 0.6.17 ended normally after 35 iterations

  Estimator                                         ML
  Optimization method                           NLMINB
  Number of model parameters                        22
  Number of equality constraints                     6

  Number of observations                          3010
  Number of missing patterns                         9

Model Test User Model:
                                              Standard      Scaled
  Test Statistic                                51.595      46.991
  Degrees of freedom                                11          11
  P-value (Chi-square)                           0.000       0.000
  Scaling correction factor                                  1.098
    Yuan-Bentler correction (Mplus variant)                       

Model Test Baseline Model:

  Test statistic                              2321.008    2010.100
  Degrees of freedom                                15          15
  P-value                                        0.000       0.000
  Scaling correction factor                                  1.155

User Model versus Baseline Model:

  Comparative Fit Index (CFI)                    0.982       0.982
  Tucker-Lewis Index (TLI)                       0.976       0.975
                                                                  
  Robust Comparative Fit Index (CFI)                         0.972
  Robust Tucker-Lewis Index (TLI)                            0.962

Loglikelihood and Information Criteria:

  Loglikelihood user model (H0)             -13446.923  -13446.923
  Scaling correction factor                                  0.815
      for the MLR correction                                      
  Loglikelihood unrestricted model (H1)     -13421.126  -13421.126
  Scaling correction factor                                  1.111
      for the MLR correction                                      
                                                                  
  Akaike (AIC)                               26925.847   26925.847
  Bayesian (BIC)                             27022.002   27022.002
  Sample-size adjusted Bayesian (SABIC)      26971.164   26971.164

Root Mean Square Error of Approximation:

  RMSEA                                          0.035       0.033
  90 Percent confidence interval - lower         0.026       0.024
  90 Percent confidence interval - upper         0.045       0.042
  P-value H_0: RMSEA <= 0.050                    0.994       0.999
  P-value H_0: RMSEA >= 0.080                    0.000       0.000
                                                                  
  Robust RMSEA                                               0.075
  90 Percent confidence interval - lower                     0.053
  90 Percent confidence interval - upper                     0.098
  P-value H_0: Robust RMSEA <= 0.050                         0.034
  P-value H_0: Robust RMSEA >= 0.080                         0.373

Standardized Root Mean Square Residual:

  SRMR                                           0.036       0.036

Parameter Estimates:

  Standard errors                             Sandwich
  Information bread                           Observed
  Observed information based on                Hessian

Latent Variables:
                   Estimate  Std.Err  z-value  P(>|z|)   Std.lv  Std.all
  SRHW1 =~                                                              
    srh_tm1_1 (L1)    0.656    0.015   42.979    0.000    0.656    0.909
    srh_tm2_1 (L2)    0.331    0.013   25.367    0.000    0.331    0.515
    chrnc_w1_ (L3)    0.824    0.039   20.950    0.000    0.824    0.472
  SRHW2 =~                                                              
    srh_tm1_2 (L1)    0.656    0.015   42.979    0.000    0.656    0.895
    srh_tm2_2 (L2)    0.331    0.013   25.367    0.000    0.331    0.515
    chrnc_w2_ (L3)    0.824    0.039   20.950    0.000    0.824    0.493

Covariances:
                         Estimate  Std.Err  z-value  P(>|z|)   Std.lv  Std.all
 .srh_item2_w1 ~~                                                             
   .srh_item2_w2            0.139    0.012   11.477    0.000    0.139    0.459
 .chronic_w1_reversed ~~                                                      
   .chrnc_w2_rvrsd          1.395    0.097   14.311    0.000    1.395    0.624
  SRHW1 ~~                                                                    
    SRHW2                   0.727    0.041   17.787    0.000    0.727    0.727

Intercepts:
                   Estimate  Std.Err  z-value  P(>|z|)   Std.lv  Std.all
    SRHW1             0.000                               0.000    0.000
    SRHW2            -0.070    0.036   -1.940    0.052   -0.070   -0.070
   .srh_tm1_1 (i1)    1.943    0.013  147.745    0.000    1.943    2.689
   .srh_tm1_2 (i1)    1.943    0.013  147.745    0.000    1.943    2.650
   .srh_tm2_1 (i2)    1.363    0.011  119.033    0.000    1.363    2.119
   .srh_tm2_2 (i2)    1.363    0.011  119.033    0.000    1.363    2.121
   .chrnc_w1_ (i3)    8.370    0.032  264.257    0.000    8.370    4.798
   .chrnc_w2_ (i3)    8.370    0.032  264.257    0.000    8.370    5.011

Variances:
                   Estimate  Std.Err  z-value  P(>|z|)   Std.lv  Std.all
    SRHW1             1.000                               1.000    1.000
    SRHW2             1.000                               1.000    1.000
   .srh_item1_w1      0.091    0.017    5.481    0.000    0.091    0.174
   .srh_item2_w1      0.304    0.007   41.554    0.000    0.304    0.735
   .chrnc_w1_rvrsd    2.365    0.078   30.404    0.000    2.365    0.777
   .srh_item1_w2      0.107    0.019    5.625    0.000    0.107    0.198
   .srh_item2_w2      0.303    0.014   22.436    0.000    0.303    0.735
   .chrnc_w2_rvrsd    2.112    0.145   14.529    0.000    2.112    0.757

# Comparing fit of the models

anova(fitSRHscalar, fitSRHmetric)

Scaled Chi-Squared Difference Test (method = "satorra.bentler.2001")

lavaan NOTE:
    The "Chisq" column contains standard test statistics, not the
    robust test that should be reported per model. A robust difference
    test is a function of two standard (not robust) statistics.
 
             Df   AIC   BIC  Chisq Chisq diff Df diff Pr(>Chisq)    
fitSRHmetric  9 26901 27009 22.923                                  
fitSRHscalar 11 26926 27022 51.595     29.467       2  3.994e-07 ***
---
Signif. codes:  0 '***' 0.001 '**' 0.01 '*' 0.05 '.' 0.1 ' ' 1

# Looking at fit indices to establish meaningful differences in fit statistics

fitmeasures(fitSRHmetric, fit.measures = c("cfi.robust", "rmsea.robust",
                                           "srmr"))
  cfi.robust rmsea.robust         srmr 
       0.993        0.040        0.026 
fitmeasures(fitSRHscalar, fit.measures = c("cfi.robust", "rmsea.robust",
                                           "srmr"))
  cfi.robust rmsea.robust         srmr 
       0.972        0.075        0.036 


# Look under the summary of the metric model to identify differences (> .2)
# in the Intercept values of the same variable across timepoints.
# Chronic illnesses is identified as potentially different, but we will relax
# constraints one-at-a-time to confirm what the best fitting model is.


# Scalar model with constraint for SRH Item 1 relaxed

modelSRHscalar_SRH1_unconstrained <- "SRHW1 =~ NA*L1*srh_item1_w1 +
                                        L2*srh_item2_w1 + L3*chronic_w1_reversed
SRHW2 =~ NA*L1*srh_item1_w2 + L2*srh_item2_w2 + L3*chronic_w2_reversed
SRHW1~0
SRHW2~NA*0
SRHW1~~1*SRHW1
SRHW2~~1*SRHW2
srh_item2_w1~~srh_item2_w2
chronic_w1_reversed~~chronic_w2_reversed
srh_item2_w1~i2*1
srh_item2_w2~i2*1
chronic_w1_reversed~i3*1
chronic_w2_reversed~i3*1"

# Fitting model and running summary statistics

fitSRHscalar_SRH1_unconstrained <- cfa(
  data = which_comes_first_data,
  model = modelSRHscalar_SRH1_unconstrained,
  estimator = "MLR", missing = "ML", std.lv = T,
  meanstructure = T
)
summary(fitSRHscalar_SRH1_unconstrained, fit.measures = TRUE,
        standardized = TRUE)
lavaan 0.6.17 ended normally after 39 iterations

  Estimator                                         ML
  Optimization method                           NLMINB
  Number of model parameters                        22
  Number of equality constraints                     5

  Number of observations                          3010
  Number of missing patterns                         9

Model Test User Model:
                                              Standard      Scaled
  Test Statistic                                41.028      36.714
  Degrees of freedom                                10          10
  P-value (Chi-square)                           0.000       0.000
  Scaling correction factor                                  1.117
    Yuan-Bentler correction (Mplus variant)                       

Model Test Baseline Model:

  Test statistic                              2321.008    2010.100
  Degrees of freedom                                15          15
  P-value                                        0.000       0.000
  Scaling correction factor                                  1.155

User Model versus Baseline Model:

  Comparative Fit Index (CFI)                    0.987       0.987
  Tucker-Lewis Index (TLI)                       0.980       0.980
                                                                  
  Robust Comparative Fit Index (CFI)                         0.980
  Robust Tucker-Lewis Index (TLI)                            0.970

Loglikelihood and Information Criteria:

  Loglikelihood user model (H0)             -13441.640  -13441.640
  Scaling correction factor                                  0.856
      for the MLR correction                                      
  Loglikelihood unrestricted model (H1)     -13421.126  -13421.126
  Scaling correction factor                                  1.111
      for the MLR correction                                      
                                                                  
  Akaike (AIC)                               26917.280   26917.280
  Bayesian (BIC)                             27019.445   27019.445
  Sample-size adjusted Bayesian (SABIC)      26965.429   26965.429

Root Mean Square Error of Approximation:

  RMSEA                                          0.032       0.030
  90 Percent confidence interval - lower         0.022       0.020
  90 Percent confidence interval - upper         0.043       0.040
  P-value H_0: RMSEA <= 0.050                    0.998       1.000
  P-value H_0: RMSEA >= 0.080                    0.000       0.000
                                                                  
  Robust RMSEA                                               0.066
  90 Percent confidence interval - lower                     0.042
  90 Percent confidence interval - upper                     0.091
  P-value H_0: Robust RMSEA <= 0.050                         0.127
  P-value H_0: Robust RMSEA >= 0.080                         0.189

Standardized Root Mean Square Residual:

  SRMR                                           0.033       0.033

Parameter Estimates:

  Standard errors                             Sandwich
  Information bread                           Observed
  Observed information based on                Hessian

Latent Variables:
                   Estimate  Std.Err  z-value  P(>|z|)   Std.lv  Std.all
  SRHW1 =~                                                              
    srh_tm1_1 (L1)    0.655    0.015   43.067    0.000    0.655    0.906
    srh_tm2_1 (L2)    0.329    0.013   25.031    0.000    0.329    0.512
    chrnc_w1_ (L3)    0.831    0.039   21.398    0.000    0.831    0.476
  SRHW2 =~                                                              
    srh_tm1_2 (L1)    0.655    0.015   43.067    0.000    0.655    0.896
    srh_tm2_2 (L2)    0.329    0.013   25.031    0.000    0.329    0.510
    chrnc_w2_ (L3)    0.831    0.039   21.398    0.000    0.831    0.499

Covariances:
                         Estimate  Std.Err  z-value  P(>|z|)   Std.lv  Std.all
 .srh_item2_w1 ~~                                                             
   .srh_item2_w2            0.139    0.012   11.368    0.000    0.139    0.455
 .chronic_w1_reversed ~~                                                      
   .chrnc_w2_rvrsd          1.401    0.097   14.443    0.000    1.401    0.632
  SRHW1 ~~                                                                    
    SRHW2                   0.729    0.041   17.915    0.000    0.729    0.729

Intercepts:
                   Estimate  Std.Err  z-value  P(>|z|)   Std.lv  Std.all
    SRHW1             0.000                               0.000    0.000
    SRHW2            -0.192    0.050   -3.838    0.000   -0.192   -0.192
   .srh_tm2_1 (i2)    1.368    0.012  118.235    0.000    1.368    2.130
   .srh_tm2_2 (i2)    1.368    0.012  118.235    0.000    1.368    2.122
   .chrnc_w1_ (i3)    8.385    0.032  263.996    0.000    8.385    4.801
   .chrnc_w2_ (i3)    8.385    0.032  263.996    0.000    8.385    5.038
   .srh_tm1_1         1.940    0.013  147.259    0.000    1.940    2.685
   .srh_tm1_2         2.044    0.033   61.771    0.000    2.044    2.795

Variances:
                   Estimate  Std.Err  z-value  P(>|z|)   Std.lv  Std.all
    SRHW1             1.000                               1.000    1.000
    SRHW2             1.000                               1.000    1.000
   .srh_item1_w1      0.093    0.016    5.654    0.000    0.093    0.179
   .srh_item2_w1      0.304    0.007   41.783    0.000    0.304    0.738
   .chrnc_w1_rvrsd    2.360    0.078   30.334    0.000    2.360    0.774
   .srh_item1_w2      0.106    0.019    5.716    0.000    0.106    0.198
   .srh_item2_w2      0.307    0.014   22.249    0.000    0.307    0.739
   .chrnc_w2_rvrsd    2.079    0.139   14.990    0.000    2.079    0.751

# Comparing fit of the models

anova(fitSRHmetric, fitSRHscalar_SRH1_unconstrained)

Scaled Chi-Squared Difference Test (method = "satorra.bentler.2001")

lavaan NOTE:
    The "Chisq" column contains standard test statistics, not the
    robust test that should be reported per model. A robust difference
    test is a function of two standard (not robust) statistics.
 
                                Df   AIC   BIC  Chisq Chisq diff Df diff
fitSRHmetric                     9 26901 27009 22.923                   
fitSRHscalar_SRH1_unconstrained 10 26917 27019 41.028     17.354       1
                                Pr(>Chisq)    
fitSRHmetric                                  
fitSRHscalar_SRH1_unconstrained  3.102e-05 ***
---
Signif. codes:  0 '***' 0.001 '**' 0.01 '*' 0.05 '.' 0.1 ' ' 1
# The difference in fit is still significant, but we also need to look at fit
# indices to establish whether such differences in fit statistics are meaningful

fitmeasures(fitSRHscalar, fit.measures = c("cfi.robust", "rmsea.robust",
                                           "srmr"))
  cfi.robust rmsea.robust         srmr 
       0.972        0.075        0.036 
fitmeasures(fitSRHmetric, fit.measures = c("cfi.robust", "rmsea.robust",
                                           "srmr"))
  cfi.robust rmsea.robust         srmr 
       0.993        0.040        0.026 
fitmeasures(fitSRHscalar_SRH1_unconstrained, fit.measures = c(
  "cfi.robust",
  "rmsea.robust",
  "srmr"
))
  cfi.robust rmsea.robust         srmr 
       0.980        0.066        0.033 
# These are both significant and meaningful drops in model fit.


# Scalar model with constraint for SRH Item 2 relaxed

modelSRHscalar_SRH2_unconstrained <- "SRHW1 =~ NA*L1*srh_item1_w1 +
                                        L2*srh_item2_w1 +
                                        L3*chronic_w1_reversed
SRHW2 =~ NA*L1*srh_item1_w2 + L2*srh_item2_w2 + L3*chronic_w2_reversed
SRHW1~0
SRHW2~NA*0
SRHW1~~1*SRHW1
SRHW2~~1*SRHW2
srh_item2_w1~~srh_item2_w2
chronic_w1_reversed~~chronic_w2_reversed
srh_item1_w1~i1*1
srh_item1_w2~i1*1
chronic_w1_reversed~i3*1
chronic_w2_reversed~i3*1"

# Fitting model and running summary statistics

fitSRHscalar_SRH2_unconstrained <- cfa(
  data = which_comes_first_data,
  model = modelSRHscalar_SRH2_unconstrained,
  estimator = "MLR", missing = "ML",
  std.lv = T, meanstructure = T
)
summary(fitSRHscalar_SRH2_unconstrained, fit.measures = TRUE,
        standardized = TRUE)
lavaan 0.6.17 ended normally after 37 iterations

  Estimator                                         ML
  Optimization method                           NLMINB
  Number of model parameters                        22
  Number of equality constraints                     5

  Number of observations                          3010
  Number of missing patterns                         9

Model Test User Model:
                                              Standard      Scaled
  Test Statistic                                49.984      45.061
  Degrees of freedom                                10          10
  P-value (Chi-square)                           0.000       0.000
  Scaling correction factor                                  1.109
    Yuan-Bentler correction (Mplus variant)                       

Model Test Baseline Model:

  Test statistic                              2321.008    2010.100
  Degrees of freedom                                15          15
  P-value                                        0.000       0.000
  Scaling correction factor                                  1.155

User Model versus Baseline Model:

  Comparative Fit Index (CFI)                    0.983       0.982
  Tucker-Lewis Index (TLI)                       0.974       0.974
                                                                  
  Robust Comparative Fit Index (CFI)                         0.973
  Robust Tucker-Lewis Index (TLI)                            0.959

Loglikelihood and Information Criteria:

  Loglikelihood user model (H0)             -13446.118  -13446.118
  Scaling correction factor                                  0.860
      for the MLR correction                                      
  Loglikelihood unrestricted model (H1)     -13421.126  -13421.126
  Scaling correction factor                                  1.111
      for the MLR correction                                      
                                                                  
  Akaike (AIC)                               26926.236   26926.236
  Bayesian (BIC)                             27028.401   27028.401
  Sample-size adjusted Bayesian (SABIC)      26974.385   26974.385

Root Mean Square Error of Approximation:

  RMSEA                                          0.036       0.034
  90 Percent confidence interval - lower         0.027       0.025
  90 Percent confidence interval - upper         0.047       0.044
  P-value H_0: RMSEA <= 0.050                    0.985       0.996
  P-value H_0: RMSEA >= 0.080                    0.000       0.000
                                                                  
  Robust RMSEA                                               0.077
  90 Percent confidence interval - lower                     0.055
  90 Percent confidence interval - upper                     0.102
  P-value H_0: Robust RMSEA <= 0.050                         0.026
  P-value H_0: Robust RMSEA >= 0.080                         0.461

Standardized Root Mean Square Residual:

  SRMR                                           0.035       0.035

Parameter Estimates:

  Standard errors                             Sandwich
  Information bread                           Observed
  Observed information based on                Hessian

Latent Variables:
                   Estimate  Std.Err  z-value  P(>|z|)   Std.lv  Std.all
  SRHW1 =~                                                              
    srh_tm1_1 (L1)    0.656    0.015   42.798    0.000    0.656    0.908
    srh_tm2_1 (L2)    0.331    0.013   25.386    0.000    0.331    0.515
    chrnc_w1_ (L3)    0.826    0.039   20.968    0.000    0.826    0.473
  SRHW2 =~                                                              
    srh_tm1_2 (L1)    0.656    0.015   42.798    0.000    0.656    0.894
    srh_tm2_2 (L2)    0.331    0.013   25.386    0.000    0.331    0.516
    chrnc_w2_ (L3)    0.826    0.039   20.968    0.000    0.826    0.494

Covariances:
                         Estimate  Std.Err  z-value  P(>|z|)   Std.lv  Std.all
 .srh_item2_w1 ~~                                                             
   .srh_item2_w2            0.139    0.012   11.470    0.000    0.139    0.459
 .chronic_w1_reversed ~~                                                      
   .chrnc_w2_rvrsd          1.396    0.097   14.326    0.000    1.396    0.625
  SRHW1 ~~                                                                    
    SRHW2                   0.729    0.041   17.877    0.000    0.729    0.729

Intercepts:
                   Estimate  Std.Err  z-value  P(>|z|)   Std.lv  Std.all
    SRHW1             0.000                               0.000    0.000
    SRHW2            -0.081    0.037   -2.169    0.030   -0.081   -0.081
   .srh_tm1_1 (i1)    1.944    0.013  147.444    0.000    1.944    2.691
   .srh_tm1_2 (i1)    1.944    0.013  147.444    0.000    1.944    2.648
   .chrnc_w1_ (i3)    8.372    0.032  264.562    0.000    8.372    4.797
   .chrnc_w2_ (i3)    8.372    0.032  264.562    0.000    8.372    5.012
   .srh_tm2_1         1.360    0.012  115.903    0.000    1.360    2.113
   .srh_tm2_2         1.387    0.022   63.014    0.000    1.387    2.160

Variances:
                   Estimate  Std.Err  z-value  P(>|z|)   Std.lv  Std.all
    SRHW1             1.000                               1.000    1.000
    SRHW2             1.000                               1.000    1.000
   .srh_item1_w1      0.092    0.017    5.515    0.000    0.092    0.176
   .srh_item2_w1      0.304    0.007   41.512    0.000    0.304    0.735
   .chrnc_w1_rvrsd    2.364    0.078   30.351    0.000    2.364    0.776
   .srh_item1_w2      0.109    0.019    5.700    0.000    0.109    0.202
   .srh_item2_w2      0.303    0.014   22.176    0.000    0.303    0.734
   .chrnc_w2_rvrsd    2.108    0.145   14.579    0.000    2.108    0.756

# Comparing fit of the models

anova(fitSRHmetric, fitSRHscalar_SRH2_unconstrained)

Scaled Chi-Squared Difference Test (method = "satorra.bentler.2001")

lavaan NOTE:
    The "Chisq" column contains standard test statistics, not the
    robust test that should be reported per model. A robust difference
    test is a function of two standard (not robust) statistics.
 
                                Df   AIC   BIC  Chisq Chisq diff Df diff
fitSRHmetric                     9 26901 27009 22.923                   
fitSRHscalar_SRH2_unconstrained 10 26926 27028 49.984     28.161       1
                                Pr(>Chisq)    
fitSRHmetric                                  
fitSRHscalar_SRH2_unconstrained  1.117e-07 ***
---
Signif. codes:  0 '***' 0.001 '**' 0.01 '*' 0.05 '.' 0.1 ' ' 1
# The difference in fit is still significant, but we also need to look at fit
# indices to establish whether such differences in fit statistics are meaningful

fitmeasures(fitSRHscalar, fit.measures = c("cfi.robust", "rmsea.robust",
                                           "srmr"))
  cfi.robust rmsea.robust         srmr 
       0.972        0.075        0.036 
fitmeasures(fitSRHmetric, fit.measures = c("cfi.robust", "rmsea.robust",
                                           "srmr"))
  cfi.robust rmsea.robust         srmr 
       0.993        0.040        0.026 
fitmeasures(fitSRHscalar_SRH2_unconstrained, fit.measures = c("cfi.robust",
                                                              "rmsea.robust",
                                                              "srmr"))
  cfi.robust rmsea.robust         srmr 
       0.973        0.077        0.035 
# These are both significant and meaningful drops in model fit.


# Scalar model with constraint for chronic illnesses relaxed

modelSRHscalar_illnesses_unconstrained <- "SRHW1 =~ NA*L1*srh_item1_w1 +
                                              L2*srh_item2_w1 +
                                              L3*chronic_w1_reversed
SRHW2 =~ NA*L1*srh_item1_w2 + L2*srh_item2_w2 + L3*chronic_w2_reversed
SRHW1~0
SRHW2~NA*0
SRHW1~~1*SRHW1
SRHW2~~1*SRHW2
srh_item2_w1~~srh_item2_w2
chronic_w1_reversed~~chronic_w2_reversed
srh_item1_w1~i1*1
srh_item1_w2~i1*1
srh_item2_w1~i2*1
srh_item2_w2~i2*1"

# Fitting model and running summary statistics

fitSRHscalar_illnesses_unconstrained <- cfa(data = which_comes_first_data,
                                model = modelSRHscalar_illnesses_unconstrained,
                                estimator = "MLR", missing = "ML", std.lv = T,
                                meanstructure = T)
summary(fitSRHscalar_illnesses_unconstrained, fit.measures = TRUE,
        standardized = TRUE)
lavaan 0.6.17 ended normally after 38 iterations

  Estimator                                         ML
  Optimization method                           NLMINB
  Number of model parameters                        22
  Number of equality constraints                     5

  Number of observations                          3010
  Number of missing patterns                         9

Model Test User Model:
                                              Standard      Scaled
  Test Statistic                                23.258      20.922
  Degrees of freedom                                10          10
  P-value (Chi-square)                           0.010       0.022
  Scaling correction factor                                  1.112
    Yuan-Bentler correction (Mplus variant)                       

Model Test Baseline Model:

  Test statistic                              2321.008    2010.100
  Degrees of freedom                                15          15
  P-value                                        0.000       0.000
  Scaling correction factor                                  1.155

User Model versus Baseline Model:

  Comparative Fit Index (CFI)                    0.994       0.995
  Tucker-Lewis Index (TLI)                       0.991       0.992
                                                                  
  Robust Comparative Fit Index (CFI)                         0.994
  Robust Tucker-Lewis Index (TLI)                            0.991

Loglikelihood and Information Criteria:

  Loglikelihood user model (H0)             -13432.755  -13432.755
  Scaling correction factor                                  0.859
      for the MLR correction                                      
  Loglikelihood unrestricted model (H1)     -13421.126  -13421.126
  Scaling correction factor                                  1.111
      for the MLR correction                                      
                                                                  
  Akaike (AIC)                               26899.510   26899.510
  Bayesian (BIC)                             27001.675   27001.675
  Sample-size adjusted Bayesian (SABIC)      26947.659   26947.659

Root Mean Square Error of Approximation:

  RMSEA                                          0.021       0.019
  90 Percent confidence interval - lower         0.010       0.008
  90 Percent confidence interval - upper         0.032       0.030
  P-value H_0: RMSEA <= 0.050                    1.000       1.000
  P-value H_0: RMSEA >= 0.080                    0.000       0.000
                                                                  
  Robust RMSEA                                               0.036
  90 Percent confidence interval - lower                     0.000
  90 Percent confidence interval - upper                     0.065
  P-value H_0: Robust RMSEA <= 0.050                         0.759
  P-value H_0: Robust RMSEA >= 0.080                         0.004

Standardized Root Mean Square Residual:

  SRMR                                           0.026       0.026

Parameter Estimates:

  Standard errors                             Sandwich
  Information bread                           Observed
  Observed information based on                Hessian

Latent Variables:
                   Estimate  Std.Err  z-value  P(>|z|)   Std.lv  Std.all
  SRHW1 =~                                                              
    srh_tm1_1 (L1)    0.654    0.015   43.251    0.000    0.654    0.905
    srh_tm2_1 (L2)    0.332    0.013   25.531    0.000    0.332    0.516
    chrnc_w1_ (L3)    0.824    0.039   21.194    0.000    0.824    0.473
  SRHW2 =~                                                              
    srh_tm1_2 (L1)    0.654    0.015   43.251    0.000    0.654    0.895
    srh_tm2_2 (L2)    0.332    0.013   25.531    0.000    0.332    0.517
    chrnc_w2_ (L3)    0.824    0.039   21.194    0.000    0.824    0.497

Covariances:
                         Estimate  Std.Err  z-value  P(>|z|)   Std.lv  Std.all
 .srh_item2_w1 ~~                                                             
   .srh_item2_w2            0.139    0.012   11.456    0.000    0.139    0.459
 .chronic_w1_reversed ~~                                                      
   .chrnc_w2_rvrsd          1.408    0.098   14.428    0.000    1.408    0.637
  SRHW1 ~~                                                                    
    SRHW2                   0.729    0.040   18.108    0.000    0.729    0.729

Intercepts:
                   Estimate  Std.Err  z-value  P(>|z|)   Std.lv  Std.all
    SRHW1             0.000                               0.000    0.000
    SRHW2            -0.027    0.037   -0.743    0.457   -0.027   -0.027
   .srh_tm1_1 (i1)    1.939    0.013  147.520    0.000    1.939    2.685
   .srh_tm1_2 (i1)    1.939    0.013  147.520    0.000    1.939    2.654
   .srh_tm2_1 (i2)    1.361    0.011  118.592    0.000    1.361    2.116
   .srh_tm2_2 (i2)    1.361    0.011  118.592    0.000    1.361    2.118
   .chrnc_w1_         8.404    0.032  263.575    0.000    8.404    4.820
   .chrnc_w2_         8.136    0.055  149.015    0.000    8.136    4.906

Variances:
                   Estimate  Std.Err  z-value  P(>|z|)   Std.lv  Std.all
    SRHW1             1.000                               1.000    1.000
    SRHW2             1.000                               1.000    1.000
   .srh_item1_w1      0.094    0.016    5.789    0.000    0.094    0.181
   .srh_item2_w1      0.304    0.007   41.622    0.000    0.304    0.733
   .chrnc_w1_rvrsd    2.360    0.077   30.468    0.000    2.360    0.776
   .srh_item1_w2      0.106    0.018    5.824    0.000    0.106    0.199
   .srh_item2_w2      0.303    0.014   22.193    0.000    0.303    0.733
   .chrnc_w2_rvrsd    2.071    0.136   15.210    0.000    2.071    0.753

# Comparing fit of the models

anova(fitSRHmetric, fitSRHscalar_illnesses_unconstrained)

Scaled Chi-Squared Difference Test (method = "satorra.bentler.2001")

lavaan NOTE:
    The "Chisq" column contains standard test statistics, not the
    robust test that should be reported per model. A robust difference
    test is a function of two standard (not robust) statistics.
 
                                     Df   AIC   BIC  Chisq Chisq diff Df diff
fitSRHmetric                          9 26901 27009 22.923                   
fitSRHscalar_illnesses_unconstrained 10 26900 27002 23.258    0.34055       1
                                     Pr(>Chisq)
fitSRHmetric                                   
fitSRHscalar_illnesses_unconstrained     0.5595
# Difference in fit between metric and scalar models is no longer significant

fitmeasures(fitSRHscalar, fit.measures = c("cfi.robust", "rmsea.robust",
                                           "srmr"))
  cfi.robust rmsea.robust         srmr 
       0.972        0.075        0.036 
fitmeasures(fitSRHmetric, fit.measures = c("cfi.robust", "rmsea.robust",
                                           "srmr"))
  cfi.robust rmsea.robust         srmr 
       0.993        0.040        0.026 
fitmeasures(fitSRHscalar_illnesses_unconstrained, fit.measures = c(
  "cfi.robust",
  "rmsea.robust",
  "srmr"
))
  cfi.robust rmsea.robust         srmr 
       0.994        0.036        0.026 
# The differences are not meaningful either, and some indices have improved.
# This is the partially invariant scalar model we will retain.


# Error model

modelSRHerror <- "SRHW1 =~ NA*L1*srh_item1_w1 + L2*srh_item2_w1 +
                     L3*chronic_w1_reversed
SRHW2 =~ NA*L1*srh_item1_w2 + L2*srh_item2_w2 + L3*chronic_w2_reversed
SRHW1~0
SRHW2~NA*0
SRHW1~~1*SRHW1
SRHW2~~1*SRHW2
srh_item2_w1~~srh_item2_w2
chronic_w1_reversed~~chronic_w2_reversed
srh_item1_w1~i1*1
srh_item1_w2~i1*1
srh_item2_w1~i2*1
srh_item2_w2~i2*1
srh_item1_w1~~e1*srh_item1_w1
srh_item1_w2~~e1*srh_item1_w2
srh_item2_w1~~e2*srh_item2_w1
srh_item2_w2~~e2*srh_item2_w2"

# Fitting model and running summary statistics

fitSRHerror <- cfa(data = which_comes_first_data, model = modelSRHerror,
                   estimator = "MLR", missing = "ML", std.lv = T,
                   meanstructure = T)
summary(fitSRHerror, fit.measures = TRUE, standardized = TRUE)
lavaan 0.6.17 ended normally after 38 iterations

  Estimator                                         ML
  Optimization method                           NLMINB
  Number of model parameters                        22
  Number of equality constraints                     7

  Number of observations                          3010
  Number of missing patterns                         9

Model Test User Model:
                                              Standard      Scaled
  Test Statistic                                23.591      22.359
  Degrees of freedom                                12          12
  P-value (Chi-square)                           0.023       0.034
  Scaling correction factor                                  1.055
    Yuan-Bentler correction (Mplus variant)                       

Model Test Baseline Model:

  Test statistic                              2321.008    2010.100
  Degrees of freedom                                15          15
  P-value                                        0.000       0.000
  Scaling correction factor                                  1.155

User Model versus Baseline Model:

  Comparative Fit Index (CFI)                    0.995       0.995
  Tucker-Lewis Index (TLI)                       0.994       0.994
                                                                  
  Robust Comparative Fit Index (CFI)                         0.995
  Robust Tucker-Lewis Index (TLI)                            0.994

Loglikelihood and Information Criteria:

  Loglikelihood user model (H0)             -13432.921  -13432.921
  Scaling correction factor                                  0.788
      for the MLR correction                                      
  Loglikelihood unrestricted model (H1)     -13421.126  -13421.126
  Scaling correction factor                                  1.111
      for the MLR correction                                      
                                                                  
  Akaike (AIC)                               26895.842   26895.842
  Bayesian (BIC)                             26985.988   26985.988
  Sample-size adjusted Bayesian (SABIC)      26938.327   26938.327

Root Mean Square Error of Approximation:

  RMSEA                                          0.018       0.017
  90 Percent confidence interval - lower         0.006       0.005
  90 Percent confidence interval - upper         0.029       0.027
  P-value H_0: RMSEA <= 0.050                    1.000       1.000
  P-value H_0: RMSEA >= 0.080                    0.000       0.000
                                                                  
  Robust RMSEA                                               0.029
  90 Percent confidence interval - lower                     0.000
  90 Percent confidence interval - upper                     0.055
  P-value H_0: Robust RMSEA <= 0.050                         0.895
  P-value H_0: Robust RMSEA >= 0.080                         0.000

Standardized Root Mean Square Residual:

  SRMR                                           0.027       0.027

Parameter Estimates:

  Standard errors                             Sandwich
  Information bread                           Observed
  Observed information based on                Hessian

Latent Variables:
                   Estimate  Std.Err  z-value  P(>|z|)   Std.lv  Std.all
  SRHW1 =~                                                              
    srh_tm1_1 (L1)    0.651    0.014   45.597    0.000    0.651    0.900
    srh_tm2_1 (L2)    0.333    0.013   25.965    0.000    0.333    0.518
    chrnc_w1_ (L3)    0.826    0.039   21.237    0.000    0.826    0.474
  SRHW2 =~                                                              
    srh_tm1_2 (L1)    0.651    0.014   45.597    0.000    0.651    0.900
    srh_tm2_2 (L2)    0.333    0.013   25.965    0.000    0.333    0.518
    chrnc_w2_ (L3)    0.826    0.039   21.237    0.000    0.826    0.498

Covariances:
                         Estimate  Std.Err  z-value  P(>|z|)   Std.lv  Std.all
 .srh_item2_w1 ~~                                                             
   .srh_item2_w2            0.139    0.012   11.699    0.000    0.139    0.459
 .chronic_w1_reversed ~~                                                      
   .chrnc_w2_rvrsd          1.407    0.098   14.409    0.000    1.407    0.636
  SRHW1 ~~                                                                    
    SRHW2                   0.727    0.040   17.955    0.000    0.727    0.727

Intercepts:
                   Estimate  Std.Err  z-value  P(>|z|)   Std.lv  Std.all
    SRHW1             0.000                               0.000    0.000
    SRHW2            -0.026    0.037   -0.712    0.477   -0.026   -0.026
   .srh_tm1_1 (i1)    1.939    0.013  147.542    0.000    1.939    2.680
   .srh_tm1_2 (i1)    1.939    0.013  147.542    0.000    1.939    2.680
   .srh_tm2_1 (i2)    1.361    0.011  118.599    0.000    1.361    2.116
   .srh_tm2_2 (i2)    1.361    0.011  118.599    0.000    1.361    2.116
   .chrnc_w1_         8.404    0.032  263.575    0.000    8.404    4.822
   .chrnc_w2_         8.136    0.055  149.223    0.000    8.136    4.901

Variances:
                   Estimate  Std.Err  z-value  P(>|z|)   Std.lv  Std.all
    SRHW1             1.000                               1.000    1.000
    SRHW2             1.000                               1.000    1.000
   .srh_tm1_1 (e1)    0.099    0.013    7.396    0.000    0.099    0.190
   .srh_tm1_2 (e1)    0.099    0.013    7.396    0.000    0.099    0.190
   .srh_tm2_1 (e2)    0.303    0.007   44.690    0.000    0.303    0.732
   .srh_tm2_2 (e2)    0.303    0.007   44.690    0.000    0.303    0.732
   .chrnc_w1_         2.355    0.077   30.641    0.000    2.355    0.775
   .chrnc_w2_         2.074    0.136   15.242    0.000    2.074    0.752

# Comparing fit of the models

anova(fitSRHerror, fitSRHscalar_illnesses_unconstrained)

Scaled Chi-Squared Difference Test (method = "satorra.bentler.2001")

lavaan NOTE:
    The "Chisq" column contains standard test statistics, not the
    robust test that should be reported per model. A robust difference
    test is a function of two standard (not robust) statistics.
 
                                     Df   AIC   BIC  Chisq Chisq diff Df diff
fitSRHscalar_illnesses_unconstrained 10 26900 27002 23.258                   
fitSRHerror                          12 26896 26986 23.591    0.43071       2
                                     Pr(>Chisq)
fitSRHscalar_illnesses_unconstrained           
fitSRHerror                              0.8063

# Looking at fit indices to establish meaningful differences in fit statistics

fitmeasures(fitSRHscalar_illnesses_unconstrained, fit.measures = c("cfi.robust",
                                                                "rmsea.robust",
                                                                "srmr"))
  cfi.robust rmsea.robust         srmr 
       0.994        0.036        0.026 
fitmeasures(fitSRHerror, fit.measures = c("cfi.robust", "rmsea.robust", "srmr"))
  cfi.robust rmsea.robust         srmr 
       0.995        0.029        0.027 


# To test for a difference in SRH across time points, look at SRHW2 under
# Intercepts in the summary of the Error model. This is a comparison with SRHW1.
```

- Back to the Table of Contents

### R/S Engagement Longitudinal Invariance

```
# Reparameterizing from constraining first loading to the standardized latent
# variable approach.

# Configural model

modelRSconfig <- "RS_Eng_W1 =~ NA*rs_identity_w1 + prayer_w1 + attendance_w1 +
                      commitment_w1 + rcope_ben_total_w1 + rcope_sup_total_w1
RS_Eng_W2 =~ NA*rs_identity_w2 + prayer_w2 + attendance_w2 + commitment_w2 +
    rcope_ben_total_w2 + rcope_sup_total_w2
RS_Eng_W1~0
RS_Eng_W2~0
RS_Eng_W1~~1*RS_Eng_W1
RS_Eng_W2~~1*RS_Eng_W2
rs_identity_w1~~rs_identity_w2
prayer_w1~~prayer_w2
attendance_w1~~attendance_w2
commitment_w1~~commitment_w2
rcope_ben_total_w1~~rcope_ben_total_w2
rcope_sup_total_w1~~rcope_sup_total_w2"

# Fitting the model to a CFA and running summary statistics

fitRSconfig <- cfa(data = which_comes_first_data, model = modelRSconfig,
                   estimator = "MLR", missing = "ML", std.lv = T,
                   meanstructure = T)
summary(fitRSconfig, fit.measures = TRUE, standardized = TRUE)
lavaan 0.6.17 ended normally after 67 iterations

  Estimator                                         ML
  Optimization method                           NLMINB
  Number of model parameters                        43

  Number of observations                          3010
  Number of missing patterns                        37

Model Test User Model:
                                              Standard      Scaled
  Test Statistic                               482.648     446.555
  Degrees of freedom                                47          47
  P-value (Chi-square)                           0.000       0.000
  Scaling correction factor                                  1.081
    Yuan-Bentler correction (Mplus variant)                       

Model Test Baseline Model:

  Test statistic                             10104.231    8201.277
  Degrees of freedom                                66          66
  P-value                                        0.000       0.000
  Scaling correction factor                                  1.232

User Model versus Baseline Model:

  Comparative Fit Index (CFI)                    0.957       0.951
  Tucker-Lewis Index (TLI)                       0.939       0.931
                                                                  
  Robust Comparative Fit Index (CFI)                         0.963
  Robust Tucker-Lewis Index (TLI)                            0.948

Loglikelihood and Information Criteria:

  Loglikelihood user model (H0)             -33260.101  -33260.101
  Scaling correction factor                                  1.168
      for the MLR correction                                      
  Loglikelihood unrestricted model (H1)     -33018.777  -33018.777
  Scaling correction factor                                  1.122
      for the MLR correction                                      
                                                                  
  Akaike (AIC)                               66606.202   66606.202
  Bayesian (BIC)                             66864.619   66864.619
  Sample-size adjusted Bayesian (SABIC)      66727.992   66727.992

Root Mean Square Error of Approximation:

  RMSEA                                          0.055       0.053
  90 Percent confidence interval - lower         0.051       0.049
  90 Percent confidence interval - upper         0.060       0.058
  P-value H_0: RMSEA <= 0.050                    0.021       0.112
  P-value H_0: RMSEA >= 0.080                    0.000       0.000
                                                                  
  Robust RMSEA                                               0.080
  90 Percent confidence interval - lower                     0.069
  90 Percent confidence interval - upper                     0.091
  P-value H_0: Robust RMSEA <= 0.050                         0.000
  P-value H_0: Robust RMSEA >= 0.080                         0.493

Standardized Root Mean Square Residual:

  SRMR                                           0.042       0.042

Parameter Estimates:

  Standard errors                             Sandwich
  Information bread                           Observed
  Observed information based on                Hessian

Latent Variables:
                   Estimate  Std.Err  z-value  P(>|z|)   Std.lv  Std.all
  RS_Eng_W1 =~                                                          
    rs_identity_w1    0.447    0.011   39.186    0.000    0.447    0.687
    prayer_w1         2.000    0.038   52.126    0.000    2.000    0.822
    attendance_w1     1.696    0.038   44.294    0.000    1.696    0.604
    commitment_w1     0.764    0.023   32.680    0.000    0.764    0.668
    rcop_bn_ttl_w1    1.419    0.037   38.711    0.000    1.419    0.701
    rcop_sp_ttl_w1    1.857    0.036   51.417    0.000    1.857    0.874
  RS_Eng_W2 =~                                                          
    rs_identity_w2    0.498    0.019   26.800    0.000    0.498    0.750
    prayer_w2         2.047    0.059   34.778    0.000    2.047    0.832
    attendance_w2     1.831    0.069   26.439    0.000    1.831    0.616
    commitment_w2     0.824    0.042   19.617    0.000    0.824    0.706
    rcop_bn_ttl_w2    1.513    0.058   25.959    0.000    1.513    0.715
    rcop_sp_ttl_w2    1.947    0.047   41.390    0.000    1.947    0.901

Covariances:
                        Estimate  Std.Err  z-value  P(>|z|)   Std.lv  Std.all
 .rs_identity_w1 ~~                                                          
   .rs_identity_w2         0.089    0.011    7.786    0.000    0.089    0.429
 .prayer_w1 ~~                                                               
   .prayer_w2              0.864    0.130    6.636    0.000    0.864    0.457
 .attendance_w1 ~~                                                           
   .attendance_w2          3.273    0.243   13.495    0.000    3.273    0.625
 .commitment_w1 ~~                                                           
   .commitment_w2          0.129    0.039    3.328    0.001    0.129    0.183
 .rcope_ben_total_w1 ~~                                                      
   .rcop_bn_ttl_w2         0.405    0.120    3.388    0.001    0.405    0.190
 .rcope_sup_total_w1 ~~                                                      
   .rcop_sp_ttl_w2         0.112    0.095    1.185    0.236    0.112    0.116
  RS_Eng_W1 ~~                                                               
    RS_Eng_W2              0.905    0.012   76.264    0.000    0.905    0.905

Intercepts:
                   Estimate  Std.Err  z-value  P(>|z|)   Std.lv  Std.all
    RS_Eng_W1         0.000                               0.000    0.000
    RS_Eng_W2         0.000                               0.000    0.000
   .rs_identity_w1    1.458    0.012  122.668    0.000    1.458    2.243
   .prayer_w1         4.987    0.045  111.991    0.000    4.987    2.050
   .attendance_w1     3.761    0.051   73.475    0.000    3.761    1.340
   .commitment_w1     2.943    0.021  140.231    0.000    2.943    2.576
   .rcop_bn_ttl_w1    2.824    0.041   69.052    0.000    2.824    1.395
   .rcop_sp_ttl_w1    4.010    0.042   95.138    0.000    4.010    1.887
   .rs_identity_w2    1.425    0.021   67.891    0.000    1.425    2.147
   .prayer_w2         4.751    0.070   68.119    0.000    4.751    1.931
   .attendance_w2     3.492    0.095   36.808    0.000    3.492    1.175
   .commitment_w2     2.915    0.038   76.828    0.000    2.915    2.496
   .rcop_bn_ttl_w2    2.718    0.074   36.829    0.000    2.718    1.285
   .rcop_sp_ttl_w2    3.792    0.064   58.803    0.000    3.792    1.753

Variances:
                   Estimate  Std.Err  z-value  P(>|z|)   Std.lv  Std.all
    RS_Eng_W1         1.000                               1.000    1.000
    RS_Eng_W2         1.000                               1.000    1.000
   .rs_identity_w1    0.223    0.007   31.053    0.000    0.223    0.528
   .prayer_w1         1.916    0.091   21.038    0.000    1.916    0.324
   .attendance_w1     4.998    0.124   40.378    0.000    4.998    0.635
   .commitment_w1     0.722    0.026   27.453    0.000    0.722    0.553
   .rcop_bn_ttl_w1    2.085    0.077   27.075    0.000    2.085    0.509
   .rcop_sp_ttl_w1    1.069    0.071   14.969    0.000    1.069    0.237
   .rs_identity_w2    0.193    0.012   16.216    0.000    0.193    0.438
   .prayer_w2         1.865    0.175   10.647    0.000    1.865    0.308
   .attendance_w2     5.483    0.297   18.477    0.000    5.483    0.621
   .commitment_w2     0.685    0.058   11.818    0.000    0.685    0.502
   .rcop_bn_ttl_w2    2.185    0.146   14.959    0.000    2.185    0.488
   .rcop_sp_ttl_w2    0.884    0.091    9.699    0.000    0.884    0.189


# Metric model

modelRSmetric <- "RS_Eng_W1 =~ NA*L4*rs_identity_w1 + L5*prayer_w1 +
                      L6*attendance_w1 + L7*commitment_w1 +
                      L8*rcope_ben_total_w1 + L9*rcope_sup_total_w1
RS_Eng_W2 =~ NA*L4*rs_identity_w2 + L5*prayer_w2 + L6*attendance_w2 +
    L7*commitment_w2 + L8*rcope_ben_total_w2 + L9*rcope_sup_total_w2
RS_Eng_W1~0
RS_Eng_W2~0
RS_Eng_W1~~1*RS_Eng_W1
RS_Eng_W2~~1*RS_Eng_W2
rs_identity_w1~~rs_identity_w2
prayer_w1~~prayer_w2
attendance_w1~~attendance_w2
commitment_w1~~commitment_w2
rcope_ben_total_w1~~rcope_ben_total_w2
rcope_sup_total_w1~~rcope_sup_total_w2"

# Fitting model and running summary statistics

fitRSmetric <- cfa(data = which_comes_first_data, model = modelRSmetric,
                   estimator = "MLR", missing = "ML", std.lv = T,
                   meanstructure = T)
summary(fitRSmetric, fit.measures = TRUE, standardized = TRUE)
lavaan 0.6.17 ended normally after 67 iterations

  Estimator                                         ML
  Optimization method                           NLMINB
  Number of model parameters                        43
  Number of equality constraints                     6

  Number of observations                          3010
  Number of missing patterns                        37

Model Test User Model:
                                              Standard      Scaled
  Test Statistic                               494.030     465.516
  Degrees of freedom                                53          53
  P-value (Chi-square)                           0.000       0.000
  Scaling correction factor                                  1.061
    Yuan-Bentler correction (Mplus variant)                       

Model Test Baseline Model:

  Test statistic                             10104.231    8201.277
  Degrees of freedom                                66          66
  P-value                                        0.000       0.000
  Scaling correction factor                                  1.232

User Model versus Baseline Model:

  Comparative Fit Index (CFI)                    0.956       0.949
  Tucker-Lewis Index (TLI)                       0.945       0.937
                                                                  
  Robust Comparative Fit Index (CFI)                         0.962
  Robust Tucker-Lewis Index (TLI)                            0.953

Loglikelihood and Information Criteria:

  Loglikelihood user model (H0)             -33265.793  -33265.793
  Scaling correction factor                                  1.041
      for the MLR correction                                      
  Loglikelihood unrestricted model (H1)     -33018.777  -33018.777
  Scaling correction factor                                  1.122
      for the MLR correction                                      
                                                                  
  Akaike (AIC)                               66605.585   66605.585
  Bayesian (BIC)                             66827.944   66827.944
  Sample-size adjusted Bayesian (SABIC)      66710.380   66710.380

Root Mean Square Error of Approximation:

  RMSEA                                          0.053       0.051
  90 Percent confidence interval - lower         0.048       0.047
  90 Percent confidence interval - upper         0.057       0.055
  P-value H_0: RMSEA <= 0.050                    0.153       0.359
  P-value H_0: RMSEA >= 0.080                    0.000       0.000
                                                                  
  Robust RMSEA                                               0.076
  90 Percent confidence interval - lower                     0.066
  90 Percent confidence interval - upper                     0.086
  P-value H_0: Robust RMSEA <= 0.050                         0.000
  P-value H_0: Robust RMSEA >= 0.080                         0.274

Standardized Root Mean Square Residual:

  SRMR                                           0.052       0.052

Parameter Estimates:

  Standard errors                             Sandwich
  Information bread                           Observed
  Observed information based on                Hessian

Latent Variables:
                   Estimate  Std.Err  z-value  P(>|z|)   Std.lv  Std.all
  RS_Eng_W1 =~                                                          
    rs_dntt_1 (L4)    0.455    0.011   42.004    0.000    0.455    0.694
    prayer_w1 (L5)    2.004    0.037   54.740    0.000    2.004    0.822
    attndnc_1 (L6)    1.713    0.037   46.109    0.000    1.713    0.608
    cmmtmnt_1 (L7)    0.773    0.022   35.311    0.000    0.773    0.673
    rcp_bn__1 (L8)    1.435    0.033   44.021    0.000    1.435    0.705
    rcp_sp__1 (L9)    1.871    0.032   58.612    0.000    1.871    0.876
  RS_Eng_W2 =~                                                          
    rs_dntt_2 (L4)    0.455    0.011   42.004    0.000    0.455    0.717
    prayer_w2 (L5)    2.004    0.037   54.740    0.000    2.004    0.828
    attndnc_2 (L6)    1.713    0.037   46.109    0.000    1.713    0.589
    cmmtmnt_2 (L7)    0.773    0.022   35.311    0.000    0.773    0.682
    rcp_bn__2 (L8)    1.435    0.033   44.021    0.000    1.435    0.696
    rcp_sp__2 (L9)    1.871    0.032   58.612    0.000    1.871    0.893

Covariances:
                        Estimate  Std.Err  z-value  P(>|z|)   Std.lv  Std.all
 .rs_identity_w1 ~~                                                          
   .rs_identity_w2         0.089    0.011    7.780    0.000    0.089    0.426
 .prayer_w1 ~~                                                               
   .prayer_w2              0.859    0.128    6.733    0.000    0.859    0.456
 .attendance_w1 ~~                                                           
   .attendance_w2          3.290    0.240   13.693    0.000    3.290    0.626
 .commitment_w1 ~~                                                           
   .commitment_w2          0.129    0.039    3.339    0.001    0.129    0.184
 .rcope_ben_total_w1 ~~                                                      
   .rcop_bn_ttl_w2         0.407    0.120    3.389    0.001    0.407    0.190
 .rcope_sup_total_w1 ~~                                                      
   .rcop_sp_ttl_w2         0.111    0.095    1.163    0.245    0.111    0.114
  RS_Eng_W1 ~~                                                               
    RS_Eng_W2              0.902    0.012   72.966    0.000    0.902    0.902

Intercepts:
                   Estimate  Std.Err  z-value  P(>|z|)   Std.lv  Std.all
    RS_Eng_W1         0.000                               0.000    0.000
    RS_Eng_W2         0.000                               0.000    0.000
   .rs_identity_w1    1.458    0.012  122.678    0.000    1.458    2.225
   .prayer_w1         4.987    0.045  112.030    0.000    4.987    2.047
   .attendance_w1     3.760    0.051   73.465    0.000    3.760    1.335
   .commitment_w1     2.943    0.021  140.309    0.000    2.943    2.563
   .rcop_bn_ttl_w1    2.823    0.041   68.868    0.000    2.823    1.387
   .rcop_sp_ttl_w1    4.010    0.042   95.377    0.000    4.010    1.876
   .rs_identity_w2    1.422    0.021   66.964    0.000    1.422    2.239
   .prayer_w2         4.748    0.071   67.297    0.000    4.748    1.961
   .attendance_w2     3.485    0.094   37.108    0.000    3.485    1.198
   .commitment_w2     2.912    0.039   75.226    0.000    2.912    2.567
   .rcop_bn_ttl_w2    2.718    0.075   36.418    0.000    2.718    1.318
   .rcop_sp_ttl_w2    3.790    0.064   58.877    0.000    3.790    1.809

Variances:
                   Estimate  Std.Err  z-value  P(>|z|)   Std.lv  Std.all
    RS_Eng_W1         1.000                               1.000    1.000
    RS_Eng_W2         1.000                               1.000    1.000
   .rs_identity_w1    0.222    0.007   31.332    0.000    0.222    0.518
   .prayer_w1         1.922    0.089   21.480    0.000    1.922    0.324
   .attendance_w1     4.994    0.123   40.691    0.000    4.994    0.630
   .commitment_w1     0.720    0.026   27.732    0.000    0.720    0.546
   .rcop_bn_ttl_w1    2.082    0.075   27.739    0.000    2.082    0.503
   .rcop_sp_ttl_w1    1.066    0.068   15.605    0.000    1.066    0.233
   .rs_identity_w2    0.196    0.011   17.295    0.000    0.196    0.486
   .prayer_w2         1.848    0.162   11.379    0.000    1.848    0.315
   .attendance_w2     5.525    0.278   19.908    0.000    5.525    0.653
   .commitment_w2     0.689    0.055   12.484    0.000    0.689    0.536
   .rcop_bn_ttl_w2    2.195    0.140   15.662    0.000    2.195    0.516
   .rcop_sp_ttl_w2    0.890    0.087   10.227    0.000    0.890    0.203

# Comparing fit of the models

anova(fitRSmetric, fitRSconfig)

Scaled Chi-Squared Difference Test (method = "satorra.bentler.2001")

lavaan NOTE:
    The "Chisq" column contains standard test statistics, not the
    robust test that should be reported per model. A robust difference
    test is a function of two standard (not robust) statistics.
 
            Df   AIC   BIC  Chisq Chisq diff Df diff Pr(>Chisq)  
fitRSconfig 47 66606 66865 482.65                                
fitRSmetric 53 66606 66828 494.03     12.537       6    0.05101 .
---
Signif. codes:  0 '***' 0.001 '**' 0.01 '*' 0.05 '.' 0.1 ' ' 1


# Scalar model

modelRSscalar <- "RS_Eng_W1 =~ NA*L4*rs_identity_w1 + L5*prayer_w1 +
                      L6*attendance_w1 + L7*commitment_w1 +
                      L8*rcope_ben_total_w1 + L9*rcope_sup_total_w1
RS_Eng_W2 =~ NA*L4*rs_identity_w2 + L5*prayer_w2 + L6*attendance_w2 +
    L7*commitment_w2 + L8*rcope_ben_total_w2 + L9*rcope_sup_total_w2
RS_Eng_W1~0
RS_Eng_W2~NA*0
RS_Eng_W1~~1*RS_Eng_W1
RS_Eng_W2~~1*RS_Eng_W2
rs_identity_w1~~rs_identity_w2
prayer_w1~~prayer_w2
attendance_w1~~attendance_w2
commitment_w1~~commitment_w2
rcope_ben_total_w1~~rcope_ben_total_w2
rcope_sup_total_w1~~rcope_sup_total_w2
rs_identity_w1~i4*1
rs_identity_w2~i4*1
prayer_w1~i5*1
prayer_w2~i5*1
attendance_w1~i6*1
attendance_w2~i6*1
commitment_w1~i7*1
commitment_w2~i7*1
rcope_ben_total_w1~i8*1
rcope_ben_total_w2~i8*1
rcope_sup_total_w1~i9*1
rcope_sup_total_w2~i9*1"

# Fitting model and running summary statistics

fitRSscalar <- cfa(data = which_comes_first_data, model = modelRSscalar,
                   estimator = "MLR", missing = "ML", std.lv = T,
                   meanstructure = T)
summary(fitRSscalar, fit.measures = TRUE, standardized = TRUE)
lavaan 0.6.17 ended normally after 68 iterations

  Estimator                                         ML
  Optimization method                           NLMINB
  Number of model parameters                        44
  Number of equality constraints                    12

  Number of observations                          3010
  Number of missing patterns                        37

Model Test User Model:
                                              Standard      Scaled
  Test Statistic                               499.050     471.311
  Degrees of freedom                                58          58
  P-value (Chi-square)                           0.000       0.000
  Scaling correction factor                                  1.059
    Yuan-Bentler correction (Mplus variant)                       

Model Test Baseline Model:

  Test statistic                             10104.231    8201.277
  Degrees of freedom                                66          66
  P-value                                        0.000       0.000
  Scaling correction factor                                  1.232

User Model versus Baseline Model:

  Comparative Fit Index (CFI)                    0.956       0.949
  Tucker-Lewis Index (TLI)                       0.950       0.942
                                                                  
  Robust Comparative Fit Index (CFI)                         0.962
  Robust Tucker-Lewis Index (TLI)                            0.957

Loglikelihood and Information Criteria:

  Loglikelihood user model (H0)             -33268.303  -33268.303
  Scaling correction factor                                  0.900
      for the MLR correction                                      
  Loglikelihood unrestricted model (H1)     -33018.777  -33018.777
  Scaling correction factor                                  1.122
      for the MLR correction                                      
                                                                  
  Akaike (AIC)                               66600.605   66600.605
  Bayesian (BIC)                             66792.915   66792.915
  Sample-size adjusted Bayesian (SABIC)      66691.239   66691.239

Root Mean Square Error of Approximation:

  RMSEA                                          0.050       0.049
  90 Percent confidence interval - lower         0.046       0.045
  90 Percent confidence interval - upper         0.054       0.053
  P-value H_0: RMSEA <= 0.050                    0.449       0.704
  P-value H_0: RMSEA >= 0.080                    0.000       0.000
                                                                  
  Robust RMSEA                                               0.072
  90 Percent confidence interval - lower                     0.063
  90 Percent confidence interval - upper                     0.082
  P-value H_0: Robust RMSEA <= 0.050                         0.000
  P-value H_0: Robust RMSEA >= 0.080                         0.106

Standardized Root Mean Square Residual:

  SRMR                                           0.052       0.052

Parameter Estimates:

  Standard errors                             Sandwich
  Information bread                           Observed
  Observed information based on                Hessian

Latent Variables:
                   Estimate  Std.Err  z-value  P(>|z|)   Std.lv  Std.all
  RS_Eng_W1 =~                                                          
    rs_dntt_1 (L4)    0.455    0.011   42.076    0.000    0.455    0.694
    prayer_w1 (L5)    2.005    0.037   54.820    0.000    2.005    0.823
    attndnc_1 (L6)    1.717    0.037   46.563    0.000    1.717    0.609
    cmmtmnt_1 (L7)    0.772    0.022   35.284    0.000    0.772    0.673
    rcp_bn__1 (L8)    1.433    0.032   44.230    0.000    1.433    0.705
    rcp_sp__1 (L9)    1.871    0.032   58.798    0.000    1.871    0.875
  RS_Eng_W2 =~                                                          
    rs_dntt_2 (L4)    0.455    0.011   42.076    0.000    0.455    0.716
    prayer_w2 (L5)    2.005    0.037   54.820    0.000    2.005    0.828
    attndnc_2 (L6)    1.717    0.037   46.563    0.000    1.717    0.591
    cmmtmnt_2 (L7)    0.772    0.022   35.284    0.000    0.772    0.680
    rcp_bn__2 (L8)    1.433    0.032   44.230    0.000    1.433    0.695
    rcp_sp__2 (L9)    1.871    0.032   58.798    0.000    1.871    0.893

Covariances:
                        Estimate  Std.Err  z-value  P(>|z|)   Std.lv  Std.all
 .rs_identity_w1 ~~                                                          
   .rs_identity_w2         0.090    0.011    7.939    0.000    0.090    0.429
 .prayer_w1 ~~                                                               
   .prayer_w2              0.860    0.128    6.744    0.000    0.860    0.456
 .attendance_w1 ~~                                                           
   .attendance_w2          3.252    0.233   13.987    0.000    3.252    0.622
 .commitment_w1 ~~                                                           
   .commitment_w2          0.131    0.039    3.366    0.001    0.131    0.185
 .rcope_ben_total_w1 ~~                                                      
   .rcop_bn_ttl_w2         0.398    0.119    3.340    0.001    0.398    0.186
 .rcope_sup_total_w1 ~~                                                      
   .rcop_sp_ttl_w2         0.114    0.095    1.197    0.231    0.114    0.117
  RS_Eng_W1 ~~                                                               
    RS_Eng_W2              0.902    0.012   73.124    0.000    0.902    0.902

Intercepts:
                   Estimate  Std.Err  z-value  P(>|z|)   Std.lv  Std.all
    RS_Eng_W1         0.000                               0.000    0.000
    RS_Eng_W2        -0.108    0.024   -4.430    0.000   -0.108   -0.108
   .rs_dntt_1 (i4)    1.460    0.012  125.688    0.000    1.460    2.229
   .rs_dntt_2 (i4)    1.460    0.012  125.688    0.000    1.460    2.298
   .prayer_w1 (i5)    4.984    0.044  113.423    0.000    4.984    2.045
   .prayer_w2 (i5)    4.984    0.044  113.423    0.000    4.984    2.057
   .attndnc_1 (i6)    3.751    0.051   73.824    0.000    3.751    1.331
   .attndnc_2 (i6)    3.751    0.051   73.824    0.000    3.751    1.292
   .cmmtmnt_1 (i7)    2.951    0.020  146.254    0.000    2.951    2.572
   .cmmtmnt_2 (i7)    2.951    0.020  146.254    0.000    2.951    2.600
   .rcp_bn__1 (i8)    2.832    0.039   72.510    0.000    2.832    1.392
   .rcp_bn__2 (i8)    2.832    0.039   72.510    0.000    2.832    1.374
   .rcp_sp__1 (i9)    4.005    0.041   98.031    0.000    4.005    1.874
   .rcp_sp__2 (i9)    4.005    0.041   98.031    0.000    4.005    1.911

Variances:
                   Estimate  Std.Err  z-value  P(>|z|)   Std.lv  Std.all
    RS_Eng_W1         1.000                               1.000    1.000
    RS_Eng_W2         1.000                               1.000    1.000
   .rs_identity_w1    0.222    0.007   31.312    0.000    0.222    0.518
   .prayer_w1         1.921    0.089   21.468    0.000    1.921    0.323
   .attendance_w1     4.992    0.123   40.650    0.000    4.992    0.629
   .commitment_w1     0.721    0.026   27.720    0.000    0.721    0.548
   .rcop_bn_ttl_w1    2.082    0.075   27.755    0.000    2.082    0.503
   .rcop_sp_ttl_w1    1.067    0.068   15.601    0.000    1.067    0.234
   .rs_identity_w2    0.197    0.011   17.972    0.000    0.197    0.488
   .prayer_w2         1.849    0.163   11.377    0.000    1.849    0.315
   .attendance_w2     5.485    0.266   20.651    0.000    5.485    0.650
   .commitment_w2     0.692    0.055   12.680    0.000    0.692    0.538
   .rcop_bn_ttl_w2    2.194    0.140   15.714    0.000    2.194    0.516
   .rcop_sp_ttl_w2    0.892    0.088   10.158    0.000    0.892    0.203

# Comparing fit of the models

anova(fitRSscalar, fitRSmetric)

Scaled Chi-Squared Difference Test (method = "satorra.bentler.2001")

lavaan NOTE:
    The "Chisq" column contains standard test statistics, not the
    robust test that should be reported per model. A robust difference
    test is a function of two standard (not robust) statistics.
 
            Df   AIC   BIC  Chisq Chisq diff Df diff Pr(>Chisq)
fitRSmetric 53 66606 66828 494.03                              
fitRSscalar 58 66601 66793 499.05     4.8576       5     0.4335


# Error model

modelRSerror <- "RS_Eng_W1 =~ NA*L4*rs_identity_w1 + L5*prayer_w1 +
                      L6*attendance_w1 + L7*commitment_w1 +
                      L8*rcope_ben_total_w1 + L9*rcope_sup_total_w1
RS_Eng_W2 =~ NA*L4*rs_identity_w2 + L5*prayer_w2 + L6*attendance_w2 +
    L7*commitment_w2 + L8*rcope_ben_total_w2 + L9*rcope_sup_total_w2
RS_Eng_W1~0
RS_Eng_W2~NA*0
RS_Eng_W1~~1*RS_Eng_W1
RS_Eng_W2~~1*RS_Eng_W2
rs_identity_w1~~rs_identity_w2
prayer_w1~~prayer_w2
attendance_w1~~attendance_w2
commitment_w1~~commitment_w2
rcope_ben_total_w1~~rcope_ben_total_w2
rcope_sup_total_w1~~rcope_sup_total_w2
rs_identity_w1~i4*1
rs_identity_w2~i4*1
prayer_w1~i5*1
prayer_w2~i5*1
attendance_w1~i6*1
attendance_w2~i6*1
commitment_w1~i7*1
commitment_w2~i7*1
rcope_ben_total_w1~i8*1
rcope_ben_total_w2~i8*1
rcope_sup_total_w1~i9*1
rcope_sup_total_w2~i9*1
rs_identity_w1~~e4*rs_identity_w1
rs_identity_w2~~e4*rs_identity_w2
prayer_w1~~e5*prayer_w1
prayer_w2~~e5*prayer_w2
attendance_w1~~e6*attendance_w1
attendance_w2~~e6*attendance_w2
commitment_w1~~e7*commitment_w1
commitment_w2~~e7*commitment_w2
rcope_ben_total_w1~~e8*rcope_ben_total_w1
rcope_ben_total_w2~~e8*rcope_ben_total_w2
rcope_sup_total_w1~~e9*rcope_sup_total_w1
rcope_sup_total_w2~~e9*rcope_sup_total_w2"

# Fitting model and running summary statistics

fitRSerror <- cfa(data = which_comes_first_data, model = modelRSerror,
                  estimator = "MLR", missing = "ML", std.lv = T,
                  meanstructure = T)
summary(fitRSerror, fit.measures = TRUE, standardized = TRUE)
lavaan 0.6.17 ended normally after 48 iterations

  Estimator                                         ML
  Optimization method                           NLMINB
  Number of model parameters                        44
  Number of equality constraints                    18

  Number of observations                          3010
  Number of missing patterns                        37

Model Test User Model:
                                              Standard      Scaled
  Test Statistic                               509.606     483.208
  Degrees of freedom                                64          64
  P-value (Chi-square)                           0.000       0.000
  Scaling correction factor                                  1.055
    Yuan-Bentler correction (Mplus variant)                       

Model Test Baseline Model:

  Test statistic                             10104.231    8201.277
  Degrees of freedom                                66          66
  P-value                                        0.000       0.000
  Scaling correction factor                                  1.232

User Model versus Baseline Model:

  Comparative Fit Index (CFI)                    0.956       0.948
  Tucker-Lewis Index (TLI)                       0.954       0.947
                                                                  
  Robust Comparative Fit Index (CFI)                         0.961
  Robust Tucker-Lewis Index (TLI)                            0.960

Loglikelihood and Information Criteria:

  Loglikelihood user model (H0)             -33273.580  -33273.580
  Scaling correction factor                                  0.762
      for the MLR correction                                      
  Loglikelihood unrestricted model (H1)     -33018.777  -33018.777
  Scaling correction factor                                  1.122
      for the MLR correction                                      
                                                                  
  Akaike (AIC)                               66599.161   66599.161
  Bayesian (BIC)                             66755.413   66755.413
  Sample-size adjusted Bayesian (SABIC)      66672.801   66672.801

Root Mean Square Error of Approximation:

  RMSEA                                          0.048       0.047
  90 Percent confidence interval - lower         0.044       0.043
  90 Percent confidence interval - upper         0.052       0.050
  P-value H_0: RMSEA <= 0.050                    0.784       0.925
  P-value H_0: RMSEA >= 0.080                    0.000       0.000
                                                                  
  Robust RMSEA                                               0.070
  90 Percent confidence interval - lower                     0.061
  90 Percent confidence interval - upper                     0.079
  P-value H_0: Robust RMSEA <= 0.050                         0.000
  P-value H_0: Robust RMSEA >= 0.080                         0.039

Standardized Root Mean Square Residual:

  SRMR                                           0.053       0.053

Parameter Estimates:

  Standard errors                             Sandwich
  Information bread                           Observed
  Observed information based on                Hessian

Latent Variables:
                   Estimate  Std.Err  z-value  P(>|z|)   Std.lv  Std.all
  RS_Eng_W1 =~                                                          
    rs_dntt_1 (L4)    0.454    0.011   41.927    0.000    0.454    0.696
    prayer_w1 (L5)    2.006    0.037   54.742    0.000    2.006    0.824
    attndnc_1 (L6)    1.718    0.037   46.757    0.000    1.718    0.607
    cmmtmnt_1 (L7)    0.772    0.022   35.314    0.000    0.772    0.674
    rcp_bn__1 (L8)    1.435    0.032   44.375    0.000    1.435    0.703
    rcp_sp__1 (L9)    1.871    0.032   58.519    0.000    1.871    0.879
  RS_Eng_W2 =~                                                          
    rs_dntt_2 (L4)    0.454    0.011   41.927    0.000    0.454    0.696
    prayer_w2 (L5)    2.006    0.037   54.742    0.000    2.006    0.824
    attndnc_2 (L6)    1.718    0.037   46.757    0.000    1.718    0.607
    cmmtmnt_2 (L7)    0.772    0.022   35.314    0.000    0.772    0.674
    rcp_bn__2 (L8)    1.435    0.032   44.375    0.000    1.435    0.703
    rcp_sp__2 (L9)    1.871    0.032   58.519    0.000    1.871    0.879

Covariances:
                        Estimate  Std.Err  z-value  P(>|z|)   Std.lv  Std.all
 .rs_identity_w1 ~~                                                          
   .rs_identity_w2         0.097    0.012    8.369    0.000    0.097    0.445
 .prayer_w1 ~~                                                               
   .prayer_w2              0.879    0.123    7.158    0.000    0.879    0.460
 .attendance_w1 ~~                                                           
   .attendance_w2          3.082    0.208   14.804    0.000    3.082    0.609
 .commitment_w1 ~~                                                           
   .commitment_w2          0.134    0.039    3.404    0.001    0.134    0.187
 .rcope_ben_total_w1 ~~                                                      
   .rcop_bn_ttl_w2         0.384    0.116    3.314    0.001    0.384    0.183
 .rcope_sup_total_w1 ~~                                                      
   .rcop_sp_ttl_w2         0.139    0.099    1.393    0.163    0.139    0.135
  RS_Eng_W1 ~~                                                               
    RS_Eng_W2              0.903    0.012   73.233    0.000    0.903    0.903

Intercepts:
                   Estimate  Std.Err  z-value  P(>|z|)   Std.lv  Std.all
    RS_Eng_W1         0.000                               0.000    0.000
    RS_Eng_W2        -0.106    0.024   -4.382    0.000   -0.106   -0.106
   .rs_dntt_1 (i4)    1.459    0.012  125.397    0.000    1.459    2.239
   .rs_dntt_2 (i4)    1.459    0.012  125.397    0.000    1.459    2.239
   .prayer_w1 (i5)    4.984    0.044  113.459    0.000    4.984    2.046
   .prayer_w2 (i5)    4.984    0.044  113.459    0.000    4.984    2.046
   .attndnc_1 (i6)    3.751    0.051   73.780    0.000    3.751    1.325
   .attndnc_2 (i6)    3.751    0.051   73.780    0.000    3.751    1.325
   .cmmtmnt_1 (i7)    2.950    0.020  145.997    0.000    2.950    2.576
   .cmmtmnt_2 (i7)    2.950    0.020  145.997    0.000    2.950    2.576
   .rcp_bn__1 (i8)    2.832    0.039   72.501    0.000    2.832    1.388
   .rcp_bn__2 (i8)    2.832    0.039   72.501    0.000    2.832    1.388
   .rcp_sp__1 (i9)    4.006    0.041   98.026    0.000    4.006    1.882
   .rcp_sp__2 (i9)    4.006    0.041   98.026    0.000    4.006    1.882

Variances:
                   Estimate  Std.Err  z-value  P(>|z|)   Std.lv  Std.all
    RS_Eng_W1         1.000                               1.000    1.000
    RS_Eng_W2         1.000                               1.000    1.000
   .rs_dntt_1 (e4)    0.219    0.007   32.645    0.000    0.219    0.516
   .rs_dntt_2 (e4)    0.219    0.007   32.645    0.000    0.219    0.516
   .prayer_w1 (e5)    1.908    0.085   22.458    0.000    1.908    0.322
   .prayer_w2 (e5)    1.908    0.085   22.458    0.000    1.908    0.322
   .attndnc_1 (e6)    5.061    0.120   42.195    0.000    5.061    0.632
   .attndnc_2 (e6)    5.061    0.120   42.195    0.000    5.061    0.632
   .cmmtmnt_1 (e7)    0.715    0.025   28.873    0.000    0.715    0.546
   .cmmtmnt_2 (e7)    0.715    0.025   28.873    0.000    0.715    0.546
   .rcp_bn__1 (e8)    2.103    0.070   30.137    0.000    2.103    0.505
   .rcp_bn__2 (e8)    2.103    0.070   30.137    0.000    2.103    0.505
   .rcp_sp__1 (e9)    1.030    0.062   16.672    0.000    1.030    0.227
   .rcp_sp__2 (e9)    1.030    0.062   16.672    0.000    1.030    0.227

# Comparing fit of the models

anova(fitRSerror, fitRSscalar)

Scaled Chi-Squared Difference Test (method = "satorra.bentler.2001")

lavaan NOTE:
    The "Chisq" column contains standard test statistics, not the
    robust test that should be reported per model. A robust difference
    test is a function of two standard (not robust) statistics.
 
            Df   AIC   BIC  Chisq Chisq diff Df diff Pr(>Chisq)
fitRSscalar 58 66601 66793 499.05                              
fitRSerror  64 66599 66755 509.61     10.412       6     0.1083


# To test for a difference in RS across time points, look at RS_Eng_W2 under
# Intercepts in the summary of the Error model. This is a comparison with RS W1.
```

- Back to the Table of Contents

### Full Model with Longitudinal Invariance

```
fullmodelLI <- "SRHW1 =~ NA*L1*srh_item1_w1 + L2*srh_item2_w1 +
                  L3*chronic_w1_reversed
SRHW2 =~ NA*L1*srh_item1_w2 + L2*srh_item2_w2 + L3*chronic_w2_reversed
RS_Eng_W1 =~ NA*L4*rs_identity_w1 + L5*prayer_w1 + L6*attendance_w1 +
    L7*commitment_w1 + L8*rcope_ben_total_w1 + L9*rcope_sup_total_w1
RS_Eng_W2 =~ NA*L4*rs_identity_w2 + L5*prayer_w2 + L6*attendance_w2 +
    L7*commitment_w2 + L8*rcope_ben_total_w2 + L9*rcope_sup_total_w2
SRHW1~0
SRHW2~NA*0
RS_Eng_W1~0
RS_Eng_W2~NA*0
SRHW1~~1*SRHW1
SRHW2~~1*SRHW2
RS_Eng_W1~~1*RS_Eng_W1
RS_Eng_W2~~1*RS_Eng_W2
srh_item2_w1~~srh_item2_w2
chronic_w1_reversed~~chronic_w2_reversed
rs_identity_w1~~rs_identity_w2
prayer_w1~~prayer_w2
attendance_w1~~attendance_w2
commitment_w1~~commitment_w2
rcope_ben_total_w1~~rcope_ben_total_w2
rcope_sup_total_w1~~rcope_sup_total_w2
srh_item1_w1~i1*1
srh_item1_w2~i1*1
srh_item2_w1~i2*1
srh_item2_w2~i2*1
rs_identity_w1~i4*1
rs_identity_w2~i4*1
prayer_w1~i5*1
prayer_w2~i5*1
attendance_w1~i6*1
attendance_w2~i6*1
commitment_w1~i7*1
commitment_w2~i7*1
rcope_ben_total_w1~i8*1
rcope_ben_total_w2~i8*1
rcope_sup_total_w1~i9*1
rcope_sup_total_w2~i9*1
srh_item1_w1~~e1*srh_item1_w1
srh_item1_w2~~e1*srh_item1_w2
srh_item2_w1~~e2*srh_item2_w1
srh_item2_w2~~e2*srh_item2_w2
rs_identity_w1~~e4*rs_identity_w1
rs_identity_w2~~e4*rs_identity_w2
prayer_w1~~e5*prayer_w1
prayer_w2~~e5*prayer_w2
attendance_w1~~e6*attendance_w1
attendance_w2~~e6*attendance_w2
commitment_w1~~e7*commitment_w1
commitment_w2~~e7*commitment_w2
rcope_ben_total_w1~~e8*rcope_ben_total_w1
rcope_ben_total_w2~~e8*rcope_ben_total_w2
rcope_sup_total_w1~~e9*rcope_sup_total_w1
rcope_sup_total_w2~~e9*rcope_sup_total_w2"

# Fitting model and running summary statistics

fitfullmodelLI <- cfa(data = which_comes_first_data, model = fullmodelLI,
                      estimator = "MLR", missing = "ML", std.lv = T,
                      meanstructure = T)
summary(fitfullmodelLI, fit.measures = TRUE, standardized = TRUE)
lavaan 0.6.17 ended normally after 71 iterations

  Estimator                                         ML
  Optimization method                           NLMINB
  Number of model parameters                        70
  Number of equality constraints                    25

  Number of observations                          3010
  Number of missing patterns                        51

Model Test User Model:
                                              Standard      Scaled
  Test Statistic                               768.079     733.118
  Degrees of freedom                               144         144
  P-value (Chi-square)                           0.000       0.000
  Scaling correction factor                                  1.048
    Yuan-Bentler correction (Mplus variant)                       

Model Test Baseline Model:

  Test statistic                             12671.421   11199.710
  Degrees of freedom                               153         153
  P-value                                        0.000       0.000
  Scaling correction factor                                  1.131

User Model versus Baseline Model:

  Comparative Fit Index (CFI)                    0.950       0.947
  Tucker-Lewis Index (TLI)                       0.947       0.943
                                                                  
  Robust Comparative Fit Index (CFI)                         0.961
  Robust Tucker-Lewis Index (TLI)                            0.959

Loglikelihood and Information Criteria:

  Loglikelihood user model (H0)             -46700.853  -46700.853
  Scaling correction factor                                  0.780
      for the MLR correction                                      
  Loglikelihood unrestricted model (H1)     -46316.813  -46316.813
  Scaling correction factor                                  1.087
      for the MLR correction                                      
                                                                  
  Akaike (AIC)                               93491.705   93491.705
  Bayesian (BIC)                             93762.141   93762.141
  Sample-size adjusted Bayesian (SABIC)      93619.159   93619.159

Root Mean Square Error of Approximation:

  RMSEA                                          0.038       0.037
  90 Percent confidence interval - lower         0.035       0.034
  90 Percent confidence interval - upper         0.041       0.039
  P-value H_0: RMSEA <= 0.050                    1.000       1.000
  P-value H_0: RMSEA >= 0.080                    0.000       0.000
                                                                  
  Robust RMSEA                                               0.053
  90 Percent confidence interval - lower                     0.047
  90 Percent confidence interval - upper                     0.059
  P-value H_0: Robust RMSEA <= 0.050                         0.219
  P-value H_0: Robust RMSEA >= 0.080                         0.000

Standardized Root Mean Square Residual:

  SRMR                                           0.057       0.057

Parameter Estimates:

  Standard errors                             Sandwich
  Information bread                           Observed
  Observed information based on                Hessian

Latent Variables:
                   Estimate  Std.Err  z-value  P(>|z|)   Std.lv  Std.all
  SRHW1 =~                                                              
    srh_tm1_1 (L1)    0.650    0.014   45.355    0.000    0.650    0.898
    srh_tm2_1 (L2)    0.333    0.013   25.997    0.000    0.333    0.518
    chrnc_w1_ (L3)    0.829    0.039   21.078    0.000    0.829    0.476
  SRHW2 =~                                                              
    srh_tm1_2 (L1)    0.650    0.014   45.355    0.000    0.650    0.898
    srh_tm2_2 (L2)    0.333    0.013   25.997    0.000    0.333    0.518
    chrnc_w2_ (L3)    0.829    0.039   21.078    0.000    0.829    0.499
  RS_Eng_W1 =~                                                          
    rs_dntt_1 (L4)    0.454    0.011   41.909    0.000    0.454    0.696
    prayer_w1 (L5)    2.007    0.037   54.772    0.000    2.007    0.824
    attndnc_1 (L6)    1.716    0.037   46.549    0.000    1.716    0.606
    cmmtmnt_1 (L7)    0.772    0.022   35.272    0.000    0.772    0.674
    rcp_bn__1 (L8)    1.435    0.032   44.436    0.000    1.435    0.704
    rcp_sp__1 (L9)    1.872    0.032   58.582    0.000    1.872    0.879
  RS_Eng_W2 =~                                                          
    rs_dntt_2 (L4)    0.454    0.011   41.909    0.000    0.454    0.696
    prayer_w2 (L5)    2.007    0.037   54.772    0.000    2.007    0.824
    attndnc_2 (L6)    1.716    0.037   46.549    0.000    1.716    0.606
    cmmtmnt_2 (L7)    0.772    0.022   35.272    0.000    0.772    0.674
    rcp_bn__2 (L8)    1.435    0.032   44.436    0.000    1.435    0.704
    rcp_sp__2 (L9)    1.872    0.032   58.582    0.000    1.872    0.879

Covariances:
                         Estimate  Std.Err  z-value  P(>|z|)   Std.lv  Std.all
 .srh_item2_w1 ~~                                                             
   .srh_item2_w2            0.139    0.012   11.669    0.000    0.139    0.459
 .chronic_w1_reversed ~~                                                      
   .chrnc_w2_rvrsd          1.407    0.098   14.401    0.000    1.407    0.637
 .rs_identity_w1 ~~                                                           
   .rs_identity_w2          0.098    0.012    8.418    0.000    0.098    0.446
 .prayer_w1 ~~                                                                
   .prayer_w2               0.882    0.122    7.219    0.000    0.882    0.462
 .attendance_w1 ~~                                                            
   .attendance_w2           3.083    0.208   14.804    0.000    3.083    0.608
 .commitment_w1 ~~                                                            
   .commitment_w2           0.135    0.039    3.439    0.001    0.135    0.188
 .rcope_ben_total_w1 ~~                                                       
   .rcop_bn_ttl_w2          0.383    0.116    3.313    0.001    0.383    0.182
 .rcope_sup_total_w1 ~~                                                       
   .rcop_sp_ttl_w2          0.134    0.099    1.353    0.176    0.134    0.131
  SRHW1 ~~                                                                    
    SRHW2                   0.729    0.040   18.109    0.000    0.729    0.729
    RS_Eng_W1              -0.052    0.021   -2.464    0.014   -0.052   -0.052
    RS_Eng_W2               0.008    0.029    0.277    0.782    0.008    0.008
  SRHW2 ~~                                                                    
    RS_Eng_W1               0.014    0.038    0.357    0.721    0.014    0.014
    RS_Eng_W2               0.045    0.038    1.197    0.231    0.045    0.045
  RS_Eng_W1 ~~                                                                
    RS_Eng_W2               0.903    0.012   73.191    0.000    0.903    0.903

Intercepts:
                   Estimate  Std.Err  z-value  P(>|z|)   Std.lv  Std.all
    SRHW1             0.000                               0.000    0.000
    SRHW2            -0.024    0.037   -0.659    0.510   -0.024   -0.024
    RS_Eng_W1         0.000                               0.000    0.000
    RS_Eng_W2        -0.116    0.025   -4.709    0.000   -0.116   -0.116
   .srh_tm1_1 (i1)    1.939    0.013  147.544    0.000    1.939    2.680
   .srh_tm1_2 (i1)    1.939    0.013  147.544    0.000    1.939    2.680
   .srh_tm2_1 (i2)    1.361    0.011  118.600    0.000    1.361    2.116
   .srh_tm2_2 (i2)    1.361    0.011  118.600    0.000    1.361    2.116
   .rs_dntt_1 (i4)    1.459    0.012  125.384    0.000    1.459    2.238
   .rs_dntt_2 (i4)    1.459    0.012  125.384    0.000    1.459    2.238
   .prayer_w1 (i5)    4.984    0.044  113.456    0.000    4.984    2.046
   .prayer_w2 (i5)    4.984    0.044  113.456    0.000    4.984    2.046
   .attndnc_1 (i6)    3.751    0.051   73.773    0.000    3.751    1.325
   .attndnc_2 (i6)    3.751    0.051   73.773    0.000    3.751    1.325
   .cmmtmnt_1 (i7)    2.950    0.020  145.996    0.000    2.950    2.576
   .cmmtmnt_2 (i7)    2.950    0.020  145.996    0.000    2.950    2.576
   .rcp_bn__1 (i8)    2.831    0.039   72.474    0.000    2.831    1.388
   .rcp_bn__2 (i8)    2.831    0.039   72.474    0.000    2.831    1.388
   .rcp_sp__1 (i9)    4.005    0.041   97.943    0.000    4.005    1.881
   .rcp_sp__2 (i9)    4.005    0.041   97.943    0.000    4.005    1.881
   .chrnc_w1_         8.404    0.032  263.575    0.000    8.404    4.822
   .chrnc_w2_         8.135    0.055  149.088    0.000    8.135    4.894

Variances:
                   Estimate  Std.Err  z-value  P(>|z|)   Std.lv  Std.all
    SRHW1             1.000                               1.000    1.000
    SRHW2             1.000                               1.000    1.000
    RS_Eng_W1         1.000                               1.000    1.000
    RS_Eng_W2         1.000                               1.000    1.000
   .srh_tm1_1 (e1)    0.101    0.013    7.480    0.000    0.101    0.193
   .srh_tm1_2 (e1)    0.101    0.013    7.480    0.000    0.101    0.193
   .srh_tm2_1 (e2)    0.303    0.007   44.723    0.000    0.303    0.731
   .srh_tm2_2 (e2)    0.303    0.007   44.723    0.000    0.303    0.731
   .rs_dntt_1 (e4)    0.219    0.007   32.639    0.000    0.219    0.516
   .rs_dntt_2 (e4)    0.219    0.007   32.639    0.000    0.219    0.516
   .prayer_w1 (e5)    1.907    0.085   22.430    0.000    1.907    0.321
   .prayer_w2 (e5)    1.907    0.085   22.430    0.000    1.907    0.321
   .attndnc_1 (e6)    5.067    0.120   42.171    0.000    5.067    0.633
   .attndnc_2 (e6)    5.067    0.120   42.171    0.000    5.067    0.633
   .cmmtmnt_1 (e7)    0.716    0.025   28.853    0.000    0.716    0.546
   .cmmtmnt_2 (e7)    0.716    0.025   28.853    0.000    0.716    0.546
   .rcp_bn__1 (e8)    2.102    0.070   30.139    0.000    2.102    0.505
   .rcp_bn__2 (e8)    2.102    0.070   30.139    0.000    2.102    0.505
   .rcp_sp__1 (e9)    1.026    0.062   16.632    0.000    1.026    0.227
   .rcp_sp__2 (e9)    1.026    0.062   16.632    0.000    1.026    0.227
   .chrnc_w1_         2.350    0.077   30.529    0.000    2.350    0.774
   .chrnc_w2_         2.076    0.136   15.259    0.000    2.076    0.751


# Looking at Residuals

lavResiduals(fitfullmodelLI)
$type
[1] "cor.bentler"

$cov
                    sr_1_1 sr_2_1 chr_1_ sr_1_2 sr_2_2 chr_2_ rs_d_1 pryr_1
srh_item1_w1        -0.003                                                 
srh_item2_w1        -0.002 -0.006                                          
chronic_w1_reversed  0.009 -0.036  0.007                                   
srh_item1_w2        -0.004 -0.006  0.086  0.028                            
srh_item2_w2         0.002 -0.001 -0.009  0.039  0.022                     
chronic_w2_reversed  0.040 -0.019  0.039  0.048 -0.022  0.027              
rs_identity_w1       0.028  0.078 -0.104 -0.016  0.064 -0.120 -0.008       
prayer_w1           -0.010  0.036 -0.119 -0.021  0.058 -0.103  0.023  0.000
attendance_w1        0.074  0.102 -0.042  0.028  0.105 -0.042  0.069 -0.002
commitment_w1        0.053  0.096 -0.077 -0.004  0.083 -0.110  0.018  0.002
rcope_ben_total_w1  -0.020 -0.004 -0.022  0.023  0.010  0.051 -0.110 -0.079
rcope_sup_total_w1  -0.018  0.014 -0.106 -0.005  0.053 -0.078 -0.081 -0.020
rs_identity_w2       0.026  0.065 -0.090 -0.026  0.059 -0.098  0.012  0.062
prayer_w2            0.012  0.072 -0.137  0.003  0.059 -0.106  0.060  0.018
attendance_w2        0.045  0.056 -0.133 -0.012  0.115 -0.101  0.088  0.020
commitment_w2        0.081  0.115 -0.079  0.033  0.112 -0.085  0.103  0.050
rcope_ben_total_w2  -0.015 -0.004 -0.068 -0.042  0.039 -0.033 -0.120 -0.013
rcope_sup_total_w2  -0.032  0.020 -0.122 -0.011  0.080 -0.079 -0.001  0.026
                    attn_1 cmmt_1 rcp_b__1 rcp_s__1 rs_d_2 pryr_2 attn_2 cmmt_2
srh_item1_w1                                                                   
srh_item2_w1                                                                   
chronic_w1_reversed                                                            
srh_item1_w2                                                                   
srh_item2_w2                                                                   
chronic_w2_reversed                                                            
rs_identity_w1                                                                 
prayer_w1                                                                      
attendance_w1       -0.018                                                     
commitment_w1        0.001 -0.004                                              
rcope_ben_total_w1  -0.052 -0.067   -0.037                                     
rcope_sup_total_w1  -0.063 -0.030    0.057   -0.021                            
rs_identity_w2       0.109  0.034   -0.071   -0.013  0.044                     
prayer_w2            0.038  0.022   -0.069    0.019  0.103  0.020              
attendance_w2        0.056  0.046   -0.039   -0.008  0.147  0.059  0.115       
commitment_w2        0.098  0.036   -0.021    0.016  0.104  0.070  0.094  0.042
rcope_ben_total_w2   0.042 -0.034   -0.028   -0.018 -0.008 -0.011  0.064  0.020
rcope_sup_total_w2   0.024  0.018   -0.028    0.003  0.079  0.023  0.051  0.030
                    rcp_b__2 rcp_s__2
srh_item1_w1                         
srh_item2_w1                         
chronic_w1_reversed                  
srh_item1_w2                         
srh_item2_w2                         
chronic_w2_reversed                  
rs_identity_w1                       
prayer_w1                            
attendance_w1                        
commitment_w1                        
rcope_ben_total_w1                   
rcope_sup_total_w1                   
rs_identity_w2                       
prayer_w2                            
attendance_w2                        
commitment_w2                        
rcope_ben_total_w2     0.048         
rcope_sup_total_w2     0.089    0.018

$mean
       srh_item1_w1        srh_item2_w1 chronic_w1_reversed        srh_item1_w2 
              0.001              -0.002               0.000               0.004 
       srh_item2_w2 chronic_w2_reversed      rs_identity_w1           prayer_w1 
              0.014               0.004              -0.002               0.001 
      attendance_w1       commitment_w1  rcope_ben_total_w1  rcope_sup_total_w1 
              0.003              -0.007               0.001               0.000 
     rs_identity_w2           prayer_w2       attendance_w2       commitment_w2 
              0.003              -0.018              -0.033               0.007 
 rcope_ben_total_w2  rcope_sup_total_w2 
              0.030              -0.001 

$cov.z
                    sr_1_1 sr_2_1 chr_1_ sr_1_2 sr_2_2 chr_2_ rs_d_1 pryr_1
srh_item1_w1        -0.359                                                 
srh_item2_w1        -0.249 -0.622                                          
chronic_w1_reversed  1.073 -3.022  0.905                                   
srh_item1_w2        -0.162 -0.232  2.358  0.554                            
srh_item2_w2         0.072 -0.042 -0.243  1.024  0.553                     
chronic_w2_reversed  1.349 -0.631  1.802  1.144 -0.550  0.724              
rs_identity_w1       2.141  4.674 -6.228 -0.586  1.918 -3.866 -0.720       
prayer_w1           -1.104  2.310 -7.793 -1.127  1.966 -3.706  2.216 -0.057
attendance_w1        4.861  5.778 -2.366  0.946  3.021 -1.350  5.225 -0.173
commitment_w1        3.812  5.644 -4.786 -0.159  2.753 -3.704  1.400  0.217
rcope_ben_total_w1  -1.318 -0.238 -1.166  0.818  0.288  1.633 -8.398 -7.190
rcope_sup_total_w1  -1.850  0.837 -6.437 -0.253  1.733 -2.648 -8.010 -2.280
rs_identity_w2       1.093  2.155 -3.034 -1.000  1.825 -2.828  0.716  2.336
prayer_w2            0.529  2.635 -5.070  0.147  1.899 -3.315  2.511  1.250
attendance_w2        1.574  2.019 -3.892 -0.409  3.310 -2.873  3.556  1.012
commitment_w2        2.563  3.388 -2.093  1.087  3.130 -2.219  3.176  1.552
rcope_ben_total_w2  -0.495 -0.133 -1.908 -1.436  1.106 -0.929 -3.802 -0.516
rcope_sup_total_w2  -1.566  0.737 -4.496 -0.519  2.627 -2.548 -0.025  1.130
                    attn_1 cmmt_1 rcp_b__1 rcp_s__1 rs_d_2 pryr_2 attn_2 cmmt_2
srh_item1_w1                                                                   
srh_item2_w1                                                                   
chronic_w1_reversed                                                            
srh_item1_w2                                                                   
srh_item2_w2                                                                   
chronic_w2_reversed                                                            
rs_identity_w1                                                                 
prayer_w1                                                                      
attendance_w1       -1.767                                                     
commitment_w1        0.063 -0.347                                              
rcope_ben_total_w1  -3.568 -4.731   -2.365                                     
rcope_sup_total_w1  -6.388 -2.539    4.704   -1.895                            
rs_identity_w2       4.064  1.348   -2.619   -0.511  1.301                     
prayer_w2            1.469  1.019   -3.040    0.842  3.454  0.739              
attendance_w2        4.103  2.206   -1.461   -0.366  5.378  2.321  5.326       
commitment_w2        3.107  2.055   -0.724    0.531  2.901  2.042  3.205  0.987
rcope_ben_total_w2   1.337 -1.131   -1.474   -0.633 -0.257 -0.410  2.089  0.639
rcope_sup_total_w2   0.944  0.738   -1.238    0.165  2.758  0.836  2.025  0.910
                    rcp_b__2 rcp_s__2
srh_item1_w1                         
srh_item2_w1                         
chronic_w1_reversed                  
srh_item1_w2                         
srh_item2_w2                         
chronic_w2_reversed                  
rs_identity_w1                       
prayer_w1                            
attendance_w1                        
commitment_w1                        
rcope_ben_total_w1                   
rcope_sup_total_w1                   
rs_identity_w2                       
prayer_w2                            
attendance_w2                        
commitment_w2                        
rcope_ben_total_w2     1.583         
rcope_sup_total_w2     3.055    0.626

$mean.z
       srh_item1_w1        srh_item2_w1 chronic_w1_reversed        srh_item1_w2 
              0.465              -0.535               0.000               0.345 
       srh_item2_w2 chronic_w2_reversed      rs_identity_w1           prayer_w1 
              0.485               0.314              -0.491               0.542 
      attendance_w1       commitment_w1  rcope_ben_total_w1  rcope_sup_total_w1 
              1.014              -1.625               0.146              -0.082 
     rs_identity_w2           prayer_w2       attendance_w2       commitment_w2 
              0.140              -1.049              -1.371               0.237 
 rcope_ben_total_w2  rcope_sup_total_w2 
              1.122              -0.036 

$summary
                           cov   mean  total
srmr                     0.060  0.012  0.057
srmr.se                  0.003  0.004  0.003
srmr.exactfit.z         11.317  0.000 11.211
srmr.exactfit.pvalue     0.000  0.500  0.000
usrmr                    0.054  0.000  0.052
usrmr.se                 0.005  0.011  0.005
usrmr.ci.lower           0.046 -0.017  0.044
usrmr.ci.upper           0.062  0.017  0.059
usrmr.closefit.h0.value  0.050  0.050  0.050
usrmr.closefit.z         0.914 -4.743  0.369
usrmr.closefit.pvalue    0.180  1.000  0.356


# Looking at Modification Indices

modindices(fitfullmodelLI, sort. = TRUE)
Warning in modindices(fitfullmodelLI, sort. = TRUE): lavaan WARNING: the modindices() function ignores equality constraints;
          use lavTestScore() to assess the impact of releasing one 
          or multiple constraints
                    lhs op                 rhs      mi    epc sepc.lv sepc.all
275  rcope_ben_total_w1 ~~  rcope_sup_total_w1 289.770  0.773   0.773    0.526
245      rs_identity_w1 ~~  rcope_sup_total_w1  79.095 -0.125  -0.125   -0.263
253           prayer_w1 ~~  rcope_ben_total_w1  58.316 -0.403  -0.403   -0.201
242      rs_identity_w1 ~~       attendance_w1  56.511  0.150   0.150    0.143
146           RS_Eng_W2 =~ chronic_w1_reversed  46.846 -0.213  -0.213   -0.122
134           RS_Eng_W1 =~ chronic_w1_reversed  44.444 -0.194  -0.194   -0.112
262       attendance_w1 ~~  rcope_sup_total_w1  43.389 -0.407  -0.407   -0.179
244      rs_identity_w1 ~~  rcope_ben_total_w1  42.724 -0.105  -0.105   -0.155
300  rcope_ben_total_w2 ~~  rcope_sup_total_w2  33.006  0.429   0.429    0.292
107               SRHW1 =~       attendance_w1  25.298  0.225   0.225    0.080
241      rs_identity_w1 ~~           prayer_w1  24.794  0.075   0.075    0.116
122               SRHW2 =~       attendance_w1  20.231  0.246   0.246    0.087
268       commitment_w1 ~~  rcope_ben_total_w1  17.649 -0.124  -0.124   -0.101
249      rs_identity_w1 ~~  rcope_ben_total_w2  12.639 -0.098  -0.098   -0.145
158        srh_item1_w1 ~~        srh_item1_w2  12.495 -0.072  -0.072   -0.717
108               SRHW1 =~       commitment_w1  12.094  0.063   0.063    0.055
194 chronic_w1_reversed ~~  rcope_ben_total_w1  11.902  0.166   0.166    0.075
188 chronic_w1_reversed ~~        srh_item1_w2  11.495  0.106   0.106    0.218
110               SRHW1 =~  rcope_sup_total_w1  10.223 -0.095  -0.095   -0.045
243      rs_identity_w1 ~~       commitment_w1  10.151  0.026   0.026    0.066
178        srh_item2_w1 ~~       attendance_w1   9.969  0.070   0.070    0.057
179        srh_item2_w1 ~~       commitment_w1   9.766  0.028   0.028    0.061
133           RS_Eng_W1 =~        srh_item2_w1   9.582  0.034   0.034    0.053
145           RS_Eng_W2 =~        srh_item2_w1   9.070  0.035   0.035    0.055
173        srh_item2_w1 ~~ chronic_w1_reversed   8.135 -0.050  -0.050   -0.059
233 chronic_w2_reversed ~~  rcope_ben_total_w1   7.930  0.231   0.231    0.111
190 chronic_w1_reversed ~~      rs_identity_w1   7.768 -0.036  -0.036   -0.050
125               SRHW2 =~  rcope_sup_total_w1   7.378 -0.101  -0.101   -0.048
176        srh_item2_w1 ~~      rs_identity_w1   7.129  0.013   0.013    0.051
26            RS_Eng_W2 ~~           RS_Eng_W2   7.003  0.123   1.000    1.000
25            RS_Eng_W1 ~~           RS_Eng_W1   7.002 -0.123  -1.000   -1.000
123               SRHW2 =~       commitment_w1   6.987  0.060   0.060    0.052
144           RS_Eng_W2 =~        srh_item1_w1   6.726  0.045   0.045    0.063
121               SRHW2 =~           prayer_w1   6.582 -0.103  -0.103   -0.042
225        srh_item2_w2 ~~       attendance_w2   6.534  0.097   0.097    0.078
106               SRHW1 =~           prayer_w1   6.362 -0.082  -0.082   -0.033
248      rs_identity_w1 ~~       commitment_w2   6.200  0.039   0.039    0.098
148           RS_Eng_W2 =~        srh_item2_w2   5.957  0.053   0.053    0.082
114               SRHW1 =~       commitment_w2   5.920  0.093   0.093    0.082
165        srh_item1_w1 ~~  rcope_ben_total_w1   5.819 -0.046  -0.046   -0.099
132           RS_Eng_W1 =~        srh_item1_w1   5.798  0.039   0.039    0.054
293           prayer_w2 ~~  rcope_ben_total_w2   5.751 -0.203  -0.203   -0.101
164        srh_item1_w1 ~~       commitment_w1   5.493  0.022   0.022    0.082
136           RS_Eng_W1 =~        srh_item2_w2   4.983  0.049   0.049    0.076
285  rcope_sup_total_w1 ~~  rcope_ben_total_w2   4.887 -0.192  -0.192   -0.131
198 chronic_w1_reversed ~~       attendance_w2   4.884 -0.204  -0.204   -0.059
181        srh_item2_w1 ~~  rcope_sup_total_w1   4.863 -0.032  -0.032   -0.058
282  rcope_sup_total_w1 ~~           prayer_w2   4.812  0.180   0.180    0.129
156        srh_item1_w1 ~~        srh_item2_w1   4.693  0.023   0.023    0.131
261       attendance_w1 ~~  rcope_ben_total_w1   4.593 -0.156  -0.156   -0.048
183        srh_item2_w1 ~~           prayer_w2   4.431  0.059   0.059    0.078
191 chronic_w1_reversed ~~           prayer_w1   4.417 -0.087  -0.087   -0.041
269       commitment_w1 ~~  rcope_sup_total_w1   4.034 -0.051  -0.051   -0.060
169        srh_item1_w1 ~~       attendance_w2   4.020  0.079   0.079    0.111
232 chronic_w2_reversed ~~       commitment_w1   4.015 -0.082  -0.082   -0.067
163        srh_item1_w1 ~~       attendance_w1   3.935  0.046   0.046    0.064
224        srh_item2_w2 ~~           prayer_w2   3.931 -0.055  -0.055   -0.073
214        srh_item1_w2 ~~  rcope_ben_total_w2   3.883 -0.064  -0.064   -0.138
160        srh_item1_w1 ~~ chronic_w2_reversed   3.791  0.058   0.058    0.126
221        srh_item2_w2 ~~  rcope_ben_total_w1   3.591 -0.068  -0.068   -0.085
254           prayer_w1 ~~  rcope_sup_total_w1   3.573 -0.093  -0.093   -0.066
150           RS_Eng_W2 =~      rs_identity_w1   3.507 -0.035  -0.035   -0.054
265       attendance_w1 ~~       commitment_w2   3.463  0.121   0.121    0.064
184        srh_item2_w1 ~~       attendance_w2   3.281 -0.069  -0.069   -0.056
172        srh_item1_w1 ~~  rcope_sup_total_w2   3.275 -0.048  -0.048   -0.149
116               SRHW1 =~  rcope_sup_total_w2   3.108 -0.100  -0.100   -0.047
212        srh_item1_w2 ~~       attendance_w2   3.098 -0.069  -0.069   -0.096
290      rs_identity_w2 ~~  rcope_sup_total_w2   3.017  0.038   0.038    0.080
180        srh_item2_w1 ~~  rcope_ben_total_w1   3.007 -0.031  -0.031   -0.039
287      rs_identity_w2 ~~       attendance_w2   2.938  0.057   0.057    0.054
129               SRHW2 =~       commitment_w2   2.917  0.065   0.065    0.057
299       commitment_w2 ~~  rcope_sup_total_w2   2.793 -0.072  -0.072   -0.084
202        srh_item1_w2 ~~        srh_item2_w2   2.751  0.019   0.019    0.109
294           prayer_w2 ~~  rcope_sup_total_w2   2.594 -0.110  -0.110   -0.079
154           RS_Eng_W2 =~  rcope_ben_total_w1   2.555 -0.113  -0.113   -0.055
138           RS_Eng_W1 =~      rs_identity_w2   2.510  0.031   0.031    0.047
170        srh_item1_w1 ~~       commitment_w2   2.435  0.029   0.029    0.106
259           prayer_w1 ~~  rcope_sup_total_w2   2.400  0.114   0.114    0.082
141           RS_Eng_W1 =~       commitment_w2   2.352  0.060   0.060    0.052
105               SRHW1 =~      rs_identity_w1   2.189  0.015   0.015    0.022
208        srh_item1_w2 ~~  rcope_ben_total_w1   2.091  0.053   0.053    0.115
211        srh_item1_w2 ~~           prayer_w2   2.036  0.042   0.042    0.095
193 chronic_w1_reversed ~~       commitment_w1   1.964 -0.034  -0.034   -0.026
280  rcope_ben_total_w1 ~~  rcope_sup_total_w2   1.962 -0.122  -0.122   -0.083
292           prayer_w2 ~~       commitment_w2   1.930  0.066   0.066    0.057
119               SRHW2 =~ chronic_w1_reversed   1.904  0.071   0.071    0.040
218        srh_item2_w2 ~~           prayer_w1   1.893  0.039   0.039    0.051
286      rs_identity_w2 ~~           prayer_w2   1.862  0.033   0.033    0.052
252           prayer_w1 ~~       commitment_w1   1.839  0.037   0.037    0.032
161        srh_item1_w1 ~~      rs_identity_w1   1.758  0.007   0.007    0.046
203        srh_item1_w2 ~~ chronic_w2_reversed   1.756 -0.041  -0.041   -0.089
140           RS_Eng_W1 =~       attendance_w2   1.746  0.107   0.107    0.038
228        srh_item2_w2 ~~  rcope_sup_total_w2   1.631  0.032   0.032    0.058
251           prayer_w1 ~~       attendance_w1   1.579  0.083   0.083    0.027
166        srh_item1_w1 ~~  rcope_sup_total_w1   1.388 -0.018  -0.018   -0.057
109               SRHW1 =~  rcope_ben_total_w1   1.352 -0.042  -0.042   -0.020
187        srh_item2_w1 ~~  rcope_sup_total_w2   1.295 -0.029  -0.029   -0.052
137           RS_Eng_W1 =~ chronic_w2_reversed   1.283 -0.058  -0.058   -0.035
229 chronic_w2_reversed ~~      rs_identity_w1   1.261 -0.023  -0.023   -0.035
266       attendance_w1 ~~  rcope_ben_total_w2   1.223  0.128   0.128    0.039
239 chronic_w2_reversed ~~  rcope_ben_total_w2   1.218  0.080   0.080    0.038
120               SRHW2 =~      rs_identity_w1   1.213  0.013   0.013    0.021
276  rcope_ben_total_w1 ~~      rs_identity_w2   1.212 -0.034  -0.034   -0.051
130               SRHW2 =~  rcope_ben_total_w2   1.200 -0.075  -0.075   -0.037
246      rs_identity_w1 ~~           prayer_w2   1.130  0.027   0.027    0.041
216        srh_item2_w2 ~~ chronic_w2_reversed   1.112 -0.026  -0.026   -0.033
205        srh_item1_w2 ~~           prayer_w1   1.107 -0.031  -0.031   -0.072
186        srh_item2_w1 ~~  rcope_ben_total_w2   1.092 -0.033  -0.033   -0.042
238 chronic_w2_reversed ~~       commitment_w2   1.091 -0.042  -0.042   -0.035
147           RS_Eng_W2 =~        srh_item1_w2   1.034 -0.032  -0.032   -0.044
250      rs_identity_w1 ~~  rcope_sup_total_w2   0.980 -0.022  -0.022   -0.047
210        srh_item1_w2 ~~      rs_identity_w2   0.946 -0.009  -0.009   -0.061
185        srh_item2_w1 ~~       commitment_w2   0.943  0.017   0.017    0.037
278  rcope_ben_total_w1 ~~       attendance_w2   0.915 -0.126  -0.126   -0.039
149           RS_Eng_W2 =~ chronic_w2_reversed   0.890 -0.048  -0.048   -0.029
281  rcope_sup_total_w1 ~~      rs_identity_w2   0.884 -0.024  -0.024   -0.050
197 chronic_w1_reversed ~~           prayer_w2   0.883 -0.064  -0.064   -0.030
274       commitment_w1 ~~  rcope_sup_total_w2   0.869  0.041   0.041    0.048
273       commitment_w1 ~~  rcope_ben_total_w2   0.851 -0.050  -0.050   -0.041
272       commitment_w1 ~~       attendance_w2   0.827  0.060   0.060    0.031
117               SRHW2 =~        srh_item1_w1   0.773 -0.022  -0.022   -0.031
104               SRHW1 =~ chronic_w2_reversed   0.734  0.045   0.045    0.027
258           prayer_w1 ~~  rcope_ben_total_w2   0.722  0.074   0.074    0.037
174        srh_item2_w1 ~~        srh_item1_w2   0.671 -0.010  -0.010   -0.058
152           RS_Eng_W2 =~       attendance_w1   0.670 -0.063  -0.063   -0.022
112               SRHW1 =~           prayer_w2   0.649  0.051   0.051    0.021
219        srh_item2_w2 ~~       attendance_w1   0.642 -0.031  -0.031   -0.025
127               SRHW2 =~           prayer_w2   0.610  0.050   0.050    0.020
195 chronic_w1_reversed ~~  rcope_sup_total_w1   0.603 -0.030  -0.030   -0.019
128               SRHW2 =~       attendance_w2   0.577 -0.063  -0.063   -0.022
167        srh_item1_w1 ~~      rs_identity_w2   0.540  0.007   0.007    0.046
171        srh_item1_w1 ~~  rcope_ben_total_w2   0.536  0.024   0.024    0.052
135           RS_Eng_W1 =~        srh_item1_w2   0.534 -0.023  -0.023   -0.032
118               SRHW2 =~        srh_item2_w1   0.500 -0.016  -0.016   -0.024
227        srh_item2_w2 ~~  rcope_ben_total_w2   0.469  0.022   0.022    0.027
113               SRHW1 =~       attendance_w2   0.463 -0.057  -0.057   -0.020
256           prayer_w1 ~~       attendance_w2   0.458 -0.072  -0.072   -0.023
209        srh_item1_w2 ~~  rcope_sup_total_w1   0.429  0.019   0.019    0.060
215        srh_item1_w2 ~~  rcope_sup_total_w2   0.406  0.017   0.017    0.052
226        srh_item2_w2 ~~       commitment_w2   0.398  0.011   0.011    0.024
207        srh_item1_w2 ~~       commitment_w1   0.346 -0.011  -0.011   -0.040
220        srh_item2_w2 ~~       commitment_w1   0.333  0.010   0.010    0.022
240 chronic_w2_reversed ~~  rcope_sup_total_w2   0.324  0.033   0.033    0.022
230 chronic_w2_reversed ~~           prayer_w1   0.321 -0.037  -0.037   -0.018
297       attendance_w2 ~~  rcope_sup_total_w2   0.307 -0.051  -0.051   -0.022
206        srh_item1_w2 ~~       attendance_w1   0.307  0.022   0.022    0.030
260       attendance_w1 ~~       commitment_w1   0.305  0.020   0.020    0.011
289      rs_identity_w2 ~~  rcope_ben_total_w2   0.300 -0.015  -0.015   -0.022
115               SRHW1 =~  rcope_ben_total_w2   0.280 -0.036  -0.036   -0.018
298       commitment_w2 ~~  rcope_ben_total_w2   0.262 -0.027  -0.027   -0.022
284  rcope_sup_total_w1 ~~       commitment_w2   0.255 -0.025  -0.025   -0.029
162        srh_item1_w1 ~~           prayer_w1   0.243 -0.008  -0.008   -0.019
23                SRHW1 ~~               SRHW1   0.230 -0.031  -1.000   -1.000
24                SRHW2 ~~               SRHW2   0.230  0.031   1.000    1.000
277  rcope_ben_total_w1 ~~           prayer_w2   0.209 -0.045  -0.045   -0.022
155           RS_Eng_W2 =~  rcope_sup_total_w1   0.203 -0.025  -0.025   -0.012
111               SRHW1 =~      rs_identity_w2   0.187  0.009   0.009    0.013
222        srh_item2_w2 ~~  rcope_sup_total_w1   0.180 -0.012  -0.012   -0.022
177        srh_item2_w1 ~~           prayer_w1   0.173 -0.007  -0.007   -0.009
159        srh_item1_w1 ~~        srh_item2_w2   0.163 -0.005  -0.005   -0.028
126               SRHW2 =~      rs_identity_w2   0.152 -0.008  -0.008   -0.012
270       commitment_w1 ~~      rs_identity_w2   0.144 -0.006  -0.006   -0.015
103               SRHW1 =~        srh_item2_w2   0.142  0.009   0.009    0.014
142           RS_Eng_W1 =~  rcope_ben_total_w2   0.130 -0.026  -0.026   -0.013
182        srh_item2_w1 ~~      rs_identity_w2   0.128  0.003   0.003    0.013
264       attendance_w1 ~~           prayer_w2   0.120 -0.036  -0.036   -0.012
267       attendance_w1 ~~  rcope_sup_total_w2   0.114 -0.032  -0.032   -0.014
151           RS_Eng_W2 =~           prayer_w1   0.114  0.020   0.020    0.008
102               SRHW1 =~        srh_item1_w2   0.104 -0.008  -0.008   -0.011
192 chronic_w1_reversed ~~       attendance_w1   0.101  0.019   0.019    0.005
279  rcope_ben_total_w1 ~~       commitment_w2   0.098 -0.019  -0.019   -0.016
295       attendance_w2 ~~       commitment_w2   0.092 -0.020  -0.020   -0.010
124               SRHW2 =~  rcope_ben_total_w1   0.083 -0.013  -0.013   -0.006
235 chronic_w2_reversed ~~      rs_identity_w2   0.082 -0.006  -0.006   -0.009
271       commitment_w1 ~~           prayer_w2   0.080 -0.014  -0.014   -0.012
139           RS_Eng_W1 =~           prayer_w2   0.078  0.017   0.017    0.007
291           prayer_w2 ~~       attendance_w2   0.072  0.028   0.028    0.009
288      rs_identity_w2 ~~       commitment_w2   0.071  0.004   0.004    0.010
236 chronic_w2_reversed ~~           prayer_w2   0.067 -0.017  -0.017   -0.008
157        srh_item1_w1 ~~ chronic_w1_reversed   0.065  0.007   0.007    0.015
175        srh_item2_w1 ~~ chronic_w2_reversed   0.056  0.006   0.006    0.007
234 chronic_w2_reversed ~~  rcope_sup_total_w1   0.056  0.015   0.015    0.011
196 chronic_w1_reversed ~~      rs_identity_w2   0.051  0.005   0.005    0.007
237 chronic_w2_reversed ~~       attendance_w2   0.045 -0.019  -0.019   -0.006
255           prayer_w1 ~~      rs_identity_w2   0.041  0.005   0.005    0.008
200 chronic_w1_reversed ~~  rcope_ben_total_w2   0.035 -0.014  -0.014   -0.006
131               SRHW2 =~  rcope_sup_total_w2   0.035 -0.011  -0.011   -0.005
143           RS_Eng_W1 =~  rcope_sup_total_w2   0.034  0.010   0.010    0.005
189 chronic_w1_reversed ~~        srh_item2_w2   0.034 -0.005  -0.005   -0.006
153           RS_Eng_W2 =~       commitment_w1   0.027 -0.006  -0.006   -0.005
231 chronic_w2_reversed ~~       attendance_w1   0.023  0.013   0.013    0.004
263       attendance_w1 ~~      rs_identity_w2   0.023  0.005   0.005    0.005
204        srh_item1_w2 ~~      rs_identity_w1   0.020  0.001   0.001    0.009
257           prayer_w1 ~~       commitment_w2   0.019 -0.007  -0.007   -0.006
217        srh_item2_w2 ~~      rs_identity_w1   0.006  0.001   0.001    0.003
201 chronic_w1_reversed ~~  rcope_sup_total_w2   0.004 -0.004  -0.004   -0.003
213        srh_item1_w2 ~~       commitment_w2   0.004  0.001   0.001    0.004
168        srh_item1_w1 ~~           prayer_w2   0.003 -0.001  -0.001   -0.003
296       attendance_w2 ~~  rcope_ben_total_w2   0.002  0.005   0.005    0.002
283  rcope_sup_total_w1 ~~       attendance_w2   0.001  0.004   0.004    0.002
223        srh_item2_w2 ~~      rs_identity_w2   0.001  0.000   0.000   -0.001
199 chronic_w1_reversed ~~       commitment_w2   0.000  0.000   0.000    0.000
247      rs_identity_w1 ~~       attendance_w2   0.000  0.000   0.000    0.000
    sepc.nox
275    0.526
245   -0.263
253   -0.201
242    0.143
146   -0.122
134   -0.112
262   -0.179
244   -0.155
300    0.292
107    0.080
241    0.116
122    0.087
268   -0.101
249   -0.145
158   -0.717
108    0.055
194    0.075
188    0.218
110   -0.045
243    0.066
178    0.057
179    0.061
133    0.053
145    0.055
173   -0.059
233    0.111
190   -0.050
125   -0.048
176    0.051
26     1.000
25    -1.000
123    0.052
144    0.063
121   -0.042
225    0.078
106   -0.033
248    0.098
148    0.082
114    0.082
165   -0.099
132    0.054
293   -0.101
164    0.082
136    0.076
285   -0.131
198   -0.059
181   -0.058
282    0.129
156    0.131
261   -0.048
183    0.078
191   -0.041
269   -0.060
169    0.111
232   -0.067
163    0.064
224   -0.073
214   -0.138
160    0.126
221   -0.085
254   -0.066
150   -0.054
265    0.064
184   -0.056
172   -0.149
116   -0.047
212   -0.096
290    0.080
180   -0.039
287    0.054
129    0.057
299   -0.084
202    0.109
294   -0.079
154   -0.055
138    0.047
170    0.106
259    0.082
141    0.052
105    0.022
208    0.115
211    0.095
193   -0.026
280   -0.083
292    0.057
119    0.040
218    0.051
286    0.052
252    0.032
161    0.046
203   -0.089
140    0.038
228    0.058
251    0.027
166   -0.057
109   -0.020
187   -0.052
137   -0.035
229   -0.035
266    0.039
239    0.038
120    0.021
276   -0.051
130   -0.037
246    0.041
216   -0.033
205   -0.072
186   -0.042
238   -0.035
147   -0.044
250   -0.047
210   -0.061
185    0.037
278   -0.039
149   -0.029
281   -0.050
197   -0.030
274    0.048
273   -0.041
272    0.031
117   -0.031
104    0.027
258    0.037
174   -0.058
152   -0.022
112    0.021
219   -0.025
127    0.020
195   -0.019
128   -0.022
167    0.046
171    0.052
135   -0.032
118   -0.024
227    0.027
113   -0.020
256   -0.023
209    0.060
215    0.052
226    0.024
207   -0.040
220    0.022
240    0.022
230   -0.018
297   -0.022
206    0.030
260    0.011
289   -0.022
115   -0.018
298   -0.022
284   -0.029
162   -0.019
23    -1.000
24     1.000
277   -0.022
155   -0.012
111    0.013
222   -0.022
177   -0.009
159   -0.028
126   -0.012
270   -0.015
103    0.014
142   -0.013
182    0.013
264   -0.012
267   -0.014
151    0.008
102   -0.011
192    0.005
279   -0.016
295   -0.010
124   -0.006
235   -0.009
271   -0.012
139    0.007
291    0.009
288    0.010
236   -0.008
157    0.015
175    0.007
234    0.011
196    0.007
237   -0.006
255    0.008
200   -0.006
131   -0.005
143    0.005
189   -0.006
153   -0.005
231    0.004
263    0.005
204    0.009
257   -0.006
217    0.003
201   -0.003
213    0.004
168   -0.003
296    0.002
283    0.002
223   -0.001
199    0.000
247    0.000
```

- Back to the Table of Contents

## Structural Equation Models

Add covariates in the model, and regress them on all indicators. The
following lines of code assess one of the primary research questions
(i.e., “does R/S Engagement at Wave 1 predict SRH at Wave 2, and does
SRH at Wave 1 predict R/S Engagement at Wave 2?”):  

SRHW2~RS\_Eng\_W1  

RS\_Eng\_W2~SRHW1

```
structuralmodel <- "SRHW1 =~ NA*L1*srh_item1_w1 + L2*srh_item2_w1 +
                      L3*chronic_w1_reversed
SRHW2 =~ NA*L1*srh_item1_w2 + L2*srh_item2_w2 + L3*chronic_w2_reversed
RS_Eng_W1 =~ NA*L4*rs_identity_w1 + L5*prayer_w1 + L6*attendance_w1 +
    L7*commitment_w1 + L8*rcope_ben_total_w1 + L9*rcope_sup_total_w1
RS_Eng_W2 =~ NA*L4*rs_identity_w2 + L5*prayer_w2 + L6*attendance_w2 +
    L7*commitment_w2 + L8*rcope_ben_total_w2 + L9*rcope_sup_total_w2
SRHW1~0
SRHW2~NA*0
RS_Eng_W1~0
RS_Eng_W2~NA*0
SRHW1~~1*SRHW1
SRHW2~~1*SRHW2
RS_Eng_W1~~1*RS_Eng_W1
RS_Eng_W2~~1*RS_Eng_W2
srh_item2_w1~~srh_item2_w2
chronic_w1_reversed~~chronic_w2_reversed
rs_identity_w1~~rs_identity_w2
prayer_w1~~prayer_w2
attendance_w1~~attendance_w2
commitment_w1~~commitment_w2
rcope_ben_total_w1~~rcope_ben_total_w2
rcope_sup_total_w1~~rcope_sup_total_w2
srh_item1_w1~i1*1
srh_item1_w2~i1*1
srh_item2_w1~i2*1
srh_item2_w2~i2*1
rs_identity_w1~i4*1
rs_identity_w2~i4*1
prayer_w1~i5*1
prayer_w2~i5*1
attendance_w1~i6*1
attendance_w2~i6*1
commitment_w1~i7*1
commitment_w2~i7*1
rcope_ben_total_w1~i8*1
rcope_ben_total_w2~i8*1
rcope_sup_total_w1~i9*1
rcope_sup_total_w2~i9*1
srh_item1_w1~~e1*srh_item1_w1
srh_item1_w2~~e1*srh_item1_w2
srh_item2_w1~~e2*srh_item2_w1
srh_item2_w2~~e2*srh_item2_w2
rs_identity_w1~~e4*rs_identity_w1
rs_identity_w2~~e4*rs_identity_w2
prayer_w1~~e5*prayer_w1
prayer_w2~~e5*prayer_w2
attendance_w1~~e6*attendance_w1
attendance_w2~~e6*attendance_w2
commitment_w1~~e7*commitment_w1
commitment_w2~~e7*commitment_w2
rcope_ben_total_w1~~e8*rcope_ben_total_w1
rcope_ben_total_w2~~e8*rcope_ben_total_w2
rcope_sup_total_w1~~e9*rcope_sup_total_w1
rcope_sup_total_w2~~e9*rcope_sup_total_w2
RS_Eng_W1~~SRHW1
RS_Eng_W2~~SRHW2
SRHW2~RS_Eng_W1
RS_Eng_W2~SRHW1
SRHW2~SRHW1
RS_Eng_W2~RS_Eng_W1
age_w1_divided_by_10~~is_male
age_w1_divided_by_10~~education
age_w1_divided_by_10~~income
age_w1_divided_by_10~~is_black
age_w1_divided_by_10~~is_hispanic
is_male~~education
is_male~~income
is_male~~is_black
is_male~~is_hispanic
education~~income
education~~is_black
education~~is_hispanic
srh_item1_w1~age_w1_divided_by_10+is_male+income+education+is_black+is_hispanic
srh_item2_w1~age_w1_divided_by_10+is_male+income+education+is_black+is_hispanic
chronic_w1_reversed~age_w1_divided_by_10+is_male+income+education+is_black+
  is_hispanic
srh_item1_w2~age_w1_divided_by_10+is_male+income+education+is_black+is_hispanic
srh_item2_w2~age_w1_divided_by_10+is_male+income+education+is_black+is_hispanic
chronic_w2_reversed~age_w1_divided_by_10+is_male+income+education+is_black+
  is_hispanic
rs_identity_w1~age_w1_divided_by_10+is_male+income+education+is_black+
  is_hispanic
prayer_w1~age_w1_divided_by_10+is_male+income+education+is_black+is_hispanic
attendance_w1~age_w1_divided_by_10+is_male+income+education+is_black+is_hispanic
commitment_w1~age_w1_divided_by_10+is_male+income+education+is_black+is_hispanic
rcope_ben_total_w1~age_w1_divided_by_10+is_male+income+education+is_black+
  is_hispanic
rcope_sup_total_w1~age_w1_divided_by_10+is_male+income+education+is_black+
  is_hispanic
rs_identity_w2~age_w1_divided_by_10+is_male+income+education+is_black+
  is_hispanic
prayer_w2~age_w1_divided_by_10+is_male+income+education+is_black+is_hispanic
attendance_w2~age_w1_divided_by_10+is_male+income+education+is_black+is_hispanic
commitment_w2~age_w1_divided_by_10+is_male+income+education+is_black+is_hispanic
rcope_ben_total_w2~age_w1_divided_by_10+is_male+income+education+is_black+
  is_hispanic
rcope_sup_total_w2~age_w1_divided_by_10+is_male+income+education+is_black+
  is_hispanic
"

# Fitting model and running summary statistics

fitstructuralmodel <- cfa(data = which_comes_first_data,
                          model = structuralmodel, estimator = "MLR",
                          missing = "ML", std.lv = T, meanstructure = T)
summary(fitstructuralmodel, fit.measures = TRUE, standardized = TRUE, ci = TRUE)
lavaan 0.6.17 ended normally after 321 iterations

  Estimator                                         ML
  Optimization method                           NLMINB
  Number of model parameters                       202
  Number of equality constraints                    25

  Number of observations                          3010
  Number of missing patterns                        94

Model Test User Model:
                                              Standard      Scaled
  Test Statistic                              1094.342    1002.298
  Degrees of freedom                               147         147
  P-value (Chi-square)                           0.000       0.000
  Scaling correction factor                                  1.092
    Yuan-Bentler correction (Mplus variant)                       

Model Test Baseline Model:

  Test statistic                             16610.877   15356.633
  Degrees of freedom                               276         276
  P-value                                        0.000       0.000
  Scaling correction factor                                  1.082

User Model versus Baseline Model:

  Comparative Fit Index (CFI)                    0.942       0.943
  Tucker-Lewis Index (TLI)                       0.891       0.894
                                                                  
  Robust Comparative Fit Index (CFI)                         0.931
  Robust Tucker-Lewis Index (TLI)                            0.870

Loglikelihood and Information Criteria:

  Loglikelihood user model (H0)             -70347.154  -70347.154
  Scaling correction factor                                  0.906
      for the MLR correction                                      
  Loglikelihood unrestricted model (H1)     -69799.983  -69799.983
  Scaling correction factor                                  1.060
      for the MLR correction                                      
                                                                  
  Akaike (AIC)                              141048.308  141048.308
  Bayesian (BIC)                            142112.024  142112.024
  Sample-size adjusted Bayesian (SABIC)     141549.626  141549.626

Root Mean Square Error of Approximation:

  RMSEA                                          0.046       0.044
  90 Percent confidence interval - lower         0.044       0.042
  90 Percent confidence interval - upper         0.049       0.046
  P-value H_0: RMSEA <= 0.050                    0.991       1.000
  P-value H_0: RMSEA >= 0.080                    0.000       0.000
                                                                  
  Robust RMSEA                                               0.075
  90 Percent confidence interval - lower                     0.069
  90 Percent confidence interval - upper                     0.081
  P-value H_0: Robust RMSEA <= 0.050                         0.000
  P-value H_0: Robust RMSEA >= 0.080                         0.084

Standardized Root Mean Square Residual:

  SRMR                                           0.074       0.074

Parameter Estimates:

  Standard errors                             Sandwich
  Information bread                           Observed
  Observed information based on                Hessian

Latent Variables:
                   Estimate  Std.Err  z-value  P(>|z|) ci.lower ci.upper
  SRHW1 =~                                                              
    srh_tm1_1 (L1)    0.524    0.016   33.011    0.000    0.493    0.555
    srh_tm2_1 (L2)    0.365    0.013   27.763    0.000    0.339    0.391
    chrnc_w1_ (L3)    0.756    0.033   22.855    0.000    0.692    0.821
  SRHW2 =~                                                              
    srh_tm1_2 (L1)    0.524    0.016   33.011    0.000    0.493    0.555
    srh_tm2_2 (L2)    0.365    0.013   27.763    0.000    0.339    0.391
    chrnc_w2_ (L3)    0.756    0.033   22.855    0.000    0.692    0.821
  RS_Eng_W1 =~                                                          
    rs_dntt_1 (L4)    0.399    0.010   39.969    0.000    0.380    0.419
    prayer_w1 (L5)    1.702    0.034   49.592    0.000    1.634    1.769
    attndnc_1 (L6)    1.477    0.037   39.697    0.000    1.404    1.550
    cmmtmnt_1 (L7)    0.681    0.020   34.192    0.000    0.642    0.720
    rcp_bn__1 (L8)    1.283    0.030   42.814    0.000    1.224    1.341
    rcp_sp__1 (L9)    1.614    0.029   55.812    0.000    1.557    1.671
  RS_Eng_W2 =~                                                          
    rs_dntt_2 (L4)    0.399    0.010   39.969    0.000    0.380    0.419
    prayer_w2 (L5)    1.702    0.034   49.592    0.000    1.634    1.769
    attndnc_2 (L6)    1.477    0.037   39.697    0.000    1.404    1.550
    cmmtmnt_2 (L7)    0.681    0.020   34.192    0.000    0.642    0.720
    rcp_bn__2 (L8)    1.283    0.030   42.814    0.000    1.224    1.341
    rcp_sp__2 (L9)    1.614    0.029   55.812    0.000    1.557    1.671
   Std.lv  Std.all
                  
    0.524    0.744
    0.365    0.577
    0.756    0.434
                  
    0.651    0.801
    0.454    0.662
    0.941    0.523
                  
    0.399    0.632
    1.702    0.729
    1.477    0.536
    0.681    0.609
    1.283    0.646
    1.614    0.795
                  
    0.537    0.735
    2.287    0.816
    1.985    0.634
    0.915    0.717
    1.724    0.757
    2.169    0.871

Regressions:
                        Estimate  Std.Err  z-value  P(>|z|) ci.lower ci.upper
  SRHW2 ~                                                                    
    RS_Eng_W1              0.087    0.039    2.225    0.026    0.010    0.164
  RS_Eng_W2 ~                                                                
    SRHW1                  0.031    0.026    1.215    0.224   -0.019    0.082
  SRHW2 ~                                                                    
    SRHW1                  0.733    0.041   17.681    0.000    0.652    0.815
  RS_Eng_W2 ~                                                                
    RS_Eng_W1              0.896    0.021   43.325    0.000    0.856    0.937
  srh_item1_w1 ~                                                             
    ag_w1_dvdd__10        -0.042    0.007   -6.221    0.000   -0.056   -0.029
    is_male               -0.037    0.025   -1.452    0.146   -0.087    0.013
    income                 0.048    0.005    9.947    0.000    0.039    0.057
    education              0.024    0.005    4.687    0.000    0.014    0.033
    is_black              -0.111    0.039   -2.854    0.004   -0.188   -0.035
    is_hispanic           -0.094    0.040   -2.370    0.018   -0.171   -0.016
  srh_item2_w1 ~                                                             
    ag_w1_dvdd__10         0.078    0.006   13.500    0.000    0.066    0.089
    is_male                0.050    0.023    2.203    0.028    0.006    0.095
    income                 0.026    0.004    5.859    0.000    0.017    0.034
    education              0.013    0.004    3.053    0.002    0.005    0.022
    is_black               0.057    0.034    1.688    0.091   -0.009    0.124
    is_hispanic            0.106    0.033    3.249    0.001    0.042    0.171
  chronic_w1_reversed ~                                                      
    ag_w1_dvdd__10        -0.472    0.014  -33.429    0.000   -0.499   -0.444
    is_male                0.115    0.054    2.120    0.034    0.009    0.221
    income                 0.109    0.010   10.838    0.000    0.089    0.128
    education              0.021    0.010    2.004    0.045    0.000    0.041
    is_black              -0.016    0.082   -0.195    0.845   -0.176    0.144
    is_hispanic            0.223    0.077    2.904    0.004    0.072    0.373
  srh_item1_w2 ~                                                             
    ag_w1_dvdd__10        -0.080    0.014   -5.713    0.000   -0.108   -0.053
    is_male               -0.042    0.050   -0.835    0.404   -0.140    0.056
    income                 0.050    0.009    5.270    0.000    0.031    0.069
    education              0.024    0.009    2.582    0.010    0.006    0.042
    is_black              -0.062    0.082   -0.758    0.449   -0.222    0.098
    is_hispanic           -0.015    0.079   -0.189    0.850   -0.171    0.141
  srh_item2_w2 ~                                                             
    ag_w1_dvdd__10         0.055    0.012    4.544    0.000    0.031    0.079
    is_male                0.020    0.046    0.442    0.658   -0.070    0.110
    income                 0.035    0.009    3.982    0.000    0.018    0.052
    education              0.012    0.007    1.621    0.105   -0.003    0.026
    is_black               0.056    0.078    0.723    0.470   -0.096    0.208
    is_hispanic           -0.010    0.072   -0.142    0.887   -0.152    0.132
  chronic_w2_reversed ~                                                      
    ag_w1_dvdd__10        -0.502    0.028  -17.897    0.000   -0.557   -0.447
    is_male                0.141    0.101    1.391    0.164   -0.058    0.339
    income                 0.096    0.018    5.235    0.000    0.060    0.133
    education             -0.013    0.020   -0.666    0.506   -0.053    0.026
    is_black               0.072    0.151    0.474    0.636   -0.224    0.367
    is_hispanic            0.062    0.159    0.390    0.697   -0.249    0.373
  rs_identity_w1 ~                                                           
    ag_w1_dvdd__10         0.063    0.006    9.712    0.000    0.050    0.075
    is_male               -0.186    0.024   -7.780    0.000   -0.233   -0.139
    income                 0.001    0.004    0.279    0.781   -0.007    0.010
    education             -0.007    0.005   -1.501    0.133   -0.016    0.002
    is_black               0.134    0.034    3.983    0.000    0.068    0.200
    is_hispanic            0.044    0.033    1.331    0.183   -0.021    0.110
  prayer_w1 ~                                                                
    ag_w1_dvdd__10         0.234    0.024    9.745    0.000    0.187    0.281
    is_male               -1.023    0.089  -11.547    0.000   -1.197   -0.850
    income                -0.017    0.016   -1.054    0.292   -0.048    0.015
    education             -0.054    0.016   -3.321    0.001   -0.086   -0.022
    is_black               1.042    0.107    9.773    0.000    0.833    1.251
    is_hispanic            0.358    0.122    2.936    0.003    0.119    0.597
  attendance_w1 ~                                                            
    ag_w1_dvdd__10         0.255    0.027    9.461    0.000    0.202    0.308
    is_male               -0.729    0.101   -7.209    0.000   -0.927   -0.531
    income                 0.053    0.019    2.785    0.005    0.016    0.090
    education              0.023    0.019    1.212    0.226   -0.014    0.059
    is_black               1.072    0.142    7.536    0.000    0.793    1.351
    is_hispanic            0.667    0.144    4.620    0.000    0.384    0.950
  commitment_w1 ~                                                            
    ag_w1_dvdd__10         0.118    0.011   10.455    0.000    0.096    0.140
    is_male               -0.254    0.042   -6.053    0.000   -0.336   -0.171
    income                 0.011    0.008    1.401    0.161   -0.004    0.027
    education             -0.006    0.007   -0.844    0.399   -0.021    0.008
    is_black               0.436    0.057    7.613    0.000    0.324    0.548
    is_hispanic            0.233    0.058    4.008    0.000    0.119    0.347
  rcope_ben_total_w1 ~                                                       
    ag_w1_dvdd__10         0.014    0.022    0.659    0.510   -0.028    0.057
    is_male               -0.590    0.081   -7.264    0.000   -0.749   -0.431
    income                -0.013    0.016   -0.837    0.403   -0.044    0.018
    education             -0.071    0.015   -4.662    0.000   -0.101   -0.041
    is_black               0.958    0.114    8.419    0.000    0.735    1.181
    is_hispanic            0.594    0.119    4.982    0.000    0.361    0.828
  rcope_sup_total_w1 ~                                                       
    ag_w1_dvdd__10         0.157    0.022    7.099    0.000    0.114    0.201
    is_male               -0.804    0.082   -9.849    0.000   -0.965   -0.644
    income                -0.029    0.015   -1.978    0.048   -0.058   -0.000
    education             -0.067    0.015   -4.457    0.000   -0.097   -0.038
    is_black               1.308    0.095   13.739    0.000    1.121    1.495
    is_hispanic            0.575    0.112    5.111    0.000    0.354    0.795
  rs_identity_w2 ~                                                           
    ag_w1_dvdd__10         0.061    0.011    5.445    0.000    0.039    0.083
    is_male               -0.218    0.043   -5.072    0.000   -0.303   -0.134
    income                 0.004    0.009    0.462    0.644   -0.013    0.021
    education             -0.007    0.007   -1.037    0.300   -0.020    0.006
    is_black               0.205    0.061    3.388    0.001    0.086    0.324
    is_hispanic            0.100    0.064    1.550    0.121   -0.026    0.226
  prayer_w2 ~                                                                
    ag_w1_dvdd__10         0.233    0.038    6.075    0.000    0.158    0.309
    is_male               -1.040    0.146   -7.125    0.000   -1.327   -0.754
    income                -0.008    0.028   -0.292    0.771   -0.062    0.046
    education             -0.061    0.024   -2.524    0.012   -0.108   -0.014
    is_black               1.189    0.236    5.045    0.000    0.727    1.652
    is_hispanic            0.653    0.219    2.980    0.003    0.223    1.082
  attendance_w2 ~                                                            
    ag_w1_dvdd__10         0.422    0.047    8.994    0.000    0.330    0.514
    is_male               -0.903    0.182   -4.957    0.000   -1.260   -0.546
    income                 0.041    0.035    1.177    0.239   -0.027    0.110
    education             -0.027    0.029   -0.931    0.352   -0.084    0.030
    is_black               0.923    0.335    2.754    0.006    0.266    1.580
    is_hispanic            0.076    0.336    0.226    0.821   -0.583    0.734
  commitment_w2 ~                                                            
    ag_w1_dvdd__10         0.109    0.020    5.413    0.000    0.069    0.148
    is_male               -0.306    0.080   -3.847    0.000   -0.462   -0.150
    income                 0.011    0.016    0.682    0.495   -0.021    0.043
    education              0.000    0.012    0.016    0.987   -0.024    0.025
    is_black               0.393    0.135    2.906    0.004    0.128    0.658
    is_hispanic            0.407    0.114    3.564    0.000    0.183    0.631
  rcope_ben_total_w2 ~                                                       
    ag_w1_dvdd__10         0.036    0.039    0.920    0.357   -0.041    0.113
    is_male               -0.674    0.149   -4.519    0.000   -0.967   -0.382
    income                -0.006    0.030   -0.209    0.835   -0.065    0.052
    education             -0.065    0.023   -2.822    0.005   -0.111   -0.020
    is_black               0.711    0.278    2.558    0.011    0.166    1.255
    is_hispanic            0.233    0.271    0.861    0.389   -0.298    0.763
  rcope_sup_total_w2 ~                                                       
    ag_w1_dvdd__10         0.161    0.037    4.398    0.000    0.089    0.232
    is_male               -0.927    0.133   -6.978    0.000   -1.188   -0.667
    income                 0.044    0.023    1.901    0.057   -0.001    0.090
    education             -0.097    0.023   -4.300    0.000   -0.141   -0.053
    is_black               1.055    0.217    4.856    0.000    0.629    1.481
    is_hispanic            0.444    0.220    2.015    0.044    0.012    0.876
   Std.lv  Std.all
                  
    0.070    0.070
                  
    0.023    0.023
                  
    0.590    0.590
                  
    0.667    0.667
                  
   -0.042   -0.116
   -0.037   -0.026
    0.048    0.221
    0.024    0.104
   -0.111   -0.055
   -0.094   -0.048
                  
    0.078    0.237
    0.050    0.039
    0.026    0.131
    0.013    0.065
    0.057    0.031
    0.106    0.061
                  
   -0.472   -0.522
    0.115    0.033
    0.109    0.202
    0.021    0.037
   -0.016   -0.003
    0.223    0.046
                  
   -0.080   -0.191
   -0.042   -0.025
    0.050    0.199
    0.024    0.091
   -0.062   -0.026
   -0.015   -0.007
                  
    0.055    0.155
    0.020    0.015
    0.035    0.164
    0.012    0.054
    0.056    0.028
   -0.010   -0.005
                  
   -0.502   -0.538
    0.141    0.039
    0.096    0.174
   -0.013   -0.023
    0.072    0.014
    0.062    0.013
                  
    0.063    0.191
   -0.186   -0.145
    0.001    0.006
   -0.007   -0.033
    0.134    0.073
    0.044    0.025
                  
    0.234    0.193
   -1.023   -0.217
   -0.017   -0.023
   -0.054   -0.072
    1.042    0.154
    0.358    0.056
                  
    0.255    0.178
   -0.729   -0.131
    0.053    0.062
    0.023    0.026
    1.072    0.134
    0.667    0.088
                  
    0.118    0.204
   -0.254   -0.112
    0.011    0.032
   -0.006   -0.018
    0.436    0.135
    0.233    0.076
                  
    0.014    0.014
   -0.590   -0.147
   -0.013   -0.021
   -0.071   -0.111
    0.958    0.166
    0.594    0.109
                  
    0.157    0.150
   -0.804   -0.196
   -0.029   -0.047
   -0.067   -0.103
    1.308    0.222
    0.575    0.103
                  
    0.061    0.162
   -0.218   -0.148
    0.004    0.018
   -0.007   -0.030
    0.205    0.097
    0.100    0.050
                  
    0.233    0.161
   -1.040   -0.184
   -0.008   -0.009
   -0.061   -0.068
    1.189    0.146
    0.653    0.085
                  
    0.422    0.260
   -0.903   -0.143
    0.041    0.043
   -0.027   -0.027
    0.923    0.102
    0.076    0.009
                  
    0.109    0.164
   -0.306   -0.119
    0.011    0.028
    0.000    0.000
    0.393    0.106
    0.407    0.116
                  
    0.036    0.031
   -0.674   -0.147
   -0.006   -0.009
   -0.065   -0.090
    0.711    0.108
    0.233    0.037
                  
    0.161    0.125
   -0.927   -0.184
    0.044    0.058
   -0.097   -0.122
    1.055    0.146
    0.444    0.065

Covariances:
                          Estimate  Std.Err  z-value  P(>|z|) ci.lower ci.upper
 .srh_item2_w1 ~~                                                              
   .srh_item2_w2             0.087    0.012    7.453    0.000    0.064    0.109
 .chronic_w1_reversed ~~                                                       
   .chrnc_w2_rvrsd           0.673    0.073    9.195    0.000    0.529    0.816
 .rs_identity_w1 ~~                                                            
   .rs_identity_w2           0.098    0.012    8.469    0.000    0.075    0.121
 .prayer_w1 ~~                                                                 
   .prayer_w2                0.895    0.121    7.406    0.000    0.658    1.131
 .attendance_w1 ~~                                                             
   .attendance_w2            3.042    0.202   15.059    0.000    2.646    3.438
 .commitment_w1 ~~                                                             
   .commitment_w2            0.122    0.038    3.171    0.002    0.047    0.197
 .rcope_ben_total_w1 ~~                                                        
   .rcop_bn_ttl_w2           0.367    0.113    3.254    0.001    0.146    0.587
 .rcope_sup_total_w1 ~~                                                        
   .rcop_sp_ttl_w2           0.150    0.096    1.557    0.120   -0.039    0.338
  SRHW1 ~~                                                                     
    RS_Eng_W1                0.014    0.021    0.676    0.499   -0.027    0.055
 .SRHW2 ~~                                                                     
   .RS_Eng_W2                0.293    0.176    1.665    0.096   -0.052    0.637
  age_w1_divided_by_10 ~~                                                      
    is_male                 -0.036    0.018   -2.043    0.041   -0.070   -0.001
    education               -0.118    0.108   -1.097    0.273   -0.330    0.093
    income                   0.402    0.119    3.380    0.001    0.169    0.635
    is_black                -0.095    0.012   -7.707    0.000   -0.120   -0.071
    is_hispanic             -0.143    0.013  -10.750    0.000   -0.169   -0.117
  is_male ~~                                                                   
    education                0.020    0.028    0.730    0.466   -0.034    0.075
    income                   0.139    0.031    4.502    0.000    0.078    0.199
    is_black                -0.002    0.003   -0.475    0.635   -0.008    0.005
    is_hispanic              0.001    0.003    0.436    0.663   -0.005    0.008
  income ~~                                                                    
    education                4.651    0.189   24.582    0.000    4.280    5.022
  education ~~                                                                 
    is_black                -0.057    0.016   -3.511    0.000   -0.088   -0.025
    is_hispanic             -0.213    0.023   -9.222    0.000   -0.258   -0.168
   Std.lv  Std.all
                  
    0.087    0.371
                  
    0.673    0.477
                  
    0.098    0.455
                  
    0.895    0.462
                  
    3.042    0.619
                  
    0.122    0.174
                  
    0.367    0.186
                  
    0.150    0.154
                  
    0.014    0.014
                  
    0.293    0.293
                  
   -0.036   -0.037
   -0.118   -0.020
    0.402    0.064
   -0.095   -0.143
   -0.143   -0.204
                  
    0.020    0.013
    0.139    0.087
   -0.002   -0.009
    0.001    0.008
                  
    4.651    0.461
                  
   -0.057   -0.053
   -0.213   -0.188

Intercepts:
                   Estimate  Std.Err  z-value  P(>|z|) ci.lower ci.upper
    SRHW1             0.000                               0.000    0.000
   .SRHW2             0.216    0.274    0.790    0.430   -0.320    0.753
    RS_Eng_W1         0.000                               0.000    0.000
   .RS_Eng_W2        -0.130    0.175   -0.739    0.460   -0.474    0.214
   .srh_tm1_1 (i1)    1.562    0.077   20.354    0.000    1.411    1.712
   .srh_tm1_2 (i1)    1.562    0.077   20.354    0.000    1.411    1.712
   .srh_tm2_1 (i2)    0.559    0.065    8.646    0.000    0.432    0.686
   .srh_tm2_2 (i2)    0.559    0.065    8.646    0.000    0.432    0.686
   .rs_dntt_1 (i4)    1.271    0.071   17.952    0.000    1.132    1.410
   .rs_dntt_2 (i4)    1.271    0.071   17.952    0.000    1.132    1.410
   .prayer_w1 (i5)    4.857    0.259   18.783    0.000    4.351    5.364
   .prayer_w2 (i5)    4.857    0.259   18.783    0.000    4.351    5.364
   .attndnc_1 (i6)    1.839    0.282    6.520    0.000    1.286    2.392
   .attndnc_2 (i6)    1.839    0.282    6.520    0.000    1.286    2.392
   .cmmtmnt_1 (i7)    2.353    0.118   19.895    0.000    2.121    2.585
   .cmmtmnt_2 (i7)    2.353    0.118   19.895    0.000    2.121    2.585
   .rcp_bn__1 (i8)    3.802    0.229   16.586    0.000    3.353    4.252
   .rcp_bn__2 (i8)    3.802    0.229   16.586    0.000    3.353    4.252
   .rcp_sp__1 (i9)    4.364    0.238   18.341    0.000    3.898    4.831
   .rcp_sp__2 (i9)    4.364    0.238   18.341    0.000    3.898    4.831
   .chrnc_w1_         9.745    0.153   63.561    0.000    9.445   10.046
   .chrnc_w2_         9.997    0.318   31.425    0.000    9.373   10.620
    ag_1___10         5.172    0.035  146.388    0.000    5.103    5.241
    is_male           0.428    0.009   47.417    0.000    0.410    0.445
    income            6.786    0.061  111.982    0.000    6.667    6.905
    education        13.468    0.058  233.980    0.000   13.355   13.581
    is_black          0.138    0.006   21.855    0.000    0.126    0.151
    is_hispnc         0.156    0.007   23.552    0.000    0.143    0.169
   Std.lv  Std.all
    0.000    0.000
    0.174    0.174
    0.000    0.000
   -0.096   -0.096
    1.562    2.220
    1.562    1.920
    0.559    0.882
    0.559    0.815
    1.271    2.010
    1.271    1.740
    4.857    2.081
    4.857    1.734
    1.839    0.667
    1.839    0.588
    2.353    2.106
    2.353    1.845
    3.802    1.915
    3.802    1.670
    4.364    2.149
    4.364    1.752
    9.745    5.588
    9.997    5.556
    5.172    2.679
    0.428    0.864
    6.786    2.096
   13.468    4.319
    0.138    0.401
    0.156    0.430

Variances:
                   Estimate  Std.Err  z-value  P(>|z|) ci.lower ci.upper
    SRHW1             1.000                               1.000    1.000
   .SRHW2             1.000                               1.000    1.000
    RS_Eng_W1         1.000                               1.000    1.000
   .RS_Eng_W2         1.000                               1.000    1.000
   .srh_tm1_1 (e1)    0.174    0.014   12.676    0.000    0.147    0.201
   .srh_tm1_2 (e1)    0.174    0.014   12.676    0.000    0.147    0.201
   .srh_tm2_1 (e2)    0.234    0.008   29.084    0.000    0.218    0.249
   .srh_tm2_2 (e2)    0.234    0.008   29.084    0.000    0.218    0.249
   .rs_dntt_1 (e4)    0.215    0.006   33.225    0.000    0.203    0.228
   .rs_dntt_2 (e4)    0.215    0.006   33.225    0.000    0.203    0.228
   .prayer_w1 (e5)    1.934    0.083   23.204    0.000    1.771    2.098
   .prayer_w2 (e5)    1.934    0.083   23.204    0.000    1.771    2.098
   .attndnc_1 (e6)    4.912    0.119   41.387    0.000    4.680    5.145
   .attndnc_2 (e6)    4.912    0.119   41.387    0.000    4.680    5.145
   .cmmtmnt_1 (e7)    0.701    0.024   29.173    0.000    0.654    0.748
   .cmmtmnt_2 (e7)    0.701    0.024   29.173    0.000    0.654    0.748
   .rcp_bn__1 (e8)    1.971    0.065   30.234    0.000    1.844    2.099
   .rcp_bn__2 (e8)    1.971    0.065   30.234    0.000    1.844    2.099
   .rcp_sp__1 (e9)    0.972    0.057   17.151    0.000    0.860    1.083
   .rcp_sp__2 (e9)    0.972    0.057   17.151    0.000    0.860    1.083
   .chrnc_w1_         1.487    0.059   24.987    0.000    1.370    1.603
   .chrnc_w2_         1.337    0.107   12.448    0.000    1.126    1.547
    ag_1___10         3.727    0.065   57.437    0.000    3.600    3.855
    is_male           0.245    0.001  185.492    0.000    0.242    0.247
    income           10.485    0.201   52.138    0.000   10.091   10.879
    education         9.726    0.307   31.656    0.000    9.123   10.328
    is_black          0.119    0.005   26.004    0.000    0.110    0.128
    is_hispnc         0.132    0.005   28.891    0.000    0.123    0.141
   Std.lv  Std.all
    1.000    1.000
    0.646    0.646
    1.000    1.000
    0.554    0.554
    0.174    0.351
    0.174    0.263
    0.234    0.582
    0.234    0.496
    0.215    0.539
    0.215    0.404
    1.934    0.355
    1.934    0.246
    4.912    0.645
    4.912    0.502
    0.701    0.561
    0.701    0.430
    1.971    0.500
    1.971    0.380
    0.972    0.236
    0.972    0.157
    1.487    0.489
    1.337    0.413
    3.727    1.000
    0.245    1.000
   10.485    1.000
    9.726    1.000
    0.119    1.000
    0.132    1.000


# Look under Latent Variables (and Std.all) for standardized factor loadings and
# under Variances (and Std.Err) for measurement error terms seen in S1 Table.
```

- Back to the Table of Contents

### Strength of Associations

Constrain the regression coefficient between R/S Engagement at Wave 1
to SRH at Wave 2 to be equal to the coefficient between SRH at Wave 1 to
R/S Engagement at Wave 2 (see \*\*“c1\*” **before**
“RS\_Eng\_W1” **and** “c1SRHW2”\*\*).

```
strength_effects <- "SRHW1 =~ NA*L1*srh_item1_w1 + L2*srh_item2_w1 +
                        L3*chronic_w1_reversed
SRHW2 =~ NA*L1*srh_item1_w2 + L2*srh_item2_w2 + L3*chronic_w2_reversed
RS_Eng_W1 =~ NA*L4*rs_identity_w1 + L5*prayer_w1 + L6*attendance_w1 +
    L7*commitment_w1 + L8*rcope_ben_total_w1 + L9*rcope_sup_total_w1
RS_Eng_W2 =~ NA*L4*rs_identity_w2 + L5*prayer_w2 + L6*attendance_w2 +
    L7*commitment_w2 + L8*rcope_ben_total_w2 + L9*rcope_sup_total_w2
SRHW1~0
SRHW2~NA*0
RS_Eng_W1~0
RS_Eng_W2~NA*0
SRHW1~~1*SRHW1
SRHW2~~1*SRHW2
RS_Eng_W1~~1*RS_Eng_W1
RS_Eng_W2~~1*RS_Eng_W2
srh_item2_w1~~srh_item2_w2
chronic_w1_reversed~~chronic_w2_reversed
rs_identity_w1~~rs_identity_w2
prayer_w1~~prayer_w2
attendance_w1~~attendance_w2
commitment_w1~~commitment_w2
rcope_ben_total_w1~~rcope_ben_total_w2
rcope_sup_total_w1~~rcope_sup_total_w2
srh_item1_w1~i1*1
srh_item1_w2~i1*1
srh_item2_w1~i2*1
srh_item2_w2~i2*1
rs_identity_w1~i4*1
rs_identity_w2~i4*1
prayer_w1~i5*1
prayer_w2~i5*1
attendance_w1~i6*1
attendance_w2~i6*1
commitment_w1~i7*1
commitment_w2~i7*1
rcope_ben_total_w1~i8*1
rcope_ben_total_w2~i8*1
rcope_sup_total_w1~i9*1
rcope_sup_total_w2~i9*1
srh_item1_w1~~e1*srh_item1_w1
srh_item1_w2~~e1*srh_item1_w2
srh_item2_w1~~e2*srh_item2_w1
srh_item2_w2~~e2*srh_item2_w2
rs_identity_w1~~e4*rs_identity_w1
rs_identity_w2~~e4*rs_identity_w2
prayer_w1~~e5*prayer_w1
prayer_w2~~e5*prayer_w2
attendance_w1~~e6*attendance_w1
attendance_w2~~e6*attendance_w2
commitment_w1~~e7*commitment_w1
commitment_w2~~e7*commitment_w2
rcope_ben_total_w1~~e8*rcope_ben_total_w1
rcope_ben_total_w2~~e8*rcope_ben_total_w2
rcope_sup_total_w1~~e9*rcope_sup_total_w1
rcope_sup_total_w2~~e9*rcope_sup_total_w2
RS_Eng_W1~~SRHW1
RS_Eng_W2~~SRHW2
SRHW2~c1*RS_Eng_W1
RS_Eng_W2~c1*SRHW1
SRHW2~SRHW1
RS_Eng_W2~RS_Eng_W1
age_w1_divided_by_10~~is_male
age_w1_divided_by_10~~education
age_w1_divided_by_10~~income
age_w1_divided_by_10~~is_black
age_w1_divided_by_10~~is_hispanic
is_male~~education
is_male~~income
is_male~~is_black
is_male~~is_hispanic
education~~income
education~~is_black
education~~is_hispanic
srh_item1_w1~age_w1_divided_by_10+is_male+income+education+is_black+is_hispanic
srh_item2_w1~age_w1_divided_by_10+is_male+income+education+is_black+is_hispanic
chronic_w1_reversed~age_w1_divided_by_10+is_male+income+education+is_black+
  is_hispanic
srh_item1_w2~age_w1_divided_by_10+is_male+income+education+is_black+is_hispanic
srh_item2_w2~age_w1_divided_by_10+is_male+income+education+is_black+is_hispanic
chronic_w2_reversed~age_w1_divided_by_10+is_male+income+education+is_black+
  is_hispanic
rs_identity_w1~age_w1_divided_by_10+is_male+income+education+is_black+
  is_hispanic
prayer_w1~age_w1_divided_by_10+is_male+income+education+is_black+is_hispanic
attendance_w1~age_w1_divided_by_10+is_male+income+education+is_black+is_hispanic
commitment_w1~age_w1_divided_by_10+is_male+income+education+is_black+is_hispanic
rcope_ben_total_w1~age_w1_divided_by_10+is_male+income+education+is_black+
  is_hispanic
rcope_sup_total_w1~age_w1_divided_by_10+is_male+income+education+is_black+
  is_hispanic
rs_identity_w2~age_w1_divided_by_10+is_male+income+education+is_black+
  is_hispanic
prayer_w2~age_w1_divided_by_10+is_male+income+education+is_black+is_hispanic
attendance_w2~age_w1_divided_by_10+is_male+income+education+is_black+is_hispanic
commitment_w2~age_w1_divided_by_10+is_male+income+education+is_black+is_hispanic
rcope_ben_total_w2~age_w1_divided_by_10+is_male+income+education+is_black+
  is_hispanic
rcope_sup_total_w2~age_w1_divided_by_10+is_male+income+education+is_black+
  is_hispanic
"

# Fitting model and running summary statistics

fitstrength_effects <- cfa(data = which_comes_first_data,
                           model = strength_effects, estimator = "MLR",
                           missing = "ML", std.lv = T, meanstructure = T)
summary(fitstrength_effects, fit.measures = TRUE, standardized = TRUE, ci = TRUE)
lavaan 0.6.17 ended normally after 321 iterations

  Estimator                                         ML
  Optimization method                           NLMINB
  Number of model parameters                       202
  Number of equality constraints                    26

  Number of observations                          3010
  Number of missing patterns                        94

Model Test User Model:
                                              Standard      Scaled
  Test Statistic                              1095.067    1006.730
  Degrees of freedom                               148         148
  P-value (Chi-square)                           0.000       0.000
  Scaling correction factor                                  1.088
    Yuan-Bentler correction (Mplus variant)                       

Model Test Baseline Model:

  Test statistic                             16610.877   15356.633
  Degrees of freedom                               276         276
  P-value                                        0.000       0.000
  Scaling correction factor                                  1.082

User Model versus Baseline Model:

  Comparative Fit Index (CFI)                    0.942       0.943
  Tucker-Lewis Index (TLI)                       0.892       0.894
                                                                  
  Robust Comparative Fit Index (CFI)                         0.931
  Robust Tucker-Lewis Index (TLI)                            0.871

Loglikelihood and Information Criteria:

  Loglikelihood user model (H0)             -70347.517  -70347.517
  Scaling correction factor                                  0.903
      for the MLR correction                                      
  Loglikelihood unrestricted model (H1)     -69799.983  -69799.983
  Scaling correction factor                                  1.060
      for the MLR correction                                      
                                                                  
  Akaike (AIC)                              141047.034  141047.034
  Bayesian (BIC)                            142104.740  142104.740
  Sample-size adjusted Bayesian (SABIC)     141545.519  141545.519

Root Mean Square Error of Approximation:

  RMSEA                                          0.046       0.044
  90 Percent confidence interval - lower         0.044       0.041
  90 Percent confidence interval - upper         0.049       0.046
  P-value H_0: RMSEA <= 0.050                    0.994       1.000
  P-value H_0: RMSEA >= 0.080                    0.000       0.000
                                                                  
  Robust RMSEA                                               0.075
  90 Percent confidence interval - lower                     0.069
  90 Percent confidence interval - upper                     0.081
  P-value H_0: Robust RMSEA <= 0.050                         0.000
  P-value H_0: Robust RMSEA >= 0.080                         0.072

Standardized Root Mean Square Residual:

  SRMR                                           0.073       0.073

Parameter Estimates:

  Standard errors                             Sandwich
  Information bread                           Observed
  Observed information based on                Hessian

Latent Variables:
                   Estimate  Std.Err  z-value  P(>|z|) ci.lower ci.upper
  SRHW1 =~                                                              
    srh_tm1_1 (L1)    0.524    0.016   33.041    0.000    0.493    0.555
    srh_tm2_1 (L2)    0.365    0.013   27.717    0.000    0.339    0.391
    chrnc_w1_ (L3)    0.756    0.033   22.838    0.000    0.691    0.821
  SRHW2 =~                                                              
    srh_tm1_2 (L1)    0.524    0.016   33.041    0.000    0.493    0.555
    srh_tm2_2 (L2)    0.365    0.013   27.717    0.000    0.339    0.391
    chrnc_w2_ (L3)    0.756    0.033   22.838    0.000    0.691    0.821
  RS_Eng_W1 =~                                                          
    rs_dntt_1 (L4)    0.399    0.010   39.956    0.000    0.380    0.419
    prayer_w1 (L5)    1.701    0.034   49.592    0.000    1.634    1.769
    attndnc_1 (L6)    1.477    0.037   39.677    0.000    1.404    1.550
    cmmtmnt_1 (L7)    0.681    0.020   34.182    0.000    0.642    0.720
    rcp_bn__1 (L8)    1.282    0.030   42.815    0.000    1.224    1.341
    rcp_sp__1 (L9)    1.613    0.029   55.838    0.000    1.557    1.670
  RS_Eng_W2 =~                                                          
    rs_dntt_2 (L4)    0.399    0.010   39.956    0.000    0.380    0.419
    prayer_w2 (L5)    1.701    0.034   49.592    0.000    1.634    1.769
    attndnc_2 (L6)    1.477    0.037   39.677    0.000    1.404    1.550
    cmmtmnt_2 (L7)    0.681    0.020   34.182    0.000    0.642    0.720
    rcp_bn__2 (L8)    1.282    0.030   42.815    0.000    1.224    1.341
    rcp_sp__2 (L9)    1.613    0.029   55.838    0.000    1.557    1.670
   Std.lv  Std.all
                  
    0.524    0.745
    0.365    0.577
    0.756    0.434
                  
    0.653    0.802
    0.455    0.663
    0.942    0.524
                  
    0.399    0.632
    1.701    0.729
    1.477    0.535
    0.681    0.609
    1.282    0.646
    1.613    0.795
                  
    0.536    0.735
    2.283    0.816
    1.982    0.634
    0.914    0.717
    1.721    0.757
    2.165    0.870

Regressions:
                        Estimate  Std.Err  z-value  P(>|z|) ci.lower ci.upper
  SRHW2 ~                                                                    
    RS_Eng_W1 (c1)         0.061    0.024    2.498    0.012    0.013    0.108
  RS_Eng_W2 ~                                                                
    SRHW1     (c1)         0.061    0.024    2.498    0.012    0.013    0.108
  SRHW2 ~                                                                    
    SRHW1                  0.739    0.042   17.466    0.000    0.656    0.822
  RS_Eng_W2 ~                                                                
    RS_Eng_W1              0.892    0.021   42.050    0.000    0.850    0.934
  srh_item1_w1 ~                                                             
    ag_1___10             -0.042    0.007   -6.221    0.000   -0.056   -0.029
    is_male               -0.037    0.025   -1.452    0.146   -0.087    0.013
    income                 0.048    0.005    9.946    0.000    0.039    0.057
    education              0.024    0.005    4.688    0.000    0.014    0.033
    is_black              -0.111    0.039   -2.854    0.004   -0.188   -0.035
    is_hispnc             -0.094    0.040   -2.370    0.018   -0.171   -0.016
  srh_item2_w1 ~                                                             
    ag_1___10              0.078    0.006   13.500    0.000    0.066    0.089
    is_male                0.050    0.023    2.203    0.028    0.006    0.095
    income                 0.026    0.004    5.859    0.000    0.017    0.034
    education              0.013    0.004    3.052    0.002    0.005    0.022
    is_black               0.057    0.034    1.687    0.092   -0.009    0.124
    is_hispnc              0.106    0.033    3.248    0.001    0.042    0.171
  chronic_w1_reversed ~                                                      
    ag_1___10             -0.472    0.014  -33.428    0.000   -0.499   -0.444
    is_male                0.115    0.054    2.120    0.034    0.009    0.221
    income                 0.109    0.010   10.838    0.000    0.089    0.128
    education              0.021    0.010    2.004    0.045    0.000    0.041
    is_black              -0.016    0.082   -0.195    0.845   -0.176    0.144
    is_hispnc              0.223    0.077    2.904    0.004    0.072    0.373
  srh_item1_w2 ~                                                             
    ag_1___10             -0.080    0.014   -5.687    0.000   -0.108   -0.052
    is_male               -0.042    0.050   -0.840    0.401   -0.140    0.056
    income                 0.050    0.009    5.268    0.000    0.031    0.069
    education              0.024    0.009    2.564    0.010    0.006    0.042
    is_black              -0.059    0.082   -0.724    0.469   -0.220    0.101
    is_hispnc             -0.014    0.079   -0.180    0.857   -0.169    0.141
  srh_item2_w2 ~                                                             
    ag_1___10              0.055    0.012    4.575    0.000    0.032    0.079
    is_male                0.020    0.046    0.437    0.662   -0.070    0.110
    income                 0.035    0.009    3.983    0.000    0.018    0.052
    education              0.012    0.007    1.603    0.109   -0.003    0.026
    is_black               0.058    0.078    0.744    0.457   -0.095    0.210
    is_hispnc             -0.010    0.072   -0.135    0.892   -0.152    0.132
  chronic_w2_reversed ~                                                      
    ag_1___10             -0.501    0.028  -17.888    0.000   -0.556   -0.446
    is_male                0.140    0.101    1.387    0.166   -0.058    0.338
    income                 0.097    0.018    5.238    0.000    0.060    0.133
    education             -0.014    0.020   -0.680    0.496   -0.053    0.026
    is_black               0.075    0.151    0.499    0.618   -0.220    0.371
    is_hispnc              0.063    0.158    0.398    0.690   -0.247    0.373
  rs_identity_w1 ~                                                           
    ag_1___10              0.063    0.006    9.712    0.000    0.050    0.075
    is_male               -0.186    0.024   -7.780    0.000   -0.233   -0.139
    income                 0.001    0.004    0.279    0.781   -0.007    0.010
    education             -0.007    0.005   -1.501    0.133   -0.016    0.002
    is_black               0.134    0.034    3.982    0.000    0.068    0.200
    is_hispnc              0.044    0.033    1.331    0.183   -0.021    0.110
  prayer_w1 ~                                                                
    ag_1___10              0.234    0.024    9.745    0.000    0.187    0.281
    is_male               -1.023    0.089  -11.548    0.000   -1.197   -0.850
    income                -0.017    0.016   -1.054    0.292   -0.048    0.015
    education             -0.054    0.016   -3.322    0.001   -0.086   -0.022
    is_black               1.042    0.107    9.773    0.000    0.833    1.251
    is_hispnc              0.358    0.122    2.936    0.003    0.119    0.597
  attendance_w1 ~                                                            
    ag_1___10              0.255    0.027    9.461    0.000    0.202    0.308
    is_male               -0.729    0.101   -7.209    0.000   -0.927   -0.531
    income                 0.053    0.019    2.785    0.005    0.016    0.090
    education              0.023    0.019    1.211    0.226   -0.014    0.059
    is_black               1.072    0.142    7.536    0.000    0.793    1.351
    is_hispnc              0.667    0.144    4.620    0.000    0.384    0.950
  commitment_w1 ~                                                            
    ag_1___10              0.118    0.011   10.455    0.000    0.096    0.140
    is_male               -0.254    0.042   -6.053    0.000   -0.336   -0.172
    income                 0.011    0.008    1.402    0.161   -0.004    0.027
    education             -0.006    0.007   -0.845    0.398   -0.021    0.008
    is_black               0.436    0.057    7.613    0.000    0.324    0.548
    is_hispnc              0.233    0.058    4.008    0.000    0.119    0.347
  rcope_ben_total_w1 ~                                                       
    ag_1___10              0.014    0.022    0.657    0.511   -0.029    0.057
    is_male               -0.590    0.081   -7.264    0.000   -0.749   -0.431
    income                -0.013    0.016   -0.837    0.403   -0.044    0.018
    education             -0.071    0.015   -4.662    0.000   -0.101   -0.041
    is_black               0.958    0.114    8.418    0.000    0.735    1.181
    is_hispnc              0.594    0.119    4.981    0.000    0.360    0.828
  rcope_sup_total_w1 ~                                                       
    ag_1___10              0.157    0.022    7.097    0.000    0.114    0.201
    is_male               -0.804    0.082   -9.849    0.000   -0.965   -0.644
    income                -0.029    0.015   -1.977    0.048   -0.058   -0.000
    education             -0.067    0.015   -4.458    0.000   -0.097   -0.038
    is_black               1.308    0.095   13.739    0.000    1.121    1.495
    is_hispnc              0.574    0.112    5.109    0.000    0.354    0.795
  rs_identity_w2 ~                                                           
    ag_1___10              0.061    0.011    5.409    0.000    0.039    0.083
    is_male               -0.217    0.043   -5.052    0.000   -0.302   -0.133
    income                 0.004    0.009    0.463    0.643   -0.013    0.021
    education             -0.007    0.007   -1.040    0.298   -0.020    0.006
    is_black               0.207    0.060    3.432    0.001    0.089    0.325
    is_hispnc              0.100    0.065    1.548    0.122   -0.027    0.227
  prayer_w2 ~                                                                
    ag_1___10              0.231    0.038    6.016    0.000    0.156    0.307
    is_male               -1.036    0.146   -7.092    0.000   -1.322   -0.749
    income                -0.008    0.028   -0.292    0.771   -0.062    0.046
    education             -0.061    0.024   -2.528    0.011   -0.109   -0.014
    is_black               1.197    0.235    5.095    0.000    0.736    1.657
    is_hispnc              0.653    0.219    2.985    0.003    0.224    1.082
  attendance_w2 ~                                                            
    ag_1___10              0.420    0.047    8.947    0.000    0.328    0.512
    is_male               -0.899    0.182   -4.935    0.000   -1.256   -0.542
    income                 0.041    0.035    1.177    0.239   -0.027    0.110
    education             -0.027    0.029   -0.934    0.350   -0.084    0.030
    is_black               0.929    0.335    2.778    0.005    0.274    1.585
    is_hispnc              0.076    0.336    0.228    0.820   -0.581    0.734
  commitment_w2 ~                                                            
    ag_1___10              0.108    0.020    5.385    0.000    0.069    0.147
    is_male               -0.304    0.079   -3.826    0.000   -0.460   -0.148
    income                 0.011    0.016    0.685    0.493   -0.021    0.043
    education              0.000    0.012    0.011    0.991   -0.024    0.025
    is_black               0.396    0.135    2.931    0.003    0.131    0.661
    is_hispnc              0.407    0.114    3.565    0.000    0.183    0.631
  rcope_ben_total_w2 ~                                                       
    ag_1___10              0.035    0.039    0.884    0.377   -0.042    0.111
    is_male               -0.671    0.149   -4.500    0.000   -0.963   -0.379
    income                -0.006    0.030   -0.208    0.835   -0.065    0.052
    education             -0.066    0.023   -2.822    0.005   -0.111   -0.020
    is_black               0.716    0.278    2.574    0.010    0.171    1.261
    is_hispnc              0.234    0.270    0.864    0.388   -0.296    0.763
  rcope_sup_total_w2 ~                                                       
    ag_1___10              0.159    0.036    4.354    0.000    0.087    0.230
    is_male               -0.923    0.133   -6.938    0.000   -1.184   -0.662
    income                 0.044    0.023    1.896    0.058   -0.001    0.090
    education             -0.097    0.023   -4.295    0.000   -0.142   -0.053
    is_black               1.062    0.218    4.864    0.000    0.634    1.490
    is_hispnc              0.445    0.221    2.017    0.044    0.012    0.878
   Std.lv  Std.all
                  
    0.049    0.049
                  
    0.045    0.045
                  
    0.593    0.593
                  
    0.665    0.665
                  
   -0.042   -0.116
   -0.037   -0.026
    0.048    0.221
    0.024    0.104
   -0.111   -0.055
   -0.094   -0.048
                  
    0.078    0.237
    0.050    0.039
    0.026    0.131
    0.013    0.065
    0.057    0.031
    0.106    0.061
                  
   -0.472   -0.522
    0.115    0.033
    0.109    0.202
    0.021    0.037
   -0.016   -0.003
    0.223    0.046
                  
   -0.080   -0.190
   -0.042   -0.026
    0.050    0.199
    0.024    0.090
   -0.059   -0.025
   -0.014   -0.006
                  
    0.055    0.156
    0.020    0.014
    0.035    0.164
    0.012    0.054
    0.058    0.029
   -0.010   -0.005
                  
   -0.501   -0.537
    0.140    0.039
    0.097    0.174
   -0.014   -0.024
    0.075    0.014
    0.063    0.013
                  
    0.063    0.191
   -0.186   -0.145
    0.001    0.006
   -0.007   -0.033
    0.134    0.073
    0.044    0.025
                  
    0.234    0.193
   -1.023   -0.217
   -0.017   -0.023
   -0.054   -0.072
    1.042    0.154
    0.358    0.056
                  
    0.255    0.178
   -0.729   -0.131
    0.053    0.062
    0.023    0.026
    1.072    0.134
    0.667    0.088
                  
    0.118    0.204
   -0.254   -0.112
    0.011    0.032
   -0.006   -0.018
    0.436    0.135
    0.233    0.076
                  
    0.014    0.014
   -0.590   -0.147
   -0.013   -0.021
   -0.071   -0.111
    0.958    0.166
    0.594    0.109
                  
    0.157    0.150
   -0.804   -0.196
   -0.029   -0.047
   -0.067   -0.103
    1.308    0.222
    0.574    0.103
                  
    0.061    0.161
   -0.217   -0.147
    0.004    0.018
   -0.007   -0.030
    0.207    0.098
    0.100    0.050
                  
    0.231    0.160
   -1.036   -0.183
   -0.008   -0.009
   -0.061   -0.068
    1.197    0.147
    0.653    0.085
                  
    0.420    0.259
   -0.899   -0.142
    0.041    0.043
   -0.027   -0.027
    0.929    0.103
    0.076    0.009
                  
    0.108    0.163
   -0.304   -0.118
    0.011    0.028
    0.000    0.000
    0.396    0.107
    0.407    0.116
                  
    0.035    0.029
   -0.671   -0.146
   -0.006   -0.009
   -0.066   -0.090
    0.716    0.109
    0.234    0.037
                  
    0.159    0.123
   -0.923   -0.184
    0.044    0.058
   -0.097   -0.122
    1.062    0.147
    0.445    0.065

Covariances:
                          Estimate  Std.Err  z-value  P(>|z|) ci.lower ci.upper
 .srh_item2_w1 ~~                                                              
   .srh_item2_w2             0.087    0.012    7.467    0.000    0.064    0.109
 .chronic_w1_reversed ~~                                                       
   .chrnc_w2_rvrsd           0.673    0.073    9.196    0.000    0.529    0.816
 .rs_identity_w1 ~~                                                            
   .rs_identity_w2           0.098    0.012    8.459    0.000    0.075    0.121
 .prayer_w1 ~~                                                                 
   .prayer_w2                0.895    0.121    7.414    0.000    0.659    1.132
 .attendance_w1 ~~                                                             
   .attendance_w2            3.041    0.202   15.055    0.000    2.645    3.437
 .commitment_w1 ~~                                                             
   .commitment_w2            0.122    0.038    3.172    0.002    0.047    0.197
 .rcope_ben_total_w1 ~~                                                        
   .rcop_bn_ttl_w2           0.366    0.113    3.249    0.001    0.145    0.587
 .rcope_sup_total_w1 ~~                                                        
   .rcop_sp_ttl_w2           0.150    0.096    1.558    0.119   -0.039    0.339
  SRHW1 ~~                                                                     
    RS_Eng_W1                0.015    0.021    0.700    0.484   -0.026    0.055
 .SRHW2 ~~                                                                     
   .RS_Eng_W2                0.291    0.177    1.641    0.101   -0.056    0.638
  age_w1_divided_by_10 ~~                                                      
    is_male                 -0.036    0.018   -2.043    0.041   -0.070   -0.001
    education               -0.118    0.108   -1.097    0.273   -0.330    0.093
    income                   0.402    0.119    3.379    0.001    0.169    0.635
    is_black                -0.095    0.012   -7.707    0.000   -0.120   -0.071
    is_hispanic             -0.143    0.013  -10.750    0.000   -0.169   -0.117
  is_male ~~                                                                   
    education                0.020    0.028    0.730    0.466   -0.034    0.075
    income                   0.139    0.031    4.502    0.000    0.078    0.199
    is_black                -0.002    0.003   -0.476    0.634   -0.008    0.005
    is_hispanic              0.001    0.003    0.436    0.663   -0.005    0.008
  income ~~                                                                    
    education                4.651    0.189   24.582    0.000    4.280    5.022
  education ~~                                                                 
    is_black                -0.057    0.016   -3.511    0.000   -0.088   -0.025
    is_hispanic             -0.213    0.023   -9.222    0.000   -0.258   -0.168
   Std.lv  Std.all
                  
    0.087    0.371
                  
    0.673    0.477
                  
    0.098    0.455
                  
    0.895    0.463
                  
    3.041    0.619
                  
    0.122    0.174
                  
    0.366    0.186
                  
    0.150    0.154
                  
    0.015    0.015
                  
    0.291    0.291
                  
   -0.036   -0.037
   -0.118   -0.020
    0.402    0.064
   -0.095   -0.143
   -0.143   -0.204
                  
    0.020    0.013
    0.139    0.087
   -0.002   -0.009
    0.001    0.008
                  
    4.651    0.461
                  
   -0.057   -0.053
   -0.213   -0.188

Intercepts:
                   Estimate  Std.Err  z-value  P(>|z|) ci.lower ci.upper
    SRHW1             0.000                               0.000    0.000
   .SRHW2             0.216    0.273    0.792    0.428   -0.319    0.752
    RS_Eng_W1         0.000                               0.000    0.000
   .RS_Eng_W2        -0.126    0.175   -0.719    0.472   -0.470    0.218
   .srh_tm1_1 (i1)    1.562    0.077   20.354    0.000    1.411    1.712
   .srh_tm1_2 (i1)    1.562    0.077   20.354    0.000    1.411    1.712
   .srh_tm2_1 (i2)    0.559    0.065    8.647    0.000    0.432    0.686
   .srh_tm2_2 (i2)    0.559    0.065    8.647    0.000    0.432    0.686
   .rs_dntt_1 (i4)    1.271    0.071   17.953    0.000    1.132    1.410
   .rs_dntt_2 (i4)    1.271    0.071   17.953    0.000    1.132    1.410
   .prayer_w1 (i5)    4.858    0.259   18.783    0.000    4.351    5.364
   .prayer_w2 (i5)    4.858    0.259   18.783    0.000    4.351    5.364
   .attndnc_1 (i6)    1.840    0.282    6.521    0.000    1.287    2.392
   .attndnc_2 (i6)    1.840    0.282    6.521    0.000    1.287    2.392
   .cmmtmnt_1 (i7)    2.353    0.118   19.896    0.000    2.121    2.585
   .cmmtmnt_2 (i7)    2.353    0.118   19.896    0.000    2.121    2.585
   .rcp_bn__1 (i8)    3.803    0.229   16.588    0.000    3.353    4.252
   .rcp_bn__2 (i8)    3.803    0.229   16.588    0.000    3.353    4.252
   .rcp_sp__1 (i9)    4.364    0.238   18.341    0.000    3.898    4.831
   .rcp_sp__2 (i9)    4.364    0.238   18.341    0.000    3.898    4.831
   .chrnc_w1_         9.745    0.153   63.560    0.000    9.445   10.046
   .chrnc_w2_         9.997    0.318   31.431    0.000    9.373   10.620
    ag_1___10         5.172    0.035  146.388    0.000    5.103    5.241
    is_male           0.428    0.009   47.417    0.000    0.410    0.445
    income            6.786    0.061  111.982    0.000    6.667    6.905
    education        13.468    0.058  233.980    0.000   13.355   13.581
    is_black          0.138    0.006   21.855    0.000    0.126    0.151
    is_hispnc         0.156    0.007   23.552    0.000    0.143    0.169
   Std.lv  Std.all
    0.000    0.000
    0.174    0.174
    0.000    0.000
   -0.094   -0.094
    1.562    2.219
    1.562    1.919
    0.559    0.882
    0.559    0.814
    1.271    2.011
    1.271    1.742
    4.858    2.082
    4.858    1.736
    1.840    0.667
    1.840    0.588
    2.353    2.106
    2.353    1.846
    3.803    1.915
    3.803    1.672
    4.364    2.149
    4.364    1.755
    9.745    5.587
    9.997    5.556
    5.172    2.679
    0.428    0.864
    6.786    2.096
   13.468    4.319
    0.138    0.401
    0.156    0.430

Variances:
                   Estimate  Std.Err  z-value  P(>|z|) ci.lower ci.upper
    SRHW1             1.000                               1.000    1.000
   .SRHW2             1.000                               1.000    1.000
    RS_Eng_W1         1.000                               1.000    1.000
   .RS_Eng_W2         1.000                               1.000    1.000
   .srh_tm1_1 (e1)    0.173    0.014   12.627    0.000    0.147    0.200
   .srh_tm1_2 (e1)    0.173    0.014   12.627    0.000    0.147    0.200
   .srh_tm2_1 (e2)    0.234    0.008   29.073    0.000    0.218    0.249
   .srh_tm2_2 (e2)    0.234    0.008   29.073    0.000    0.218    0.249
   .rs_dntt_1 (e4)    0.215    0.006   33.228    0.000    0.203    0.228
   .rs_dntt_2 (e4)    0.215    0.006   33.228    0.000    0.203    0.228
   .prayer_w1 (e5)    1.934    0.083   23.208    0.000    1.771    2.097
   .prayer_w2 (e5)    1.934    0.083   23.208    0.000    1.771    2.097
   .attndnc_1 (e6)    4.912    0.119   41.392    0.000    4.680    5.145
   .attndnc_2 (e6)    4.912    0.119   41.392    0.000    4.680    5.145
   .cmmtmnt_1 (e7)    0.701    0.024   29.176    0.000    0.653    0.748
   .cmmtmnt_2 (e7)    0.701    0.024   29.176    0.000    0.653    0.748
   .rcp_bn__1 (e8)    1.972    0.065   30.239    0.000    1.844    2.100
   .rcp_bn__2 (e8)    1.972    0.065   30.239    0.000    1.844    2.100
   .rcp_sp__1 (e9)    0.972    0.057   17.162    0.000    0.861    1.083
   .rcp_sp__2 (e9)    0.972    0.057   17.162    0.000    0.861    1.083
   .chrnc_w1_         1.487    0.060   24.989    0.000    1.371    1.604
   .chrnc_w2_         1.337    0.107   12.444    0.000    1.126    1.547
    ag_1___10         3.727    0.065   57.437    0.000    3.600    3.855
    is_male           0.245    0.001  185.492    0.000    0.242    0.247
    income           10.485    0.201   52.138    0.000   10.090   10.879
    education         9.726    0.307   31.656    0.000    9.123   10.328
    is_black          0.119    0.005   26.004    0.000    0.110    0.128
    is_hispnc         0.132    0.005   28.891    0.000    0.123    0.141
   Std.lv  Std.all
    1.000    1.000
    0.645    0.645
    1.000    1.000
    0.555    0.555
    0.173    0.350
    0.173    0.262
    0.234    0.582
    0.234    0.496
    0.215    0.539
    0.215    0.405
    1.934    0.355
    1.934    0.247
    4.912    0.646
    4.912    0.503
    0.701    0.561
    0.701    0.431
    1.972    0.500
    1.972    0.381
    0.972    0.236
    0.972    0.157
    1.487    0.489
    1.337    0.413
    3.727    1.000
    0.245    1.000
   10.485    1.000
    9.726    1.000
    0.119    1.000
    0.132    1.000


# Comparing fit of the models with and without constrained main effects

anova(fitstrength_effects, fitstructuralmodel)

Scaled Chi-Squared Difference Test (method = "satorra.bentler.2001")

lavaan NOTE:
    The "Chisq" column contains standard test statistics, not the
    robust test that should be reported per model. A robust difference
    test is a function of two standard (not robust) statistics.
 
                     Df    AIC    BIC  Chisq Chisq diff Df diff Pr(>Chisq)
fitstructuralmodel  147 141048 142112 1094.3                              
fitstrength_effects 148 141047 142105 1095.1     1.4891       1     0.2224
```

- Back to the Table of Contents

# Appendix

# Self-Rated Health Item 3

Re-running structural equation models with self-rated health item 3
included (this was removed due to a low factor loading observed in the
measurement model stage). Results did not differ with and without this
item included.

```
# Structural Model

structuralmodel_with_SRH_item3 <- "SRHW1 =~ NA*L1*srh_item1_w1 +
                                      L2*srh_item2_w1 + L3*chronic_w1_reversed +
                                      L10*srh_item3_w1
SRHW2 =~ NA*L1*srh_item1_w2 + L2*srh_item2_w2 + L3*chronic_w2_reversed +
    L10*srh_item3_w2
RS_Eng_W1 =~ NA*L4*rs_identity_w1 + L5*prayer_w1 + L6*attendance_w1 +
    L7*commitment_w1 + L8*rcope_ben_total_w1 + L9*rcope_sup_total_w1
RS_Eng_W2 =~ NA*L4*rs_identity_w2 + L5*prayer_w2 + L6*attendance_w2 +
    L7*commitment_w2 + L8*rcope_ben_total_w2 + L9*rcope_sup_total_w2
SRHW1~0
SRHW2~NA*0
RS_Eng_W1~0
RS_Eng_W2~NA*0
SRHW1~~1*SRHW1
SRHW2~~1*SRHW2
RS_Eng_W1~~1*RS_Eng_W1
RS_Eng_W2~~1*RS_Eng_W2
srh_item2_w1~~srh_item2_w2
srh_item3_w1~~srh_item3_w2
chronic_w1_reversed~~chronic_w2_reversed
rs_identity_w1~~rs_identity_w2
prayer_w1~~prayer_w2
attendance_w1~~attendance_w2
commitment_w1~~commitment_w2
rcope_ben_total_w1~~rcope_ben_total_w2
rcope_sup_total_w1~~rcope_sup_total_w2
srh_item1_w1~i1*1
srh_item1_w2~i1*1
srh_item2_w1~i2*1
srh_item2_w2~i2*1
srh_item3_w1~i10*1
srh_item3_w2~i10*1
[truncated: 668,833 more chars]
