# Supplementary material for: “Which comes first”: Religious/spiritual engagement or health? Initial observations from longitudinal analyses
Source: PLoS One. 2025 May 7;20(5):e0320410. doi: 10.1371/journal.pone.0320410 (PMC12057932; doi:10.1371/journal.pone.0320410)
Supplement: S2 File — (DOCX) [file pone.0320410.s004.docx]

**S2 File. List of Chronic Illnesses.**

Next, I’d like to know if you have experienced any of the following health problems during the past 12 months, that is, since last [INTERVIEWER NOTE: MONTH DESIGNATING THE CURRENT MONTH (IN YEAR) IN WHICH INTERVIEW IS TAKING PLACE]. Have you had [READ EACH ITEM] or not?

1 Arthritis or rheumatism

2 Cataracts, glaucoma, or other eye diseases

3 Asthma, emphysema, chronic bronchitis, tuberculosis, or other respiratory diseases

4 Hypertension, sometimes called high blood pressure, or have you taken medication for it.

5 Heart attack or other heart trouble.

6 Diabetes or high blood sugar, or have you taken medication for it.

7 Ulcers (of the digestive system) or other stomach or intestinal disorders

8 Liver disease

9 Kidney disease

10 Other urinary tract disorders

11 Cancer or malignant tumor of any kind (include melanoma but not other skin cancer)

12 Other major health problem IF YES: SPECIFY:________________
