# Supplementary material for: “Which comes first”: Religious/spiritual engagement or health? Initial observations from longitudinal analyses
Source: PLoS One. 2025 May 7;20(5):e0320410. doi: 10.1371/journal.pone.0320410 (PMC12057932; doi:10.1371/journal.pone.0320410)
Supplement: S3 File — Table 6 has been redacted to prevent disclosure risk to participants. (DOC) [file pone.0320410.s005.doc]

# S3 File. Sampling and Weighting Report. Table 6 has been redacted to prevent disclosure risk to participants.

# Landmark Spirituality and Health Survey Sampling and Weighting Report

# Version 2

Rebecca Wang

Steven Pedlow

February 2015

**Executive Summary**

The Landmark Spirituality and Health Survey is a Nationwide Face-to-Face Survey of 3,000 respondents with this targeted age breakdown: 1,000 aged 18-40; 1,000 aged 41-64; and 1,000 aged 65 and older. This study includes a 70-minute interview in English and Spanish with some basic biomarker data collection.

We used the NORC 2010 National Sampling Frame. A total of 3,010 face-to-face interviews were collected using a representative sample of housing units from the NORC 2010 National Sampling Frame. The actual age breakdown of the 3,010 completed interviews was the following: 1,000 aged 18-40; 1,002 aged 41-64; and 1,008 aged 65 and older.

In this sampling report, we first describe our sampling approach; then we describe the sample releases. Finally, we have a section on weighting.

1. **Selection of First and Second Stage Units**

For the Landmark Spirituality and Health Survey, we used a subset of the 2010 NORC National Frame to achieve an equal probability sample so the resulting data sets can be analyzed with the smallest possible variation in the weights (which will be due mostly to non-response). Achieving a small variation in the weights leads to smaller design effects and larger effective sample sizes. For details about the NORC 2010 National Sampling Frame, please refer to the *Landmark Spirituality and Health Survey* *Sampling Plan (S. Pedlow, September 2012)*.

To achieve an equal-probability sample, we selected the first-stage (NFAs, or National Frame Areas) and second-stage units (which we refer to as segments) with probabilities proportional to size. This allows us to use our NORCSuite FrameTool Sample System to select equal-probability samples of housing units by equalizing the probabilities of selection at the third (housing unit) stage.

The 2010 NORC National Sample Design contains a total of 126 NFAs, but this many NFAs is only required for our largest national studies. For the Landmark Spirituality and Health Survey, we used a quarter-sample of these NFAs, which results in 44 NFAs. Of the 38 largest CSAs, eight are still certainty, but the remaining 30 are large enough to deserve 14 more selections. Table 1 shows the breakdown by NFA category.

| **Table 1. Summary of the First-Stage Units** | | | | |
| --- | --- | --- | --- | --- |
| **NFA Category** | **Description** | **Share of U.S. population** | **National Frame** | **Landmark** |
| 1 | The largest CSAs, certainty selections | 56% | 38 | 22 |
| 2A | DSF portions (8 or more tracts) | 30% | 60 | 15 |
| 2B | Tracts where listing is needed | 5% | 28 | 7 |
| 3 | Counties/CBSAs with less than 8 urban tracts | 9% |
| **TOTAL** |  | **100%** | **126** | **44** |

## NFAs in NFA Category 1may have more than eight segments. For the eight that are still certainty, we used one-quarter of the 431 segments (108). For the remaining 14 Category 1 NFAs, we used eight segments from each (112 total).

## For category 2A NFAs, the National Frame has 8 segments per first-stage selection and we used them all, resulting in 120 segments within the 15 NFAs. For category 2B/3 first-stage selections, the 2010 National Sample Design selected 5 segments per first-stage selection for cost efficiency, and we used all of them (7 * 5 = 35). Table 2 summarizes the number of segments in the 2010 NORC National Frame and the number that was used for this study.

| **Table 2. Summary of the Second-Stage Units** | | | | | |
| --- | --- | --- | --- | --- | --- |
| **Category** | **Expected Type of Address Listing** | **National Frame NFAs** | **Sample Segments** | **Landmark NFAs** | **Landmark Sample Segments** |
| **1** | Still Certainty | 8 | 431 | 8 | 108 |
|  | Now Non-Certainty | 30 | 465 | 14 | 112 |
| **2a** | City Style Address | 60 | 480 | 15 | 120 |
| **2b/3** | Rural | 28 | 138 | 7 | 35 |
| **TOTAL** |  |  | **1,514** | **44** | **375** |

1. **Selection of Housing Units**

NORC used proprietary software called FrameTool to select the housing units within the segments selected so that the resulting sample is an equal probability sample of housing units within the United States. The Study Design Outline indicates that the desired population of interest is the coterminous United States only (excluding Alaska and Hawaii). Alaska and Hawaii are represented in the full 2010 NORC National Sampling Frame, but none of the 44 NFAs we used for this study are in Alaska or Hawaii.

Within each household, up to two adults are selected at random. This results in an average of 1.59 eligible respondents per household, based on American Community Survey (ACS) data. To achieve 3,000 interviews regardless of age, a total of 5,132 housing units would be needed with assumed interview and screener response rates as well as housing unit and language eligibility rates. Table 3 below shows the derivation of the numbers.

| **Table 3. Sample Size to Achieve 3,000 Interviews (Two Adults per Household)** | | | |
| --- | --- | --- | --- |
| **Unit** | **Operation/ Sample Size** | **Rate** | **Number** |
| Housing Units Selected |  |  | 5,132 |
| Housing Unit Eligibility Rate | * | 86 percent |  |
| Number of Eligible Households | = |  | 4,414 |
| Language Eligibility Rate | * | 95 percent |  |
| Number of Language-Eligible Households | = |  | 4,193 |
| Screener Response Rate | * | 90 percent |  |
| Number of Screened Households | = |  | 3,774 |
| Number of Eligibles per Household | * | 1.59 |  |
| Number of Eligible Respondents | = |  | 6,000 |
| Interview Response Rate | * | 50 percent |  |
| Number of Respondents | = |  | 3,000 |

Based on the segments selected, we expect approximately 21.3 percent of respondents to be 65 years and older, 34.7 percent to be 18-40 years old and 43.9 percent to be 41-64 years old. Therefore, to achieve 1,000 respondents who are 65 years old and older, we need to increase the number of housing units selected and subsample the selected respondents who are younger than 65. These numbers are summarized in Table 4 below.

|  | **Table 4. Sample Size and Screening to Achieve 1,000 65+ Respondents** | | | | |
| --- | --- | --- | --- | --- | --- |
| **Unit** | | **Percentage of Respondents** | **Sample Size** | **Selection Rate** | **Sample Size** |
| Housing Units Selected | |  | 5,132 |  | 8,024 |
| Number of Respondents | |  | 3,000 |  | 3,000 |
| 18-40 year-old respondents | | 34.7 | 1,042 | 61.4 percent | 1,000 |
| 41-64 year-old respondents | | 43.9 | 1,318 | 48.5 percent | 1,000 |
| 65 years and older respondents | | 21.3 | 640 | 100 percent | 1,000 |

1. **Oversampling Segments with Higher 65+ Populations**

To reduce the total number of housing units selected in order to achieve 1,000 respondents 65 years and older and also to increase the subsampling rates for younger age groups, we tried to divide the segments in our sample into two groups based on the percentage of adults aged 65 and older. Specifically, the following steps were taken-

1. All 375 segments were sorted in descending order by percentage of adults aged 65 and older;
2. To divide the segments into HIGH and LOW groups, there are a total of 373 possible breakpoints. Sum up the number of housing units in the two groups under each scenario (n_high and n_low);
3. A probability factor x (x=2, 3, 4, 5) is applied to each scenario and n_newhigh and n_newlow are calculated such that n_newhigh/n_newlow=x*n_high/n_low;
4. Steps 2 and 3 above result in a total of 373*4=1,492 combinations;
5. For each combination, we calculated the number of interviews in each of the three age groups;
6. Using the number of interviews, we determined the number of housing units we need to select and the subsampling rates for the younger age groups;
7. The number of housing units that need to be selected is deducted from 8,024, the baseline of total housing units without oversampling. The difference of the two numbers is referred to as housing unit savings. For each probability factor, the breakpoint among all 373 that achieves the maximum housing unit saving is considered the optimal breakpoint;
8. Next, the design effect due to weighting (deff) and effective sample size (effective_n) associated with each optimal breakpoint is calculated.
9. Finally, cost of effective sample size (cost_sampsize) is calculated as (3,000-effective_n)/3,000.

Table 5 below summarizes the housing unit saving and design effect associated with each optimal scenario for probability factors 2, 3, 4 and 5 respectively.

**Table 5. Comparison of Optimal Oversampling Scenarios (Response Rate=50%)**

| **Base HU** | **prob_factor** | **Optimal breakpoint** | **HU** | **HU_saving** | **HU_saving_pct** | **deff** | **Cost_sampsize** |
| --- | --- | --- | --- | --- | --- | --- | --- |
| *8,024* | *2* | *121* | *7,087* | *937* | *11.7%* | *1.11* | *10.3%* |
| 8,024 | 3 | 102 | 6,611 | 1,413 | 17.6% | 1.28 | 21.8% |
| 8,024 | 4 | 85 | 6,299 | 1,725 | 21.5% | 1.42 | 29.7% |
| 8,024 | 5 | 73 | 6,069 | 1,955 | 24.4% | 1.55 | 35.4% |

As can be seen from Table 5 above, housing unit savings increase as the probability factor increases at the cost of effective sample size. Comparing the probability factor of 5 to the probability factor of 2, while the percentage of housing unit saving increase from 11.7 percent to 24.4 percent, the cost in effective sample size would more than triple. Therefore, we decided that the probability factor of 2 and breakpoint of 121 would be the optimal scenario. This means the top 121 segments in terms of percentage of adults aged 65 and older would be in the HIGH group. They would receive twice the selection probability as opposed to the rest of the segments, or the LOW group. The subsampling rates for the 18-40 and 41-64 age groups under this scenario are 73.95% and 54.64% respectively. These rates are higher than those in Table 4, making more efficient use of the sampled housing units. The total number of housing units selected is 7,087. Based on the ratio of n_newhigh/n_newlow of this final scenario, 3,514 housing units are to be selected from the HIGH group and 3,573 housing units are to be selected from the LOW group.

We classify a household as follows based on the age composition of adults in the household:

SENR-If there is at least one 65+ adult;

YNGA-If there are 0 adults 65+, but there is at least one adult 18-40; and

MDDL-If all the adults are 41-64 (or of unknown age).

Based on the result of subsampling analysis, the following subsampling rules were put forth for each type of household initially:

SENR- Select up to two adults at random;

YNGA-Activate up to two adults with 73.95% probability and activate ZERO adults with 26.05% probability (this household is subsampled out).

MDDL-Activate up to two adults with 54.64% probability and activate ZERO adults with 45.36% probability (this household is subsampled out).

During actual sample release however, we had to make some adjustment the subsampling rules in order to meet the targeted age distribution. This is detailed below.

1. **Sample Releases**

4.1 Samples for All Age Groups

Initially, 10,000 housing units were selected with FrameTool in February 2014. This includes 5,000 housing units from the 121 HIGH segments and 5,000 housing units from the 254 LOW segments. The sample was selected in replicates of 100 housing units each. The LOW sample replicates were numbered 101-150 and the HIGH sample replicates were numbered 201-250.

Release #1: The first batch of sample was released in February. This release consists of replicates 101-136 (LOW group) and 201-235 (HIGH group), which reflects the calculated sample size of 3,514 housing units from the HIGH group and 3,573 housing units from the LOW group under the optimal scenario. This initial release assumed a response rate of 50 percent.

The subsampling rules as described above were applied to each household type initially. In July 2014 however, due to the shortage of MDDL cases, a decision was made to turn the MDDL members that were subsampled out back on in YNGA/MDDL households in replicates 101-112 and 201-215 in the hope of getting 150 more MDDL interviews and also keeping the current ratio between the LOW and HIGH groups.

Cancelled Release #2: In May, we reduced our response rate assumption from 50% to 42% and released replicates 137-144 from LOW and 236-244 from HIGH as our next step with the exclusion of 5 NFAs that were to be closed for cost efficiency:

**Table 6. NFAs to Be Closed**

<redacted to prevent disclosure risk>

This release, however, was quickly pulled back with the exception of the SENR respondents.

4.2 Additional Release and Sample Draw for SENR Respondents

Release #3: In July, we decided to release all the remaining replicates from both HIGH and LOW groups in order to get 250 SNR-only completes from the new addresses. Therefore, this batch consists of replicates 137-150 (LOW group) and 236-250 (HIGH group).

For this release, the subsampling rate for YNGA and MDDL households was changed to 0. Within SENR households, up to two adults were selected at random.

In order to maximize the number of eligible SENR cases and to continue to target our goal of 1000 SENR completes, a decision was made in September to release more sample from the HIGH/SENR-likely segments. As the entire original sample of 10,000 housing units has already been released, an additional sample of 2,000 housing units was drawn from the HIGH segments. This additional sample was numbered as replicates 301 -500 with each replicate containing 10 housing units.

Release #4: In September, a fourth and last batch of sample was released from the additional sample. Based on the number of SENR completes we had yet to get at the time to reach our target and the production ratio of SENR completes in the original HIGH sample, we estimated that we needed roughly 637 housing units from the additional sample. Out of the 121 segments in the additional sample, 62 were areas in which we had no staff or plans for travel. These would be extremely difficult to work in the time we had left and would be categorized NIR. Therefore, we only released sample from the 59 remaining segments. The sample shows that we released replicates 301-444 within these 59 segments in order to get 637 housing units.

This sample followed the same selection rules as the third release: only SENR households were selected; Within SENR households, up to two adults were selected at random.

4.3 Activation of Un-selected SENR Members

In August, a decision was made to activate any SENR members that have not already been activated. That means we would activate every 65+ respondent we could find whether there are one, two, three, or four at one household. The idea was that every 65+ respondent has a probability of 1 of being selected within the selected household.

Table 7 below summarizes the sample source, release date, replicate numbers, number of cases released and subsampling rates for each of the releases.

**Table 7. Summary of Sample Releases**

| **Sample Source** | *Original*  *(n=10,000, incl. 5,000 from HIGH and 5,000 from LOW)* | | | *Additional*  *(n=2,000, all from HIGH)* |
| --- | --- | --- | --- | --- |
| **Release Batch** | #1 | #2 | #3 | #4 |
| **Release date** | February 2014 | May 2014 | July 2014 | September 2014 |
| **Replicate No.** | 101-136;  201-235 | 137-144;  236-244 | 137-150;  236-250 | 301-444 |
| **Number of**  **cases released** | 7,100 | This release was quickly pulled back. | 2,900 | 637 |
| **Subsampling Rates** | SENR: 1;  YNGA: 73.95%; MDDL: 54.64% | SENR: 1;  YNGA: 73.95%; MDDL: 54.64% | SENR: 1;  YNGA: 0;  MDDL: 0. | SENR: 1;  YNGA: 0;  MDDL: 0. |

1. **Weighting**

The following steps were followed to develop analysis weights for the LSHS sample. All selected households from the national frame received a base weight that reflects the probability of selection. An adjustment for household eligibility status was applied to the base weights to account for households with unknown eligibility. The base weights were further adjusted to account for screening nonresponse, subsampling, special cases that were completed in the pulled-back release, probability of selection for persons within a selected household, MDDL cases that were turned back on in subsampled out YNGA/MDDL households, main interview nonresponse, YNGA/MDDL member completes that were worked due to random error, and scaling to sample totals.

The details of the steps used to develop analysis weights are presented below.

- 1. Household Base Weight ($wt_{1}$)

All selected households received a base weight that reflects the probability of selection. For cases from LOW segments and highlighted HIGH segments (i.e. segments that have a chance of selection only in the original sample), the selection probability is the equal probability from FrameTool; for cases from un-highlighted HIGH segments, because they have two chances of selection (once in the original sample and once in the additional sample), their selection probability is greater. Therefore,

$$pr_{1}=\left\{\begin{matrix} equalprob\_low\text{ (.0000596361)}, & for LOW cases \\ equalprob\_high\text{ (.0001212745)}, & for HIGH cases\text{ in }62\text{ segments} \\ equalprob\_high+equalprob\_extra*144/200, & for HIGH cases\text{ in 59 segments} \end{matrix}\begin{matrix} \\ \\ \end{matrix}\right.$$

Equalprob_extra = .0000485099. By definition, the base weight $wt_{1}$ is the inverse of the probability of selection. The three different base weights are 16,768.37; 8,245.76; and 1,401.98. The sum of the base weights is 124,935,663, which is an estimate of the total number of U.S. housing units.

- 1. Adjustment for Household Eligibility ($wt_{2}$)

The base weights were further adjusted to account for household with unknown eligibility. The base weights associated with cases of unknown eligibility were distributed to cases for which the eligibility status is known. This adjustment preserves the weighted distribution of known eligible and known ineligible cases. Through this adjustment, part of the base weight total carried by the unknown eligibility cases is distributed to cases with known eligibility and known ineligibility, under the assumption that the eligibility rate among the two sets is the same. The adjustment cells were defined by the cross of NFA category (1, 2A, 2B and 3) and segment group (HIGH and LOW). The overall eligibility rate was 86.44 percent. Table 8 gives the eligibility rates by adjustment cell.

**Table 8. Eligibility Rates by Adjustment Cell**

|  | | 65+ Strata | |
| --- | --- | --- | --- |
|  |  | High | Low |
| NFA Type | Certainty | 84.83 percent | 89.53 percent |
|  | Large Urban | 88.78 percent | 89.45 percent |
|  | Other Urban | 92.16 percent | 89.76 percent |
|  | Rural | 76.99 percent | 94.35 percent |

- 1. Adjustment for Screener Nonresponse ($wt_{3}$)

The base weights for the complete screeners were adjusted to compensate for missing screeners identified as eligible. The adjustment cells were defined the same as in the previous step. Through this adjustment, the weight total associated with eligible non-complete screeners is transferred to the eligible screener completes. Eligible non-complete screeners are assigned adjusted weights of 0. The overall screener response rate was 77.33 percent. Table 9 gives the screener response rates by adjustment cell.

**Table 9. Screener Response Rates by Adjustment Cell**

|  | | 65+ Strata | |
| --- | --- | --- | --- |
|  |  | High | Low |
| NFA Type | Certainty | 72.86 percent | 76.24 percent |
|  | Large Urban | 74.01 percent | 76.87 percent |
|  | Other Urban | 80.42 percent | 80.22 percent |
|  | Rural | 77.35 percent | 76.33 percent |

- 1. Adjustment for Subsampling ($wt_{4}$)

Both YNGA and MDDL households were subject to subsampling during sample releases. The next step adjusts for subsampling.

- 1. Adjustment for Special Cases ($wt_{5}$)

As mentioned previously, the second batch of sample was pulled back shortly after it was released. During the time that the sample was in the field, we completed 28 YNGA and 26 MDDL interviews. Rather than waste these completed interviews, we decided to include these cases in delivery but down-weight them during weighting. These 54 cases come from 38 households, which include 21 households from 7 LOW replicates and 17 households from 9 HIGH replicates. Since these cases were completed early in the field before the sample was pulled back, our weighting strategy was to identify the “early” completes in the first batch of sample release (replicates 101-136 and 201-235) and re-distribute the weights associated with those early completes to all “early” cases, which includes the special cases.

$wt_{5}$is the final household weight. Only eligible households that have completed the screener have a positive weight$wt_{5}$. The weights now sum to the estimated number of eligible households in the United States (111,394,274).

- 1. Adjustment for Member Selection Probability ($wt_{6}$)

All persons within the eligible household have the same household weight. Once persons are selected, however, only the selected persons have a positive respondent weight. Within each household, the selection probability $pr_{2}$ is:

$$pr_{2}=\left\{\begin{matrix} 1, & for SENR members and MDDL re-release \\ \text{\# of non-SENR selected}/\text{/\# of total non-SENR members,} & \text{for selected} \text{YNGA/MDDL} \text{members} \\ \text{0,} & \text{for un-selected} \text{YNGA/MDDL} \text{members} \end{matrix}\right.$$

Then the respondent weight is:

- 1. Adjustment for MDDL Re-released Cases ($wt_{6\_2}$)

To account for the MDDL cases that were re-released in the subsampled out YNGA/MDDL households in replicates 101-112 and 201-215, we adjusted the member base weight ($wt_{6}$) of MDDL cases in YNGA/MDDL households by (1-SOR_original_)/(1-SOR_actual_), where SOR_original_ is the *original* subsampling-out rate for MDDL cases in YNGA/MDDL households from replicates 101-136 and 201-235 and SOR_original_ is the *actual* subsampling-out rate with the re-release taken into account. Therefore, for MDDL cases in YNGA/MDDL households,

$$wt_{6\_2}=wt_{6}\times(1-SOR_{original})/(1-SOR_{actual})$$

SOR_original_ = .2896 and SOR_actual_ = .1865, so roughly .1865/.2896 = 64.40 percent of the subsampled out MIDL cases were re-released. The adjustment to the weights above is .8733. For SENR and YNGA cases, and MDDL cases from SENR households, $wt_{6\_2}$is the same as$wt_{6}$because these cases were not affected by the MDDL re-release.

- 1. Adjustment for Main Interview Nonresponse ($wt_{7}$)

To compensate for unit nonresponse, we next adjusted respondent weights within nonresponse adjustment classes. Adjustment classes were defined by age category (YNGA, MDDL, SENR). Within each adjustment class, the weight total associated with eligible non-respondents is transferred to the selected eligible persons who actually completed the interviews. Only respondents to the main interview have a positive weight $wt_{7}$. Overall, the interview response rate was 51.62 percent (56.31 for YNGA respondents, 48.06 percent for MIDL respondents, and 49.43 percent for SENR respondents)

- 1. Adjustment for Completed Cases due to Random Error ($wt_{7\_2}$)

Two types of random error occurred during data collection: (1) Some YNGA/MDDL households from SENR-only replicates were accidentally worked that was *not* part of the pulled-back replicates; and (2) Some YNGA/MDDL members randomly selected from SENR households in the SENR-only releases were not sent to Expected Surplus. Together, these two groups of households have produced a total of 42 completes. Instead of discarding these 42 cases, we have decided to assign the minimum weight within the main interview adjustment class to these cases. For the 28 MIDL respondents, this minimum weight is 17,973.93. For the 14 YNGA respondents, this minimum weight is 15,340.4. This step does not affect the weight for the rest of the cases.

- 1. Scale Adjustment ($wt_{8}$)

As a final step, $wt_{7\_2}$ are scaled so that they sum to the final sample size of 3,010.

$$wt_{8}=\frac{3,010}{\sum wt_{7\_2}}wt_{7\_2}$$

$wt_{8}$ is the final member weight. The sum of the $wt_{7\_2}$weights is 176,120,761, so the adjustment factor 3010/176120761 = .0000170905. Table 10 is a final summary of the final respondent weights, which makes clear the oversampling of SENR respondents:

**Table 10. Summary of the Final Respondent Weights**

| Age Category | Sample Size | Sum of Weights |
| --- | --- | --- |
| YNGA | 1,000 | 1,204.44 |
| MIDL | 1,002 | 1,245.21 |
| SENR | 1,008 | 560.35 |
| **TOTAL** | **3,010** | **3,010.00** |
